# Recurrent somatic mutations reveal new insights into consequences of mutagenic processes in cancer

**DOI:** 10.1371/journal.pcbi.1007496

**Published:** 2019-11-25

**Authors:** Miranda D. Stobbe, Gian A. Thun, Andrea Diéguez-Docampo, Meritxell Oliva, Justin P. Whalley, Emanuele Raineri, Ivo G. Gut

**Affiliations:** 1 CNAG-CRG, Centre for Genomic Regulation (CRG), The Barcelona Institute of Science and Technology, Barcelona, Spain; 2 Universitat Pompeu Fabra (UPF), Barcelona, Spain; Carnegie Mellon University, UNITED STATES

## Abstract

The sheer size of the human genome makes it improbable that identical somatic mutations at the exact same position are observed in multiple tumours solely by chance. The scarcity of cancer driver mutations also precludes positive selection as the sole explanation. Therefore, recurrent mutations may be highly informative of characteristics of mutational processes. To explore the potential, we use recurrence as a starting point to cluster >2,500 whole genomes of a pan-cancer cohort. We describe each genome with 13 recurrence-based and 29 general mutational features. Using principal component analysis we reduce the dimensionality and create independent features. We apply hierarchical clustering to the first 18 principal components followed by k-means clustering. We show that the resulting 16 clusters capture clinically relevant cancer phenotypes. High levels of recurrent substitutions separate the clusters that we link to UV-light exposure and deregulated activity of POLE from the one representing defective mismatch repair, which shows high levels of recurrent insertions/deletions. Recurrence of both mutation types characterizes cancer genomes with somatic hypermutation of immunoglobulin genes and the cluster of genomes exposed to gastric acid. Low levels of recurrence are observed for the cluster where tobacco-smoke exposure induces mutagenesis and the one linked to increased activity of cytidine deaminases. Notably, the majority of substitutions are recurrent in a single tumour type, while recurrent insertions/deletions point to shared processes between tumour types. Recurrence also reveals susceptible sequence motifs, including TT[C>A]TTT and AAC[T>G]T for the POLE and ‘gastric-acid exposure’ clusters, respectively. Moreover, we refine knowledge of mutagenesis, including increased C/G deletion levels in general for lung tumours and specifically in midsize homopolymer sequence contexts for microsatellite instable tumours. Our findings are an important step towards the development of a generic cancer diagnostic test for clinical practice based on whole-genome sequencing that could replace multiple diagnostics currently in use.

## Author summary

Mutations found in the DNA of a tumour are expected to be largely unique to each tumour as there are three billion places in the DNA that can be mutated. However, despite these odds, in a cancer study with 2,583 participants covering 37 tumour types we observe in total over a million non-unique mutations. Based on this observation, we hypothesize that these mutations can be highly informative of the biological processes that caused them. Using characteristics of these non-unique mutations and general statistics like the total number of mutations, we classify the tumours into 16 groups. These groups not only delineate various mutational processes, but also characterize them in more detail. Moreover, we can link the groups to several clinically actionable phenotypes. Our work is a crucial step towards the development of a generic and personalized cancer diagnostic test that only uses the mutations found in the tumour.

## Introduction

Mutational processes induced by exogenous sources and/or endogenous mechanisms determine the mutational burden of a cell. They each leave their own genomic fingerprint that differs in terms of the number, types and distribution of mutations. Cancer cells usually show higher mutation rates than normal cells due to elevated cell proliferation and lack of proper DNA repair. The mutations accumulated before, during and after the oncogenic transformation may result in a mutational load exceeding several thousand per cancer genome [1]. Even with such a high burden, the sheer size of the human genome with over three billion bp still makes it improbable that by chance alone identical somatic mutations are found at exactly the same genomic location in two or more cancer patients. Such mutations we will henceforth refer to as being ‘recurrent’. Positive selection is one possible explanation for the recurrence of mutations. Recurrent mutations or often more general, recurrently mutated genes and regulatory elements, are used in the prediction of cancer drivers that provide a growth advantage to the cell [2]. However, the number of mutations per cancer genome that so far has been identified as being under positive selection is very small [3, 4] and the discussion on what are sufficient conditions for driver mutations to cause cancer is on-going [5, 6]. Instead of focusing on driver mutations, we hypothesize that recurrent mutations may be highly informative of the non-randomness of mutagenesis and provide a different way to group cancer genomes. In support of this, at both megabase as well as local scale cancer-specific patterns of the non-random distribution of mutations have been well described [7]. For instance, mutation rate is influenced by replication time [8], is linked to epigenomic features [9], shows a periodic pattern around nucleosomes [10], and can depend strongly on the 5’ and 3’ flanking base as shown in mutational signatures for several mutational processes [11]. This enrichment of mutations in specific genomic regions or sequence contexts increases the probability of recurrence as does the number of mutations per sample, which also varies across mutagenic processes.

We use recurrence as a starting point for a systematic analysis of cancer genomes from the Pan-Cancer Analysis of Whole Genomes (PCAWG) consortium [12]. This cohort study, brought together by an initiative of the International Cancer Genome Consortium (ICGC) and The Cancer Genome Atlas (TCGA), covers 37 tumour types from 2,583 donors (S1 Table) and is the largest publicly available dataset of its kind. It allows a comprehensive pan-cancer analysis of recurrence in particular since the somatic mutation calling pipeline was identical across all genomes. Moreover, the whole-genome sequencing data that is available for all donors provides a more complete view than whole-exome sequencing data that so far has been used for large-scale pan-cancer analyses [13]. To make full use of the whole-genome sequencing data and analyse recurrence in an unbiased way, we take here a purely data driven approach that is independent of the completeness and correctness of current genome annotations. Hereby we will focus on Somatic Single-base Mutations (SSMs) and Somatic Insertion/deletion Mutations (SIMs). We first confirm that the number of recurrent mutations is far higher than expected by chance alone and shed light on the relationship between recurrence and the number of samples. Next, we analyse recurrence in the context of general mutational characteristics that capture the effect of mutational processes on the genome. Finally, these general features together with recurrence-related features form the base for clustering cancer genomes in a novel way and determine what recurrence can tell us about mutagenesis. To help interpret the recurrence observed in the 16 identified clusters, link clusters to potential mutational processes and provide further details of each cluster, we use various types of metadata, including tumour type information, driver predictions, and replication time. As a result, we are not only able to refine the mutational consequences of many exposure-specific processes, but also capture clinically relevant phenotypes by using hitherto unused, but easily obtainable mutational features from whole-genome sequences.

## Results

### Recurrence is higher than expected by chance

There are 1,057,935 recurrent SSMs, which represent 2.44% of the total number of SSMs found in the PCAWG cohort. This is around five times higher (Fig A-I in S1 Text) than expected if only chance would drive recurrence (based on 5,000 simulations, S1 Text). For the six SSM subtypes (see Methods) the observed recurrence is around three (C>G and T>C SSMs) to twelve times (T>G SSMs) higher than expected by chance (Fig A-II in S1 Text). On tumour type level, we can either determine recurrence by only considering the samples from the same tumour type (‘within tumour type’) or across all samples (‘pan-cancer’). In both cases, Kidney-RCC, Liver-HCC, Lung-AdenoCA and Lung-SCC stand out as the observed number of recurrent SSMs is only around three times (within tumour type) and around two times (pan-cancer) higher than expected by chance (Fig A-III+IV in S1 Text). In contrast, the largest ratio is 86 times for recurrence ‘within tumour type’ (Prost-AdenoCA) and 7 times for recurrence ‘pan-cancer’ (Eso-AdenoCA).

### Number of samples does not always correspond to the level of recurrence

**Fig 1:**
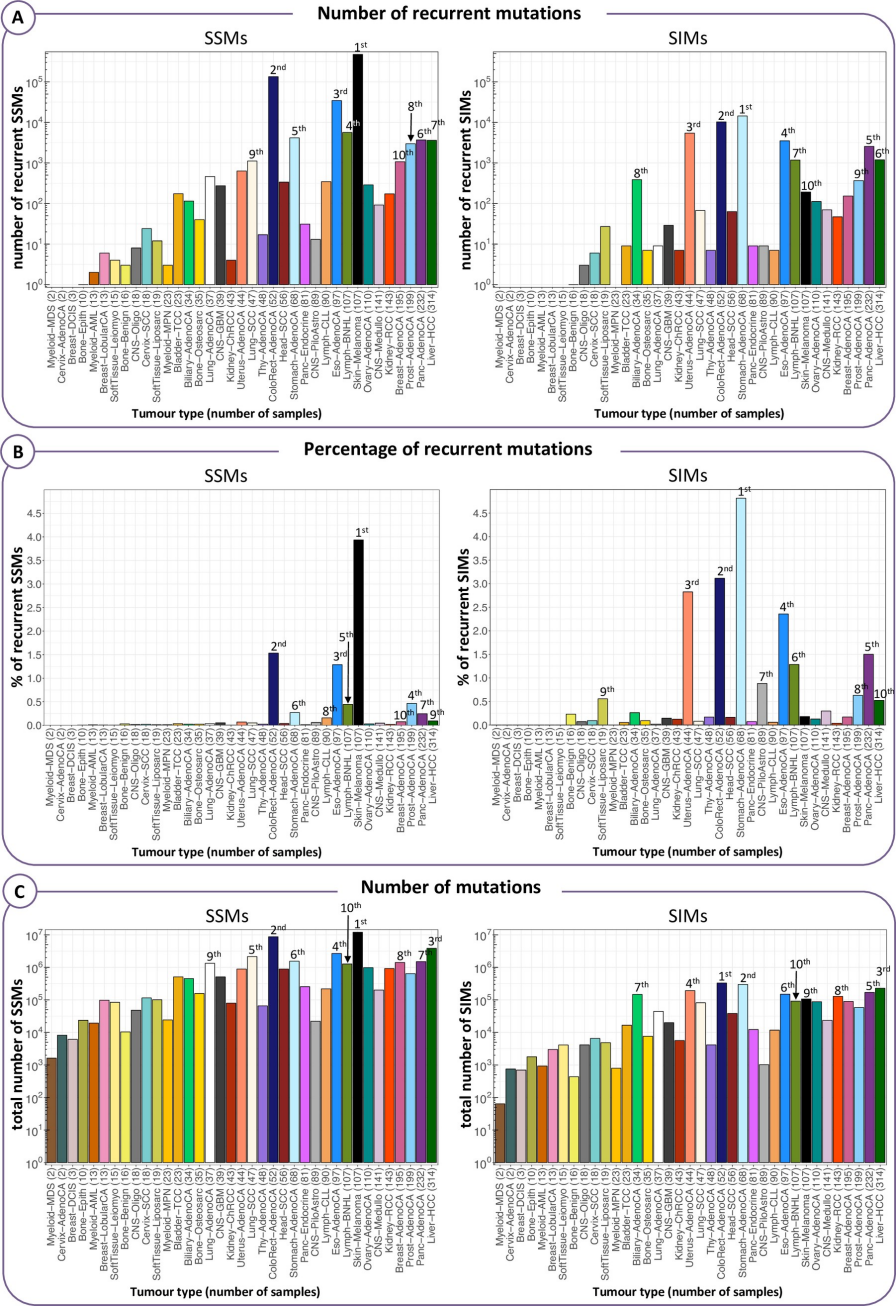
Recurrence within each tumour type in absolute numbers and percentages. The tumour types are ordered from the lowest to the highest number of samples. We labelled the top 10 ranking tumour types in terms of the following three values: (A) Absolute number of recurrent mutations, where recurrence is defined by considering each tumour type separately (‘within tumour type’ recurrence). (B) Percentage of recurrent mutations ‘within tumour type’. (C) Total number of mutations, counting recurrent mutations only once.

To see the effect of the number of samples on recurrence, we look at the overall recurrence within each tumour type (Fig 1). Although tumour types with more samples generally have a higher total number of recurrent mutations (Fig 1A), there are notable exceptions. For example, Liver-HCC has the most samples of all tumour types (314), but less recurrent SSMs and SIMs than six and five other tumour types, respectively. If we look at the percentage of recurrent mutations, even more tumour types overtake Liver-HCC as in this respect it ranks 9^th^ and 10^th^ in terms of SSMs and SIMs, respectively (Fig 1B). The opposite is true for Eso-AdenoCA (97 samples), which has a higher absolute number and percentage of recurrent SSMs than eight other tumour types that have more samples. Stomach-AdenoCA has the highest absolute number and percentage of recurrent SIMs of all tumour types, but less samples than 13 of them. One partial explanation for this is that a lower number of samples does not always translate to a lower total number of mutations (Fig 1C), even though the correlation is strong (Spearman's Rank correlation coefficient *r*_*S*_ = 0.73, p = 2.8e-07). However, even if the number of samples and the number of mutations are in line, the level of recurrence may still give a different picture. Liver-HCC, for instance, has also a higher total mutational load than Eso-AdenoCA (1.2·10^6^ and 7.9·10^4^ more SSMs and SIMs, respectively), but still a lower level of recurrence.

### General mutational characteristics versus recurrence

For each cancer genome, we compute 29 basic mutational characteristics that capture the effects of mutagenesis (*e*.*g*. relative frequency of each SSM subtype) and 13 features capturing recurrence at different levels (Table A in S1 File, see Methods). Recurrence for these features is determined based on the entire cohort. A detailed description of each of these 42 measures is available in S1 File. Based on the comparison of the recurrence-related features with the general ones (S2 Text), the key findings are that across the entire cohort: 1) the correlation between mutational load and the absolute level of recurrence is stronger for SSMs (*r*_*S*_ = 0.89) than for SIMs (*r*_*S*_ = 0.76); 2) the same correlation, but instead taking the percentage of recurrent mutations, is weak and negative for SSMs (*r*_*S*_ = -0.21) and non-significant for SIMs; 3) relative recurrence for SIMs is higher than for SSMs; 4) a particularly high percentage of C>T SSMs and 1 bp A/T deletions are recurrent (4.19% and 15.27%, respectively); 5) there is a strong tendency for T>G SSMs to be recurrent despite its modest total number; 6) there is a strong correlation between the level of recurrence for SIMs and the percentage of 1 bp SIMs in a long homopolymer context. Looking into the different tumour types, there are clear contrasts in terms of the associations between general and recurrence-related characteristics. For example, there is a statistically significant positive correlation between the number of mutations and the percentage recurrent for only two tumour types in the case of SSMs (Eso-AdenoCA: *r*_*S*_ = 0.48 and Skin-Melanoma: *r*_*S*_ = 0.58) and for seven types with respect to SIMs (most notably: Biliary-AdenoCA: *r*_*S*_ = 0.71 and Eso-AdenoCA: *r*_*S*_ = 0.67) (Fig D in S2 Text).

### Recurrence characteristics divide the cohort

**Fig 2:**
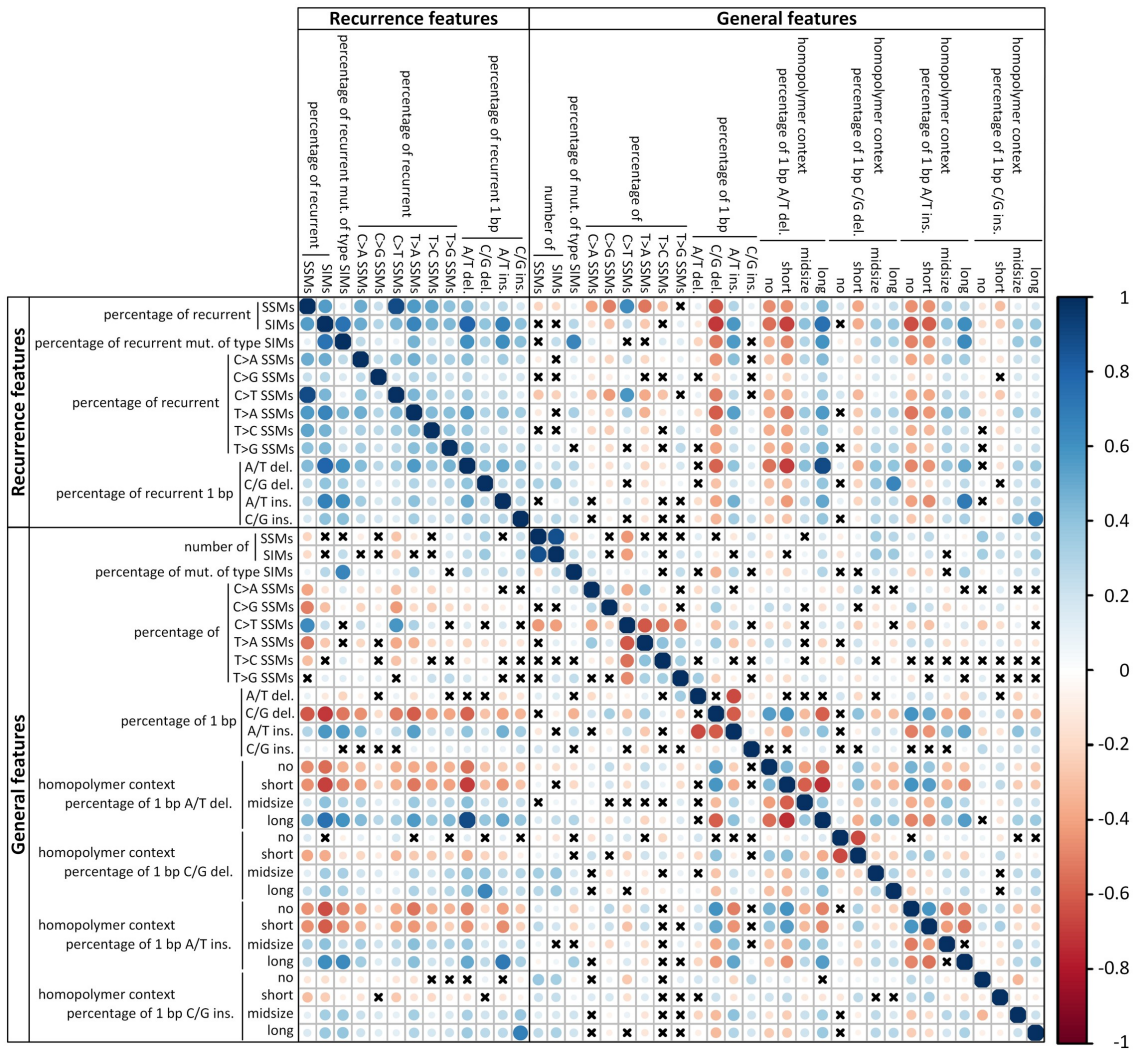
Spearman’s rank correlation between the 42 mutational features. The colour of the circles indicate positive (blue) and negative (red) correlations, colour intensity represents correlation strength as measured by the Spearman’s rank correlation coefficient. The size of the circle indicates the adjusted p-value with larger circles corresponding to lower p-values. The p-values were corrected for multiple testing using the Benjamini-Yekutieli method. Crosses indicate that the correlation is not significant (adjusted p-value > 0.05).

**Fig 3:**
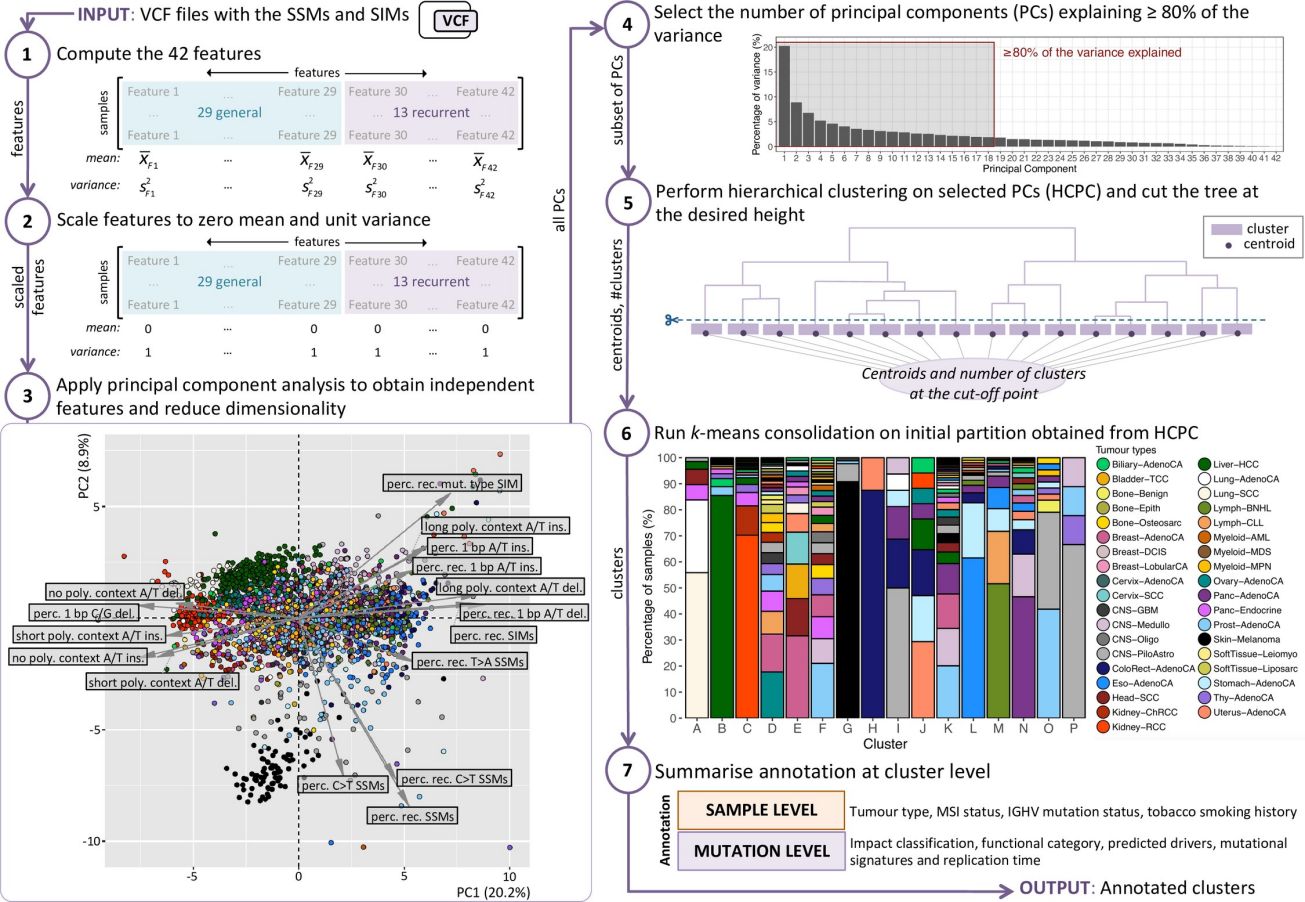
Workflow of the recurrence-based approach to group cancer genomes. The 42 features are described in detail in S1 File (Step 1). We scale all features to zero mean and unit variance to compensate for the differences between the ranges of the features (Step 2). The arrows in the PCA plot indicate the direction and level of contribution of the features that contribute above average to the first two PCs (Step 3). Seven of these features are related to recurrence. An interactive 3D version of the PCA plot is available here: https://plot.ly/~biomedicalGenomicsCNAG/1.embed. We take a subset of the PCs and consider the remaining PCs to capture noise (Step 4). For the hierarchical clustering we use the Euclidean distance as a dissimilarity measure and Ward’s method as the linkage criterion (Step 5). The results of the hierarchical clustering are used as a starting point for k-means clustering (Step 6). Some samples will in this step switch to a different cluster compared to the initial partition. This consolidation step is repeated a maximum of 10 times. Further details on the annotation of the clusters (Step 7) are described in S3 Text.

Next, we use the recurrence-based and general mutational features all together to see if we can uncover meaningful clusters of cancer genomes. As there are strong correlations between some of these features (Fig 2), we first perform a principal component analysis (PCA) to obtain independent features and reduce dimensionality (Fig 3). We take as many principal components (PCs) as needed to explain at least 80% of the variance in the data and consider the remaining PCs to capture noise. We use this subset of PCs as input for hierarchical clustering [14]. The resulting hierarchical tree is cut at the desired height to obtain clusters. The centroids are computed for each cluster and used as input to the k-means consolidation step, which further improves the initial clustering (see Methods) [15]. To get a pan-cancer perspective we analyse all samples together.

**Fig 4:**
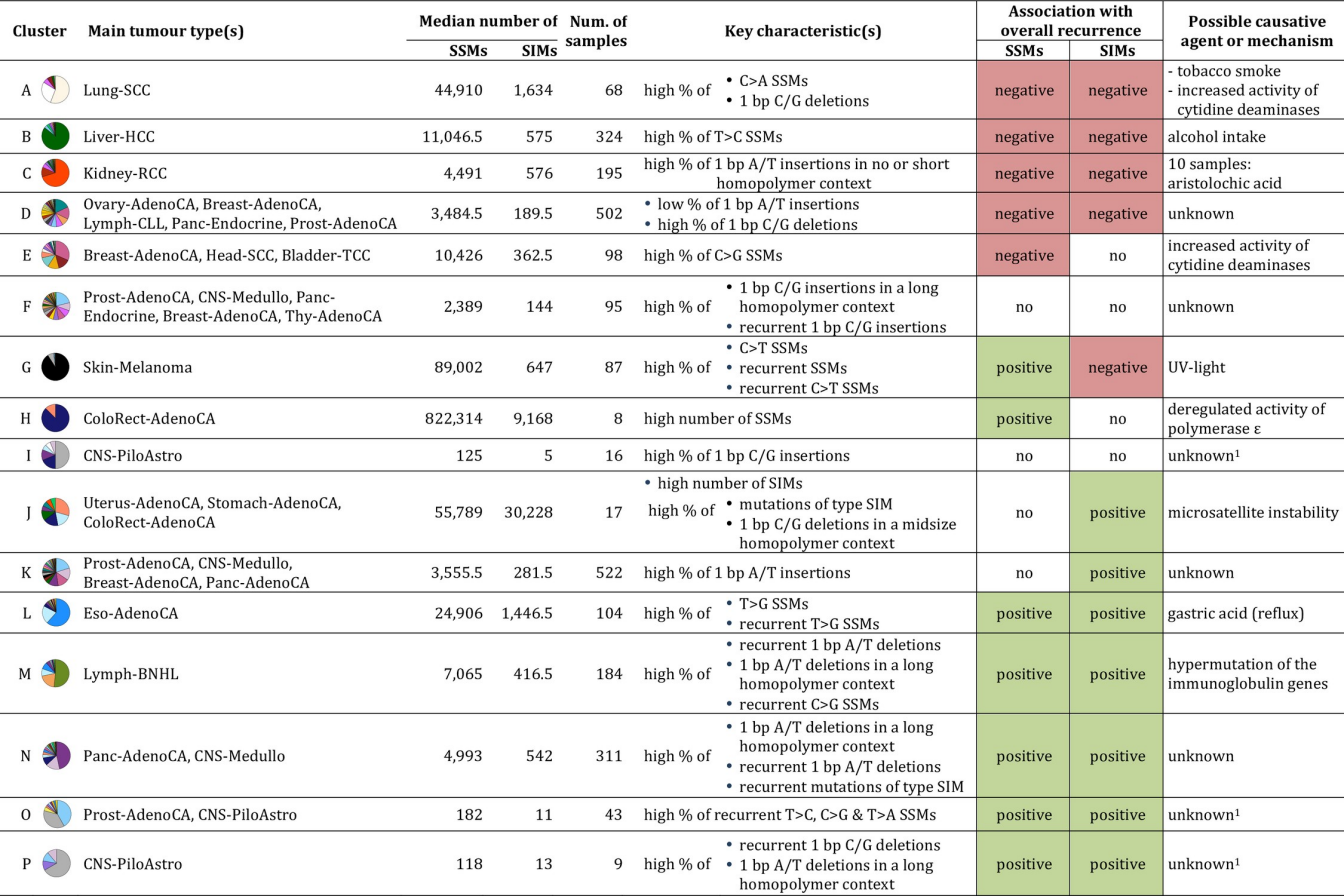
Key characteristics of the 16 clusters. Tumour types that form together ≥50% of the cluster are listed. The legend for colours for the pie chart is provided in Fig 3. The key characteristics are those features with the strongest significantly negative or positive association with the cluster. Only if the association with overall recurrence is significant, the respective direction is indicated. ^1^Cluster has a low median number of SSMs (<200) and SIMs (<20).

**Fig 5:**
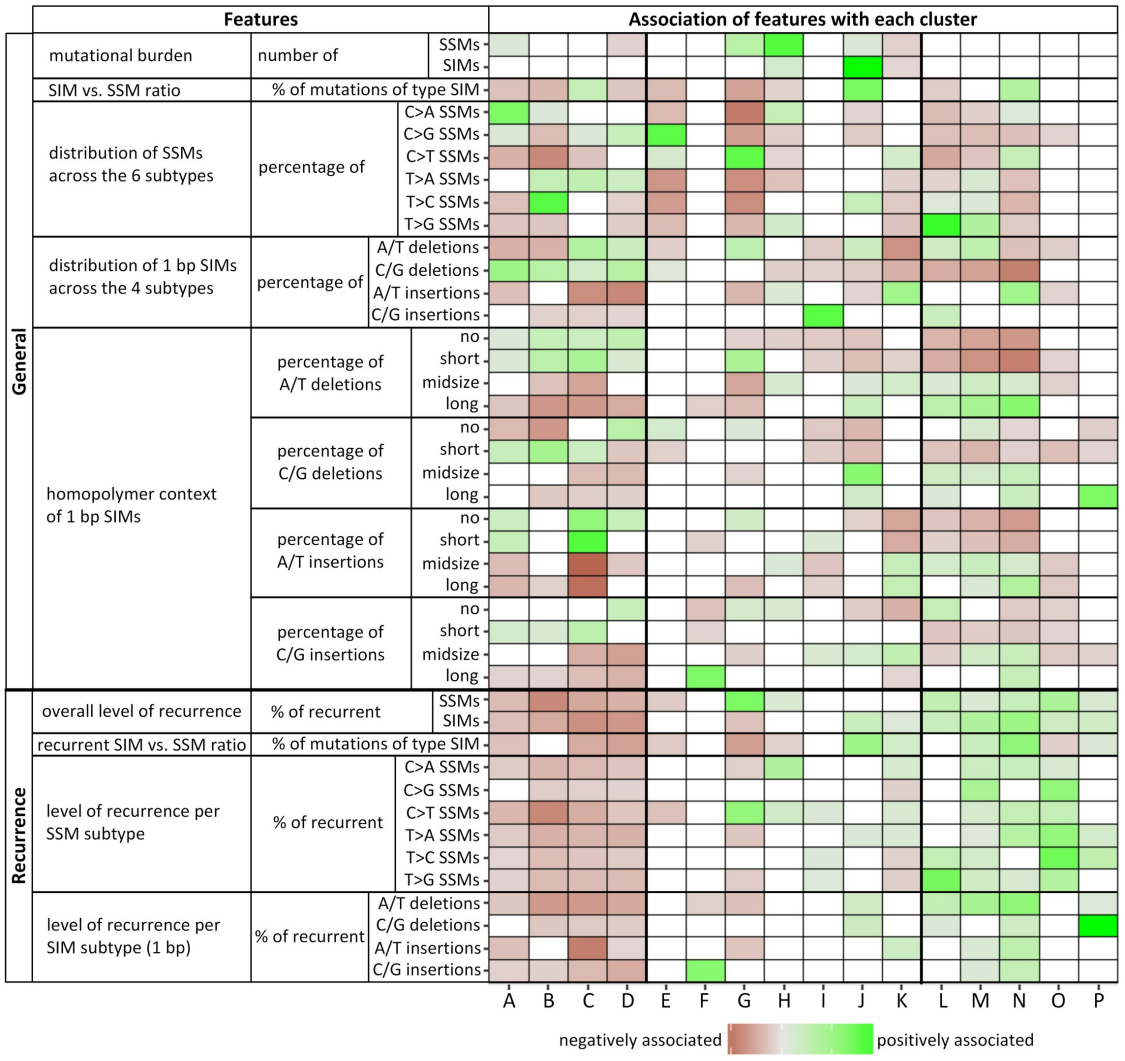
Overview of the 42 features and their association with each cluster. Red and green squares indicate statistically significant negative and positive associations, respectively, where the gradient indicates the strength of the association. White coloured squares indicate no significant association (adjusted p-value > 0.05). For deletions a ‘no homopolymer context’ means that the base next to the deleted one is not of the same type. For insertions this refers to a base inserted 5’ to either a base of a different type or a single base of the same type. Note that we do not have to consider preceding bases as all SIM calls were left aligned. A short homopolymer context is defined as a 2–4 bp mononucleotide repeat of the same type of base as the 1 bp SIM, midsize is 5–7 bp in length and long ≥ 8 bp.

The crude division into two clusters separates the cancer genomes with low relative levels of recurrent SIMs (*e*.*g*. Liver-HCC, Kidney-RCC and Lung-SCC) from those with high levels (*e*.*g*. ColoRect-AdenoCA, Eso-AdenoCA, Lymph-BNHL and Panc-AdenoCA) (S1 Fig). At three clusters, the relative level of recurrent SSMs splits off a group of mainly Skin-Melanoma samples from the two other clusters. This cluster largely remains unchanged when increasing the number of clusters while the two other clusters continue to divide and become more specific to a tumour type or a particular mutational process. At the level of six clusters, for example, we see a cluster split off that we can connect to microsatellite instability (MSI). We will discuss in further detail the division into 16 clusters, chosen as a trade-off between too many clusters, which would each be specific to just a handful of samples, and too few, which would result in loss of meaningful information (Fig 4). There are nine clusters (A, B, C, G, H, I, L, M and P) for which at least half of the samples are from the same tumour type. For another two clusters (O and N) samples from two tumour types constitute a majority. In the remaining five clusters (D, E, F, J and K) three or more tumour types are required for this. For each tumour type the percentage of samples in each of the 16 clusters is shown in S2 File. The association of each of the 42 features with the clusters is shown in Fig 5. The key characteristics of each cluster are shown in Fig 4. To facilitate a tight linkage of the clusters to mutational processes, we consider, in addition to the mutational features of a cancer genome, also tumour type assignment, microsatellite instability (MSI) status, immunoglobulin heavy-chain variable region gene (IGHV) mutation status (Lymph-CLL only) and tobacco smoking history of the donor (where available) (S3 Text). To provide further details on each cluster we integrate annotation based on GENCODE [16], Oncotator [17], driver predictions [3, 18], replication time [19] and mutational signatures [20]. A summary of this and further details are described in S3 Text. In the following sections we will show how the level of recurrence can be indicative of the mutational processes, often in combination with the general features. Moreover, we show that our recurrence-based approach groups cancer genomes in a novel way that complements current classification approaches and captures clinically actionable cancer phenotypes.

### High levels of recurrent SSMs and low levels of recurrent SIMs characterize exposure to UV light

**Fig 6:**
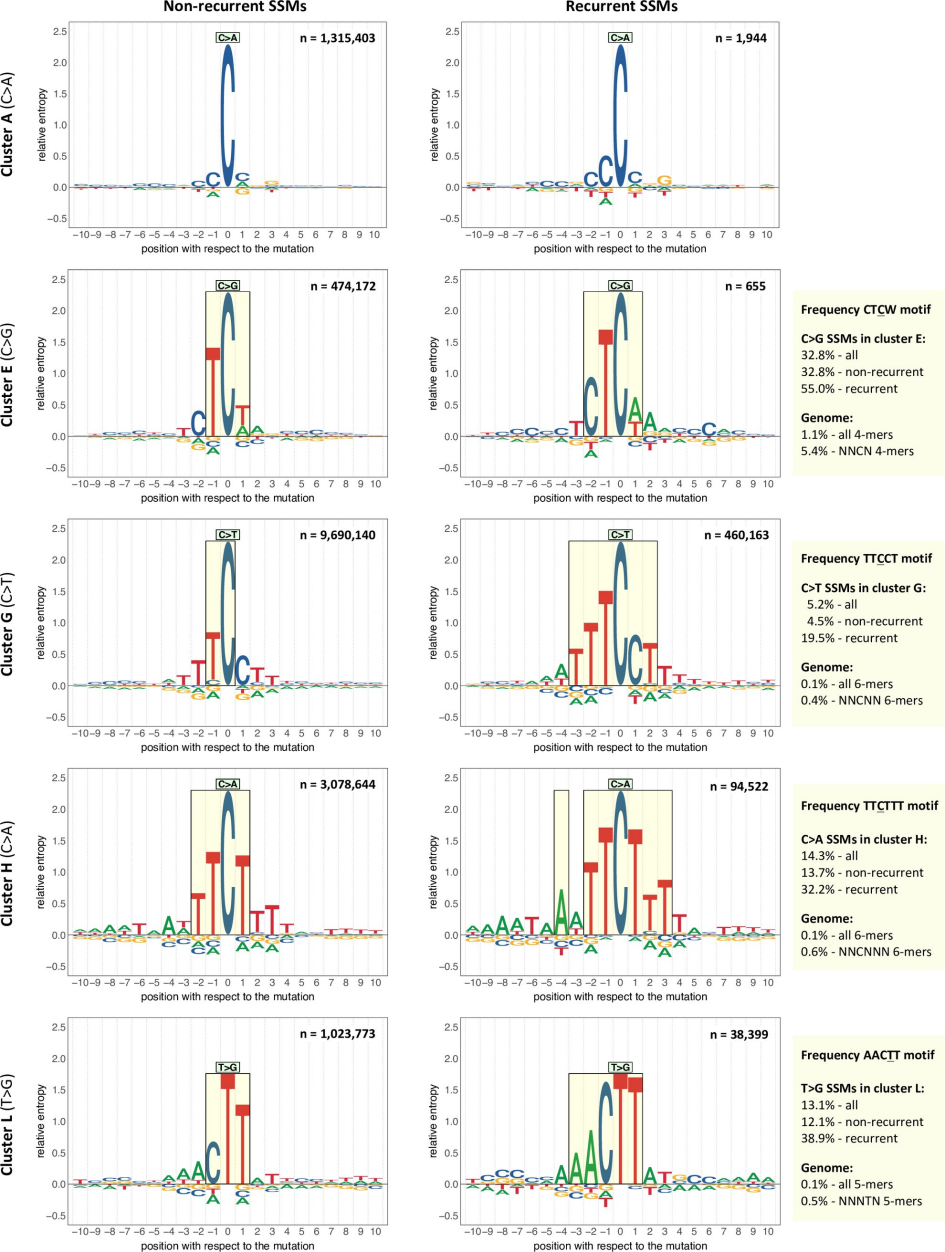
Enriched sequence motifs. The sequence logos represent the sequence context of ten bp 5’ and 3’ of the non-recurrent (left-side) or recurrent (right-side) mutations of the indicated cluster and SSM subtype. Here recurrence is defined as a mutation at the same genomic location in two or more samples from the same cluster. Each recurrent SSM is included only once to avoid giving extra weight to highly recurrent mutations. Relative entropy is used as a measure of information content (see Methods). Setting a threshold of 0.25 for the relative entropy results in the motifs highlighted in the rectangles. In the upper right corner of each sequence logo the number of mutations is indicated. To the right of the sequence logos are the percentages in which the enriched motif found for the recurrent SSMs is present in context of the mutations in the cluster and the corresponding k-mers in the genome (N = A, C, G or T). The enrichment for the motif for recurrent SSMs is in all four cases significantly higher than for the non-recurrent SSMs (χ^2^ test: p<2.2e-16).

A positive association with overall recurrence of SSMs and more specifically with recurrence of C>T SSMs characterizes cluster G that mainly consists of Skin-Melanoma samples (Fig 5). The association is negative with the recurrence of SIMs. We link this cluster to mutagenesis induced by UV light (S3 Text). The samples assigned to cluster G account by themselves for 60.7% of the total number of recurrent C>T SSMs. The combination of the high total number of SSMs per sample and the high percentage of C>T substitutions in this cluster is what contributes to the high level of recurrence. The mechanisms inherent to UV-light exposure further increase the probability of recurrence as it tends to result in C>T SSMs near energy sinks in the genome. The energy from UV-light-exposed DNA usually travels along the DNA strand until it arrives at the lowest energy point, a dT, particularly when it is next to a dC, which acts as energy barrier [21]. In agreement with this, for C>T mutations that are recurrent within this cluster there is a strong enrichment of a TTTCCT motif (the underlined C is mutated) (see Methods). While the percentage of this motif in the genome is estimated to be only 0.4% of all 6-mers with a C at the central position, 4.5% and 19.5% of the non-recurrent and recurrent C>T SSMs, respectively, within this cluster are at this motif (Fig 6). An enrichment of a CTTCCG motif was found for frequently recurrent mutations in promoters in 38 melanoma samples [22]. In another set of 184 melanoma samples a CTTCCGG motif was found at the majority of ETS transcription factor binding sites (TFBSs) [23]. As the sequences are centred at the core consensus ETS binding motif TTCC, instead of at a mutation, the underlined nucleotide is the most frequently mutated base. In a subset of highly mutable ETS TFBSs the second C is the most mutated. These and our specific sequence motif help explain the observed high level of recurrence. Furthermore, a decreased activity level of the nucleotide excision repair pathway was detected in melanoma at active transcription factor binding sites and nucleosome embedded DNA compared to the flanking sites [24]. This increases local mutation rates and hence also increases the probability of recurrence.

### High levels of recurrent SSMs characterize deregulated activity of POLE

A high level of recurrent SSMs also characterizes cluster H, specifically C>T and C>A SSMs. This cluster captures samples that can be considered ultra-hypermutators and their mutations are mainly caused by deregulated activity of POLE (S3 Text). These samples have a very high total number of C>A SSMs (median: 297,750) and the median percentage of recurrent C>A SSMs across the samples is 7.7%. Two thirds of all recurrent C>A SSMs in the entire cohort are also recurrent within only this cluster. The C>A mutations that are recurrent within this cluster are enriched for the motif TTCTTT, when considering only ungapped motifs (Fig 6, see Methods). Of the recurrent C>A SSMs 32.2% are at this motif, while for non-recurrent ones this is true for only 13.7% (χ^2^ test: p<2.2e-16). In the genome, the estimated percentage of this motif of all corresponding 6-mers (NNCNNN) is far smaller (0.6%), suggesting that effects of deregulated activity of POLE are most likely dependent on a sequence context exceeding a single neighbouring base on each side as also observed for whole-exome data by Martincorena *et al*. [25].

### High levels of recurrent SIMs characterize microsatellite instability

The highest level of recurrent SIMs across all clusters is observed for cluster J, which could be linked to a defective mismatch repair (MMR) pathway resulting in MSI (S3 Text). Of the 179,691 recurrent 1 bp SIMs in the entire cohort, almost half of them are recurrent when only considering this cluster. The very high median number of SIMs (30,228) in this cluster may play a role in the high level of recurrence. The key factor, however, is most likely the mutational process behind MSI, which is slipping of the DNA polymerase during replication of repetitive sequences and the lack of repair by the MMR pathway [26]. This not only explains the elevated number of SIMs [27], but also the association of this cluster with all SIM subtypes in the context of midsize-to-long homopolymers. As such homopolymers are scarce in the genome, the shift towards specifically altering them increases the probability of recurrence (Table F in S2 Text). Especially striking in this cluster is the proportion of 1 bp C/G deletions that are in the context of a midsize homopolymer (median: 73.2% vs. 8.4% for the other clusters combined, p = 1.2e-12). This translates to 6.0% recurrent 1 bp C/G deletions within this cluster versus <0.7% for any other cluster (S3 Text).

### Positive association with recurrence of SSMs and SIMs: Gastric-acid exposure and hypermutation of immunoglobulin genes

Clusters L, M and N all positively associate with recurrence of both SSMs and SIMs. Cluster L, which for >80% consists of Eso-AdenoCA and Stomach-AdenoCA samples, can potentially be linked to gastric-acid exposure (S3 Text). The T>G and T>C SSMs that are recurrent within this cluster cover 45% and ~20%, respectively, of the total observed in the whole cohort. The median percentage of SSMs falling in late-replicating regions (Table C and Fig A in S3 Text) is significantly higher than in the rest of the clusters combined (75.2% vs. 61.0%, p<2.2e-16). In general, the mutational load is expected to be higher in late-replicating regions as the MMR pathway is said to be less efficient there [28]. However, the question is why the effect is so strong in cluster L compared to the others (Fig B in S3 Text). It could be that transient single strand-DNA at stalled replication forks, whose formation has been suggested to be more prevalent in late-replicating regions [29], is particularly vulnerable to the mutagenicity of acid-exposure. Alternatively, if the oxidative stress induced by gastric-acid exposure leads to the oxidation of dG in the dNTP pool [30], the use of error-prone DNA polymerases that incorporate the oxidized dG into the DNA [31] may be more frequent in late-replicating regions [32]. The strong shift towards late-replicating regions favours higher levels of recurrence. The same holds for the enrichment of the specific sequence context ‘AACTT’ that we observe for T>G mutations that are recurrent within this cluster (Fig 6, see Methods). Nearly 39% of the recurrent T>G SSMs are confined to this motif and ~12% of the non-recurrent ones (χ^2^ test: p<2.2e-16), which is still far higher than the estimated percentage of this motif in the genome (0.5% of all NNNTN 5-mers). For SIMs, the cluster has a positive association with recurrence for three out of the four SIM subtypes as well as with the same subtypes in a midsize and/or long homopolymer context. This suggests similar mechanisms as for cluster J. Finally, as observed for SSMs in this cluster, SIMs also show a tendency to fall into late-replicating regions (67.2%, Table C and Fig C in S3 Text). This may further add to the high level of recurrence for SIMs.

Cluster M, with mainly Lymph-BNHL and Lymph-CLL samples, is linked to the somatic hypermutation of the immunoglobulin genes (S3 Text). In the aforementioned tumour types, this process is indicative of memory B cells being the cell of origin as opposed to naïve B cells [33]. The cluster has positive associations with the level of recurrence for all six SSM subtypes. The association is particularly strong for C>G. Of all recurrent C>G SSMs, 10.7% can be found in this cluster alone. The high level of recurrence may partially be explained by the hypermutation observed in the limited area of the genome where the immunoglobulin genes are located. For SIMs, the cluster has positive associations with the level of recurrence for all four subtypes as well as with those subtypes in general when in a midsize and/or long homopolymer context. This cluster has the highest median percentage of SIMs in late-replicating regions (67.5% vs. 57.8% for the other cluster combined, p<2.2e-16, Table C and Fig C in S3 Text), which may contribute to the high level of recurrence.

In cluster N, which consists of ~47% Panc-AdenoCA samples, the sources of mutagenesis are less clear, even after the inclusion of all annotation layers (S3 Text). Except for C>G and T>C SSMs, the cluster has positive associations with the recurrence of all other subtypes of SSMs and every SIM subtype. This is especially noticeable as the median of the total number of mutations across samples is intermediate. A high percentage of the recurrent mutations are SIMs in this cluster with a median of 35.0%. This is far higher than for samples of the other clusters combined (median: 15.5%, p<2.2e-16). The positive associations with all SIM subtypes when in a midsize-to-long homopolymer context may point to a slippage-related mechanism (see also cluster J).

### Negative association with recurrence: Tobacco-smoke exposure, alcohol use and increased activity of cytidine deaminases

There are also several mutagenic processes that are associated with low levels of recurrence (Fig 5) including those represented by clusters A, B, C and E. Cluster A, of which 84% are lung cancer samples, is linked to mutational processes induced by tobacco-smoke exposure (S3 Text). This cluster shows a positive association with the total number of SSMs and the percentage of C>A SSMs, the latter is a known consequence of tobacco-smoke exposure [34]. There are several factors that increase the probability of recurrence in this cluster, including the high total mutational load together with the high percentage of C>A SSMs and the enrichment of mutations in late-replicating regions (S3 Text). Also, tobacco-smoke induced mutations have been shown to be enriched in linker DNA (*i*.*e*. DNA not wrapped around a nucleosome) [10], which constitute only between 10% and 25% of the genome in eukaryotes [35]. The key to explaining the lack of recurrence seems to be that there is little tendency to favour a specific sequence context for the C>A SSMs (Fig 6). This can also be observed in the ‘tobacco smoking signature’ [11], which is present in nearly 90% of the samples in this cluster (S3 Text). Unlike for several clusters mentioned above, there is a positive association with SIMs in short homopolymer contexts, which are more frequent in the genome than longer homopolymers, and the resulting distribution is therefore also more random. Note that cluster A also has a strong association with the percentage of total 1 bp C/G deletions, which has not been described previously as a possible consequence of tobacco-smoke exposure (S3 Text and S4 Text).

Cluster B, consisting of 85% Liver-HCC samples, is likely to be linked to mutational processes indirectly induced by excessive alcohol use (S3 Text). The level of recurrence is low despite the high number of samples of the same tumour type (277) and the consistent pattern of a high percentage of T>C SSMs (median: 31.7% vs. 14.6% in the other cluster combined, p<2.2e-16). With regard to 1 bp SIMs, there is a positive association with a short homopolymer context, as for cluster A, with the exception of 1 bp A/T insertions.

In cluster C, in which ~82% are Kidney-RCC and Kidney-ChRCC samples, the mutational processes remain largely obscure except for a few samples that can be connected to aristolochic-acid exposure (S3 Text). Unlike for clusters A and B, the median number of SSMs across samples is relatively low. Furthermore, mutations are nearly equally spread between early- and late-replicating regions as only 53.9% of the SSMs and 47.5% of SIMs are in late (Table C, Figs B and C in S3 Text). SIMs are preferentially located in no or short homopolymer context, similar to clusters A and B.

In cluster E nearly one third are Breast-AdenoCA samples and key mutational characteristics point to the endogenous mutational process of increased activity of cytidine deaminases (S3 Text). There is a general paucity of associations with characteristics of recurrence. In line with this, the mutations in this cluster are nearly equally spread between early- and late-replicating regions of the genome (Table C, Figs B and C in S3 Text). The most outstanding feature of this cluster is the high percentage of C>G SSMs. This is the rarest substitution type, making the detection of recurrence unlikely, particularly if not confined to specific genomic regions. Interestingly though, the 655 C>G SSMs that are recurrent within this cluster are enriched for the motif CTCW (W = A or T) (Fig 6, see Methods). Very similar motifs have been described as being characteristic for deamination mediated by APOBEC3 [36]. The number of recurrent mutations is much lower than for the other motifs discussed. The CTCW motif is also shorter, more general and therefore relatively frequent in the genome (5.4% of all NNCN 4-mers), all possible causes for the lacking trend towards recurrence.

### The added value of the recurrence-related features

The PCA shows that seven of the sixteen features that contribute above average to the first two PCs are related to recurrence (Fig 3). In addition, all 16 clusters have a statistically significant association with two or more recurrence-related features (Fig 5). The importance of the recurrence-related features is further demonstrated by the results of running the entire workflow (Fig 3) using only the general features. In this case we are no longer able to separate all ultra-hypermutator samples from the rest of the cohort (S2 Fig). Furthermore, the cluster linked to hypermutation of the immunoglobulin genes (cluster M) is dissolved, and the cluster possibly linked to gastric-acid exposure (cluster L) is less cancer-specific as it absorbs 90 samples of the dissolved cluster M and thereby nearly doubles in size. Another key difference is that only ~55% of the Lymph-CLL samples without hypermutation of the immunoglobulin genes are confined to a single cluster as opposed to ~86% when using all features.

## Discussion

Only a very small percentage of the 1,057,935 recurrent SSMs and 186,576 recurrent SIMs in the PCAWG cohort are expected to be purely by chance. We estimate based on simulations that only around 0.47% of the SSMs would be recurrent if no biological factors would play a role, which is less than one fifth of the observed 2.44%. Technical artefacts could contribute to the level of recurrence, but although they can never be fully excluded, the PCAWG consortium has made a great effort to minimise false positive calls. A consensus was taken of the individual results from multiple somatic mutation callers, followed by the application of various filters to remove, *e*.*g*., germline variants [12] (see Methods). This resulted in a conservative, but reliable dataset of somatic mutations. Increasing the size of the cohort may change the percentage of recurrent mutations, but in which direction depends on the tumour type of the additional samples, their mutational burden and importantly the mutational processes underlying the observed mutations.

Recurrence is considered an important indication that a mutation might be under selective pressure in protein-coding regions [37, 38]. Hence, by focusing on recurrence we are inherently not only looking at the mutational consequences of mutational and repair processes, but also at positively selected mutations. One way that has been used to reduce the influence of the latter is to count all recurrent mutations only once [39]. However, in our approach, as we describe each individual cancer genome with the 42 features, this is not an option as we would not know to which samples to add this single count for each recurrent mutation. Instead, we would need to leave out all recurrent mutations, but this would even be more rigorous. In either case, it also implies that over a million mutations are assumed to be under positive selection. Besides the fact that recurrence is not a sufficient condition for positive selection [37], it may not even be a necessary one in a dataset of the size of our cohort [3, 38]. Another option is to remove all predicted driver mutations. In total there are only 4,223 predicted driver mutations that are either SSMs or SIMs, which constitutes just 0.009% of the total amount of mutations. It is, therefore, unlikely that leaving them out will affect the general features. Their effect on the percentage of overall recurrence is also negligible (-0.001% for SSMs and +0.002% for SIMs), partly because only ~12% of the predicted driver mutations are recurrent within the PCAWG cohort. Based on the overall statistics, removing the predicted driver mutations will also hardly affect the recurrence-related features of individual cancer genomes and consequently not result in any noticeable change in the uncovered clusters. As identifying the driver mutations is, in addition, far from unambiguous and a dynamic area of research [3, 18], it is of limited practicality to our workflow to remove them. Of note, the impact of positive selection might be greater when analyzing only the exome [39] as there are less mutations in total and the large majority of drivers is found in protein-coding loci [3, 18].

**Fig 7:**
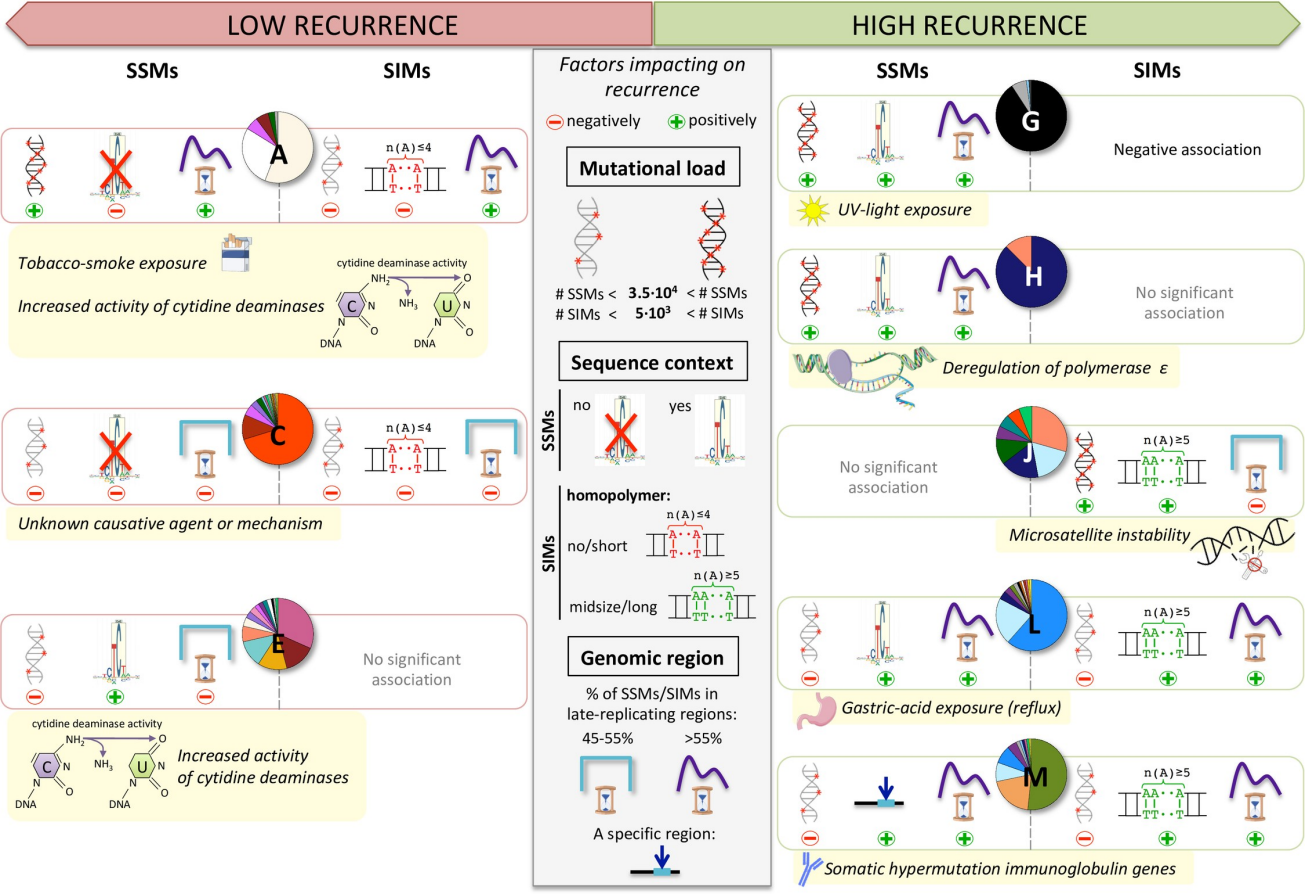
Factors impacting on recurrence in the context of the clusters. None of the three key factors (middle panel) that impact on recurrence individually explain the observed level of recurrence in the clusters. Whether a cluster has a relatively high or a comparatively lower mutational load is based on the median number of SSMs/SIMs across its samples (Fig 4). The actual specific sequence contexts for SSMs are shown in Fig 6. For cluster M there is enrichment for a specific sequence context as well, which is AGCT for C>G SSMs that are recurrent within this cluster (n = 949) (S3 Fig). For SIMs a homopolymer of A/T’s is used to represent any type of homopolymer. Clusters A and C have a positive association to no and/or short homopolymer context for all types of 1 bp SIMs (red), while for clusters J, L and M this is the case for midsize and/or long homopolymer context (green) (Fig 5). For the replication time region we compute the percentage of SSMs/SIMs that are in late-replicating regions (S3 Text). If this percentage is between 45–55%, then we consider the mutations to be nearly equally spread between early- and late-replicating regions of the genome. The specific region that is enriched in cluster M refers to the immunoglobulin genes. The recurrence in clusters A and G is also likely to be positively impacted by an increased mutation rate in a specific region as the majority of their samples are from a particular tumour type for which this has been reported. For lung cancer (cluster A) the mutation rate is increased in linker DNA [10] and for Skin-Melanoma (cluster G) at active transcription factor binding sites [24].

Mutational load, enrichment of mutations in a specific sequence context or in specific parts of the genome all impact on recurrence. However, none of these factors provide individually a universal explanation for the observed levels of recurrence per cluster (Fig 7). For example, the cluster linked to tobacco-smoke exposure has a very low percentage of recurrence, despite the high mutational load, the enrichment of mutations in late-replicating regions and increased mutation rate in linker DNA. The absence of a preferred sequence context likely plays a role in this. The short and non-specific motif found in samples with increased activity of cytidine deaminases (CTCW) is also not sufficient by itself to result in high levels of recurrence. For causative agents like UV light and deregulated activity of POLE, however, the high total number of mutations combined with the observed 6 bp specific sequence context does lead to high levels of recurrent SSMs. For the cluster linked to gastric-acid exposure, the number of SSMs is much lower than for the clusters linked to those two agents or tobacco-smoke exposure. Nevertheless, it has a high level of recurrence, likely because of the 5 bp sequence motif for T>G SSMs and the three times higher occurrence of SSMs in late-replicating regions than in early. One possible caveat here is that replication timing is a process with very high plasticity across cell types [19], and taking the median timing across the available five cancer cell lines (S3 Text) may individually lead to non-adequate interpretations. A typical example for the potential impact of an elevated local mutation rate on the proportion of recurrence is the hypermutation of the immunoglobulin genes in memory B cells. As mutations detected in several lymphoma samples are largely confined to those genes, their modest total number of mutations still results in a high relative level of recurrence. Finally, in the case of the MSI samples, the slippage of the DNA polymerase during replication of repetitive sequences, combined with a lack of repair capacity results in a high percentage of SIMs in a midsize-to-long homopolymer context. This coincides with a high level of recurrence for SIMs, despite the relatively equal distribution of SIMs between early- and late-replicating regions that we observe and that has been reported before [28]. Associations with the much more frequent short homopolymers do not translate into high level of SIM recurrence, not even in the case of a high number of total SIMs (*e*.*g*. as observed in the ‘tobacco-smoke exposure’ cluster). The effect of the sequence context may be stronger for SIMs than for SSMs. This would explain the ~3.6 fold higher percentage of recurrent SIMs (8.69%) versus SSMs (2.44%), despite the fact that there are 20 times more SSMs. Unlike for SSMs, the actual position of an insertion/deletion in a homopolymer cannot be determined, contributing to loss in resolution and a higher likelihood of recurrence. In summary, we infer that the non-randomness in the distribution of mutations strongly depends on the causative agent. Consequently, recurrence is generally able to cluster the genomes in a way that shows clear associations with tumour type assignments and mutational processes. For SSMs 60.0% is only recurrent in one particular tumour type, while for SIMs this percentage is 10.7% (S2 Table). This suggests a higher resemblance of mutational patterns within tumour types for SSMs than for SIMs. In contrast, 79.8% of the recurrent SIMs (versus 37.1% for SSMs) can only be detected in a pan-cancer approach, pointing to shared mutational processes which allow us to group samples in a more tumour type independent way. The recurrence-related features based on these recurrent SSMs and SIMs are key to our ability to cluster the cancer genomes into biologically relevant clusters. If we only use the general features we lose important information about mutational processes (S2 Fig).

The simple general mutational features, the different types of annotation and the uncovered sequence motifs do provide a deeper understanding of several mutational processes (S3 Text). For instance, MSI samples (cluster J) have a particularly high percentage of 1 bp C/G deletions in the context of midsize homopolymers. We also see a strong shift towards the presence of SIMs compared to SSMs resulting in a high absolute and relative number of SIMs. Ultra-hypermutators (cluster H) form a mirror image in this respect as we observe a shift in the opposite direction, resulting instead in a high absolute and relative number of SSMs. Another difference is that in cluster H there is a significantly higher percentage of mutations in late-replicating regions than for cluster J (SSMs: 60.2% vs. 52.8%, p = 0.0011, SIMs: 66.7% vs. 51.3%, p = 1.8e-06). The mutational processes induced by tobacco-smoke exposure (cluster A), whose link to an increased percentage of C>A SSMs is well-known, are also associated with a high percentage of 1 bp C/G deletions (S4 Text). A third example is the high percentage of 1 bp A/T insertions in context of a short homopolymer observed for cluster C that mainly consists of Kidney-RCC and Kidney-ChRCC samples. For this cluster there is also a nearly equal distribution of mutations between early and late-replicating regions, which is in contrast to what is generally observed for cancer genomes [8] with the exception of MSI samples [28]. However, unlike for MSI genomes, for cluster C a deficient MMR pathway can most likely not explain it. Deficient translesion synthesis has been shown in yeast to also lead to a more equal distribution [40]. In the opposite direction, the cluster possibly linked to gastric-acid exposure (cluster L) has an unexpectedly strong tendency of both SSMs and SIMs to be in late-replicating regions compared to all other clusters, which could point to the extensive usage of error-prone polymerases. The sequence motif (AACTT) found for the T>G SSMs recurrent within this cluster (n = 38,399, 38.9% with the motif) provides another interesting characteristic (Fig 6). Only 8.9% of the T>G SSMs recurrent in the 2,479 samples not in cluster L (n = 25,318) are confined to this motif. An important contributor to the recurrent T>G SSMs not in cluster L is the cluster linked to the deregulated activity of POLE (cluster H). The T>G SSMs that are recurrent within cluster H (n = 11,553) are instead enriched for the sequence motif AAATTTAT (S4 Fig). There are some interesting parallels between cluster H and L. First, for both holds that the Eso-AdenoCA and ColoRect-AdenoCA samples that form the majority of cluster L and H, respectively, have a higher median number of SSMs than samples from the same tumour types not assigned to the respective clusters (Eso-AdenoCA: 29,302.5 vs. 11,404, p = 1.3e-09, ColoRect-AdenoCA: 850,298 vs. 15,045, p = 1.5e-08). Second, changes to the dNTP pool are in both cases likely linked to the observed mutations together with the more frequent usage of alternative (error-prone) polymerases (cluster L) or a polymerase with a deregulated activity (cluster H). Third, the sequence motifs found for both clusters exceed the single neighbouring base. The latter is the case for all sequence motifs that we found (Fig 6) and also none of them have the same number of bases on both sides of the mutated position. These two observations and the motifs themselves are also important to take into account when estimating the background mutation rate used in *e*.*g*. driver prediction [25, 37]. The motifs point to an increased mutational probability of individual bases [22] that is context-specific and characteristic for certain mutational processes. This has primarily been shown and taken into account for a sequence context of a single neighbouring base [37] or, less frequently, for an equal number of several bases at both sides of the mutation [25]. As we extract these motifs based on recurrent mutations there is a possibility that positive selection plays a role. However, this is likely negligible as the number of recurrent, predicted driver mutations is only 427 when considering all six SSM subtypes together.

Several of our clusters are linked to cancer phenotypes that are relevant for treatment and/or have prognostic value. Our division into 16 clusters and their characteristics could, therefore, be valuable for complementing current classification schemes, which are mainly based on histology and organ of origin. We can assign a new sample to one of our 16 clusters by first projecting it onto the PCA space based on the PCAWG cohort. Next, we use the first 18 principal components to compute the Euclidean distance to the centroid of each of the 16 clusters and assign the sample to the nearest one. If there are multiple clusters with a minimum difference in distance to the new sample, then to select one cluster we use the sequence motifs (Fig 6) and various layers of annotation (S3 Text) like replication time. Ideally, we would use only the samples in the ‘reference set’, which currently is the PCAWG cohort, to compute the recurrence-related features for a new sample. However, ~90% and ~72% of the recurrent SSMs and SIMs, respectively, in this set are only recurrent in two samples (Fig F in S2 Text). Therefore, the recurrence-based features of the new sample might be underestimated in which case the sample is also less likely to be assigned to clusters that have a positive association with recurrence. Instead we would need to include the new sample for computing recurrence, which could also affect the recurrence-related features for some samples in the reference set. This might result in changes in the clustering, but the impact of a single sample is most likely minimal. Of note, the interdependence of samples in terms of the recurrence-related features also makes cross-validation difficult. The level of recurrence is not high enough to compute recurrence for the training and test set separately, and even a leave-one-out strategy would create dependence between the two sets. We hypothesize that, by increasing the size of the reference set, we will reach at a certain point a plateau in terms of recurrence. This would enable us to compute the recurrence-based features for a new sample using only the reference set. A larger dataset would also allow further insights into the non-randomness of mutational processes, especially of those that are not active across a large set of samples or that are only observed in specific tumour types for which the number of samples is currently limited. Efforts are, in fact, already on their way to expand the PCAWG dataset with more whole-genome sequences from ICGC and other consortia.

Given that incorporating whole-genome sequencing in a clinical setting is gaining traction as evidenced by projects like Genomics England (www.genomicsengland.co.uk) and the Hartwig Medical Foundation (www.hartwigmedicalfoundation.nl), analyses making full use of this kind of data are urgently needed. Ultimately, whole-genome sequencing can then replace multiple diagnostic tests currently in use and make diagnoses more accurate. One example illustrating the value of our clusters towards this goal is the MSI phenotype linked to cluster J. For these patients, immunotherapy may be beneficial [41] while adjuvant chemotherapy may not be needed [42]. To classify a cancer genome as MSI, we can use our 42 features to determine whether or not a sample belongs to cluster J, as detailed above. A high percentage of 1 bp C/G deletions in a midsize homopolymer is, however, even by itself already a strong indication for MSI. The MSI phenotype cluster J captures, forms a possible alternative to either explicitly identifying all microsatellite alterations between tumour and normal tissue [43] or using specific markers to detect alterations in five or seven of them like the Bethesda markers [44]. There are also 10 mutational signatures linked to a deficient MMR pathway of which seven are based on single base substitutions, two on doublet base substitutions and one on small indels [20]. Two more indel-based signatures (ID1 and ID2) that are found in nearly all cancer genomes, are linked to a deficient MMR pathway if they contribute >10,000 indels. Signatures look at mutational processes at mutation level rather than sample level. A non-zero contribution of an individual MSI-linked signature or a high contribution (>10,000) of ID1 and ID2 is not sufficient to classify a sample as MSI given that this naïve approach would results in 368 possible candidates. Instead it requires a combination of signatures and/or thresholds on the amount of mutations contributed to the sample to be able to use the signatures for MSI classification. A second example of an actionable phenotype that we capture with one of our clusters is ultra-hypermutation (cluster H), which has also been related to beneficial results from immunotherapy [45, 46]. A third example is the somatic hypermutation of the immunoglobulin genes, which identifies memory B-cells as the cell of origin in the case of lymphomas. This has been linked to a less aggressive form of Lymph-CLL and more favourable prognosis [33], which may in turn influence treatment selection. Without explicitly analysing the immunoglobulin genes [47], we were largely able to separate the Lymph-CLL samples with somatic hypermutation (cluster M) from those without (cluster D). The characteristics of the former group include a high percentage of recurrent C>G SSMs and 1 bp A/T deletions. A final example relates to those Eso-AdenoCA samples that are assigned to cluster L, which have a high percentage of T>C as well as T>G SSMs and a higher total mutational load than Eso-AdenoCA samples not assigned to this cluster. Eso-AdenoCA samples with the characteristics of cluster L have also been suggested to benefit from immunotherapy [48]. The same treatment option may therefore be prioritized for the 22 Stomach-AdenoCA samples that are also in cluster L. Similarly, a refined investigation of tumour samples that do not cluster with the vast majority of its own kind may ideally point to differences in disease prognosis or treatment response and even has the potential to define novel subtypes or reveal misclassification. Such an analysis would be especially worthwhile for the ~20% or less samples from Kidney-RCC, Liver-HCC, Lung-SCC or Lymph-BNHL that are not assigned to the main cluster. Another possible application of our classification scheme is to assign a metastatic sample with unknown primary site to a cluster to shed light on the possible tissue of origin or pan-cancer characteristics like MSI.

In conclusion, we provide here a comprehensive analysis of somatic mutations in cancer genomes irrespective of tumour type using 42 features with a truly pan-cancer focus. This allows us to include tumour types with very few samples for which individual analysis is little informative. Moreover, information can be borrowed across the entire data set enabling the detection of processes present in multiple tumour types. We let the genome prioritize what is important by using position-specific recurrence and by considering features that do not depend on the completeness and correctness of current genome annotations. This has enabled us to delineate various mutational processes, uncover new mutational manifestations and characterize several actionable clinical phenotypes in a novel way. Findings from this and similar analyses in the future will be of utmost importance for the goal to tailor treatment to the individual patient.

## Methods

### PCAWG cohort – quality control

We used the cohort of cancer genomes assembled by the PCAWG project [12] of the ICGC and TCGA. For every donor, whole-genome sequencing data was available for a normal-tumour pair and all samples were analysed uniformly. A detailed description of the quality control is provided in the PCAWG marker paper [12]. In short, 176 samples were excluded for various reasons as part of the quality control, most commonly because of contamination with RNA. Samples of another 75 donors were of borderline quality for various reasons, including a high percentage of paired reads mapping to different chromosomes [12, 49]. We decided not to include the samples of those donors, which left us with genomic data of 2,583 donors covering 37 tumour types (S1 Table). The distribution of the samples across the tumour types is also indicated in S1 Table. In case there were multiple tumour samples for the same donor, we selected a single sample following the decision made within the consortium. To make the decision five criteria were used as described by the PCAWG Drivers and Functional Interpretation Group [18]. In order of importance, they prioritized the sample: 1) of a primary tumour over metastatic and recurrent ones; 2) with a OxoG score over 40, which indicates low levels of oxidative damage artefacts [50]; 3) with the highest quality according to the star rating system [49]; 4) with RNA-Seq data available; 5) with the lowest level of contamination with foreign DNA. If none of these criteria led to the selection of a single sample, a random selection was made.

### PCAWG cohort – mutation calls

The description of the procedure for the mutation calls is provided in the marker paper of the PCAWG consortium [12]. In brief, the sequenced reads of the respective normal and tumour sample pairs were aligned with BWA-MEM to the GRCh37/h19 genome. Four mutation calling pipelines were run on the resulting BAM-files for each normal/tumour sample pair. The pipelines used for calling SSMs were MuSE [51] and three in-house pipelines developed at the Deutsches Krebsforschungszentrum (DKFZ) in collaboration with the European Molecular Biology Laboratory (EMBL), Wellcome Sanger Institute and Broad Institute, respectively. A consensus set was built by keeping those calls on which two or more callers agreed. SIMs were called by SMuFIN [52] and three pipelines developed by the same institutes as mentioned for SSMs. The consensus was determined by stacked logistic regression instead, as the level of agreement between the callers was lower than for SSMs. Furthermore, the SIM calls were left aligned to make them comparable across samples. Several filters were applied to both the SSM and SIM calls to remove, among other things, calls due to oxidative damage artefacts [50] and germline variants. Great care was taken by the consortium to reduce the number of false positive mutation calls, resulting in a reliable dataset that is believed to be a conservative representation of the true set of mutations.

### Definition of mutations

For SSMs there are 16 possible subtypes. However, we can neither detect substitutions with a base of the same type (*e*.*g*. A>A) nor do we usually know on which strand the (pre-)mutagenic event happened first (*e*.*g*. A>C is equivalent to T>G on the other strand). Therefore, we combined the substitutions that are each other’s reverse complement and refer to them by the pyrimidine of the mutated base pair: C>A, C>G, C>T, T>A, T>C and T>G. We regarded substitutions directly next to each other (median number across samples: 25) as separate single base events since, aside from the very limited numbers, in several cases the individual callers only supported one single base event, and only the consensus resulted in a multiple base substitution call. For 1 bp SIMs, these are the four subtypes A/T deletions, C/G deletions, A/T insertions and C/G insertions, as analogously to SSMs, we cannot determine on which strand the (pre-) mutagenic event happened first.

### Features describing each cancer genome

We computed 29 general features and 13 related to recurrence (Table A in S1 File) to characterize different aspects of the somatic mutations in a cancer genome. We used the vcfR package in R to read in the VCF files [53]. The general features comprised the number of SSMs and SIMs (two features), the percentage of SIMs with respect to the total number of mutations (one feature), the distribution of SSMs and SIMs across the different subtypes (six and four features, respectively), and the homopolymer context of 1 bp SIMs for each of the four subtypes (four times four features). We used the BCFtools (version 1.5) to compute recurrence using the VCF files as input. Recurrence was captured by the overall percentage of recurrent SSMs and SIMs (two features), percentage of recurrent mutations of type SIM (one feature) and recurrence per SSM and SIM subtype (six and four features, respectively). The homopolymer context is not included in the recurrence features, as the number of recurrent SIMs is too low to stratify into 16 additional features. Except for the number of SSMs and SIMs, all other 40 features were in percentages.

### Principal Component Analysis and hierarchical clustering on Principal Components

The R package FactoMineR (v1.41) was used for the PCA [14]. All input features for the PCA were scaled to zero mean and unit variance to account for the differences between the ranges of the features, especially with respect to the two features in absolute terms versus the ones in terms of percentages. The first 18 PCs explained together over 80% of the variance of the data. The remaining components were assumed to mostly represent noise in the data. The PCs were used as input to the ‘hierarchical clustering on principal components’ (HCPC) function from the FactoMineR package. The Euclidean distance was used as a measure of dissimilarity and the Ward criterion for linkage. We cut the hierarchical clustering tree at various heights to see a more global down to a more specific division of the samples. The HCPC function includes a consolidation step in the form of k-means clustering [15], which uses the centroids of the hierarchical clustering as a starting point. This consolidation step was repeated a maximum of 10 times. The k-means clustering increased the variance between clusters from 17.5 to 18.9. Other advantages of this hybrid approach are that it reduces the sensitivity of k-means clustering to outliers and the initial centroids are selected in an informed way instead of at random. As a consequence of this step, some samples were finally assigned to a different cluster than after the hierarchical clustering. A ‘v test’, included in the FactoMineR package, was used to determine which features were significantly associated with each cluster. This test compares the mean of a particular feature in a cluster to the overall mean in the dataset. We corrected the p-values of all ‘v tests’ for multiple testing using the Benjamini-Yekutieli method. A feature is considered to be significantly associated to a cluster if the adjusted p-value < 0.05.

### Detection and enrichment of motifs

We collected for clusters A, E, G, H, L and M all SSMs of the subtype that is the most characteristic. This is C>A for clusters A and H, C>G for cluster E and M, C>T for cluster G and T>G for cluster L. In addition, we looked at T>G SSMs in cluster H to compare them to cluster L. Next, we extracted from the reference genome (GRCh37/h19) the ten adjacent bases in 5’ and 3’ direction of the mutation using the Rsamtools package in R. We used the extracted sequence context as input to construct two sequence logos per cluster: one for the mutations that are recurrent within the cluster and one for those that are not. We include each recurrent mutation only once to avoid giving extra weight to highly recurrent mutations. As a measure of information content we used the relative entropy [54, 55], which is defined for position *i* by:

$${RE}_{i}=\sum_{b\in \{A,C,G,T\}}f(b_{i}){log}_{2}\frac{f(b_{i})}{P(b)}$$

Here, *f*(*b*_*i*_) stands for the frequency of base *b* (A, C, G or T) in position *i* and *P*(*b*) stands for the prior probability of base *b* as determined by the frequency in the human genome (GRCh37/h19). The height of each base in the sequence plot is proportional to $f(b_{i}){log}_{2}\frac{f(b_{i})}{P(b)}$. A positive value corresponds to an enrichment of the base with respect to the prior probability and a negative value to a depletion. The relative entropy (RE_i_) is zero, if all four bases are observed with the same frequency as the prior in position *i*. We set 0.25 as a threshold for RE_i_ to define the enriched motif. Furthermore, we computed per cluster the percentages of all, non-recurrent and recurrent SSMs that were in the sequence context that was found to be enriched in the recurrent SSMs. To estimate the percentage of the respective motifs in the human genome, we first slid a window of the same size (k) as the motif across the genome with a shift equal to the length of the motif and counted all possible k-mers. Next, we added to this the counts retrieved in the same way for the reverse complement of the reference sequence (corresponding to the opposing strand), since we also combined the reverse complements for each of the SSM subtypes. From this we computed the percentage of the enriched motif with respect to all k-mers and to the k-mer with the base that is mutated in the enriched motif at the same position.

### Statistical tests

The correlation between every possible pair of the 42 features was measured by the Spearman’s rank correlation coefficient using the R package Hmisc (v4.1–1). Multiple testing correction of the p-values of all correlation tests (including those in S2 Text) was done by the Benjamini-Yekutieli method. For the other correlations mentioned we also used the Spearman’s rank correlation coefficient.

We used the Wilcoxon rank-sum test with continuity correction as the test of significance for differences in features observed between clusters.

The different proportions of sequence motifs between recurrent and non-recurrent SSMs were assessed by using χ^2^ tests.

### Plots

Figs 1, 3, 5 and 6, the pie charts in Fig 4 and the plots in Supporting Information, except for S1, were made using the R package ggplot2 (v3.0.0). Fig 6, S3 Fig and S4 Fig additionally required ggseqlogo (v0.1) [56] and Fig 2 was made with the use of the R package corrplot (v0.84). Fig 7 was made using Microsoft PowerPoint and we also included images from the Servier Medical Art website (http://smart.servier.com/). The ‘clustering tree’ in S1 Fig was made using the clustree R package [57]. We have manually replaced the nodes in the tree with the pie diagram showing the distribution of tumour types in each cluster. For the colours of the different tumour types we have made use of the script provided by the PCAWG consortium, available at: https://github.com/ICGC-TCGA-PanCancer/pcawg-colour-palette.

## Supporting information

S1 Fig Clustering tree showing tumour type distribution for 2 to 20 clusters. The clustering tree shows how clusters evolve across different clustering resolutions ranging from 2 to 20 clusters. For example, cluster G splits off from the rest of the cohort at a resolution of three clusters and remains largely unchanged in higher resolutions. We have marked for each of our 16 clusters the clustering resolutions across which they remain largely stable, *i*.*e*. the Jaccard similarity index between a cluster at resolution 16 and one at a higher or lower resolution is at least 0.85. The number under each cluster indicates the number of samples in that particular cluster. The colour of an arrow indicates the number of samples the two connected clusters have in common. The transparency of the arrow indicates the proportion of samples the two connected clusters have in common with respect to the cluster at the higher resolution. Only arrows representing a proportion of more than 0.1 are shown. Consequently, the number of samples in a cluster at a certain clustering resolution may not match with the connected cluster(s) at a higher resolution. Note that the clustering shown is the result after the k-means clustering step.
(PDF)

S2 Fig PCA and clustering with and without the recurrence-related features. When using only the 29 general features for the PCA (A), the first two PCs explain less variance than when using all 42 features for the PCA (B) (27.5% vs. 29.1%). The features indicated in the two PCA plots are those that contribute above average to the first two PCs. The subsequent clustering also differs as shown in (C) and (D). Without using the recurrence-related features, only five of the eight samples linked to ultra-hypermutation (D – cluster H) are in a separate cluster (C – cluster VIII). Also the cluster linked to hypermutation of the immunoglobulin genes (D–cluster M) is dissolved as evidenced by the fact that the samples are spread across eight clusters (C – clusters III, IV, VI, XI, XII, XIII, XIV and XV). One consequence of this is that only 19 of the 40 the Lymph-CLL samples with hypermutation are in the same cluster as opposed to 36 when using all features (E). In addition, the largest fraction of cluster M ends up in a cluster with Eso-AdenoCA and Stomach-AdenoCA samples (C – cluster XII), making that cluster less cancer-specific than when using all features (D – cluster L). The Lymph-CLL samples without hypermutation of the immunoglobulin genes are also no longer largely confined to a single cluster (E). Moreover, the samples with and without hypermutation end up more often in the same cluster than when recurrence-related features are also used.
(PDF)

S3 Fig Enriched sequence motifs for C>G SSMs in cluster M. The sequence logos represent the sequence context of ten bp 5’ and 3’ of the non-recurrent (left-side) or recurrent (right-side) C>G mutations of cluster M. Here recurrence is defined as a mutation at the same genomic location in two or more samples from cluster M. Relative entropy is used as a measure of information content (see Methods). Setting a threshold of 0.25 for the relative entropy results in the motifs highlighted in the rectangles. In the upper right corner of both sequence logos the number of mutations is indicated. To the right of the sequence logos are the percentages in which the enriched motif found for the recurrent C>G SSMs is present in context of the mutations in the cluster and the corresponding k-mers in the genome (N = A, C, G or T). The enrichment for the motif for recurrent C>G SSMs is significantly higher than for the non-recurrent C>G SSMs (χ^2^ test: p<2.2e-16).
(TIF)

S4 Fig Enriched sequence motifs for T>G SSMs in cluster H. The sequence logos represent the sequence context of ten bp 5’ and 3’ of the non-recurrent (left-side) or recurrent (right-side) T>G mutations of cluster H. Here recurrence is defined as a mutation at the same genomic location in two or more samples from cluster H. Relative entropy is used as a measure of information content (see Methods). Setting a threshold of 0.25 for the relative entropy results in the motifs highlighted in the rectangles. In the upper right corner of both sequence logos the number of mutations is indicated. To the right of the sequence logos are the percentages in which the enriched motif found for the recurrent T>G SSMs is present in context of the mutations in the cluster and the corresponding k-mers in the genome (N = A, C, G or T). The enrichment for the motif for recurrent T>G SSMs is significantly higher than for the non-recurrent T>G SSMs (χ^2^ test: p<2.2e-16).
(TIF)

S1 Table Tumour type abbreviation, full name and number of samples. (PDF)

S2 Table Recurrence in pan-cancer context and within tumour type(s). (PDF)

S1 Text Estimation of the levels of recurrence when purely driven by chance. (PDF)

S2 Text Recurrence versus general mutational characteristics. (PDF)

S3 Text Detailed cluster-specific descriptions. (PDF)

S4 Text Smoking history and related mutational subtypes. (PDF)

S1 File Characteristic plots summarising each of the 42 features. (PDF)

S2 File Sample distribution per tumour type across the 16 clusters. (PDF)
