# Supplementary figures and images for: Recurrent somatic mutations reveal new insights into consequences of mutagenic processes in cancer
Source: PLoS Comput Biol. 2019 Nov 25;15(11):e1007496. doi: 10.1371/journal.pcbi.1007496 (PMC6901237; doi:10.1371/journal.pcbi.1007496)

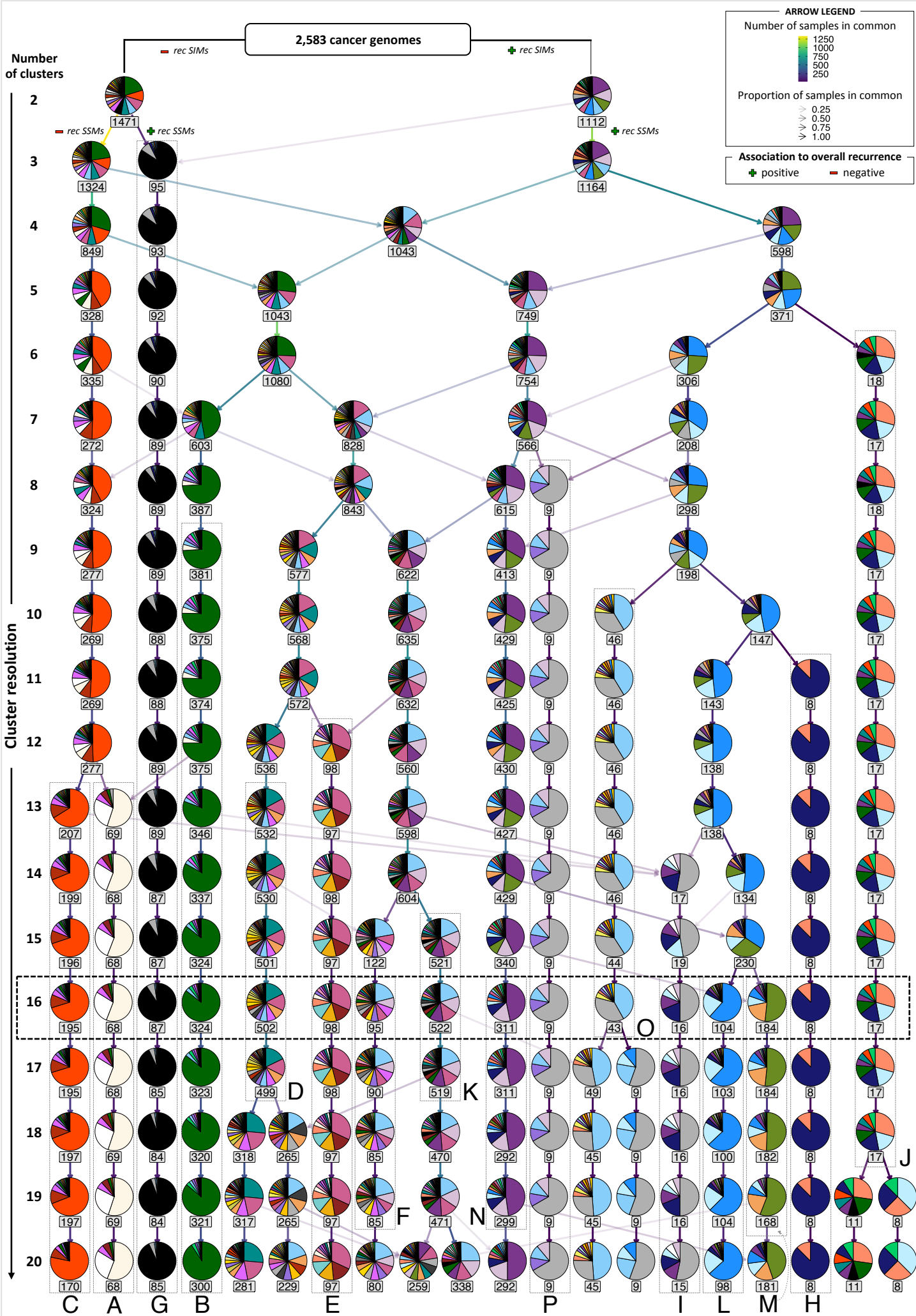

Supplement: S1 Fig — The clustering tree shows how clusters evolve across different clustering resolutions ranging from 2 to 20 clusters. For example, cluster G splits off from the rest of the cohort at a resolution of three clusters and remains largely unchanged in higher resolutions. We have marked for each of our 16 clusters the clustering resolutions across which they remain largely stable, i.e. the Jaccard similarity index between a cluster at resolution 16 and one at a higher or lower resolution is at least 0.85. The number under each cluster indicates the number of samples in that particular cluster. The colour of an arrow indicates the number of samples the two connected clusters have in common. The transparency of the arrow indicates the proportion of samples the two connected clusters have in common with respect to the cluster at the higher resolution. Only arrows representing a proportion of more than 0.1 are shown. Consequently, the number of samples in a cluster at a certain clustering resolution may not match with the connected cluster(s) at a higher resolution. Note that the clustering shown is the result after the k-means clustering step. (PDF) [file pcbi.1007496.s001.pdf]

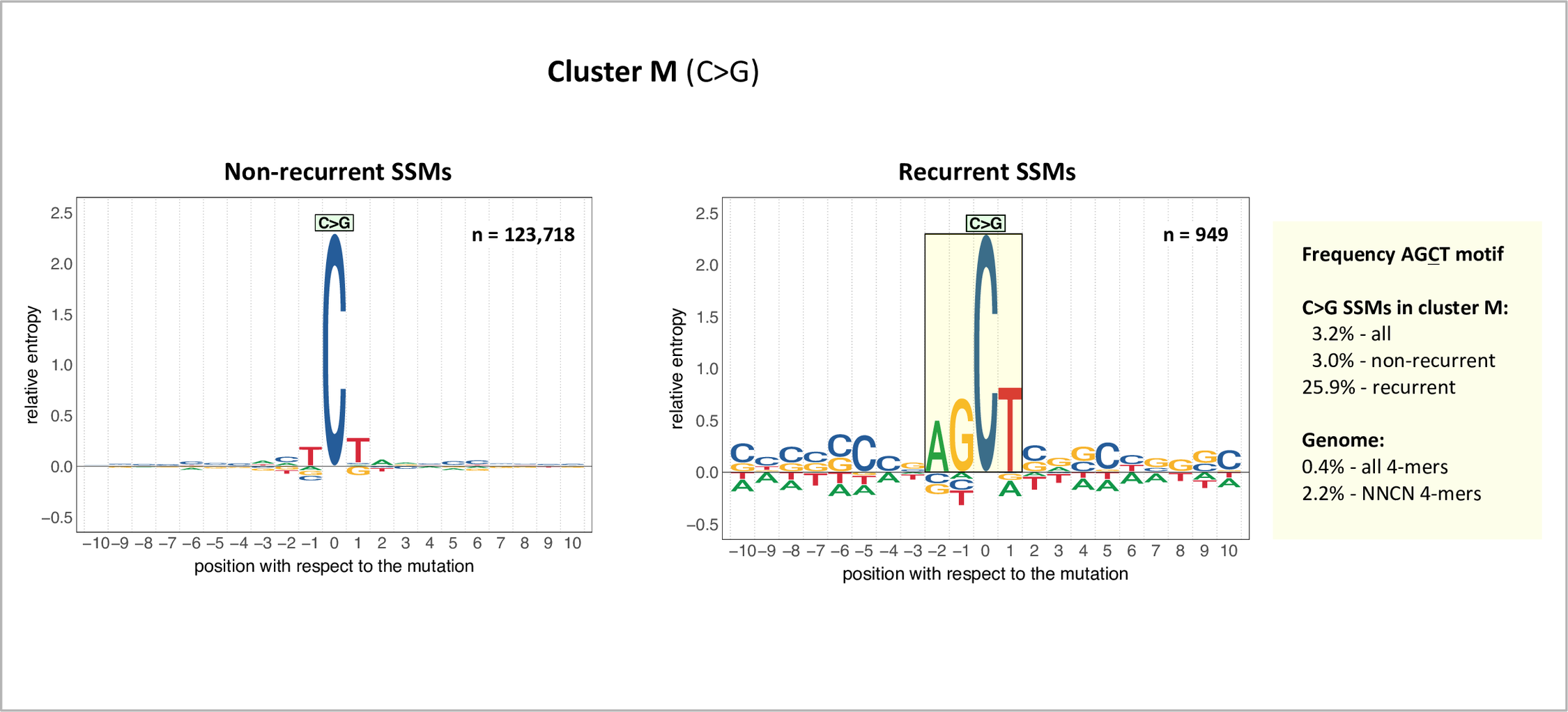

Supplement: S3 Fig — The sequence logos represent the sequence context of ten bp 5’ and 3’ of the non-recurrent (left-side) or recurrent (right-side) C>G mutations of cluster M. Here recurrence is defined as a mutation at the same genomic location in two or more samples from cluster M. Relative entropy is used as a measure of information content (see Methods). Setting a threshold of 0.25 for the relative entropy results in the motifs highlighted in the rectangles. In the upper right corner of both sequence logos the number of mutations is indicated. To the right of the sequence logos are the percentages in which the enriched motif found for the recurrent C>G SSMs is present in context of the mutations in the cluster and the corresponding k-mers in the genome (N = A, C, G or T). The enrichment for the motif for recurrent C>G SSMs is significantly higher than for the non-recurrent C>G SSMs (χ2 test: p<2.2e-16). (TIF) [file pcbi.1007496.s003.tif]

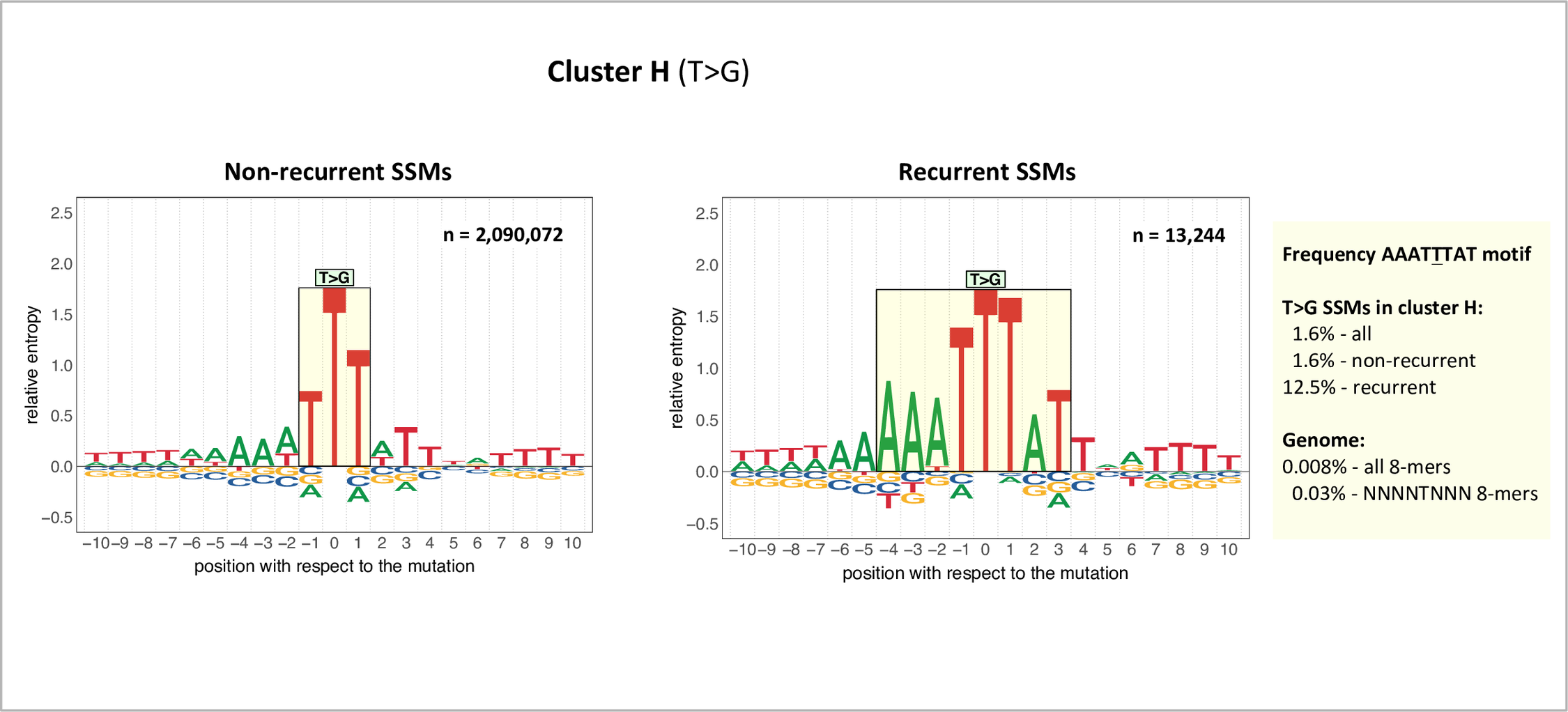

Supplement: S4 Fig — The sequence logos represent the sequence context of ten bp 5’ and 3’ of the non-recurrent (left-side) or recurrent (right-side) T>G mutations of cluster H. Here recurrence is defined as a mutation at the same genomic location in two or more samples from cluster H. Relative entropy is used as a measure of information content (see Methods). Setting a threshold of 0.25 for the relative entropy results in the motifs highlighted in the rectangles. In the upper right corner of both sequence logos the number of mutations is indicated. To the right of the sequence logos are the percentages in which the enriched motif found for the recurrent T>G SSMs is present in context of the mutations in the cluster and the corresponding k-mers in the genome (N = A, C, G or T). The enrichment for the motif for recurrent T>G SSMs is significantly higher than for the non-recurrent T>G SSMs (χ2 test: p<2.2e-16). (TIF) [file pcbi.1007496.s004.tif]
