# Supplementary material for: Recurrent somatic mutations reveal new insights into consequences of mutagenic processes in cancer
Source: PLoS Comput Biol. 2019 Nov 25;15(11):e1007496. doi: 10.1371/journal.pcbi.1007496 (PMC6901237; doi:10.1371/journal.pcbi.1007496)
Supplement: S2 Fig — When using only the 29 general features for the PCA (A), the first two PCs explain less variance than when using all 42 features for the PCA (B) (27.5% vs. 29.1%). The features indicated in the two PCA plots are those that contribute above average to the first two PCs. The subsequent clustering also differs as shown in (C) and (D). Without using the recurrence-related features, only five of the eight samples linked to ultra-hypermutation (D – cluster H) are in a separate cluster (C – cluster VIII). Also the cluster linked to hypermutation of the immunoglobulin genes (D–cluster M) is dissolved as evidenced by the fact that the samples are spread across eight clusters (C – clusters III, IV, VI, XI, XII, XIII, XIV and XV). One consequence of this is that only 19 of the 40 the Lymph-CLL samples with hypermutation are in the same cluster as opposed to 36 when using all features (E). In addition, the largest fraction of cluster M ends up in a cluster with Eso-AdenoCA and Stomach-AdenoCA samples (C – cluster XII), making that cluster less cancer-specific than when using all features (D – cluster L). The Lymph-CLL samples without hypermutation of the immunoglobulin genes are also no longer largely confined to a single cluster (E). Moreover, the samples with and without hypermutation end up more often in the same cluster than when recurrence-related features are also used. (PDF) [file pcbi.1007496.s002.pdf]

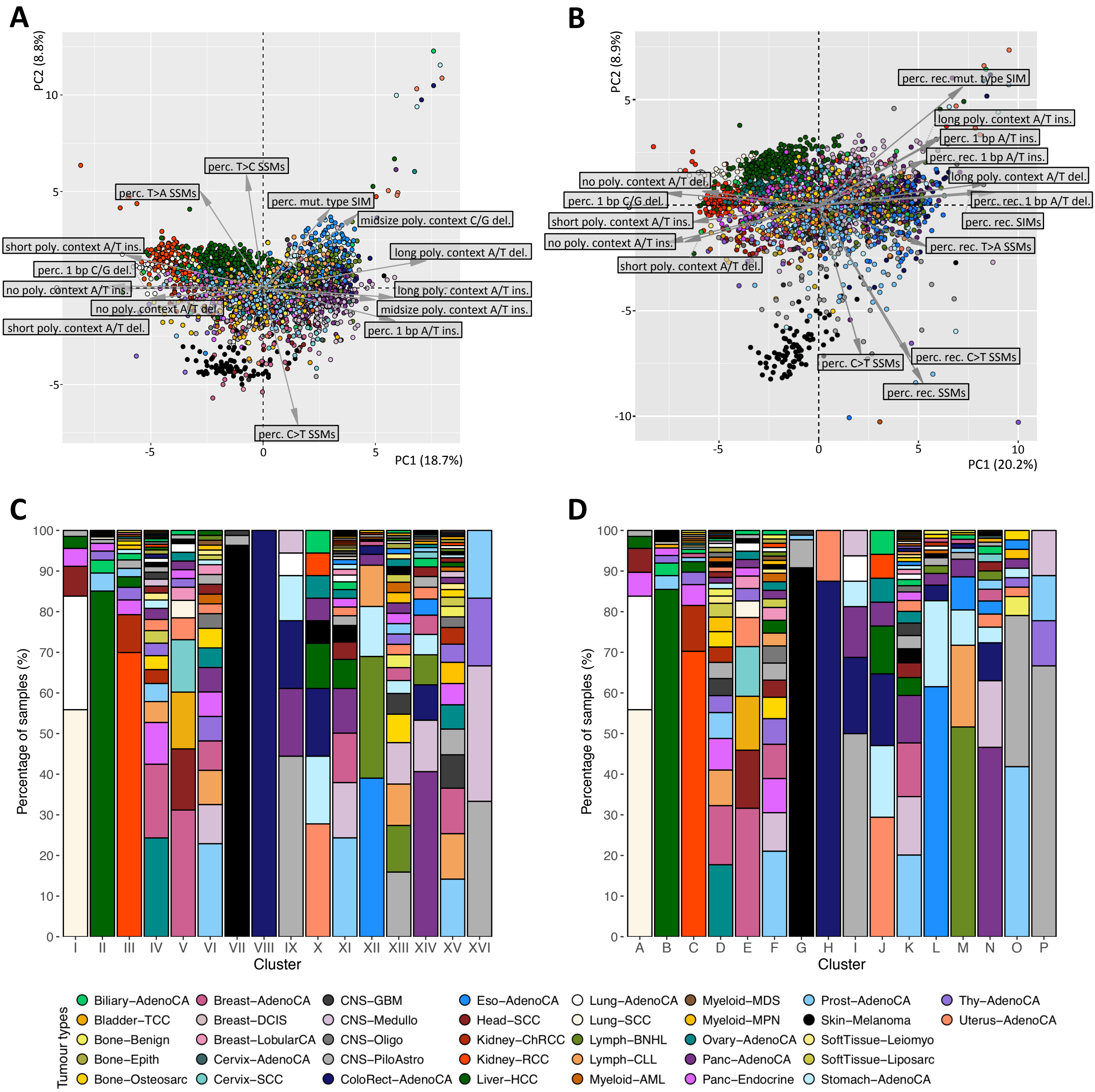

E

Lymph-CLL samples

Using only the general features

Cluster	I	II	III	IV	V	VI	VII	VIII	IX	X	XI	XII	XIII	XIV	XV	XVI
WITH…	0	0	0	2	0	6	0	0	0	0	0	19	8	2	3	0
WITHOUT…	0	0	0	13	0	0	0	0	0	0	1	0	8	0	27	0

…hypermutation of the immunoglobulin genes

Using the general and recurrence-related features

Cluster	A	B	C	D	E	F	G	H	I	J	K	L	M	N	O	P
WITH…	0	0	0	2	0	2	0	0	0	0	0	0	36	0	0	0
WITHOUT…	0	1	1	42	0	0	0	0	0	0	4	0	1	0	0	0

…hypermutation of the immunoglobulin genes
