# Supplementary material for: Recurrent somatic mutations reveal new insights into consequences of mutagenic processes in cancer
Source: PLoS Comput Biol. 2019 Nov 25;15(11):e1007496. doi: 10.1371/journal.pcbi.1007496 (PMC6901237; doi:10.1371/journal.pcbi.1007496)
Supplement: S1 Table — (PDF) [file pcbi.1007496.s005.pdf]

Table S1. Tumour type abbreviation, full name and number of samples.

Abbreviation	Full name	Number of samples
Biliary-AdenoCA	biliary adenocarcinoma	34
Bladder-TCC	bladder transitional cell carcinoma	23
Bone-Benign	benign neoplasm of the bone	16
Bone-Epith	epithelial neoplasm of bone	10
Bone-Osteosarc	bone osteosarcoma	35
Breast-AdenoCA	breast adenocarcinoma	195
Breast-DCIS	breast ductal carcinoma in situ	3
Breast-LobularCA	breast lobular carcinoma	13
Cervix-AdenoCA	cervical adenocarcinoma	2
Cervix-SCC	cervical squamous cell carcinoma	18
CNS-GBM	central nervous system - glioblastoma multiforme	39
CNS-Medullo	central nervous system - medulloblastoma	141
CNS-Oligo	central nervous system - oligodendroglioma	18
CNS-PiloAstro	central nervous system - pilocytic astrocytoma	89
ColoRect-AdenoCA	colorectal adenocarcinoma	52
Eso-AdenoCA	oesophageal adenocarcinoma	97
Head-SCC	head/neck squamous cell carcinoma	56
Kidney-ChRCC	chromophobe renal cell carcinoma	43
Kidney-RCC	renal cell carcinoma	143
Liver-HCC	hepatocellular carcinoma	314
Lung-AdenoCA	lung adenocarcinoma	37
Lung-SCC	lung squamous cell carcinoma	47
Lymph-BNHL	B-cell non-Hodgkin lymphoma	107
Lymph-CLL	chronic lymphocytic leukaemia	90
Myeloid-AML	acute myeloid leukaemia	13
Myeloid-MDS	myelodysplastic syndromes	2
Myeloid-MPN	myeloproliferative neoplasm	23
Ovary-AdenoCA	ovarian adenocarcinoma	110
Panc-AdenoCA	pancreatic adenocarcinoma	232
Panc-Endocrine	pancreatic endocrine neoplasm	81
Prost-AdenoCA	prostate adenocarcinoma	199
Skin-Melanoma	skin melanoma	107
SoftTissue-Leiomyo	soft tissue leiomyosarcoma	15
SoftTissue-Liposarc	soft tissue liposarcoma	19
Stomach-AdenoCA	stomach adenocarcinoma	68
Thy-AdenoCA	thyroid adenocarcinoma	48
Uterus-AdenoCA	uterus adenocarcinoma	44
