# Supplementary material for: Recurrent somatic mutations reveal new insights into consequences of mutagenic processes in cancer
Source: PLoS Comput Biol. 2019 Nov 25;15(11):e1007496. doi: 10.1371/journal.pcbi.1007496 (PMC6901237; doi:10.1371/journal.pcbi.1007496)
Supplement: S2 Table — (PDF) [file pcbi.1007496.s006.pdf]

Table S2. Recurrence in pan-cancer context and within tumour type(s).

Recurrent in	Unique to tumour type(s) in which it is recurrent	Percentage of recurrent	
		SSMs	SIMs
pan-cancer context only		37.1%	79.8%
single tumour type	Yes	60.0%	10.7%
	No	2.8%	8.2%
multiple tumour types	Yes	0.1%	0.3%
	No	0.05%	1.0%

Overview of the percentages of SSMs and SIMs that are recurrent in a pan-cancer setting only, within a single tumour type and in multiple tumour types.
