# Supplementary material for: Recurrent somatic mutations reveal new insights into consequences of mutagenic processes in cancer
Source: PLoS Comput Biol. 2019 Nov 25;15(11):e1007496. doi: 10.1371/journal.pcbi.1007496 (PMC6901237; doi:10.1371/journal.pcbi.1007496)
Supplement: S1 Text — (PDF) [file pcbi.1007496.s007.pdf]

Estimation of the levels of recurrence when purely driven by chance

For the estimation of the levels of recurrence if only chance was the driving force we performed the following simulation in which we only take C+G content into account. All other factors that may influence the probability of recurrence (*e.g.* replication time) did not match our definition of chance. For each cancer genome we randomly sampled the same number of SSMs as had been observed in the sample and also kept the counts for each of the six SSM subtypes the same. To take into account the C+G content of the human genome, random numbers were sampled for the C>A/G/T SSMs within the range of 1 to 1,144,530,852, which corresponds to the number of C/G bases in the GRCh37/h19 genome. Once a number had been selected it could not be selected again for the same cancer genome. The same was done for the T>A/C/G mutations, where we sampled numbers within the range of 1 to 1,716,796,279. Simulations were repeated 5,000 times and for each simulation we computed the recurrence overall, recurrence per SSM subtype and for each tumour type the recurrence 'within tumour type' and 'pan-cancer' (Fig A). Only for the recurrence within tumour type there were cases for which there were simulations with an equal or higher number of recurrent SSMs than observed. For three tumour types (Breast-DCIS, Cervix-AdenoCA and Myeloid-MDS) the observed number of recurrent SSMs was zero and nearly all simulated values were also zero (<0.5% were higher). For another five tumour types (Bone-Epith, Breast-LobularCA, Kidney-ChRCC, Myeloid-AML and SoftTissue-Leiomyo) between 2 and 186 of the 5,000 simulated values were equal or higher.

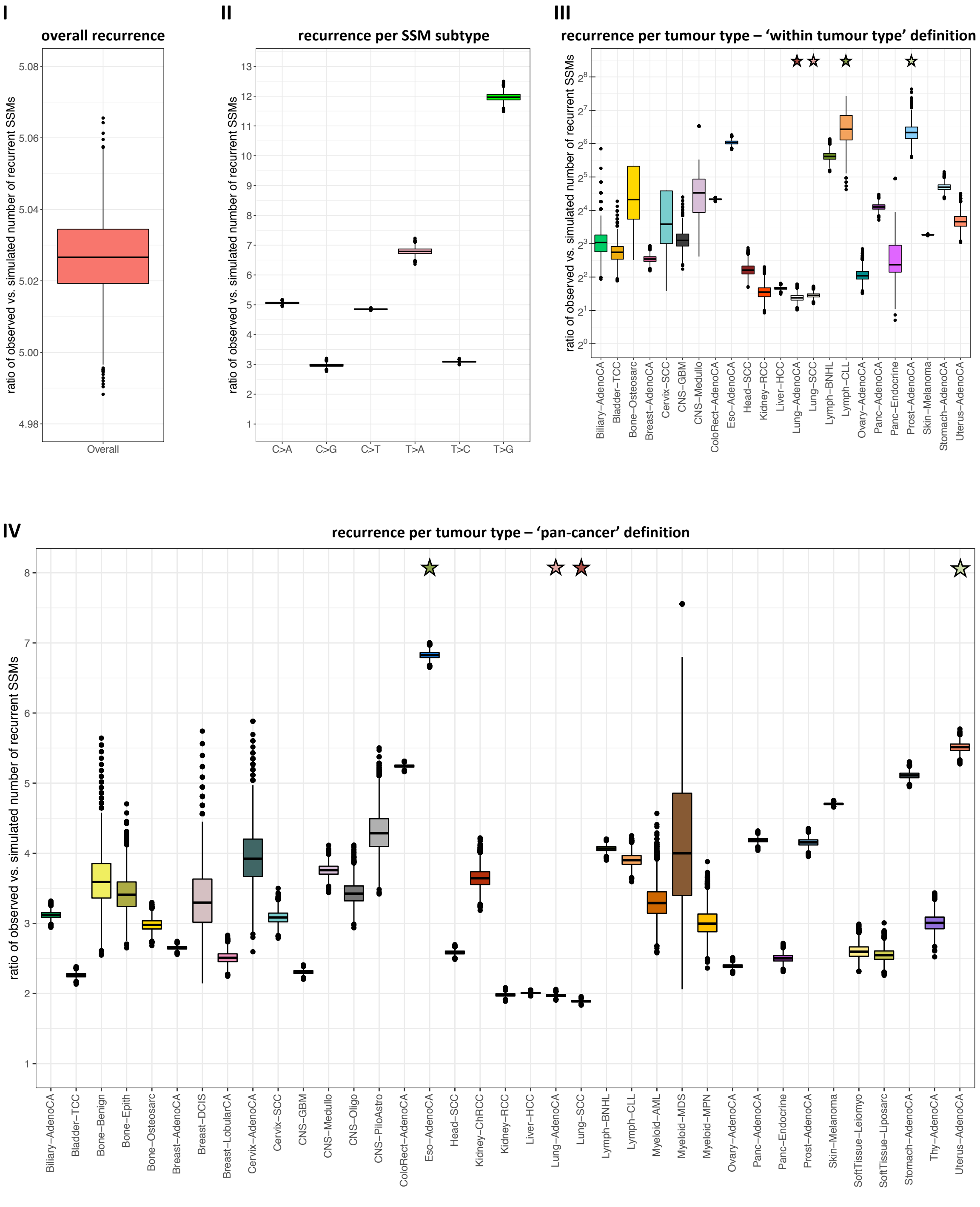

Fig A. Observed recurrence of SSMs versus what is expected by chance.

Each boxplot shows the ratio of the observed number of recurrent SSMs and the number of recurrent SSMs calculated in the simulation (N=5,000) in the following settings: (I) overall recurrence; (II) recurrence for each of the six SSM subtypes; (III) recurrence per tumour type using the 'within tumour type' definition; (IV) recurrence per tumour type using the 'pan-cancer' definition. The dark green stars at the top of plots III and IV indicate the tumour types with the highest median and the light green stars the second highest. The dark red ones indicate the lowest median and the light red ones the second lowest. For visualization purposes we left out in plot III the results of 14 tumour types for which >40% of the simulations resulted in zero recurrent SSMs, which led to a ratio that is infinite. For the boxplots of Bone-Osteosarc, Cervix-SCC, CNS-Medullo, Lymph-CLL and Panc-Endocrine we left out between 0.4% and 21.7% of the simulations in which no recurrent SSMs were found. In plot IV we left out for visualization purposes the results of 77 simulations for Myeloid-MDS that were all between 8.5 and 17.
