# Supplementary material for: Recurrent somatic mutations reveal new insights into consequences of mutagenic processes in cancer
Source: PLoS Comput Biol. 2019 Nov 25;15(11):e1007496. doi: 10.1371/journal.pcbi.1007496 (PMC6901237; doi:10.1371/journal.pcbi.1007496)
Supplement: S2 Text — (PDF) [file pcbi.1007496.s008.pdf]

Recurrence versus general mutational characteristics

We analyse recurrence in the context of the 29 general mutational features (S1 File) that capture effects of mutational processes. Recurrence is defined across the entire cohort ('pan-cancer'). We computed the Spearman's rank correlation coefficients (r_S) between every possible pair of the 42 features (861 correlations), which are shown in Fig 2 (**main**). An additional 149 correlations (plus two that are in common with Fig 2) are described here (Tables A to D), which include among others correlations on tumour type level and recurrence in absolute terms (as opposed to relative recurrence in Fig 2). Multiple testing correction was done using the Benjamini-Yekutieli method. False discovery rate for the 1,010 tests is controlled at 5%, *i.e.* we consider a correlation significant if the adjusted p-value is below 0.05.

Mutational load versus recurrence

The correlations between the total number and the number of recurrent SSMs and SIMs are positive for both cases, although stronger for SSMs than for SIMs (r_S=0.89 – SSMs, r_S=0.76 – SIMs). At tumour type level (Fig A and Table A), the significant correlation coefficients for SSMs range from 0.78 (CNS-PiloAstro) to 0.99 (Skin-Melanoma). For SIMs, they range from only 0.29 (Ovary-AdenoCA) to 0.97 (Lymph-BNHL), and for two tumour types the correlation coefficient is non-significant (Kidney-ChRCC and SoftTissue-Leiomyo). If we rank the tumour types according to the median total number of SSMs/SIMs, and compare this to the ranking based on the median number of recurrent SSMs/SIMs, we observe many shifts in both directions (Figs B and C). For example, Lung-SCC ranks second with respect to the median number of SSMs, but in terms of recurrent ones, it ranks fourth behind ColoRect-AdenoCA and Eso-AdenoCA (Fig B). Likewise, a low mutational burden per sample does not necessarily translate into a low number of recurrent ones. Panc-AdenoCA, for example, ranks 18th with respect to the median number of SSMs across samples, but 11th in terms of recurrent ones. For SIMs similar trends can be observed (Fig C). Lung-SCC samples have the highest median number of SIMs, but this tumour type ranks only sixth with respect to the median number of recurrent SIMs. The opposite trend is observed for Lymph-BNHL, which ranks 14th with regard to the median number of total SIMs across samples, but fourth when only considering recurrent ones.

Table A. Correlations between number of mutations and number of recurrent mutations.

	number of mutations vs. number of recurrent mutations			
	SSMs		SIMs	
	correlation	adjusted p-value	correlation	adjusted p-value
entire cohort	0.89	<3.1e-15	0.76	<3.1e-15
Biliary-AdenoCA	0.88	8.1e-11	0.90	5.8e-12
Bladder-TCC	0.91	2.1e-08	0.63	0.012
Bone-Benign	0.97	1.1e-08	0.79	0.0028
Bone-Epith	0.89	0.0058	0.87	0.011
Bone-Osteosarc	0.86	6.8e-10	0.68	7.1e-05
Breast-AdenoCA	0.90	<3.1e-15	0.65	<3.1e-15
Breast-LobularCA	0.98	1.9e-08	0.75	0.029
Cervix-SCC	0.90	4.3e-06	0.80	6.8e-04
CNS-GBM	0.86	3.5e-11	0.86	2.1e-11
CNS-Medullo	0.92	<3.1e-15	0.95	<3.1e-15
CNS-Oligo	0.98	5.0e-11	0.84	1.1e-04
CNS-PiloAstro	0.78	<3.1e-15	0.58	3.7e-08
ColoRect-AdenoCA	0.90	<3.1e-15	0.95	<3.1e-15
Eso-AdenoCA	0.96	<3.1e-15	0.96	<3.1e-15
Head-SCC	0.98	<3.1e-15	0.89	<3.1e-15
Kidney-ChRCC	0.84	4.1e-11	0.37	0.14
Kidney-RCC	0.85	<3.1e-15	0.57	1.4e-12
Liver-HCC	0.93	<3.1e-15	0.74	<3.1e-15
Lung-AdenoCA	0.96	<3.1e-15	0.54	0.0054
Lung-SCC	0.93	<3.1e-15	0.43	0.026
Lymph-BNHL	0.98	<3.1e-15	0.97	<3.1e-15
Lymph-CLL	0.87	<3.1e-15	0.74	<3.1e-15
Myeloid-AML	0.95	4.3e-06	0.78	0.016
Myeloid-MPN	0.88	2.4e-07	0.64	0.0092
Ovary-AdenoCA	0.83	<3.1e-15	0.29	0.020
Panc-AdenoCA	0.83	<3.1e-15	0.85	<3.1e-15
Panc-Endocrine	0.92	<3.1e-15	0.83	<3.1e-15
Prost-AdenoCA	0.85	<3.1e-15	0.92	<3.1e-15
Skin-Melanoma	0.99	<3.1e-15	0.55	1.4e-08
SoftTissue-Leiomyo	0.82	0.0017	0.55	0.30
SoftTissue-Liposarc	0.93	7.8e-08	0.81	2.9e-04
Stomach-AdenoCA	0.95	<3.1e-15	0.94	<3.1e-15
Thy-AdenoCA	0.90	<3.1e-15	0.66	3.1e-06
Uterus-AdenoCA	0.85	2.3e-12	0.73	1.8e-07

The indicated correlation is the Spearman's rank correlation coefficient. The tumour types Breast-DCIS, Cervix-AdenoCA and Myeloid-MDS are left out because they have four or less samples, which is too few to compute the correlation. Correlations in grey are not significant. In dark green is the highest correlation, light green the second highest, dark red the lowest and light red the second lowest.

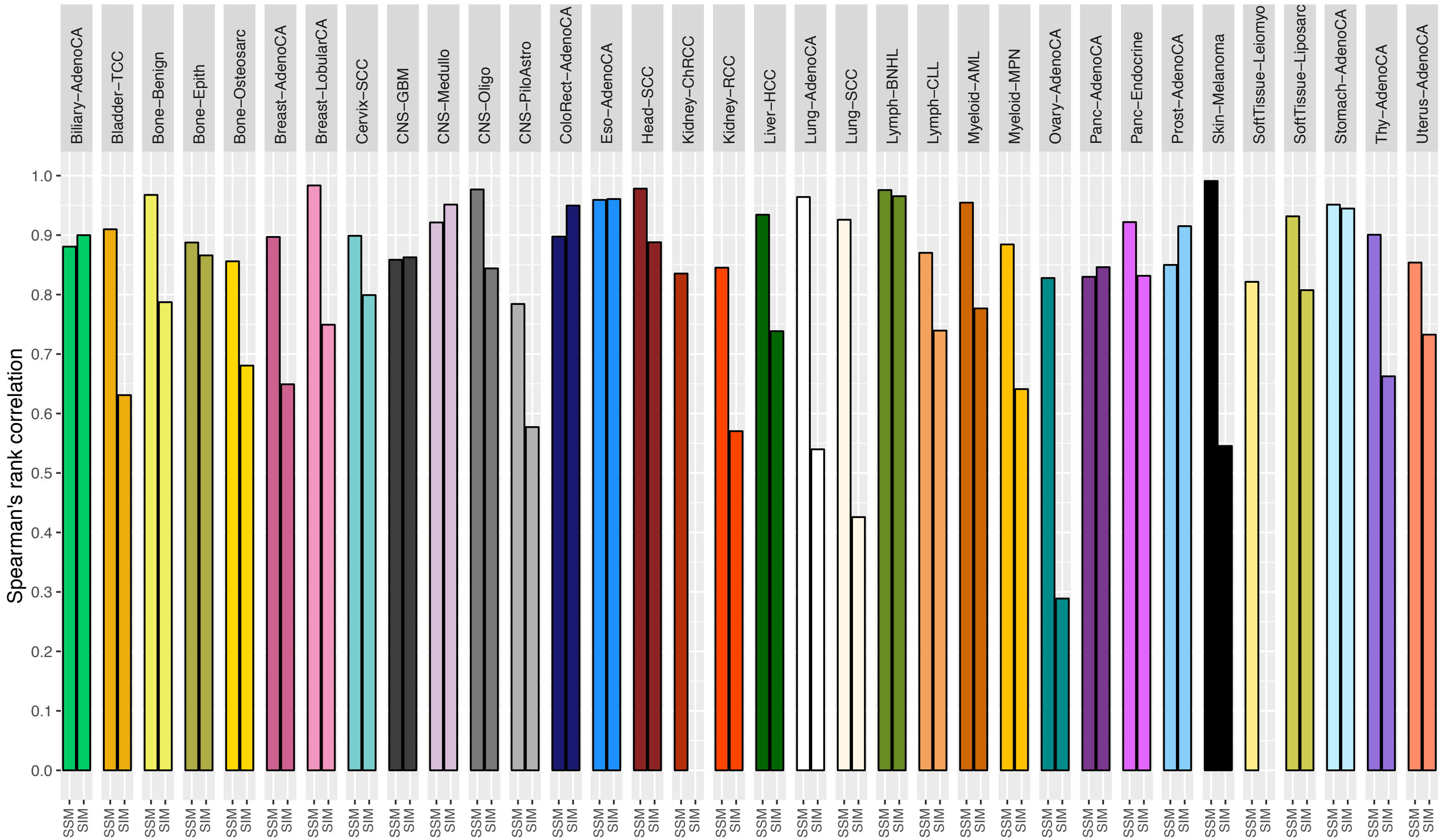

Fig A. Spearman's rank correlation between the total and recurrent number of SSMs and SIMs. The three tumour types with less than four samples are left out as the correlation could not be computed for them. Furthermore, for SIMs the correlation is not shown for Kidney-ChRCC and SoftTissue-Leiomyo as it was not significant.

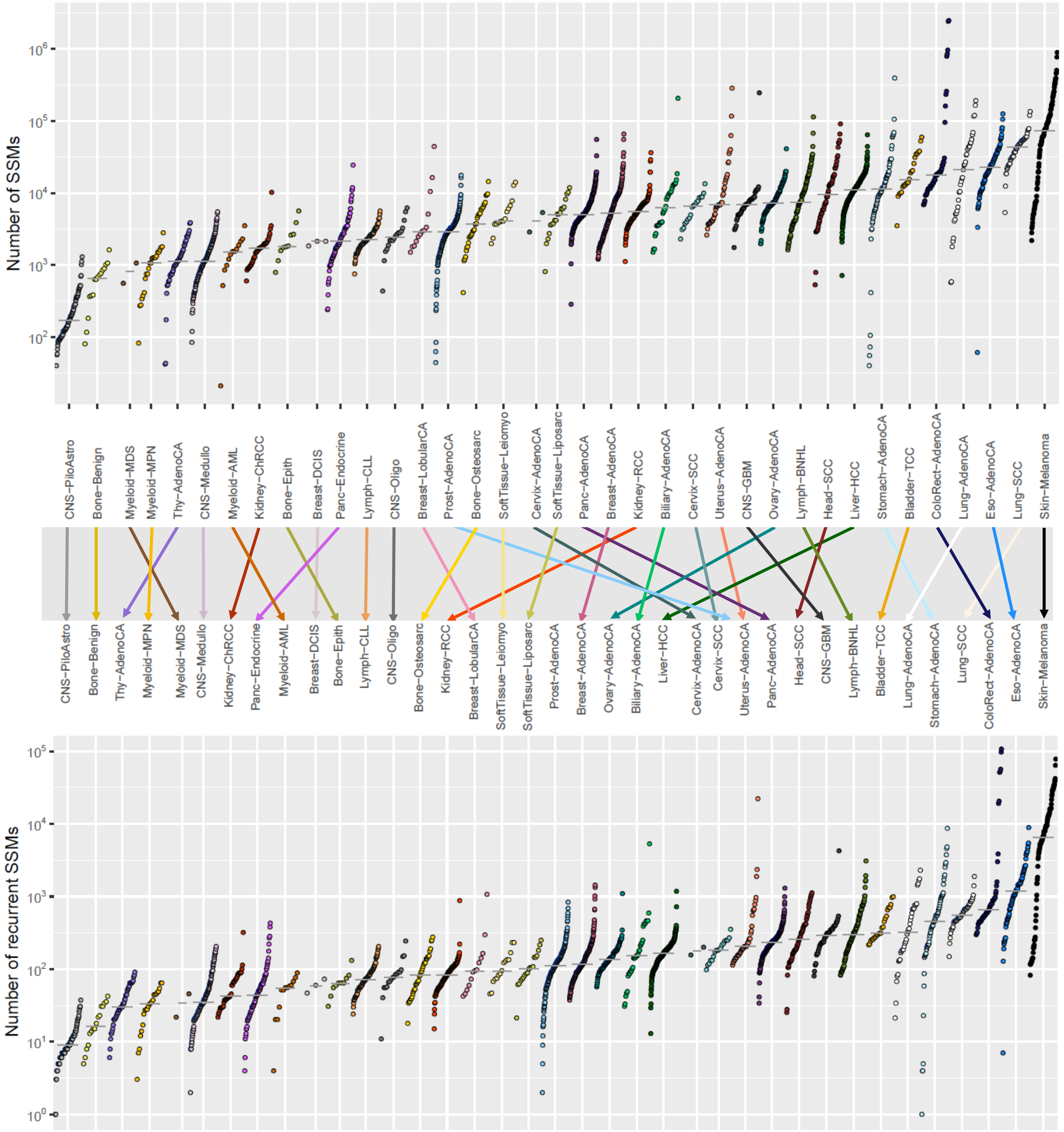

Fig B. Number of all and recurrent SSMs per tumour type.

Number of all SSMs (top) and number of recurrent SSMs (bottom) ordered by the median of each tumour type.

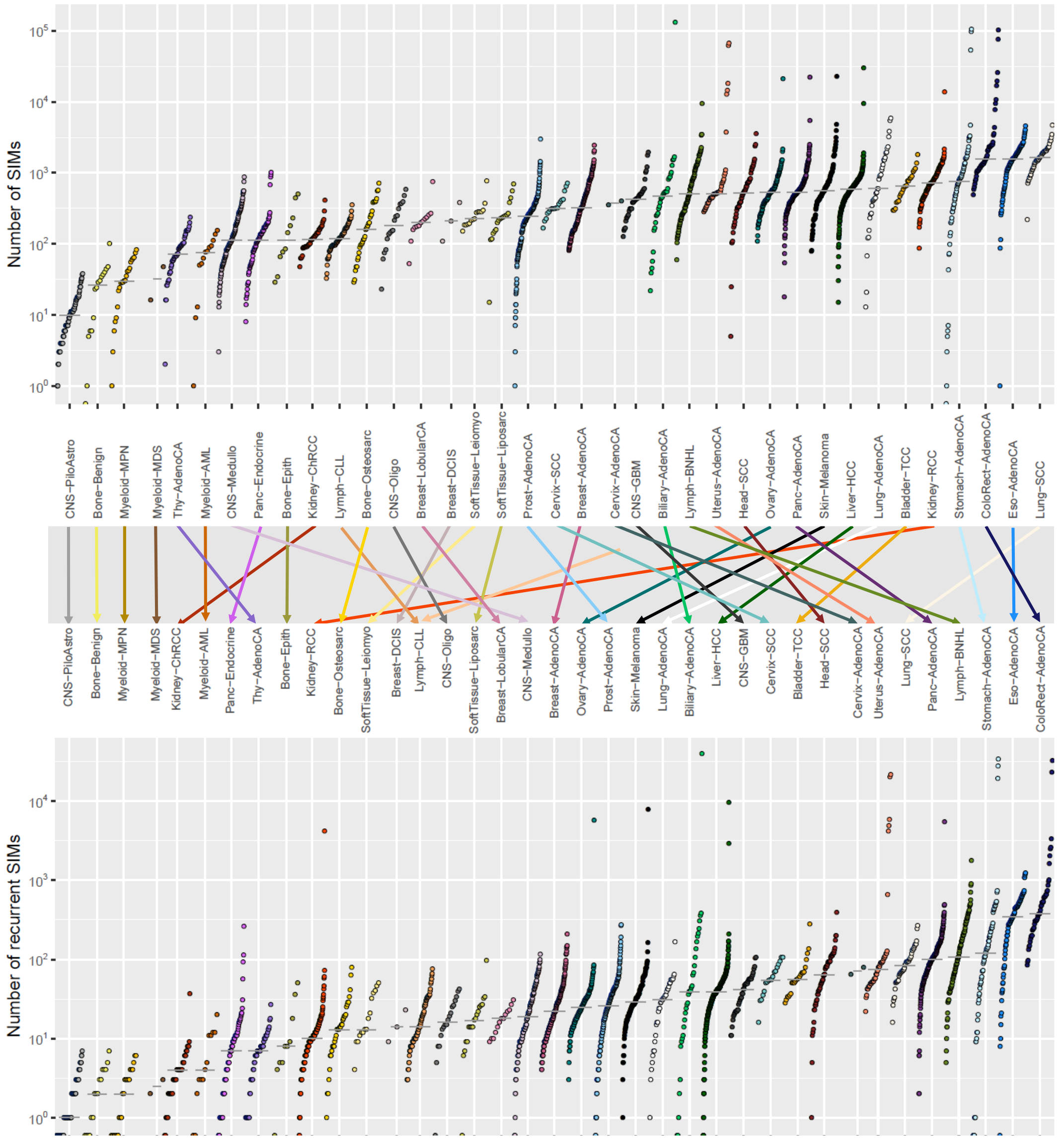

Fig C. Number of all and recurrent SIMs per tumour type.
Number of all SIMs (top) and number of recurrent SIMs (bottom) ordered by the median of each tumour type.

If we consider the percentage of recurrence instead of the absolute number, the general correlation for SSMs is weak and negative (r_S=-0.21). For SIMs this correlation is not significant (r_S=0.04), unless we only consider 1 bp SIMs in which case there is a weak positive correlation (r_S=0.18). If we look at the tumour types individually, the only positive correlations for SSMs that are statistically significant are found for Eso-AdenoCA (r_S=0.48) and Skin-Melanoma (r_S=0.58) (Fig D and Table B). For 15 other tumour types the correlation is negative, ranging from correlation coefficients of -0.82 for Bone-Epith to -0.27 for Kidney-RCC. For SIMs the correlation is positive for seven tumour types (*e.g.* Biliary-AdenoCA and Eso-AdenoCA) and negative for four others (*e.g.* Skin-

Melanoma and Lung-AdenoCA). Finally, Fig E further illustrates that neither a high nor a low median number of mutations across samples of a tumour type necessarily corresponds to the relative level of recurrence observed for the tumour type.

Table B. Correlations between the number of mutations and percentage of recurrent mutations.

	number of mutations vs. percentage of recurrent mutations			
	SSMs		SIMs	
	correlation	adjusted p-value	correlation	adjusted p-value
entire cohort	-0.21	<3.1e-15	0.04	0.43
Biliary-AdenoCA	0.13	1	0.71	2.6e-05
Bladder-TCC	-0.65	0.0074	-0.43	0.35
Bone-Benign	-0.28	1	0.11	1
Bone-Epith	-0.82	0.034	0.04	1
Bone-Osteosarc	-0.45	0.059	-0.26	1
Breast-AdenoCA	-0.65	<3.1e-15	-0.31	1.1e-04
Breast-LobularCA	-0.28	1	-0.14	1
Cervix-SCC	-0.74	0.0045	-0.01	1
CNS-GBM	-0.21	1	-0.19	1
CNS-Medullo	-0.01	1	0.18	0.31
CNS-Oligo	-0.07	1	-0.18	1
CNS-PiloAstro	-0.43	2.3e-04	0.08	1
ColoRect-AdenoCA	0.01	1	0.14	1
Eso-AdenoCA	0.48	5.4e-06	0.67	7.6e-13
Head-SCC	-0.80	2.4e-12	-0.33	0.12
Kidney-ChRCC	-0.03	1	-0.11	1
Kidney-RCC	-0.27	0.012	-0.11	1
Liver-HCC	-0.58	<3.1e-15	0.11	0.48
Lung-AdenoCA	-0.80	4.4e-08	-0.69	2.3e-05
Lung-SCC	-0.64	1.1e-05	-0.40	0.046
Lymph-BNHL	-0.48	1.4e-06	0.28	0.030
Lymph-CLL	0.05	1	0.37	0.0032
Myeloid-AML	-0.32	1	0.26	1
Myeloid-MPN	-0.31	1	0.23	1
Ovary-AdenoCA	-0.59	2.1e-10	-0.57	1.3e-09
Panc-AdenoCA	-0.31	1.9e-05	0.27	2.3e-04
Panc-Endocrine	-0.09	1	0.27	0.1
Prost-AdenoCA	-0.16	0.17	0.24	0.0073
Skin-Melanoma	0.58	9e-10	-0.51	2.4e-07
SoftTissue-Leiomyo	-0.38	1	0.09	1
SoftTissue-Liposarc	-0.54	0.15	0.36	1
Stomach-AdenoCA	0.09	1	0.56	6.8e-06
Thy-AdenoCA	-0.45	0.014	-0.25	0.70
Uterus-AdenoCA	-0.59	2.5e-04	0.27	0.60

The indicated correlation is the Spearman's rank correlation coefficient. The tumour types Breast-DCIS, Cervix-AdenoCA and Myeloid-MDS are left out because they have four or less samples, which is too few to compute the correlation. Correlations in grey are not significant. In dark green is the highest correlation, light green the second highest, dark red the lowest and light red the second lowest.

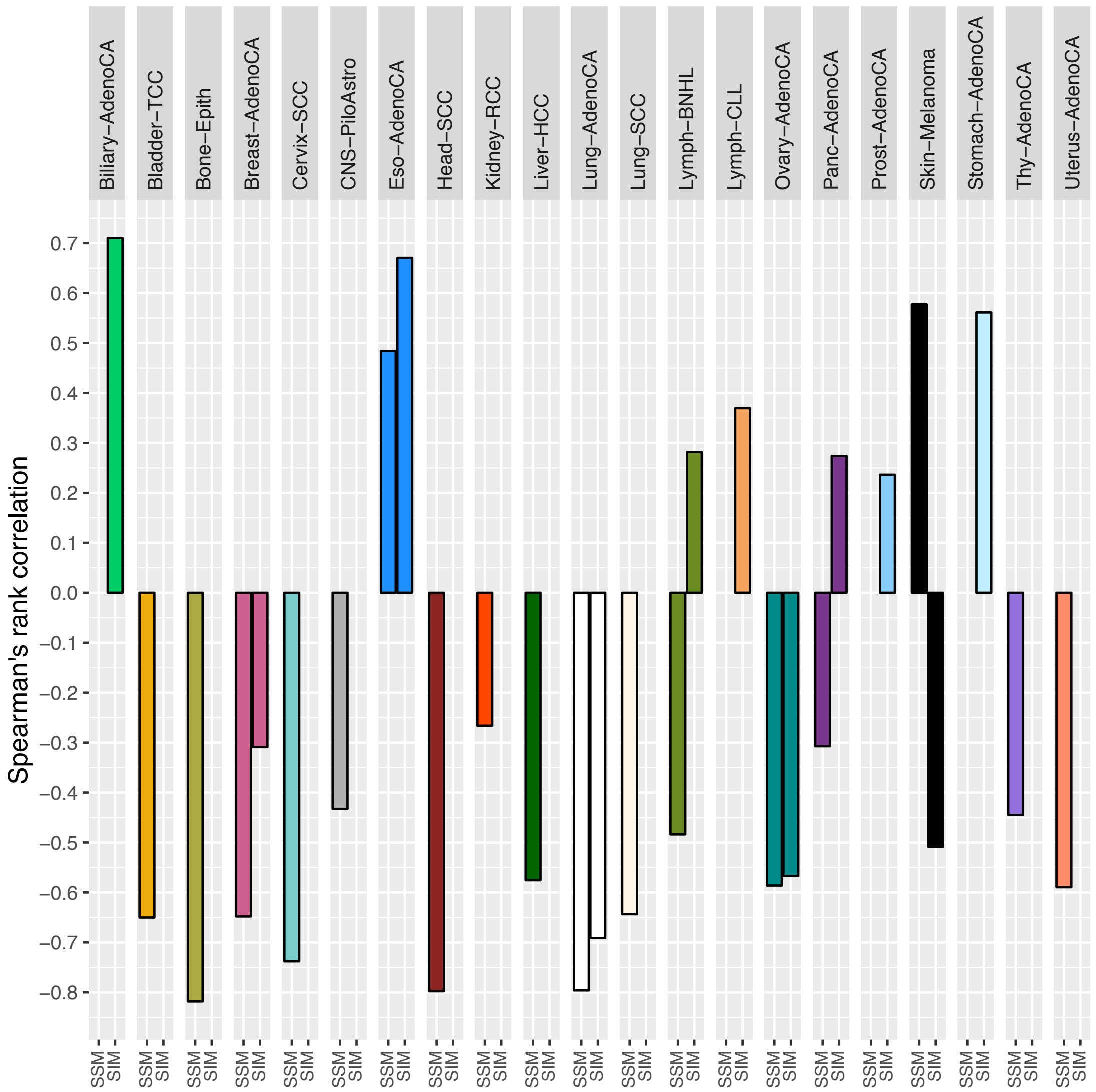

Fig D. Spearman's rank correlation between the total number and percentage of recurrent SSMs and SIMs.

The correlation is only shown for those tumour types for which it is significant.

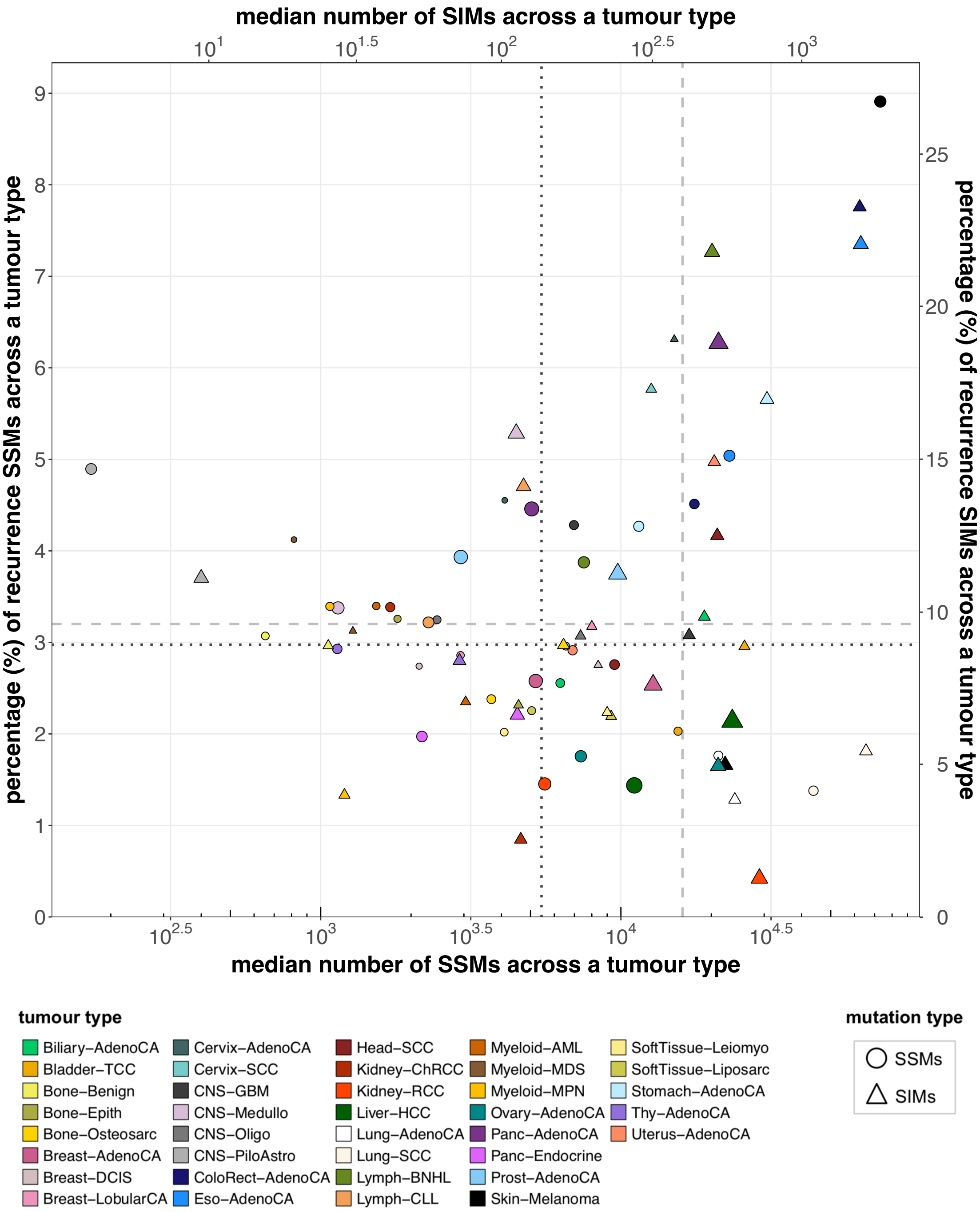

Fig E. Median number of mutations vs. median percentage of recurrent mutations per tumour type. The median number of SSMs (circle) and SIMs (triangle) across a tumour type is shown on the two horizontal axes (log-scale). The median percentage of recurrent SSMs and SIMs are shown on the two vertical axes. Recurrence is defined 'pan-cancer'. The size of circle/triangle corresponds to the number of samples of that tumour type. The dotted lines indicate the median across the cohort for the total number and percentage of recurrent SSMs and the dashed lines the corresponding values for SIMs.

Ratio of SSMs and SIMs versus recurrence

There are 1,057,935 recurrent SSMs and 186,576 recurrent SIMs, which represent 2.44% and 8.69% of the total number of SSMs and SIMs, respectively,

found in the PCAWG cohort (Fig F). The 5.7-fold difference in absolute number of recurrent mutations reflects the much higher total number of SSMs in a cancer genome. In relative terms, however, the percentage of recurrent SIMs is 3.6-fold higher. For recurrent mutations in three or more samples this ratio in relative terms increases to 10-fold (Fig F-I). In absolute numbers the SIMs even surpass the SSMs if presence in at least five samples is required (Fig F-II).

I

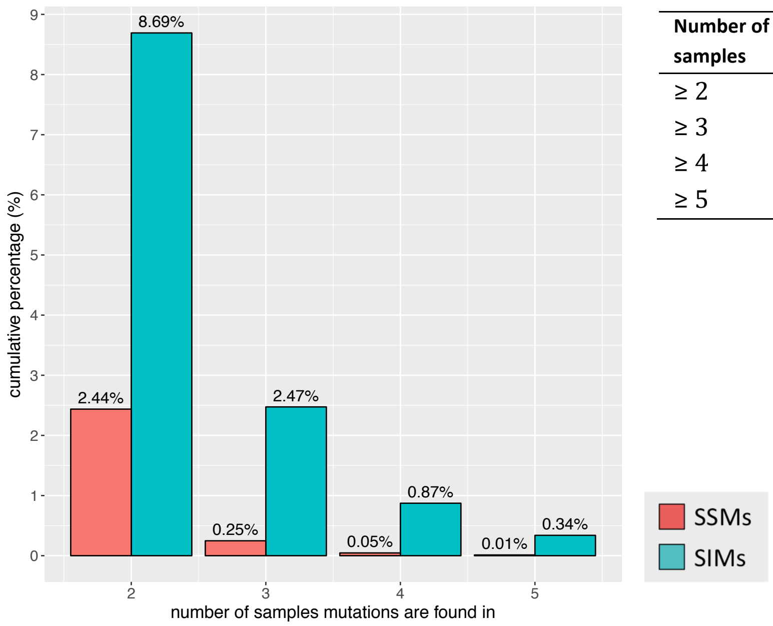

II

Number of samples	Number of SSMs	Number of SIMs
≥ 2	1,057,935	186,576
≥ 3	107,472	53,111
≥ 4	20,006	18,727
≥ 5	5,326	7,266

Fig F. Level of recurrence for SSMs and SIMs.
(A) Cumulative percentage of recurrent SSMs and SIMs found in two to five samples. (B) Absolute number of recurrent SSMs and SIMs found in at least two to five samples.

At a per sample basis, there are 45 samples for which more than half of their recurrent mutations are SIMs. Across all samples the median percentage of SIMs of the recurrent mutations is ~17%. However, when looking at mutations independent of recurrence, only one sample has a majority of SIMs and the median percentage overall is just ~6%. The percentages of total and recurrent mutations of type SIM vary strongly across the tumour types (Fig G). For the majority of the tumour types the percentage of recurrent mutations of type SIM tends to be higher than the percentage of all mutations of type SIM. Skin-Melanoma, Kidney-ChRCC and Kidney-RCC do not follow this trend.

Mutation subtypes versus recurrence

The C>T transitions have with 4.19% (Table C) the highest percentage of recurrence of the six SSM subtypes. Skin-Melanoma samples alone, which have a

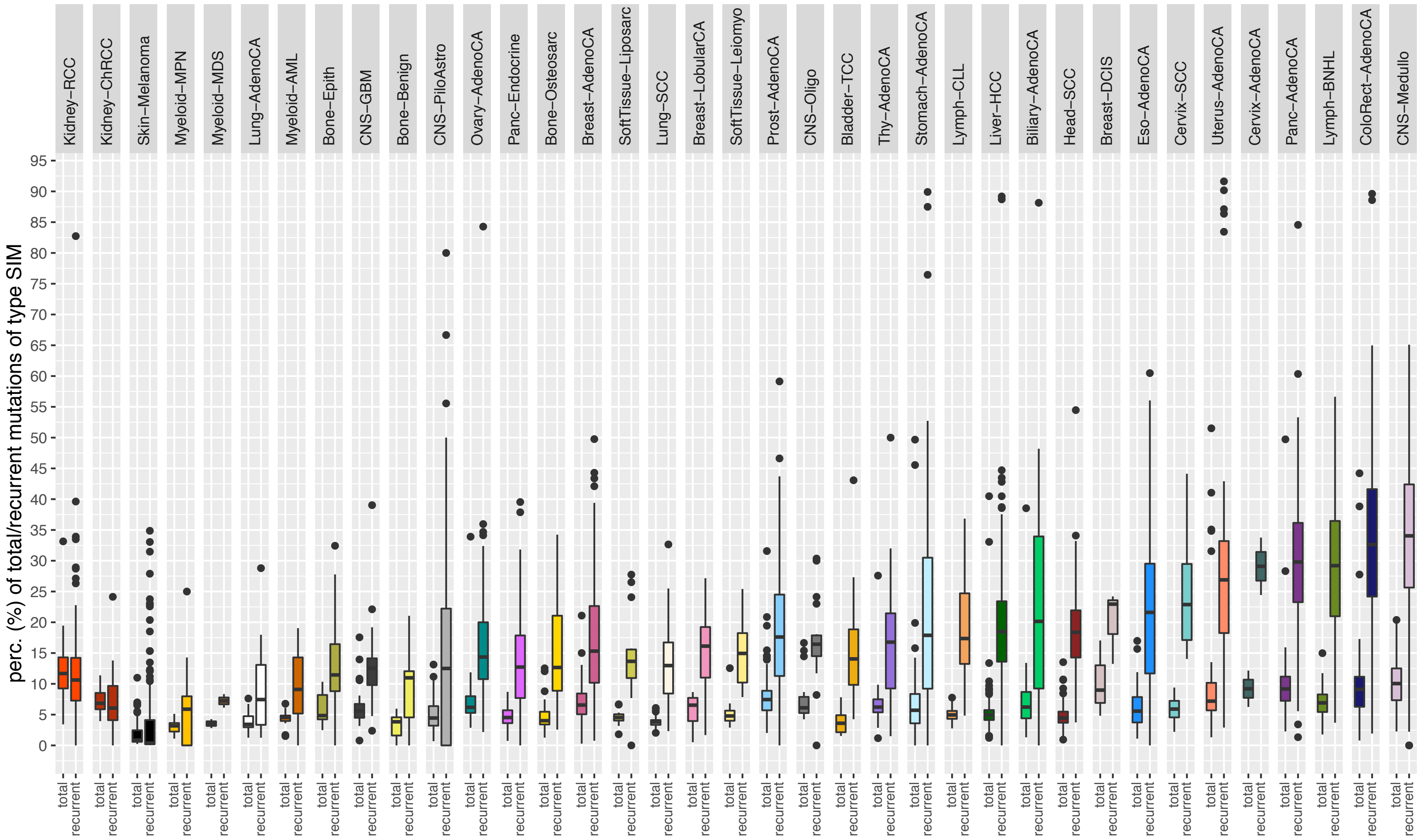

Fig G. Percentage of total/recurrent mutations of type SIM. The first of every pair of boxplots represents the percentage of all mutations of type SIM and the second the percentage of recurrent mutations of type SIM. The tumour types are ordered according to the difference in median between the two percentages.

Table C. Total and recurrent mutations per SSM and SIM subtype.

Mutation type			All mutations			Recurrent mutations			
			number	median per sample	mean per sample	number	percentage of total	median per sample	mean per sample
SSM		C>A	7,959,694	941	3139.5	141,747	1.78%	16	112.8
		C>G	2,588,475	473	1006.1	8,856	0.34%	3	7.4
		C>T	18,121,448	1,809	7349.6	758,411	4.19%	85	627.6
		T>A	3,473,029	613	1356.8	25,167	0.72%	11	22.0
		T>C	6,393,997	871	2491.7	38,336	0.60%	9	31.1
		T>G	4,871,710	385	1924.6	85,418	1.75%	5	71.6
		Total	43,408,353	5,439	17268.3	1,057,935	2.44%	143	872.5
SIM (1 bp)	deletion	A/T	780,494	88	374.6	119,160	15.27%	8	118.5
		C/G	238,272	40	94.7	4,437	1.86%	1	4.2
	insertion	A/T	570,960	97	246.2	53,103	9.30%	15	45.8
		C/G	70,067	10	28.7	2,991	4.27%	1	2.8
	Total		1,659,793	263	744.3	179,691	10.83%	27	171.2

The difference between the median and the mean gives a measure of the skewness of the distribution of the number of (recurrent) mutations across samples. Note that here we only included for SIMs those of 1 bp in length. The total number of SIMs of any length is 2,146,551 of which 186,576 are recurrent (8.69%).

very high percentage of C>T SSMs, account for 62.2% of the recurrent C>T SSMs. In line with this, there is a positive correlation between the percentage of recurrent SSMs and the percentage of C>T SSMs (r_S=0.62, Fig 2 – **main**), whereas it is negative or non-significant for all the other SSM subtypes. The C>A transversions rank second in terms of the percentage that is recurrent (1.78%) and ColoRect-AdenoCA contributes more than half of the recurrent C>A SSMs. Ranking third are the T>G SSMs (1.75% recurrent), even though this subtype ranks only fourth in terms of total number. Furthermore, this SSM subtype is the only one for which the total number and the percentage of recurrent ones shows a positive correlation (r_S=0.20) (Table D), which suggests a particularly strong tendency towards non-randomness. This is line with the fact that the observed recurrence is twelve times higher than expected by chance, which is the highest difference in ratio between observed and simulated of all six SSM subtypes (S1 Text). Eso-AdenoCA samples harbour 31.4% of the recurrent T>G SSMs. For C>G, T>A and T>C SSM subtypes recurrence is below 1% and there is no single tumour type of which the samples contain a large proportion of the recurrent mutations.

Table D. Correlations between the number of SSMs per subtype and the percentage recurrent SSMs per subtype.

	SSM subtypes	
	correlation	adjusted p-value
C>A	-0.11	6.9e-08
C>G	-0.05	0.15
C>T	-0.14	3.1e-12
T>A	-0.19	<3.1e-15
T>C	-0.06	0.014
T>G	0.20	<3.1e-15

The indicated correlation is the Spearman's rank correlation coefficient. The correlation in grey is not significant.

The highest level of recurrence of the four 1 bp SIM subtypes is observed for 1 bp A/T deletions with 15.27% (Table C). This is followed by 1 bp A/T insertions with 9.30%. For the 1 bp C/G SIMs, the percentage of recurrent insertions (4.27%) is higher than the corresponding value of deletions (1.86%). For all four SIM subtypes there is a positive correlation between the total number and the percentage that is recurrent (ranging from r_S=0.24 to r_S=0.36, Table E). There is no single tumour type that by itself accounts for a large percentage of the recurrent SIMs. The two highest proportions are 12.2% and 10.6% for the 1 bp A/T and C/G deletions, respectively, both accounted for by the Stomach-AdenoCA samples.

Table E. Correlations between the number of SIM per subtype and the percentage recurrent SIMs per subtype.

	1 bp SIM subtypes	
	**correlation**	**adjusted p-value**
A/T deletions	0.27	<3.1e-15
C/G deletions	0.24	<3.1e-15
A/T insertions	0.24	<3.1e-15
C/G insertions	0.36	<3.1e-15

The indicated correlation is the Spearman's rank correlation coefficient.

Homopolymer context of 1 bp SIMs versus recurrence

One general mechanism that can result in SIMs is replication slippage in repetitive regions. As a proxy for this, we look at the homopolymer context of 1 bp SIMs. The percentage of recurrent SIMs positively correlates with the percentage of 1 bp A/T deletions in a long homopolymer context (r_S=0.75) (Fig 2 – **main**), *i.e.* a mononucleotide repeat of at least eight dA or dT. For each of the four SIM subtypes individually there is a strong correlation between the percentage that is recurrent and the percentage in a long homopolymer context (r_S=0.66-0.90, Fig 2 – **main**). For the recurrent 1 bp A/T SIMs, one factor that could help explain this is that ~35% and ~31% of all 1 bp A/T deletions and insertions, respectively, are in this context (Table F). In contrast, only ~1% and ~4% of all 1 bp C/G deletions and insertions, respectively, are in this context. However, the number of long C/G homopolymers in the genome is ~62 times lower than long A/T homopolymers (Table F). Therefore, the low percentages of 1 bp C/G SIMs in this context can nevertheless translate into high relative levels of recurrence (Table F).

Table F. Sequence characteristics of the location of 1 bp SIMs.

I

**whole genome**		**1 bp A/T deletions**						
length A/T repeat	frequency	total num. mutations	perc. of total num. mutations	perc. of total num. in genome	num. recurrent mutations	perc. of total num. recurrent mutations	perc. recurrent of num. in genome	perc. recurrent of total num. mutated
1 bp	790,532,160	48,449	6.21%	0.01%	85	0.07%	<0.01%	0.18%
2 bp	208,951,666	49,290	6.32%	0.02%	90	0.08%	<0.01%	0.18%
3 bp	81,250,925	42,338	5.42%	0.05%	122	0.10%	<0.01%	0.29%
4 bp	31,072,823	55,952	7.17%	0.18%	268	0.22%	<0.01%	0.48%
5 bp	11,916,965	76,497	9.80%	0.64%	579	0.49%	<0.01%	0.76%
6 bp	3,665,679	87,945	11.27%	2.40%	3,355	2.82%	0.09%	3.81%
7 bp	1,523,245	148,677	19.05%	9.76%	16,433	13.79%	1.08%	11.05%
8 bp	552,745	141,564	18.14%	25.61%	39,676	33.30%	7.18%	28.03%
9 bp	316,551	117,795	15.09%	37.21%	55,190	46.32%	17.43%	46.85%
≥ 10 bp	1,003,583	11,987	1.54%	1.19%	3,362	2.82%	0.33%	28.05%

II

**whole genome**		**1 bp C/G deletions**						
length C/G repeat	frequency	total num. mutations	perc. of total num. mutations	perc. of total num. in genome	num. recurrent mutations	perc. of total num. recurrent mutations	perc. recurrent of num. in genome	perc. recurrent of total num. mutated
1 bp	649,178,072	68,855	28.90%	0.01%	146	3.29%	<0.01%	0.21%
2 bp	165,431,921	55,697	23.38%	0.03%	92	2.07%	<0.01%	0.17%
3 bp	43,760,744	36,815	15.45%	0.08%	72	1.62%	<0.01%	0.20%
4 bp	10,658,485	21,587	9.06%	0.20%	91	2.05%	<0.01%	0.42%
5 bp	2,519,277	22,561	9.47%	0.90%	228	5.14%	0.01%	1.01%
6 bp	500,193	19,488	8.18%	3.90%	782	17.62%	0.16%	4.01%
7 bp	76,327	10,325	4.33%	13.53%	1,664	37.50%	2.18%	16.12%
8 bp	15,210	2,573	1.08%	16.92%	1,137	25.63%	7.48%	44.19%
9 bp	5,900	249	0.10%	4.22%	153	3.45%	2.59%	61.45%
≥ 10 bp	9,166	122	0.05%	1.33%	72	1.62%	0.79%	59.02%

(continues below)

III

whole genome		1 bp A/T insertions						
length A/T repeat	frequency	total num. mutations	perc. of total num. mutations	perc. of total num. in genome	num. recurrent mutations	perc. of total num. recurrent mutations	perc. recurrent of num. in genome	perc. recurrent of total num. mutated
0 bp	-	12,968	2.27%	-	109	0.21%	-	0.84%
1 bp	790,532,160	26,517	4.64%	<0.01%	52	0.10%	<0.01%	0.20%
2 bp	208,951,666	21,913	3.84%	0.01%	59	0.11%	<0.01%	0.27%
3 bp	81,250,925	17,048	2.99%	0.02%	72	0.14%	<0.01%	0.42%
4 bp	31,072,823	20,589	3.61%	0.07%	59	0.11%	<0.01%	0.29%
5 bp	11,916,965	48,999	8.58%	0.41%	250	0.47%	<0.01%	0.51%
6 bp	3,665,679	120,529	21.11%	3.29%	4,142	7.80%	0.11%	3.44%
7 bp	1,523,245	126,228	22.11%	8.29%	9,689	18.25%	0.64%	7.68%
8 bp	552,745	107,778	18.88%	19.50%	21,142	39.81%	3.82%	19.62%
9 bp	316,551	62,678	10.98%	19.80%	16,139	30.39%	5.10%	25.75%
≥ 10 bp	1,003,583	5,713	1.00%	0.57%	1,390	2.62%	0.14%	24.33%

IV

whole genome		1 bp C/G insertions						
length C/G repeat	frequency	total num. mutations	perc. of total num. mutations	perc. of total num. in genome	num. recurrent mutations	perc. of total num. recurrent mutations	perc. recurrent of num. in genome	perc. recurrent of total num. mutated
0 bp	-	22,910	32.70%	-	104	3.48%	-	0.45%
1 bp	649,178,072	6,880	9.82%	<0.01%	22	0.74%	<0.01%	0.32%
2 bp	165,431,921	4,393	6.27%	<0.01%	26	0.87%	<0.01%	0.59%
3 bp	43,760,744	3,701	5.28%	0.01%	24	0.80%	<0.01%	0.65%
4 bp	10,658,485	6,555	9.36%	0.06%	20	0.67%	<0.01%	0.31%
5 bp	2,519,277	5,992	8.55%	0.24%	29	0.97%	<0.01%	0.48%
6 bp	500,193	7,474	10.67%	1.49%	144	4.81%	0.03%	1.93%
7 bp	76,327	9,461	13.50%	12.40%	1,334	44.60%	1.75%	14.10%
8 bp	15,210	2,534	3.62%	16.66%	1,214	40.59%	7.98%	47.91%
9 bp	5,900	131	0.19%	2.22%	60	2.01%	1.02%	45.80%
≥ 10 bp	9,166	36	0.05%	0.39%	14	0.47%	0.15%	38.89%

Overview of the number of (recurrent) 1 bp SIMs not in the context of a homopolymer (0 bp for deletions and 0-1 bp for insertions) and the number in the context of homopolymers of length 2 to ≥ 10 bp. In dark green is the highest number/percentage, light green the second highest, dark red the lowest and light red the second lowest.
