# Supplementary material for: Recurrent somatic mutations reveal new insights into consequences of mutagenic processes in cancer
Source: PLoS Comput Biol. 2019 Nov 25;15(11):e1007496. doi: 10.1371/journal.pcbi.1007496 (PMC6901237; doi:10.1371/journal.pcbi.1007496)
Supplement: S3 Text — (PDF) [file pcbi.1007496.s009.pdf]

Detailed cluster-specific descriptions

Here we describe in more detail the level of recurrence, the mutational pattern observed and possible mechanisms associated with each cluster (Fig 4 – **main**). We will describe only the most characteristic features for each cluster. An overview of the levels of recurrence within each cluster and across the entire cohort is shown in Table A for the six SSM subtypes and in Table B for the 1 bp SIM subtypes. Furthermore, there were several types of annotation available, provided either on sample or mutation level, which we overlay onto each cluster. The boxplots for the statistics related to the annotation layers on mutation level are shown in Fig A and an overview of the median values is provided in Table C as a summary. A more detailed view of the distribution of the mutations in terms of replication time on a sample level in each cluster is shown in Figs B (SSMs) and C (SIMs). Table D shows the top three predicted drivers for each cluster. Finally, there are three types of mutational signatures based on different types of mutations: single base substitutions (SBS), doublet base substitutions (DBS), and insertions/deletions (ID). Note that doublet base substitutions were considered as single events in the PCAWG Mutational Signatures Working Group. We will use the aforementioned abbreviations when referring to the different types of signatures. For each cluster, the top three for each type of signatures are provided in Table E, including a short description of the proposed aetiology that has been linked to the signatures [1].

Annotations on sample level

On sample level the annotation includes tumour type, MSI status, IGHV mutation status (Lymph-CLL only) and tobacco smoking history of the corresponding donor. We used two MSI classifications provided by the PCAWG consortium, one by the Mutational Signatures Working Group (unpublished) and one by the Germline Cancer Genome Working Group [2]. The first method, which we will refer to as **MSI-Method 1,** classified a sample MSI if the proportion of mutated microsatellites versus all testable microsatellites was >0.01. The second (**MSI-Method 2**) used the MSIsensor tool [3] to determine the percentage of mutated microsatellites and adjusted this percentage with a linear model for factors like age at diagnosis, sex and sample purity [2]. Cancer genomes with an adjusted percentage of >3.5% were considered MSI. The IGHV mutation status of Lymph-CLL samples was retrieved from the original article in which these samples were first described [4]. The tobacco smoking history was provided for a subset of the donors by the PCAWG consortium. We were able to retrieve this information for an additional set of PCAWG donors that are also part of TCGA, directly via the TCGA webportal.

Annotations on mutation level

On mutation level the annotation includes impact classification, functional category, the overlap with predicted drivers linked to the samples, mutational signatures and replication time. The functional impact of a mutation was predicted by Oncotator [5], which was made available by the consortium for 99.9% of the SSMs and 97.3% of the SIMs. We used GENCODE v19 [6] as annotation of the human reference to get the functional category of a mutation. The complete and 'per-patient' list of predicted cancer driver mutations together with the affected gene or regulatory element were provided by the PCAWG Drivers and Functional Interpretation Group [7, 8]. The mutational signatures and their proposed aetiology were provided by the corresponding working group [1]. We downloaded the replication time data in the form of wavelet-smoothed signals of the following five cancer cell lines from the ENCODE project [9]: HeLa-S3 (cervical adenocarcinoma), HepG2 (hepatocellular carcinoma), K562 (chronic myelogenous leukemia), MCF-7 (breast adenocarcinoma) and SK-N-SH (neuroblastoma). The scores were defined for regions of 1 kb in length, but were unavailable for chromosome Y and the chromosomal end regions. Mutations in these regions were left out. Furthermore, samples with less than ten SSMs/SIMs were left out of the density plots. As there was no matching cell line for several tumour types in the PCAWG cohort, we combined the cell lines by taking the median of the scores. To determine the percentage of mutations of a sample that falls into late-replicating regions, we took as a threshold the median score across the summarized scores of the five cell lines.

Identification of peaks of recurrent mutations

Specifically for the Lymph-BNHL and Lymph-CLL samples in cluster M, we identified peaks of recurrent mutations by sliding a window across the genome and computing per window the number of recurrent mutations, using the entire cohort to define recurrence. The size of a window was set to 11,415 bp such that it would contain on average ten recurrent mutations if all recurrent mutations (SSMs and SIMs) were equally distributed along the genome. At each step the window was shifted by half its size.

Table A. Cluster-specific overview of total and recurrent SSMs per subtype.

Cluster		Total number (median)						Number recurrent (percentage)											
		C>A	C>G	C>T	T>A	T>C	T>G	C>A		C>G		C>T		T>A		T>C		T>G	
								within cluster	entire cohort	within cluster	entire cohort	within cluster	entire cohort	within cluster	entire cohort	within cluster	entire cohort	within cluster	entire cohort
A	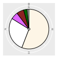	1,317,347 (17,004)	448,934 (6,133.5)	645,301 (8,482.5)	429,710 (5,630)	415,818 (5,886)	121,338 (1,604)	1,944 (0.1%)	13,266 (1.0%)	212 (<0.1%)	2,073 (0.5%)	384 (0.1%)	19,243 (3.0%)	167 (<0.1%)	2,135 (0.5%)	168 (<0.1%)	2,836 (0.7%)	14 (<0.1%)	1,560 (1.3%)
B	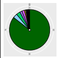	717,422 (2,009.5)	268,940 (744)	843,956 (2,401.5)	543,460 (1,475)	1,238,809 (3,577)	241,002 (662.5)	580 (0.1%)	8,607 (1.2%)	167 (0.1%)	1,271 (0.5%)	880 (0.1%)	26,312 (3.1%)	389 (0.1%)	3,981 (0.7%)	1,356 (0.1%)	7,815 (0.6%)	277 (0.1%)	2,500 (1.0%)
C	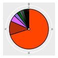	183,556 (911)	104,418 (499)	252,527 (1,309)	194,296 (633)	162,667 (768)	92,458 (444)	28 (<0.1%)	2,012 (1.1%)	14 (<0.1%)	409 (0.4%)	108 (<0.1%)	9,262 (3.7%)	24 (<0.1%)	830 (0.4%)	28 (<0.1%)	1,064 (0.7%)	10 (<0.1%)	529 (0.6%)
D	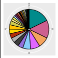	531,489 (639.5)	411,868 (355)	822,287 (1,252)	374,018 (420.5)	456,758 (612.5)	218,449 (226)	362 (0.1%)	6,881 (1.3%)	219 (0.1%)	2,186 (0.5%)	1673 (0.2%)	36,727 (4.5%)	255 (0.1%)	3,501 (0.9%)	243 (0.1%)	3,914 (0.9%)	124 (0.1%)	3,344 (1.5%)
E	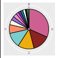	170,959 (1,262.5)	474,827 (2,982)	672,018 (4,487)	70,687 (489.5)	110,004 (705.5)	44,798 (304.5)	48 (<0.1%)	2,486 (1.5%)	655 (0.1%)	2,844 (0.6%)	1,116 (0.2%)	24,364 (3.6%)	22 (<0.1%)	1,350 (1.9%)	18 (<0.1%)	1,017 (0.9%)	9 (<0.1%)	792 (1.8%)
F	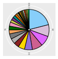	39,403 (351)	22,357 (183)	84,138 (866)	27,409 (238)	45,160 (416)	16,574 (155)	9 (<0.1%)	684 (1.7%)	3 (<0.1%)	199 (0.9%)	38 (<0.1%)	4,671 (5.6%)	8 (<0.1%)	554 (2.0%)	7 (<0.1%)	514 (1.1%)	1 (<0.1%)	235 (1.4%)
G	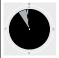	238,573 (2,139)	118,568 (1,023)	10,150,303 (76,575)	530,071 (3,304)	566,370 (4,403)	269,893 (1,959)	103 (<0.1%)	3,247 (1.4%)	15 (<0.1%)	477 (0.4%)	460,163 (4.5%)	607,157 (6.0%)	466 (0.1%)	2,438 (0.5%)	855 (0.2%)	4,053 (0.7%)	102 (<0.1%)	2,703 (1.0%)
H	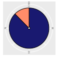	3,173,166 (297,750)	28,329 (2,226)	1,527,447 (177,687)	173,232 (13,197.5)	1,100,630 (103,035.5)	2,103,316 (176,074.5)	94,522 (3.0%)	118,703 (3.7%)	2 (<0.1%)	95 (0.3%)	34,075 (2.2%)	119,052 (7.8%)	575 (0.3%)	4,196 (2.4%)	896 (0.1%)	6,349 (0.6%)	13,244 (0.6%)	25,080 (1.2%)
I	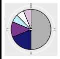	12,627 (17.5)	7,778 (9.5)	18,639 (62.5)	10,454 (11)	11,924 (24.5)	8,070 (10.5)	0 (0%)	241 (1.9%)	0 (0%)	41 (0.5%)	2 (<0.1%)	1,287 (6.9%)	0 (0%)	209 (2.0%)	0 (0%)	138 (1.2%)	1 (<0.1%)	212 (2.6%)
J	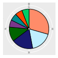	167,472 (5,846)	31,741 (1,675)	486,097 (15,688)	144,459 (4,485)	575,578 (19,892)	88,684 (4,197)	74 (<0.1%)	1,918 (1.1%)	56 (0.2%)	300 (0.9%)	1,938 (0.4%)	19,475 (4.0%)	642 (0.4%)	4,359 (3.0%)	1,150 (0.2%)	5,748 (1.0%)	260 (0.3%)	3,090 (3.5%)
K	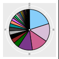	437,956 (611.5)	238,010 (289)	867,597 (1,366)	261,495 (374.5)	389,580 (586)	164,001 (225)	382 (0.1%)	7,537 (1.7%)	101 (<0.1%)	1,525 (0.6%)	3,024 (0.3%)	47,636 (5.5%)	533 (0.2%)	5,607 (2.1%)	285 (0.1%)	3,893 (1.0%)	152 (0.1%)	3,300 (2.0%)
L	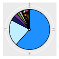	325,436 (2,949.5)	156,633 (1,231.5)	527,812 (5,137.5)	298,610 (2,485)	658,236 (4,956)	1,062,172 (6,769)	250 (0.1%)	6,188 (1.9%)	49 (<0.1%)	901 (0.6%)	967 (0.2%)	27,291 (5.2%)	1,276 (0.4%)	6,548 (2.2%)	7,568 (1.1%)	15,551 (2.4%)	38,399 (3.6%)	61,856 (5.8%)
M	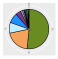	282,907 (946.5)	124,667 (364)	523,378 (1,918.5)	224,557 (870.5)	365,855 (1,289.5)	324,489 (906)	373 (0.1%)	6,596 (2.3%)	949 (0.8%)	1,768 (1.4%)	2,668 (0.5%)	26,925 (5.1%)	928 (0.4%)	5,710 (2.5%)	1,198 (0.3%)	6,056 (1.7%)	956 (0.3%)	12,618 (3.9%)
N	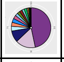	394,726 (845)	151,789 (337)	951,440 (2,225)	205,215 (515)	303,620 (740)	147,028 (291)	532 (0.1%)	8,419 (2.1%)	93 (0.1%)	1,211 (0.8%)	4,492 (0.5%)	65,138 (6.8%)	1,159 (0.6%)	7,401 (3.6%)	326 (0.1%)	4,294 (1.4%)	301 (0.2%)	5,658 (3.8%)
O	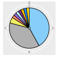	11,824 (45)	6,247 (11)	26,094 (71)	9,400 (24)	18,691 (31)	7,827 (15)	136 (1.2%)	413 (3.5%)	118 (1.9%)	276 (4.4%)	403 (1.5%)	2,157 (8.3%)	239 (2.5%)	597 (6.4%)	526 (2.8%)	1,074 (5.7%)	238 (3.0%)	442 (5.6%)
P	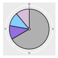	287 (18)	91 (6)	758 (56)	211 (15)	291 (23)	115 (8)	0 (0%)	5 (1.7%)	0 (0%)	2 (2.2%)	0 (0%)	58 (7.7%)	0 (0%)	6 (2.8%)	0 (0%)	14 (4.8%)	0 (0%)	3 (2.6%)
overall		7,959,694 (941)	2,588,475 (473)	18,121,448 (1,809)	3,473,029 (613)	6,393,997 (871)	4,871,710 (385)	-	141,747 (1.8%)	-	8,856 (0.3%)	-	758,411 (4.2%)	-	25,167 (0.7%)	-	38,336 (0.6%)	-	85,418 (1.75%)

The number of SSMs refers to the number all samples of a cluster have combined, counting recurrent SSMs once. The number (and percentage) of recurrent SSMs is computed based on only the samples from the particular cluster (within cluster) and on the entire cohort. In dark red is indicated per SSM subtype the cluster with the lowest median number of SSMs or the lowest percentage of recurrence for 'within cluster' and 'entire cohort'. In light red the second lowest is indicated. The highest and second highest are indicated in dark and light green, respectively.

Table B. Cluster-specific overview of total and recurrent 1 bp SIMs per subtype.

Cluster		Total number (median)				Number recurrent (percentage)							
		1 bp A/T del.	1 bp C/G del.	1 bp A/T ins.	1 bp C/G ins.	1 bp A/T deletion		1 bp C/G deletion		1 bp A/T insertion		1 bp C/G insertion	
						within cluster	entire cohort	within cluster	entire cohort	within cluster	entire cohort	within cluster	entire cohort
A	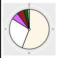	21,751 (307)	50,154 (659)	24,171 (336.5)	4,147 (55)	5 (<0.1%)	1,541 (7.1%)	21 (<0.1%)	317 (0.6%)	31 (0.1%)	2,082 (8.6%)	0 (0%)	102 (2.5%)
B	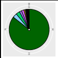	39,653 (119.5)	37,960 (111.5)	52,075 (151.5)	4,797 (14)	44 (0.1%)	2,145 (5.4%)	31 (0.1%)	254 (0.7%)	461 (0.9%)	7,991 (15.3%)	21 (0.4%)	313 (6.5%)
C	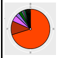	40,632 (185)	18,128 (88)	15,411 (74)	2,387 (11)	6 (<0.1%)	644 (1.6%)	4 (<0.1%)	65 (0.4%)	9 (0.1%)	637 (4.1%)	4 (0.2%)	32 (1.3%)
D	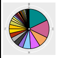	34,652 (47)	22,492 (30)	24,758 (34.5)	3,455 (4)	81 (0.2%)	3,663 (10.6%)	27 (0.1%)	236 (1.0%)	111 (0.4%)	4,025 (16.3%)	13 (0.4%)	177 (5.1%)
E	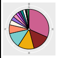	8,825 (65)	5,922 (44)	11,037 (96.5)	1,164 (10)	18 (0.2%)	1,833 (20.8%)	5 (0.1%)	100 (1.7%)	23 (0.2%)	2,052 (18.6%)	1 (0.1%)	119 (10.2%)
F	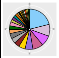	3,966 (30)	2,125 (19)	4,860 (35)	405 (4)	4 (0.1%)	384 (9.7%)	2 (0.1%)	40 (1.9%)	4 (0.1%)	858 (17.7%)	5 (1.2%)	139 (34.3%)
G	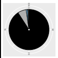	26,260 (227)	8,224 (70)	11,473 (104)	2,457 (20)	26 (0.1%)	1,010 (3.8%)	34 (0.4%)	112 (1.4%)	13 (0.1%)	1,217 (10.6%)	5 (0.2%)	111 (4.5%)
H	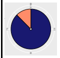	30,262 (2,059)	2,994 (232)	50,704 (6,078)	4,119 (406.5)	211 (0.7%)	6,044 (20.0%)	11 (0.4%)	119 (4.0%)	348 (0.7%)	6,122 (12.1%)	5 (0.1%)	84 (2.0%)
I	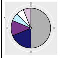	997 (1)	252 (0.5)	4,887 (1.5)	3,744 (1)	0 (0%)	280 (28.1%)	0 (0%)	8 (3.2%)	0 (0%)	389 (8.0%)	0 (0%)	18 (0.5%)
J	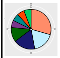	431,323 (19,035)	43,387 (1,362)	172,855 (9,367)	19,070 (1,245)	74,137 (17.2%)	107,563 (24.9%)	2,588 (6.0%)	3,783 (8.7%)	7,733 (4.5%)	29,421 (17.0%)	837 (4.4%)	2,131 (11.2%)
K	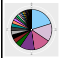	34,767 (53)	17,859 (27)	59,677 (92)	5,097 (8)	220 (0.6%)	5,883 (16.9%)	35 (0.2%)	370 (2.1%)	837 (1.4%)	11,017 (18.5%)	46 (0.9%)	517 (10.1%)
L	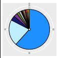	58,340 (522.5)	8,270 (77.5)	44,788 (434)	10,421 (72.5)	2,409 (4.1%)	19,939 (34.2%)	50 (0.6%)	399 (4.8%)	556 (1.2%)	9,069 (20.2%)	46 (0.4%)	539 (5.2%)
M	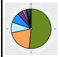	51,175 (156.5)	8,702 (30)	47,209 (119)	4,167 (10.5)	1,710 (3.3%)	17,947 (35.1%)	24 (0.3%)	350 (4.0%)	567 (1.2%)	8,933 (18.9%)	41 (1.0%)	495 (11.9%)
N	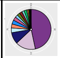	72,981 (129)	13,914 (35)	96,438 (236)	7,323 (16.0)	3,421 (4.7%)	26,216 (35.9%)	102 (0.7%)	854 (6.1%)	2,744 (2.8%)	19,724 (20.5%)	165 (2.3%)	1,010 (13.8%)
O	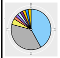	916 (2)	477 (2)	1,309 (2)	132 (0)	6 (0.7%)	93 (10.2%)	1 (0.2%)	20 (4.2%)	10 (0.8%)	275 (21.0%)	2 (1.5%)	25 (18.9%)
P	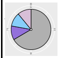	26 (3)	13 (1)	23 (2)	3 (0)	0 (0%)	7 (26.9%)	0 (0%)	11 (84.6%)	0 (0%)	6 (26.1%)	0 (0%)	0 (0%)
overall		780,494 (88)	238,272 (40)	570,960 (97)	70,067 (10)	-	119,160 (15.3%)	-	4,437 (1.9%)	-	53,103 (9.3%)	-	2,991 (4.3%)

The number of 1 bp SIMs refers to the number all samples of a cluster have combined, counting recurrent 1 bp SIMs once. The number (and percentage) of recurrent 1 bp SIMs is computed based on only the samples from the particular cluster (within cluster) and on the entire cohort. In dark red is indicated per SIM subtype the cluster with the lowest median number of 1 bp SIMs or the lowest percentage of recurrence for 'within cluster' and 'entire cohort'. In light red the second lowest is indicated. The highest and second highest are indicated in dark and light green, respectively.

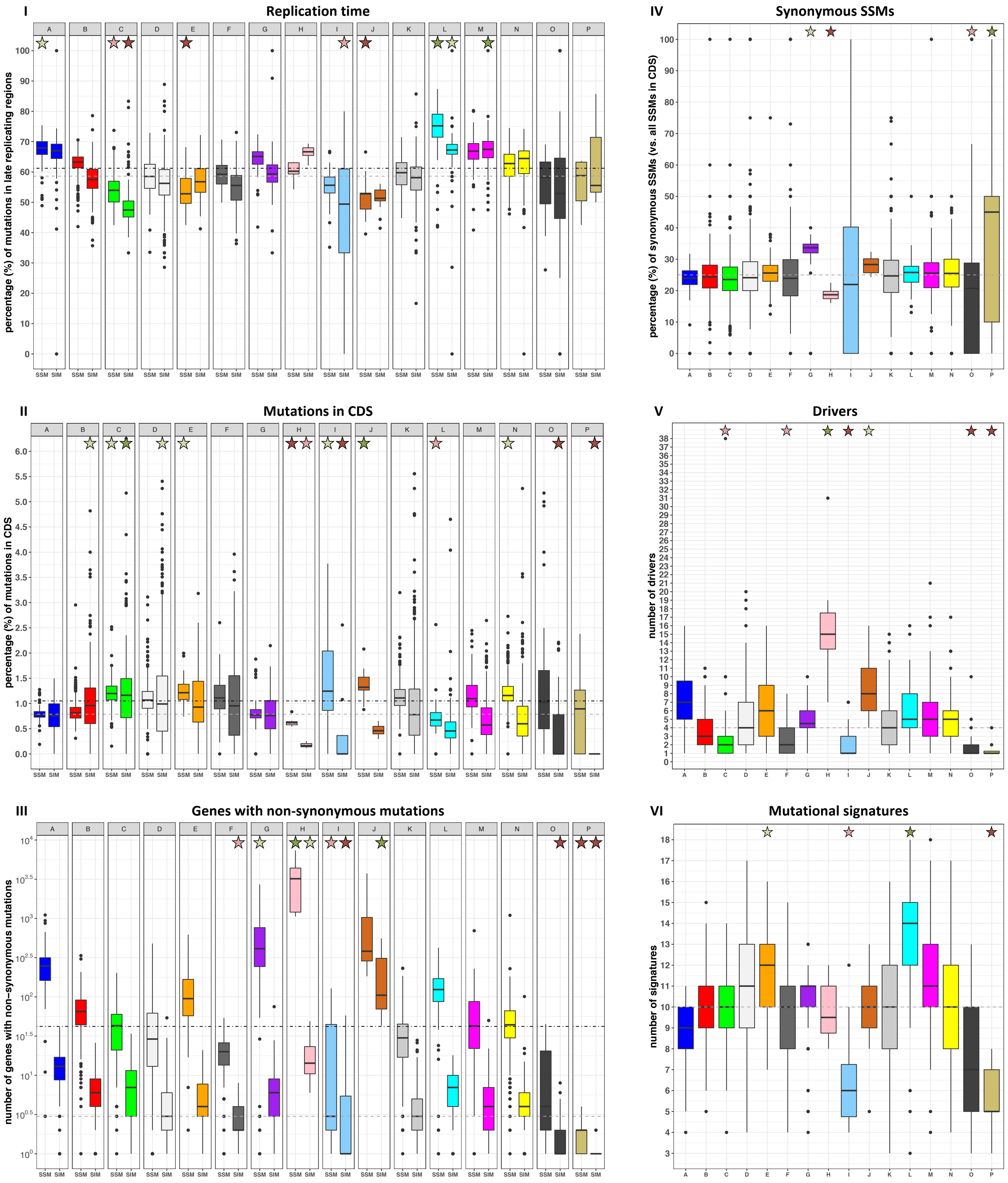

Fig A. Overview of annotation layers laid on top of each cluster.

The boxplots represent for each cluster: (I) percentage of SSMs/SIMs in late-replicating regions; (II) percentage of SSMs/SIMs in coding sequence (CDS); (III) number of genes with non-synonymous SSMs/SIMs; (IV) percentage of SSMs in CDS that are synonymous (excluding samples without any SSMs in CDS); (V) number of predicted drivers (excluding samples for which no drivers were predicted); (VI) number of mutational signatures. For the three annotation layers specifically related to SIMs, two samples were left out as no SIMs were detected. Plot II shows only percentages up to ~6% for better visualization. The values for 17 samples are, therefore, not shown. Numbers of genes are increased by one in plot III to be able to visualize them on a log-scale. The black and grey dashed lines indicate the overall median for SSMs and SIMs, respectively. The dark green stars at the top of each of the plots indicate the clusters with the highest median and the light green stars the second highest. The dark red ones indicate the lowest median and the light red ones the second lowest.

Table C. Overview of the median values across samples for the various layers of annotation.

Cluster		MEDIAN									
		Perc. in late-replicating regions (ratio high vs. early)		Percentage in CDS		Number of genes mutated		Perc. of synonymous SSMs from total number in CDS	Number of predicted drivers	Number of mutational signatures
		SSMs	SIMs	SSMs	SIMs	SSMs	SIMs			
A	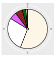	67.9% (2.1x)	67.0% (2.0x)	0.8%	0.8%	246,5	12	23.9%	7	9
B	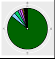	63.3% (1.7x)	57.5% (1.4x)	0.8%	1.0%	64	5	24.4%	3	10
C	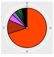	53.9% (1.2x)	47.5% (0.9x)	1.2%	1.2%	42	6	23.5%	2	10
D	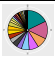	58.5% (1.4x)	56.2% (1.3x)	1.1%	1.0%	28	2	24.1%	4	11
E	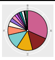	52.8% (1.1x)	56.8% (1.3x)	1.2%	0.9%	94	3	25.6%	6	12
F	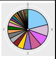	59.2% (1.5x)	55.6% (1.2x)	1.1%	1.0%	19	1	23.9%	2	10
G	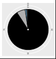	65.1% (1.9x)	59.3% (1.5x)	0.8%	0.8%	410	5	33.6%	4.5	11
H	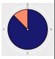	60.2% (1.5x)	66.7% (2.0x)	0.6%	0.2%	3,223	13.5	18.7%	15	9.5
I	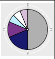	55.6% (1.3x)	49.4% (1.0x)	1.2%	0%	2	0	22.0%	1	6
J	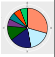	52.8% (1.1x)	51.3% (1.1x)	1.3%	0.5%	381	104	28.3%	8	10
K	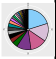	59.8% (1.5x)	58.1% (1.4x)	1.1%	0.8%	29	2	24.7%	4	10
L	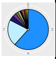	75.2% (3.0x)	67.2% (2.1x)	0.7%	0.5%	123	6	25.8%	5	14
M	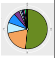	66.9% (2.0x)	67.5% (2.1x)	1.1%	0.6%	41,5	3	25.6%	5	11
N	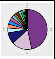	62.8% (1.7x)	64.4% (1.8x)	1.2%	0.6%	43	3	25.5%	5	10
O	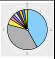	58.2% (1.4x)	52.9% (1.1x)	1.0%	0%	3	0	20.7%	1	7
P	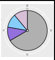	58.8% (1.4x)	55.6% (1.2x)	0.9%	0%	1	0	45.0%	1	5
overall		61.2% (1.6x)	58.6% (1.4x)	1.1%	0.8%	42	3	25.0%	4	10

For each annotation the dark and light green boxes indicate the top- and second ranking cluster, respectively. Dark and light red indicate the lowest and second lowest cluster, respectively. For the three medians related to SIMs, two samples without any SIMs are left out. For the median percentage of synonymous SSMs (versus all in CDS), samples without any SSMs in CDS are not included. For the median number of predicted drivers, samples without any driver predicted are excluded.

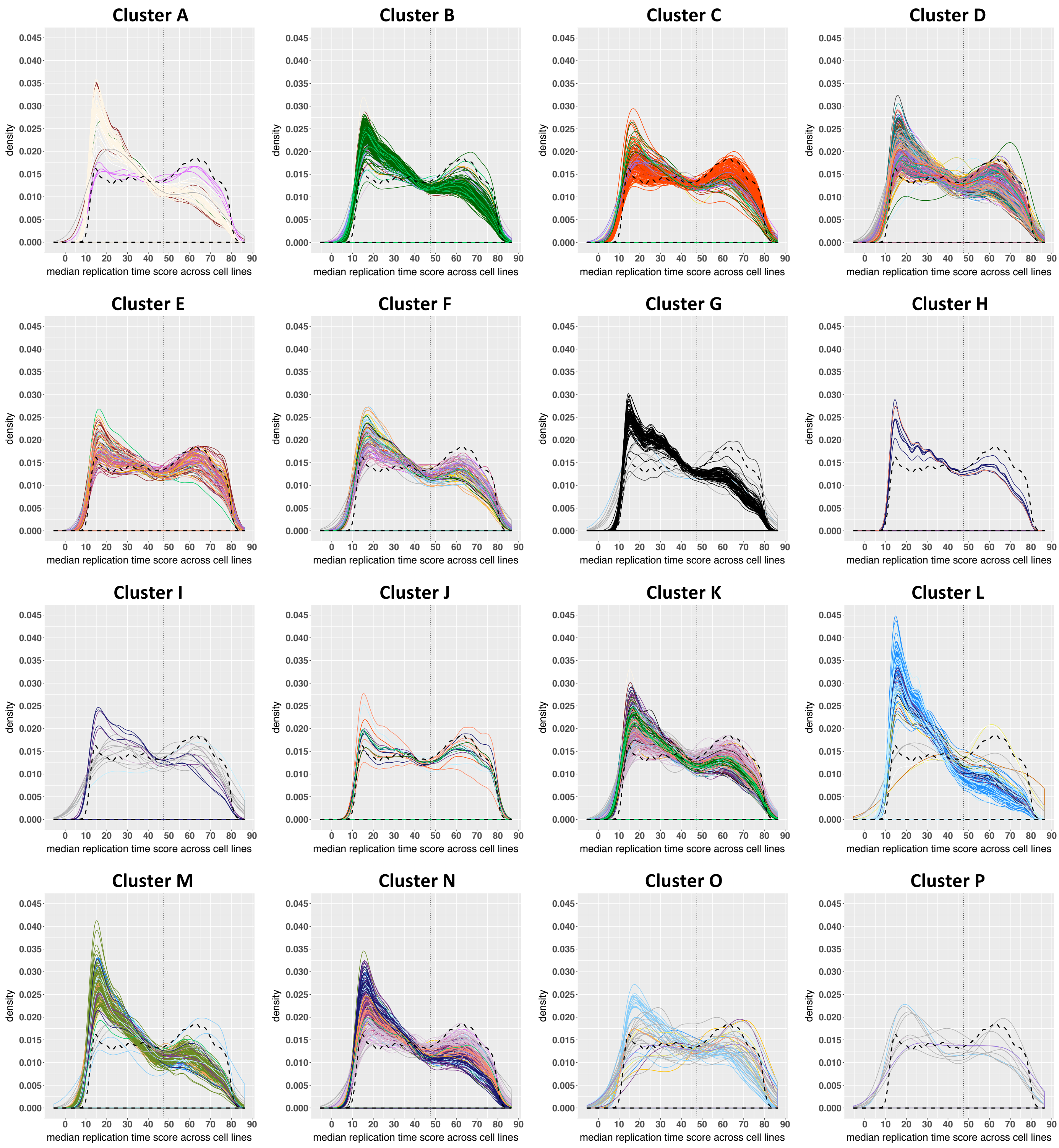

Fig B. The clusters differ in terms of their distribution of SSMs in early- versus late-replicating regions.

We collected for each SSM the corresponding replication time score except for those in chromosome Y or chromosomal end regions. Lower replication time scores refer to later replicating regions and higher scores to earlier replicating regions. The density plots per cluster show the distribution of the collected replication time scores, where each line indicates a cancer genome and is coloured according to the tumour type. Cancer genomes with less than 10 SSMs were left out. The dashed line indicates the density plot of the median replication time scores of the five cancer cell lines from the ENCODE project. The vertical line indicates the median of these scores and is used to separate early from late-replicating regions.

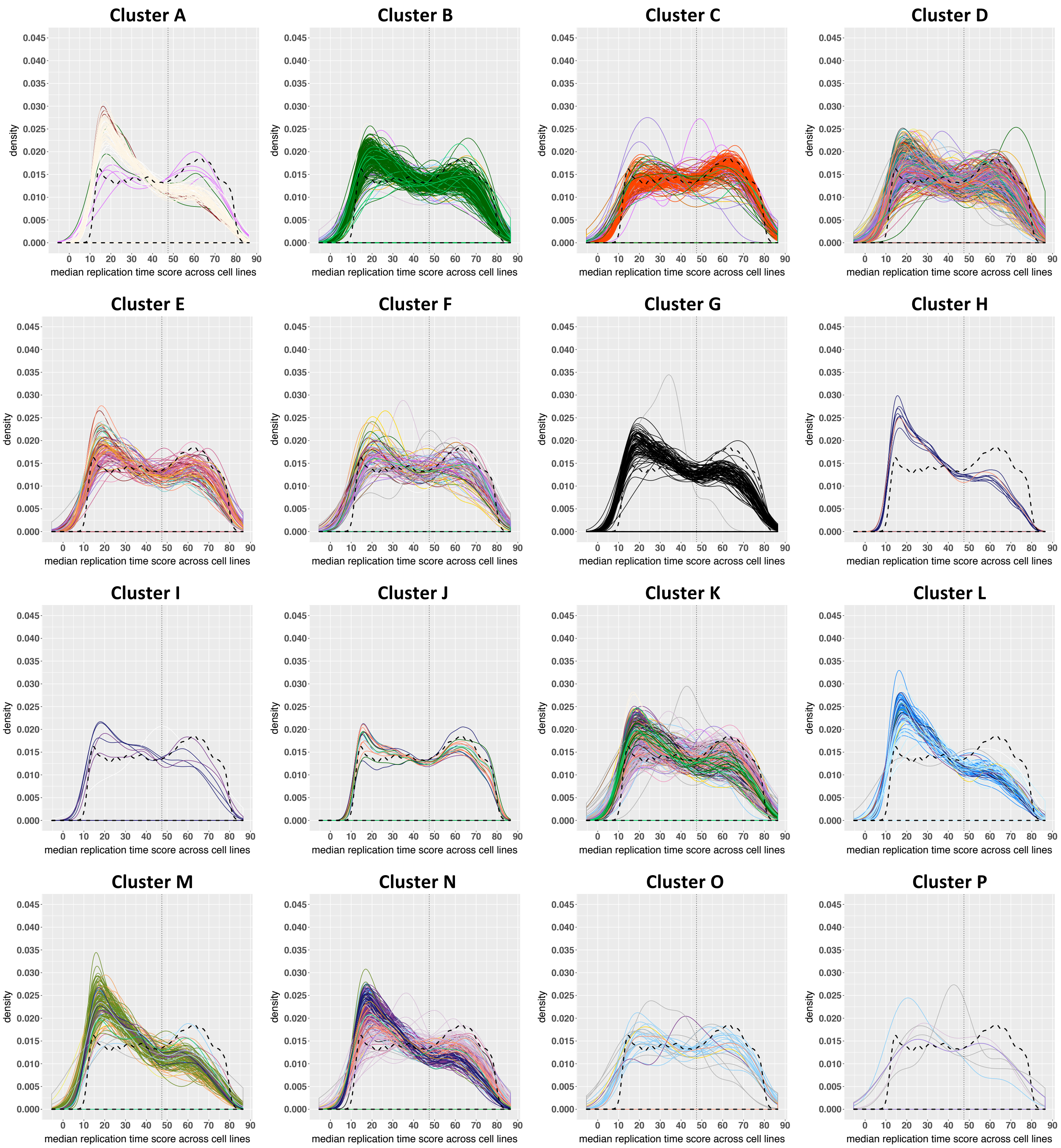

Fig C. The clusters differ in terms of their distribution of SIMs in early- versus late-replicating regions.

We collected for each SIM the corresponding replication time score except for those in chromosome Y or chromosomal end regions. Lower replication time scores refer to later replicating regions and higher scores to earlier replicating regions. The density plots per cluster show the distribution of the collected replication time scores, where each line indicates a cancer genome and is coloured according to the tumour type. Cancer genomes with less than 10 SIMs were left out. The dashed line indicates the density plot of the median replication time scores of the five cancer cell lines from the ENCODE project. The vertical line indicates the median of these scores and is used to separate early from late-replicating regions.

Table D. Top 3 predicted drivers per cluster.

Cluster			Top 3 predicted drivers		
A	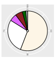	driver	*TP53*	*CDKN2A*	*NOTCH1*
		perc. of samples	73.5%	35.3%	22.1%
B	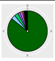	driver	*TP53*	*CTNNB1*	*ARID1A*
		perc. of samples	31.2%	23.5%	17.6%
C	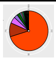	driver	*VHL*	*PBRM1*	*SETD2*
		perc. of samples	42.6%	28.2%	11.3%
D	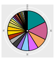	driver	*TP53*	*PTEN*	*19p13.3a*
		perc. of samples	40%	12.4%	12.2%
E	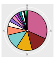	driver	*TP53*	*PIK3CA*	*CDKN2A*, *TERT*
		perc. of samples	52%	24.5%	18.4%
F	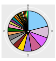	driver	*TP53*	*PTEN*	*ERG*
		perc. of samples	23.2%	16.8%	12.6%
G	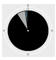	driver	*TERT*	*BRAF*	*CDKN2A*
		perc. of samples	69%	56.3%	50.6%
H	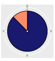	driver	*PIK3CA*, *POLE*	*APC*, *KRAS*	*ATM*, *CTNNB1*
		perc. of samples	100%	87.5%	62.5%
I	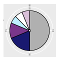	driver	*BRAF*	*TP53*	*APC*
		perc. of samples	50%	25%	18.8%
J	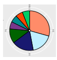	driver	*ARID1A*, *RPL22*	*ACVR2A*	*PIK3CA*, *PTEN*, *TP53*
		perc. of samples	47.1%	41.2%	35.3%
K	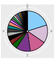	driver	*TP53*	*CDKN2A*	*PTEN*
		perc. of samples	33.7%	15.7%	13%
L	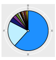	driver	*TP53*	*CDKN2A*	*ARID1A*, *SMAD4*
		perc. of samples	75%	31.7%	18.3%
M	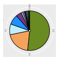	driver	*TP53*	*BCL2*	*CREBBP*
		perc. of samples	29.3%	23.4%	19%
N	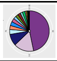	driver	*TP53*	*KRAS*	*CDKN2A*
		perc. of samples	56.3%	51.8%	43.7%
O	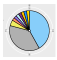	driver	*BRAF*	ERG	*CDKN1B*
		perc. of samples	32.6%	18.6%	11.6%
P	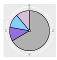	driver	*BRAF*	*ACO1*, *APTX*, *FANCG*, *LDB1*, *POLE*, *PTCH1*	
		perc. of samples	66.7%	11.1%	
overall		driver	*TP53*	*CDKN2A*	*ARID1A*
		perc. of samples	36.9%	18.4%	12.2%

The top 3 are based on the percentage of samples in a cluster in which the gene or locus is considered a driver. Drivers that are found in the top 3 of multiple clusters are coloured.

Table E. Top 3 mutational signatures of type SBS, DBS and ID per cluster.

Cluster			Top 3 SBS signatures			Top 3 DBS signatures			Top 3 ID signatures		
A	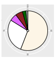	signature	SBS5	SBS4	SBS1	DBS2	DBS11	DBS6	ID3	ID1	ID2
		perc. of samples	94.1%	89.7%	72.1%	92.6%	7.4%	7.4%	97.1%	86.8%	55.9%
		median contribution	41.7%	48.0%	0.6%	100%	45.8%	30.6%	71.9%	11.2%	8.0%
B	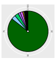	signature	SBS5	SBS1	SBS12	DBS2	DBS4	DBS5	ID1	ID5	ID3
		perc. of samples	100%	67.6%	59.3%	96.9%	67.0%	8.3%	99.7%	96.0%	94.4%
		median contribution	66.9%	1.0%	19.4%	61.0%	41.2%	23.7%	21.0%	32.6%	25.4%
C	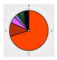	signature	SBS1	SBS5	SBS40	DBS2	DBS4	DBS9	ID8	ID5	ID9
		perc. of samples	99.5%	94.4%	87.7%	88.7%	68.2%	56.9%	96.4%	95.9%	60.0%
		median contribution	5.4%	17.5%	74.8%	20.8%	23.9%	62.2%	16.0%	60.3%	10.7%
D	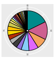	signature	SBS1	SBS5	SBS40	DBS2	DBS4	DBS6	ID1	ID9	ID8
		perc. of samples	99.8%	97.2%	46.6%	68.3%	63.5%	37.1%	91.4%	70.9%	69.1%
		median contribution	8.7%	32.8%	47.0%	26.1%	29.7%	30.0%	13.6%	20.7%	21.6%
E	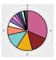	signature	SBS5	SBS1	SBS13	DBS11	DBS2	DBS4	ID1	ID2	ID8
		perc. of samples	100%	100%	99.0%	75.5%	70.4%	62.2%	100%	79.6%	70.4%
		median contribution	28.3%	4.9%	28.7%	50.0%	15.5%	23.8%	24.6%	12.4%	21.4%
F	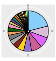	signature	SBS5	SBS1	SBS40	DBS2	DBS4	DBS11	ID1	ID2	ID8
		perc. of samples	100%	100%	41.1%	55.8%	47.4%	28.4%	96.8%	70.5%	66.3%
		median contribution	51.2%	12.6%	52.3%	35.3%	41.7%	46.2%	26.1%	9.0%	16.5%
G	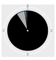	signature	SBS7a	SBS7b	SBS7d	DBS1	DBS5	DBS9	ID1	ID13	ID8
		perc. of samples	90.8%	90.8%	90.8%	92.0%	1.1%	1.1%	93.1%	87.4%	87.4%
		median contribution	68.4%	19.3%	3.1%	100%	100%	100%	10.7%	31.6%	22.4%
H	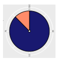	signature	SBS10a	SBS28	SBS10b	DBS3	DBS10	DBS4	ID1	ID2	ID5
		perc. of samples	100%	100%	100%	87.5%	50.0%	37.5%	100%	100%	12.5%
		median contribution	46.8%	20.1%	13.3%	67.7%	44.6%	9.2%	69.8%	25.7%	30.4%
I	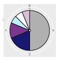	signature	SBS1	SBS5	SBS40	DBS7	DBS4	DBS2	ID2	ID1	ID14
		perc. of samples	100%	81.2%	56.2%	31.2%	31.2%	25.0%	56.2%	50.0%	18.8%
		median contribution	23.0%	52.3%	57.2%	86.2%	6.9%	10.9%	21.5%	17.4%	72.0%
J	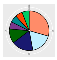	signature	SBS5	SBS1	SBS44	DBS9	DBS2	DBS7	ID2	ID1	-
		perc. of samples	100%	100%	52.9%	76.5%	76.5%	64.7%	100%	94.1%	-
		median contribution	33.3%	5.6%	40.0%	35.7%	9.5%	38.8%	73.6%	26.5%	-
K	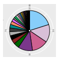	signature	SBS1	SBS5	SBS40	DBS2	DBS4	DBS6	ID1	ID2	ID8
		perc. of samples	100%	99.6%	47.3%	66.1%	59.4%	33.3%	99.8%	78.9%	57.1%
		median contribution	15.0%	38.4%	50.1%	26.1%	31.8%	29.1%	34.9%	11.1%	17.6%
L	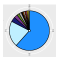	signature	SBS1	SBS17b	SBS17a	DBS4	DBS2	DBS6	ID2	ID1	ID5
		perc. of samples	99.0%	95.2%	95.2%	90.4%	89.4%	65.4%	98.1%	96.2%	53.8%
		median contribution	6.4%	23.7%	12.1%	19.9%	13.1%	14.2%	31.8%	30.1%	32.8%
M	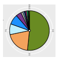	signature	SBS1	SBS5	SBS40	DBS4	DBS9	DBS11	ID2	ID1	ID5
		perc. of samples	100%	99.5%	64.1%	73.4%	68.5%	66.8%	99.5%	98.9%	32.1%
		median contribution	8.5%	33.6%	42.5%	15.4%	36.2%	23.5%	36.8%	36.8%	36.5%
N	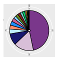	signature	SBS5	SBS1	SBS40	DBS4	DBS2	DBS9	ID1	ID2	ID8
		perc. of samples	100%	99.7%	52.7%	78.1%	65.6%	57.6%	100%	93.9%	22.8%
		median contribution	29.6%	20.8%	44.9%	25.0%	20.6%	36.4%	52.6%	28.7%	25.0%
O	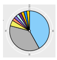	signature	SBS5	SBS1	SBS40	DBS2	DBS9	DBS4	ID1	ID5	ID2
		perc. of samples	100%	100%	53.5%	32.6%	23.3%	14.0%	62.8%	55.8%	51.2%
		median contribution	42.4%	16.3%	43.5%	18.8%	71.3%	38.8%	24.2%	34.7%	7.7%
P	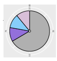	signature	SBS5	SBS1	SBS40	DBS2	DBS9	-	ID1	ID5	ID2
		perc. of samples	100%	100%	55.6%	11.1%	11.1%	-	77.8%	66.7%	66.7%
		median contribution	39.0%	25.7%	47.7%	100%	100%	-	25.0%	65.2%	10.8%
overall		signature	SBS5	SBS1	SBS40	DBS2	DBS4	DBS9	ID1	ID2	ID8
		perc. of samples	97.8%	93.7%	43.9%	69.3%	61.4%	34.3%	93.1%	69.0%	56.8%
		median contribution	35.6%	9.7%	48.6%	27.0%	28.6%	41.2%	25.1%	13.4%	17.5%

The top 3 are based on the percentage of samples in which the signature is present. The median contribution of the signatures is computed based on only those samples in which the signature is found. If there are multiple signatures present in the same percentage of samples, the one with the highest median contribution is selected. Signatures that are found in the top 3 of multiple clusters are coloured.

Proposed aetiology of the SBS signatures [1]:
SBS1: spontaneous or enzymatic deamination of 5-methylcytosine to thymine;
SBS4: tobacco-smoke exposure;
SBS7a/b/d: UV-light exposure;
SBS10a/b: polymerase ε exonuclease domain mutations;
SBS13: activity of the AID/APOBEC family of cytidine deaminases;
SBS44: defective DNA MMR;
SBS5, SBS12, SBS17a, SBS17b, SBS28, SBS40: unknown.

Proposed aetiology of the DBS signatures [1]:
DBS1: UV-light exposure;
DBS2: tobacco-smoke exposure and other endogenous and/or exogenous mutagens;
DBS3: polymerase ε exonuclease domain mutations;
DBS5: prior chemotherapy treatment with platinum drugs;
DBS7, DBS10: defective DNA MMR;
DBS11: possibly related to activity of the AID/APOBEC family of cytidine deaminases
DBS4, DBS6, DBS9: unknown.

Proposed aetiology of the ID signatures [1]:
ID1, ID2: slippage during DNA replication of the replicated DNA strand;
ID3: tobacco-smoke exposure;
ID8: repair of DNA double strand breaks by non-homologous DNA end-joining mechanisms;
ID13: UV-light exposure;
ID5, ID9, ID14: unknown.

Cluster A – high percentage of C>A SSMs and 1 bp C/G deletions

Cluster A is dominated by samples from the two subtypes of lung cancer, Lung-AdenoCA and Lung-SCC. Together they make up nearly 84% of the 68 samples in this cluster. The two key features that are positively associated are the percentage of C>A SSMs and 1 bp C/G deletions (Fig D). For close to 80% of the 58 samples in this cluster with collected smoking history, the donor was either a current smoker at diagnosis or had quit less than 15 years before. C>A transversions have been linked to the effects of tobacco smoke before, suggesting a preferential incorporation of dA opposite some of the tobacco-related guanine adducts [10]. The 1 bp C/G deletions have not yet been associated with smoking related DNA damage. Interestingly, we do not observe the same association between smoking and the high percentage of C>A SSMs and 1 bp C/G deletions as in the two lung cancer subtypes for ten other tumour types for which information on smoking history of a sufficient number of the respective donors is available (Table A in S4 Text). Only three samples of Head-SCC reach similar percentages for both mutation types as the lung cancer samples (S4 Text). These donors also smoked at the time of diagnosis or had quit less than 15 years before. This supports the idea that direct tobacco-smoke exposure is essential for the 1 bp C/G deletions as well as the C>A SSMs. Several mechanisms for the deletions are conceivable including that the incoming dNTP pairs to the next template base and is stabilized by stacking interactions with the guanine adduct [10]. Alternatively, if base excision repair removes the modified guanine, the abasic site in the template strand could loop out in a way that a 1 bp deletion manifests in the newly synthesized strand [11]. Another interesting observation for this cluster is that it has the highest median number of C>G SSMs of all clusters (Table A), which is the least common SSM subtype overall. Cytosine deamination followed by base excision repair results in abasic sites, which combined with the activity of the REV1 polymerase has been suggested as one possible explanation for this SSM subtype [12]. Finally, there is a significant negative association with all features reflecting recurrence. The cluster ranks in the bottom two in terms of relative recurrence (across the cohort) for four SSM subtypes and two SIM subtypes (Table A and B) despite having the second highest median number for two of these four SSM subtypes (C>A and T>A, Table A) and one of the two SIM subtypes (1 bp C/G deletions, Table B).

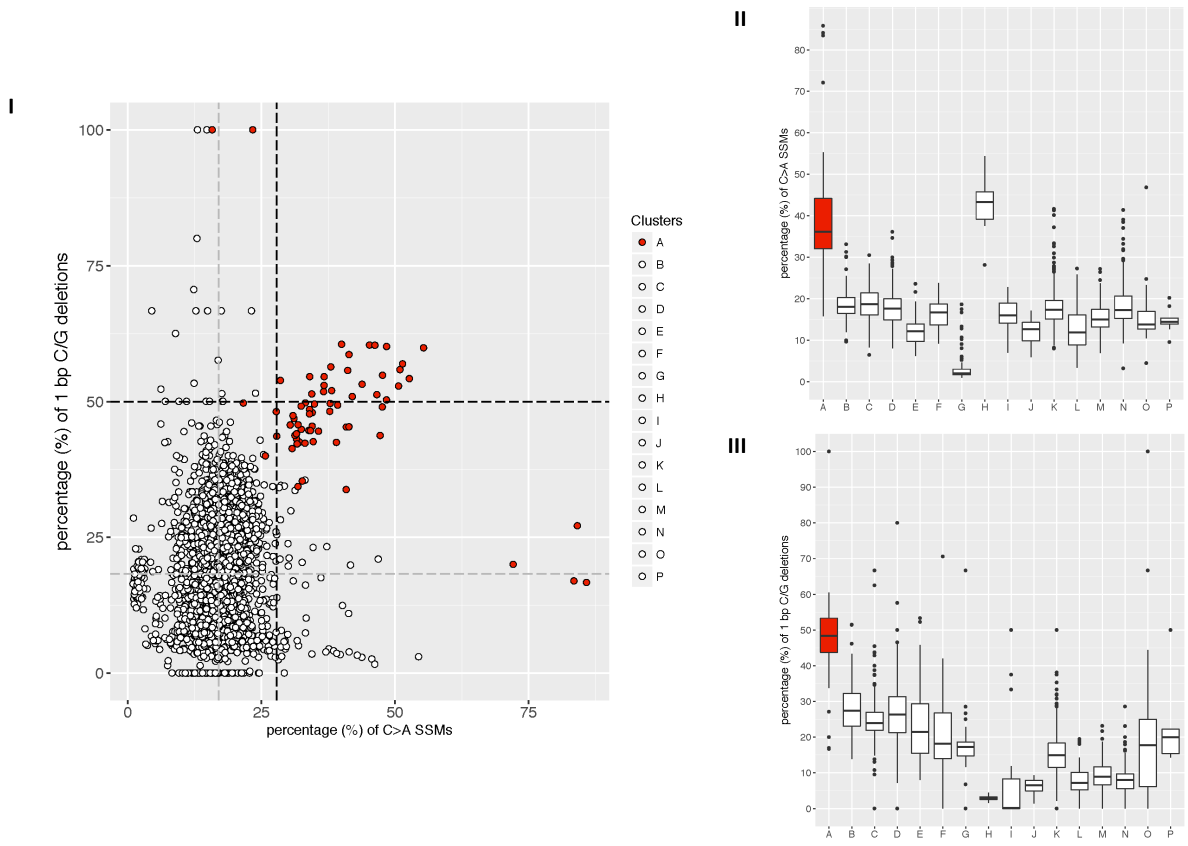

Fig D. Key characteristics of cluster A.

For cluster A the two features with the strongest association are the <u>percentage of C>A SSMs</u> and <u>1 bp C/G deletions</u>. (I) Percentage of C>A SSMs versus 1 bp C/G deletions. The grey lines indicate the medians and the black lines indicate the third quartiles plus 1.5 times the interquartile range (Q3+1.5xIQR), above which samples are outliers. (II) Boxplots of the percentage of C>A SSMs for each cluster. (III) Boxplots of the percentage of 1 bp C/G deletions for each cluster.

Annotation

There are 2.1 times more SSMs (median across the cluster) in late-replicating regions compared to early, which is the second highest ratio of all clusters. The most frequently predicted driver in cluster A is the tumour suppressor gene *TP53* (73.5% of samples). This gene also ranks first in eight other clusters, but is only in one other cluster as often predicted a driver as in cluster A. *CDKN2A*, encoding an essential tumour suppressor, ranks second and is predicted to be a driver in 35.3% of the samples. Third is *NOTCH1*, a gene encoding a transmembrane protein relevant for interactions among adjacent cells (22.1% of samples). Another interesting observation is that the *RYR2*, although not considered a driver in any sample, has non-synonymous mutations in as many as 39.7% of the samples. This gene is involved in calcium signalling and has been found with elevated mutation frequency in air pollution-related lung cancer [13].

In line with the clinical data on smoking history, the 'tobacco smoking' signature based on single base substitutions (SBS4) is present in 89.7% of the samples and explains

nearly half of the SBSs in these samples. For ~84% of the samples signature DBS2 explains all doublet base substitutions. This signature is potentially linked to exposure to tobacco smoking as well, but also to other exogenous and endogenous mutagens [1]. Finally, tobacco-smoke exposure was also proposed to underlie the main ID signature in this cluster (ID3, median contribution: 71.9%).

Cluster B – high percentage of T>C SSMs

This cluster contains 324 samples of which just over 85% are Liver-HCC, which account for ~88% of the total number of samples of this tumour type. The strongest positively associated feature is the percentage of T>C transitions (Fig E). A recent study of liver cancer with a high number of samples in common with the PCAWG cohort, uncovered two mutational signatures with high levels of T>C SSMs [14]. While both signatures were linked to mutations in the *TERT* promoter, one was associated with alcohol intake and the other with age at cancer diagnosis. Based on whole-exome sequencing data of liver cancer, Totoki *et al.* earlier suggested that the high percentage of T>C SSMs is more prevalent in Japanese males than in donors from the USA and that transcription-coupled repair pathways are crucial for the formation of this mutation type [15]. In line with this, ~93% of Liver-HCC samples of male donors originating from the two Japanese studies are in cluster B versus ~81% of female Japanese donors and donors from studies not originating from Japan combined (Fisher Exact Test, p=0.0022). Within cluster B the male Japanese donors have a higher percentage of T>C SSMs than the other donors (34.3% vs. 28.3%, Wilcoxon rank-sum test, p=1.9e-10). With respect to a possible mechanism, in liver cells from individuals with diseases related to alcohol abuse increased levels of 1,N^6-ethenodeoxyadenosine DNA adducts were detected [16]. This type of adduct is likely a product of alcohol-induced oxidative stress and lipid peroxidation [16] and could lead to T>C transitions. Alcohol can also induce error-prone repair involving polymerase η, which leads to elevated levels of T>C transitions as well [17].

There is significant negative association with features capturing recurrence. Intuitively, one would expect the contrary given the large number of samples from the same tumour type and the fact that this cluster contains the highest total number of T>C SSMs (1,238,809). However, only 0.1% of the T>C SSMs are recurrent within the cluster (Table A). In fact, the median number of T>C SSMs per sample (3,577) is not as high and puts this cluster in sixth position, which could play a role in the lack of recurrence.

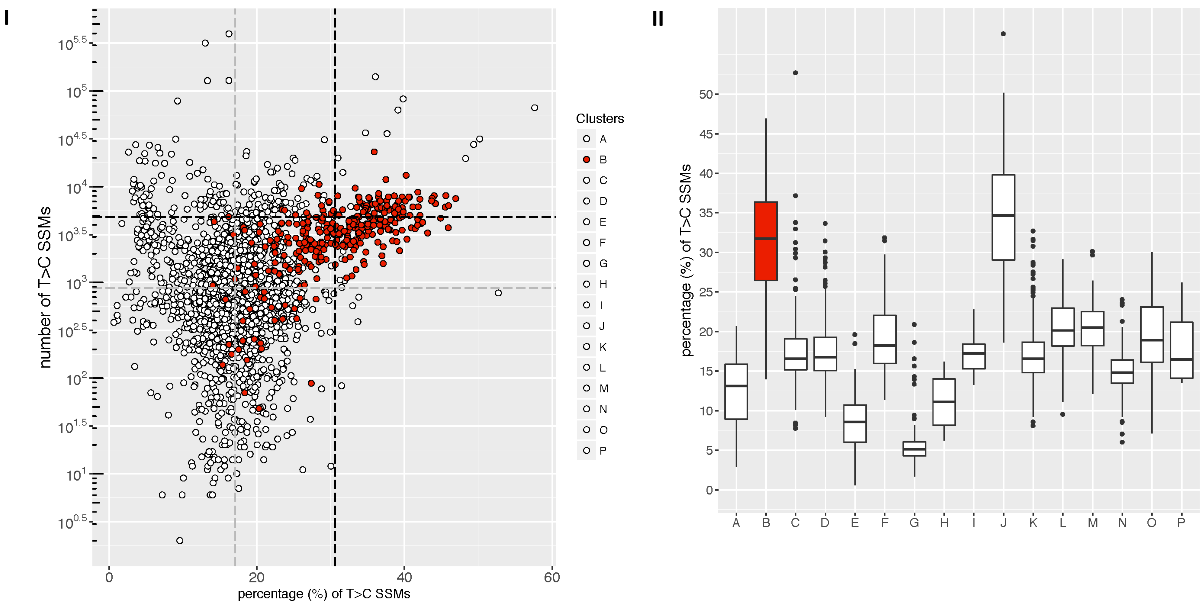

Fig E. Key characteristic of cluster B.

For cluster B the feature with the strongest association is the percentage of T>C SSMs. (I) Percentage versus absolute number of T>C SSMs. The grey lines indicate the medians and the black lines indicate Q3+1.5xIQR, above which samples are outliers. (II) Boxplots of the percentages of T>C SSMs for each cluster.

Annotation

Around 1% of SIMs (median across samples) are in CDS, which is a higher proportion than in any other cluster with the exception of cluster C and D. *TP53* is the most frequently predicted driver (31.2% of samples), followed by the gene encoding the cell adhesion factor *CTNNB1* (23.5%), which has been related to alcohol-related liver cancer before [18]. Ranking third is *ARID1A* (17.6%), a ubiquitously expressed tumour suppressor gene exerting its function by contributing to chromatin remodelling and transcriptional activation. In terms of mutational signatures, SBS5 stands out as it is present in all samples with a median contribution of 66.9% (Table E). No specific aetiology has been identified, but the number of mutations this signature explains, was correlated with age in normal as well as tumour cells [1]. Another prominent signature is SBS12, as 192 of the 196 samples with a non-zero contribution of this signature belong to this cluster. The transcriptional strand bias observed for this signature [19] is consistent with transcription-coupled repair pathways acting upon dA, as described by Totoki *et al*. SBS12, however, is not an exact match to the signature that was linked to alcohol use by Fujimoto *et al*. and is of unknown aetiology (Table E). Finally, cluster B has in absolute numbers the most samples (77) with a non-zero contribution of the 'tobacco smoking signature' SBS4, which corresponds to ~24% of the samples in this cluster. However, the median contribution is much smaller than for cluster A (19% vs. 48%). In terms of SIMs the ID3 signature, which has been linked to tobacco-smoke

exposure (and other mutagens), is omnipresent in this cluster (94.4% of all samples), but also with a much lower median contribution (25.4%) than for cluster A (71.9%).

Cluster C – high percentage of 1 bp A/T insertions in context of a short homopolymer

Cluster C contains ~96% of the Kidney-RCC and ~51% of the Kidney-ChRCC samples. Together they make up nearly 82% of the 195 samples in this cluster. The most striking feature is a strong positive association with the percentage of 1 bp A/T insertions in no and short homopolymer context (Fig F). This is in contrast to what is observed overall, where 87.4% of the 2,546 samples with 1 bp A/T insertions have more than half of them in the context of a midsize-to-long homopolymer. Also for the three other SIM subtypes there is a positive association for this cluster with a short homopolymer context. There is a negative association with the percentage of recurrent SIMs, which is what we would expect given that recurrence is correlated with the midsize-to-long homopolymer context (Fig 2 - **main**). The features related to recurrence of SSMs are also all negatively associated with this cluster. Other characteristics of this cluster include a high percentage of 1 bp A/T deletions and T>A SSMs. There are nine samples in this cluster that are outliers in terms of the percentage of T>A transversions, meaning a percentage that is larger than the third quartile by at least 1.5 times the interquartile range based on the entire cohort. The median percentage is 44.5% across these nine samples versus 11.4% in the rest of the dataset. This could point to an exposure to aristolochic acid, which is a highly mutagenic substance that forms purine adducts leading to misincorporation of dA opposite dA adducts [20, 21]. The error-prone polymerase ζ (REV3L) may be responsible for this mechanism resulting in T>A SSMs [22].

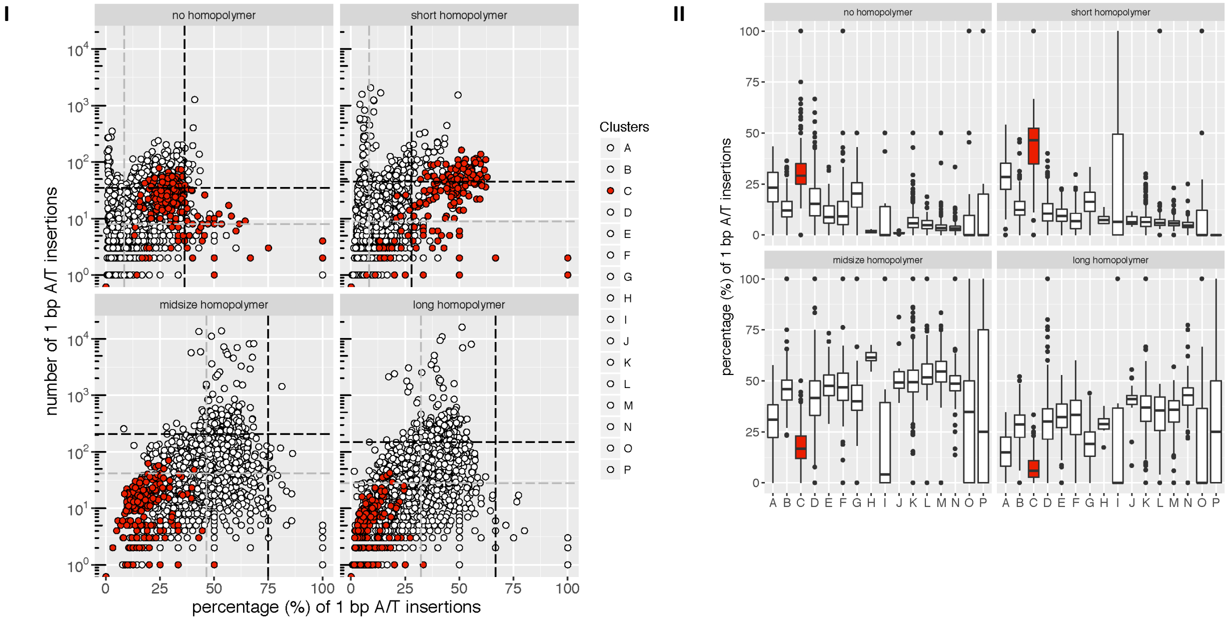

Fig F. Key characteristics of cluster C.

For cluster C the features with the strongest associations are the percentages of 1 bp A/T insertions in no and short homopolymer context (0-4 bp). (I) Percentage versus absolute number of 1 bp A/T insertions in the different homopolymer contexts. The grey lines indicate the medians and the black lines indicate Q3+1.5xIQR, above which samples are outliers. (II) Boxplots of the percentage of 1 bp A/T insertions in the different homopolymer contexts for each cluster.

Annotation

In the whole dataset there are 1.6 and 1.4 times (median across all samples) more SSMs and SIMs, respectively, in late- versus early-replicating regions. In contrast, SSMs and SIMs in this cluster are relatively evenly distributed, as the median ratios are 1.2 and 0.9, respectively. The absence of enrichment of mutations in late-replicating regions matches also the relatively high percentage of the SSMs and SIMs that fall in CDS, regions that are typically replicated early [23], with a median across samples of 1.2% for both mutation types. The most frequently predicted driver in this cluster is the tumour-suppressor gene *VHL* (42.6% of the samples). Another tumour-suppressor gene, *PBRM1*, ranks second with 28.2% of the samples affected, and for 11.3% of the samples *SETD2*, a gene encoding a histone methyltransferase, is likely a driver. All three genes have been described before as playing a role in kidney cancer [24-26]. Interestingly, for as many as ~24% of the samples no driver was discovered and the median number of suggested drivers for the remaining samples is only two. Finally, the three most prevalent SBS signatures (SBS1, SBS5, SBS40) coincide with the most common ones in the entire cohort. Seven of the nine samples with a particular high percentage of T>A SSMs have a contribution of between 16.3% and 82.6% by signature SBS22. This signature largely consists of T>A SSMs in various trinucleotide contexts and has been linked to aristolochic-acid exposure [27]. For ID signatures this is the only

cluster with neither ID1 nor ID2 within the top three. Both of these signatures have been related to the slippage during DNA replication of the replicated DNA strand [1].

Cluster D – low percentage of 1 bp A/T insertions and high percentage of 1 bp C/G deletions

This clusters contains 502 samples from 31 of the 37 tumour types. The five tumour types that contribute the most samples and together constitute more than half of this cluster are Ovary-AdenoCA (17.7%), Breast-AdenoCA (14.5%), Lymph-CLL (8.8%), Panc-Endocrine (7.8%) and Prost-AdenoCA (6.4%). For Ovary-AdenoCA this corresponds to 80.9% of the samples, while for the other four this fraction is below 50% (S2 File). Lymph-CLL is classically divided into two groups [28], those with somatic hypermutation in the variable segments of immunoglobulin genes and those without. We retrieved this information from the article in which the Lymph-CLL samples were described first [4]. Based on this classification 42 of the 49 samples without hypermutation belong to this cluster and only two of the 40 samples with hypermutation. The two features that have the strongest association with this cluster are in positive direction the percentage of 1 bp C/G deletions and in negative direction the percentage of 1 bp A/T insertions (Fig G). Interestingly, this cluster has a positive association with the percentages of the two symmetric substitutions, C>G and T>A, which are the least frequent SSM subtypes. The cluster has negative associations with the number of SIMs and SSMs as well as with any recurrence features. In line with this, there is a positive association with 1 bp SIMs in a non-homopolymer context.

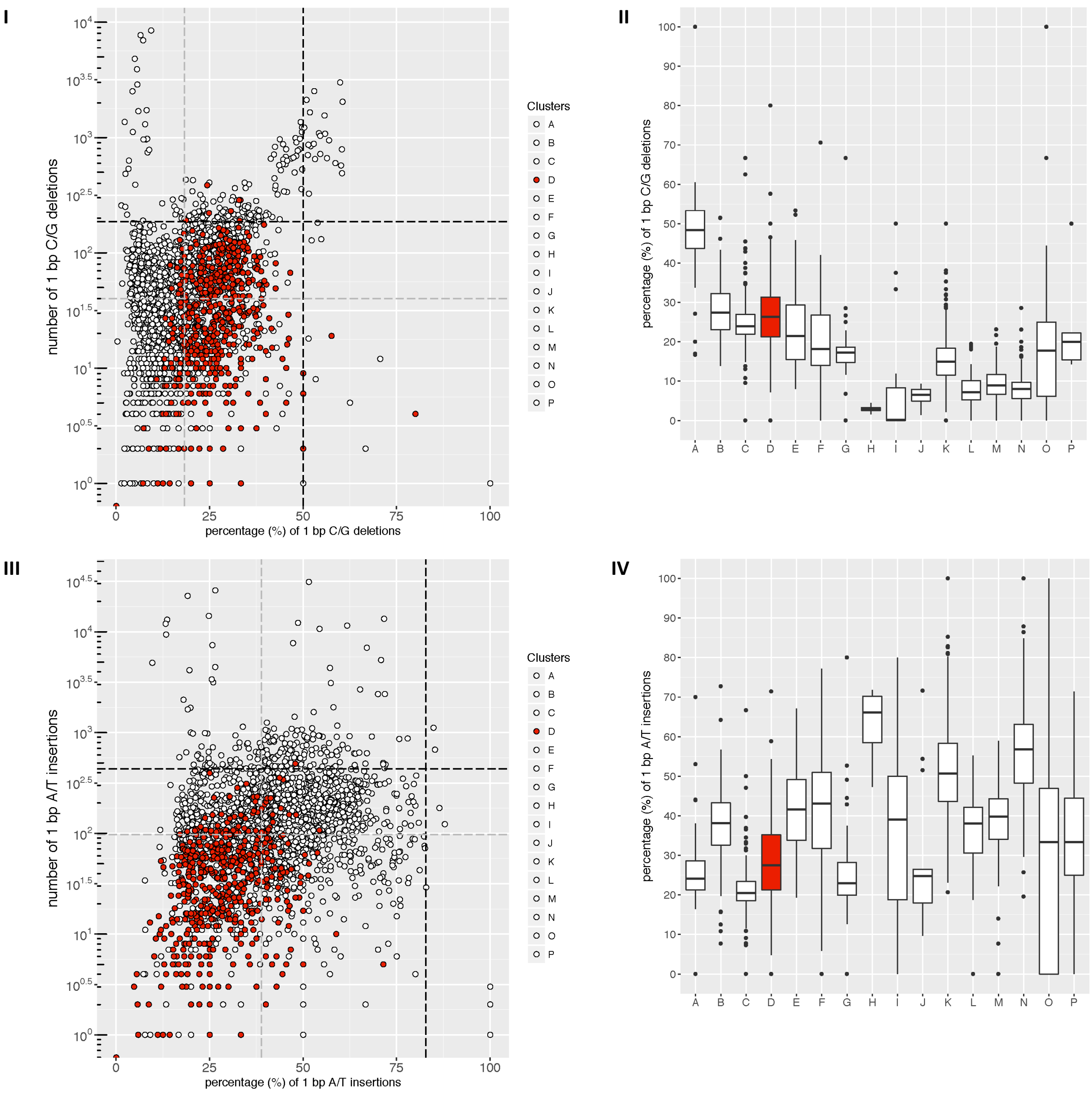

Fig G. Key characteristics of cluster D.

For cluster D the feature with the strongest positive association is the percentage of 1 bp C/G deletions and the strongest negative is the percentage of 1 bp A/T insertions. (I) Percentage versus absolute number of 1 bp C/G deletions. The grey lines indicate the medians and the black lines indicate Q3+1.5xIQR, above which samples are outliers. (II) Boxplots of the percentage of 1 bp C/G deletions for each cluster. (III) Percentage versus absolute number of 1 bp A/T insertions. The grey lines indicate the medians and the black lines indicate Q3+1.5xIQR, above which samples are outliers. (IV) Boxplots of the percentage of 1 bp A/T insertions for each cluster.

Annotation

The median percentage of SIMs in CDS is 1.0%, which is the second highest value (together with cluster B) across all clusters. For 40% of the samples *TP53* is a predicted driver. The second most common driver candidate is *PTEN* (12.4%), encoding a protein relevant for the AKT/PKB signalling pathway. Closely behind follows the *19p13.3a* locus (12.2%). At the level of mutational signatures the top three SBS signatures are the most common ones in the entire cohort (SBS1, SBS5, SBS40). Also for the DBS signatures

nothing stands out with respect to the other clusters. Finally, signature ID9, present in 70.9% of the samples, is more frequent than in any other cluster. The underlying process for this signature is unknown [1].

Cluster E – high percentage of C>G SSMs

There is not a single tumour type that dominates cluster E. The highest numbers of samples are from Breast-AdenoCA (31.6%), Head-SCC (14.3%) and Bladder-TCC (13.3%), which combined form more than half of this cluster. The majority of the Bladder-TCC samples belong to this cluster. The most striking characteristic of this cluster is a strong positive association with the percentage of C>G SSMs (Fig H). The median percentage of C>G SSMs across the samples in this cluster is 26.5%, compared to only 7.7% overall. This also translates into a high absolute number, ranking this cluster second in this respect. There is a positive, but weaker association with the percentage of C>T SSMs and 1 bp C/G deletions. The elevated percentage of C>G combined with C>T SSMs has been described before as being due to increased activity of cytidine deaminases [12] (see also cluster A). The enriched motif uncovered for (recurrent) C>G SSMs (Fig 6 - **main**), suggests a link to deamination mediated by APOBEC3 [29].

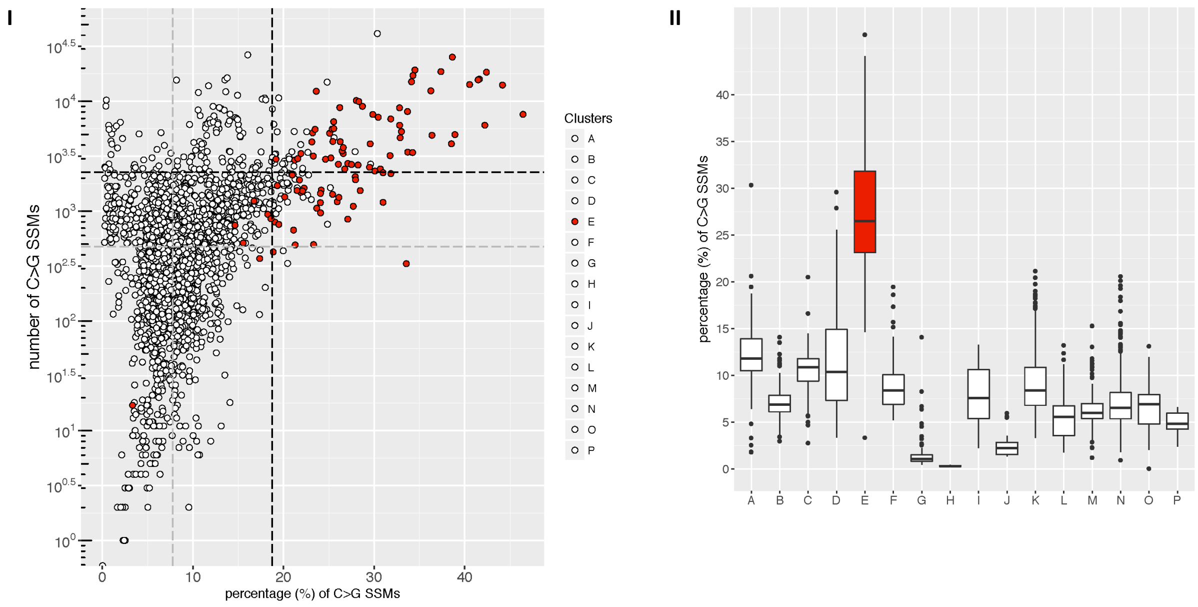

Fig H. Main characteristic of cluster E.

For cluster E the feature with the strongest association is the <u>percentage of C>G SSMs</u>. (I) Percentage versus absolute number of C>G SSMs. The grey lines indicate the medians and the black lines indicate Q3+1.5xIQR, above which samples are outliers. (II) Boxplots of the percentage of C>G SSMs for each cluster.

Annotation

This cluster has the lowest median percentage of SSMs in late-replicating regions (52.8%), together with cluster J. Furthermore, 1.2% of the SSMs are in CDS, which is the second highest value across all clusters. The most frequently predicted driver in this cluster is *TP53* (52% of the samples), followed by *PIK3CA* (24.5%), a gene encoding a kinase implicated in many cancers including breast cancer [30]. *CDKN2A* and the telomerase gene *TERT,* whose expression counteracts cellular senescence, are the third most frequently proposed drivers (both with 18.4%). A high median number of 12 mutational signatures are present per sample (Table C, second highest of all clusters). Signatures SBS13 and SBS2 are both present in 99.0% of the samples with a median contribution of 28.7% and 25.3%, respectively. The first signature has a high proportion of C>G SSMs and the latter a high proportion of C>T SSMs. Both have been suggested to be linked to the activity of the AID/APOBEC family of cytidine deaminases in combination with the faulty repair by error-prone polymerases [31]. Further evidence for APOBEC's role in these signatures and hence in the mutations of the samples of this cluster comes from the observation that germline variations in genes encoding APOBEC3 enzymes are linked to predisposition to bladder and breast cancer as well as to mutational loads of signatures SBS2 and SBS13 [32]. Finally, in 75.5% of the samples DBS11 is present, which may have the same aetiology as the two SBS signatures [1].

Cluster F – high percentage of 1 bp C/G insertions in context of a long homopolymer

There are 95 samples from 26 different tumour types in this cluster. The five tumour types that contribute the most samples and constitute more than half of this cluster are Prostate-AdenoCA (21.1%), CNS-Medullo (9.5%), Breast-AdenoCA (8.4%), Panc-Endocrine (8.4%), and Thyroid-AdenoCA (6.3%). The two features that have the strongest association with this cluster are the percentage of 1 bp C/G insertions in context of a long homopolymer and the percentage of recurrent 1 bp C/G insertions (Fig I). The absolute number of mutations is relatively low in this cluster. The total number of 1 bp C/G insertions per sample ranges from only 1 to 16. Still, of all the 1 bp C/G insertions of this cluster combined, 34.3% of the 405 are recurrent (Table B), when defining recurrence across the entire cohort. In contrast, there is no significant association with overall recurrence, neither for SSMs nor for SIMs.

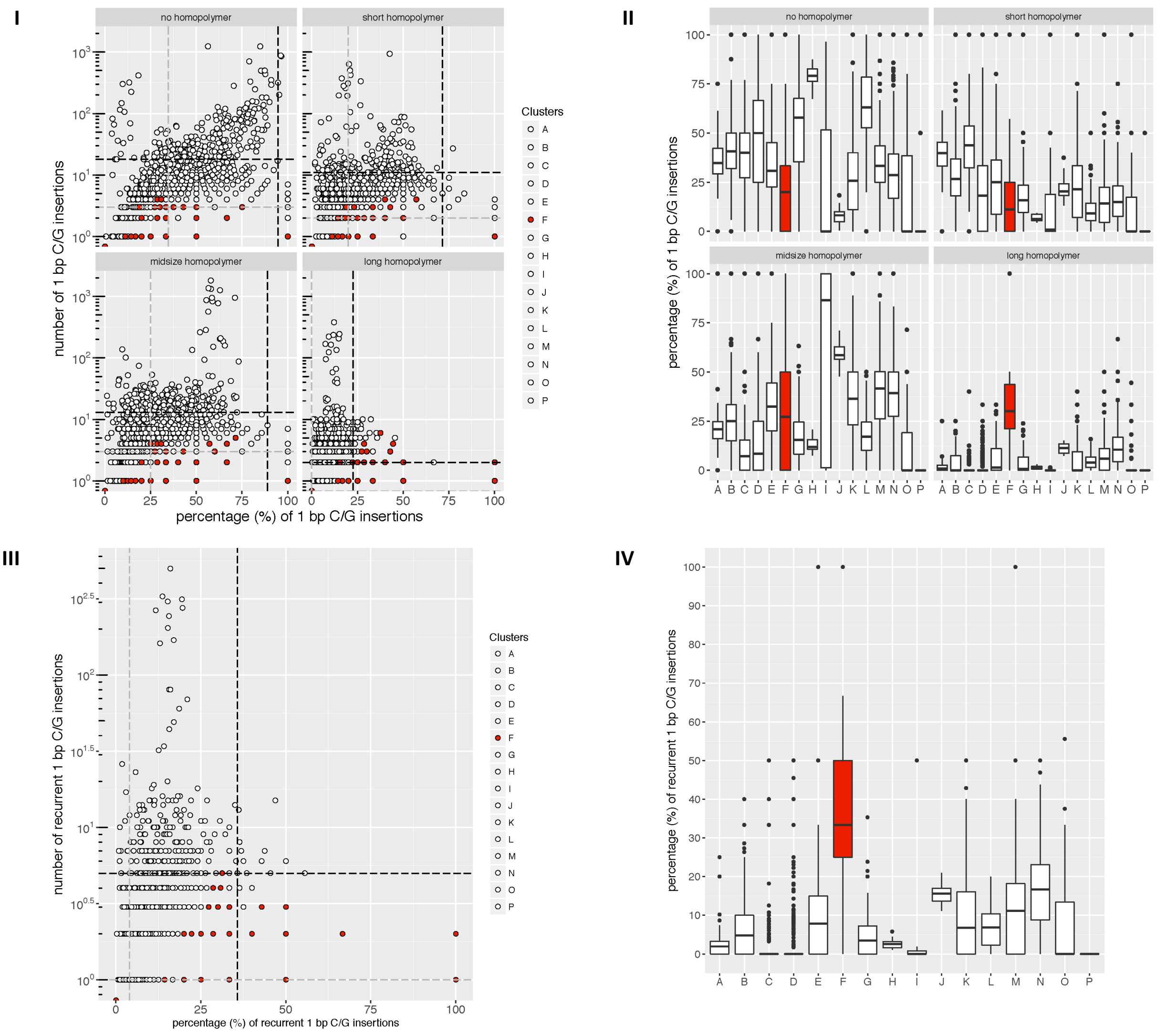

Fig I. Main characteristics of cluster F.

For cluster F the features with the strongest association are the percentage of 1 bp C/G insertions in a long homopolymer (≥ 8 bp) context and the percentage of recurrent 1 bp C/G insertions. (I) Percentage versus absolute number of 1 bp C/G insertions in the different homopolymer contexts. The grey lines indicate the medians and the black lines indicate Q3+1.5xIQR, above which samples are outliers. (II) Boxplots of the percentage of 1 bp C/G insertions in the different homopolymer contexts for each cluster. (III) Percentage versus absolute number of recurrent 1 bp C/G insertions. The grey lines indicate the medians and the black lines indicate Q3+1.5xIQR, above which samples are outliers. (IV) Boxplots of the percentage of recurrent 1 bp C/G insertions for each cluster.

Annotation

The median number of genes mutated by a SIM is only one per sample. With two, the median number of detected drivers is low as well. The top three predicted driver genes are *TP53* (23.2%), *PTEN* (16.8%) and *ERG* (12.6%). The latter encodes a transcription factor with oncogenic potential. In terms of signatures, the most notable are SBS1 and SBS5, which are present in all samples with a median contribution of 12.6% and 51.2%, respectively.

Cluster G – high percentage of recurrent C>T SSMs

This cluster is dominated by Skin-Melanoma samples (79 out of 87) and contains ~74% of all the samples of this tumour type. The high percentage of C>T SSMs, with a median of 84.7% per sample, is the key characteristic of this cluster (Fig J). The high number of C>T substitutions is likely due to UV-induced DNA damage [33-35]. There is a positive association with recurrence of SSMs in general and specifically with recurrent C>T SSMs. The median percentage of recurrent C>T substitutions across samples is 11.1%. Of the 10,150,303 C>T SSMs the samples in this cluster have combined, 4.5% are recurrent within this cluster alone (Table A). This translates to 460,163 in absolute numbers, which corresponds to 60.7% of the total number of recurrent C>T SSMs in the entire cohort.

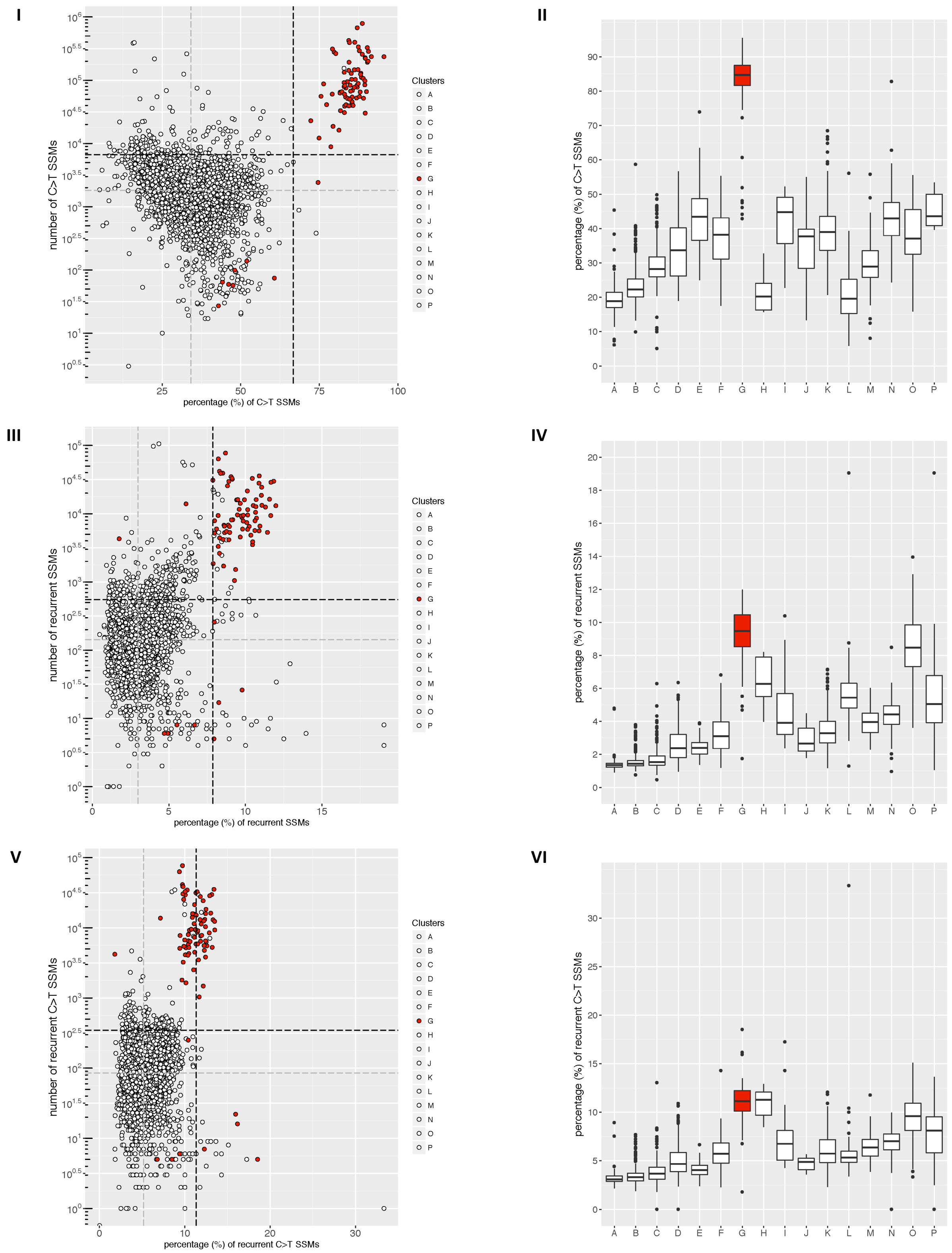

Fig J. Top three characteristics of cluster G.

For cluster G the three features with the strongest association are the <u>percentage of C>T SSMs</u>, the <u>percentage of all recurrent SSMs</u> and the <u>percentage of recurrent C>T SSMs</u>. (I) Percentage versus absolute number of C>T SSMs. The grey lines indicate the medians and the black lines indicate Q3+1.5xIQR, above which samples are outliers. (II) Boxplots of the percentage of C>T SSMs for each cluster. (III) Percentage versus absolute number of recurrent SSMs of all subtypes together. The grey lines indicate the medians and the black lines indicate Q3+1.5xIQR, above which samples are outliers. (IV) Boxplots of the percentage of recurrent SSMs for each cluster. (V) Percentage versus absolute number of recurrent C>T SSMs. The grey lines indicate the medians and the black lines indicate Q3+1.5xIQR, above which samples are outliers. (VI) Boxplots of the percentage of recurrent C>T SSMs for each cluster.

Annotation

The median ratio of synonymous versus non-synonymous SSMs within CDS is significantly higher (~⅓) than in the remaining clusters (~¼) ($p<2.2e-16$, Fig A). Nevertheless, there are still many genes affected by non-synonymous mutations (median: 410, ranked second in this respect). More than two thirds of the samples have *TERT* as a possible driver. Furthermore, over 50% have *BRAF*, a proto-oncogene that plays a crucial role in cell division, and/or *CDKN2A* as a driver. *TP53* is somewhat less frequently altered in this cluster (21.8% of the samples). In terms of the top three of mutational signatures, several signatures are linked to UV-light exposure (SBS7a/b/d, DBS1, ID13) [1] and contribute, as expected, strongly to the observed mutations (between 87.4% and 92.0%, Table E).

Cluster H – high mutational burden of SSMs

There are seven samples from ColoRect-AdenoCA in this cluster and one from Uterus-AdenoCA. Cluster H has the highest median number of SSMs (822,314) across its samples (Fig K) and the second highest for SIMs (9,168), behind cluster J. Cancer genomes with very high number of SSMs are often referred to as ultra-hypermutators [36]. The cluster ranks first for the absolute number of all SSM subtypes with the exception of C>G of which cluster B and F exhibit even more (Table A). In agreement, the median percentage of C>G SSMs per sample is much lower than for the other clusters combined (0.3% vs. 7.8%, $p \approx 1e-06$). The main cause for the ultra-hypermutation is a mutated *POLE* gene, which is considered a driver in all eight samples. It may also explain the low percentage of C>G transversions. Mertz *et al.* showed for yeast that mutations in polymerase δ, the other high-confidence polymerase of the B-family that complements polymerase ε's role in nuclear DNA replication, led to intracellular changes in the dNTP concentrations [37]. This in turn further increased the nucleotide misincorporation rate. They showed that the proportionally lower availability of dG coincided with a reduced percentage of C>G transversions, the observation we also made for these ultra-hypermutated samples. In terms of recurrence there is positive association for C>A and C>T SSMs. There is a high level of recurrence for these two subtypes within the cluster, 3% and 2.2%, respectively (Table A). This translates in absolute numbers into 94,522 C>A SSMs, which corresponds to two thirds of all recurrent C>A SSMs in the entire cohort. For C>T SSMs this corresponds to only 4.5% of all recurrent ones, which is clearly less than in cluster G, despite the higher median number of this SSM subtype in cluster H. This is probably explainable by the low number of samples in this cluster. There is no significant association with recurrence of

the other four SSM subtypes despite the high median number across samples. There is also no significant association with recurrence of SIMs, even though the cluster ranks second in terms of median number across samples for all 1 bp SIM subtypes except for C/G deletions (Table B).

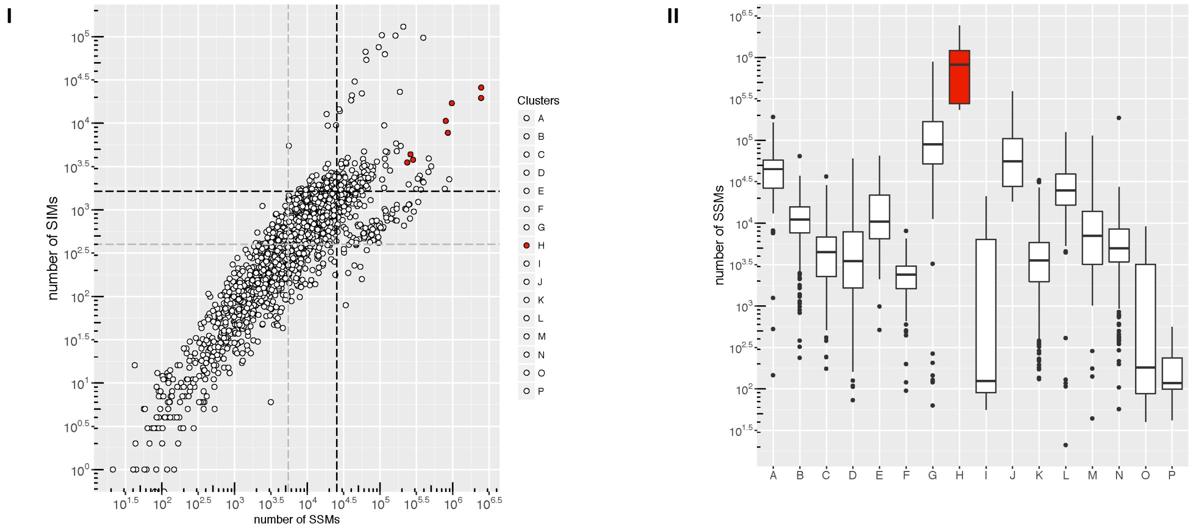

Fig K. Top characteristic of cluster H

For cluster H the strongest association is with the number of SSMs. (I) The absolute number of SSMs versus SIMs. The grey lines indicate the medians and the black lines indicate Q3+1.5xIQR, above which samples are outliers. (II) Boxplots of the absolute number of SSMs for each cluster.

Annotation

About two third of the samples in the entire cohort have a higher percentage of SSMs than SIMs in late-replicating regions. However, this cluster has the largest median difference in the opposite direction, 66.7% of SIMs are in late-replicating regions and only 60.2% of SSMs. This cluster has the lowest percentage of SSMs in CDS (0.6%) and the second lowest for SIMs (0.2%). Nevertheless, the high absolute number of mutations results in the top-ranking position in terms of median number of genes affected by non-synonymous SSMs (3,223) and the second position for the median number of genes altered by SIMs (13.5). In line with this, the cluster has the lowest median percentage of synonymous SSMs (18.7% of all SSMs in CDS). Furthermore, samples of no other cluster have such a high number of suggested drivers (median: 15). Beside *POLE*, all samples have *PIK3CA* as predicted driver. In seven out of the eight samples *APC*, a gene involved in apoptosis and cell migrations, which is often found mutated in malignancies, is considered a driver. In the same number of samples *KRAS*, a well-described proto-oncogene and a major regulator of cell proliferation, is a suggested driver. The only gene mutated by a SIM in more than one sample is *TTN*, which does not point to oncogenic potential, but is likely due to the huge size of this gene encoding the largest human

protein with over 30k amino acids and relevant for muscle contraction. In contrast to many other clusters, *TP53* is considered a driver in only three of the eight samples. Finally, in all samples between 72.4% and 92.7% of the single base substitutions are explained by a combination of the mutational signatures SBS10a, SBS10b and SBS28. The former two are both linked to mutations in *POLE*. Signature SBS28 is of unknown aetiology although usually found in combination with the two SBS10 signatures [1]. Signature DBS3 is also linked to a mutated *POLE* and is found in seven of the eight samples with a contribution of at least 38.9%. For six samples all SIMs are explained by ID1 and ID2 combined. For the other two samples signature ID5 or ID14, both of unknown aetiology, explain the remaining 30.4% and 22.3% of the insertions/deletions, respectively.

Cluster I – high percentage of C/G insertions

This cluster contains 16 samples of which eight are from CNS-PiloAstro. There is a strong positive association with the percentage of 1 bp C/G insertions (Fig L). Although the median of the total number of 1 bp SIMs for samples in this cluster is only four, there are five outliers (three from ColoRect-AdenoCA and two from Panc-AdenoCA) that have relatively high absolute numbers of 1 bp SIMs ranging from 379 to 5,392. With a median number of 125 SSMs this cluster has the second lowest number of substitutions of all clusters and only the five aforementioned samples have substantially higher numbers of SSMs (5,530 - 21,116).

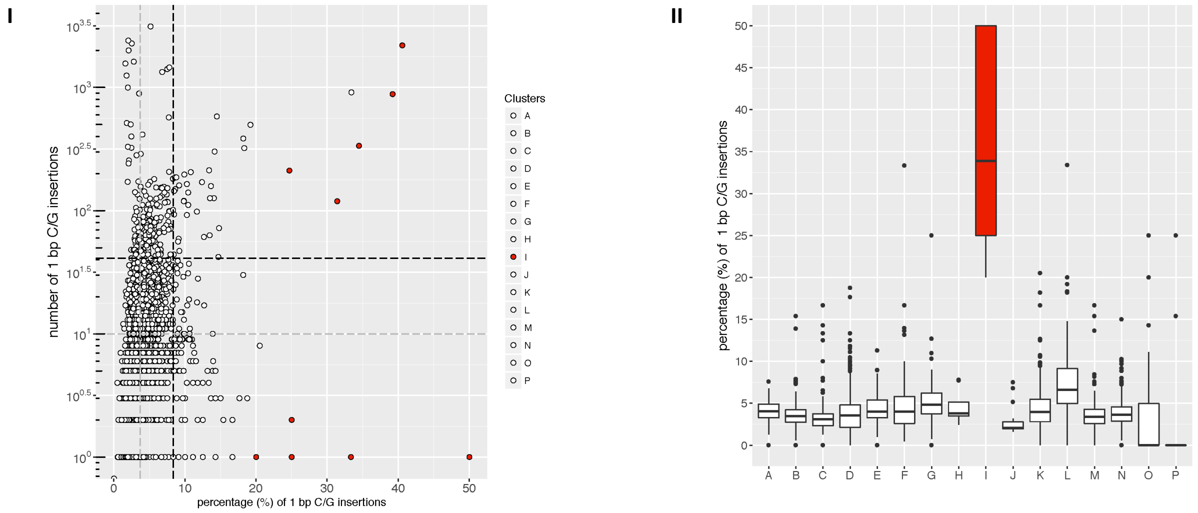

Fig L. Top characteristic of cluster I.

For cluster I the strongest association is with the <u>percentage of 1 bp C/G insertions</u>. (I) Percentage versus absolute number of 1 bp C/G insertions. The grey lines indicate the medians and the black lines indicate Q3+1.5xIQR, above which samples are outliers. (II) Boxplots of the percentage of 1 bp C/G insertions for each cluster.

Annotation

The median number of genes mutated by SSMs and SIMs is two and zero, respectively. In terms of suggested drivers, the median number across samples is only one. A driver affecting all the CNS-PiloAstro samples is *BRAF*. Despite the low number of mutations in this cluster, the median number of signatures per sample is still six. The top three SBS signatures are the most common ones in the entire cohort (SBS1, SBS5, SBS40). For the DBS and ID signatures, only ID1 and ID2 are found in at least half of the samples.

Cluster J – high mutational burden of SIMs

This cluster consists of 17 samples from eight different tumour types, including Uterus-, Stomach- and ColoRect-AdenoCA. It is characterized by the highest median number of SIMs of all clusters (30,228) (Fig M). The high number of SIMs also translates into a high proportion of SIMs per sample. The median percentage is 35.1%, which is almost six times more than the percentage for the entire dataset. The SIM-related feature that particularly stands out is the high percentage of 1 bp C/G deletions in the context of a midsize homopolymer. Moreover, there is a high level of recurrence of SIMs. Of the 179,691 recurrent 1 bp SIMs in the entire cohort, 47.5% are also recurrent when only considering the samples in this cluster. The highest level of recurrence is observed for 1 bp A/T deletions. The samples in this cluster combined have 431,323 different 1 bp A/T deletions of which 17.2% are recurrent within this cluster (Table B). For the 1 bp C/G deletions 6.0% are recurrent within this cluster while for the other clusters this value ranges from 0% to 0.7% (Table B). For SSMs there is no significant association with recurrence and even though this cluster has the second highest median number of T>C SSMs across samples, only 0.2% are recurrent within this cluster and 1%, when referring to the entire cohort.

MSI-Method 1 classified 16 samples in the entire dataset as MSI of which 15 belong to this cluster. The MSI-marked sample not assigned to this cluster is a Skin-Melanoma sample, which is assigned to cluster N instead. It has a clearly lower proportion of total (11.0%) and recurrent (33.0%) SIMs compared to samples that are assigned to cluster J (median of 35.1% and 87.5%, respectively), but higher proportions than other Skin-Melanoma samples (median of 1% and 0.4%). The sample does have the characteristic peak of 1 bp C/G deletions in a midsize homopolymer context. The two samples in this cluster **MSI-Method 1** did not identify as MSI, one from ColoRect-AdenoCA and one from Uterus-AdenoCA, have a high degree of correlation with the MSI-annotated samples for all 42 features. This suggests that they could have been misclassified and

actually be MSI. In fact, MSI assignment is not always straightforward. **MSI-Method 2** labelled 14 of the 17 samples in this cluster as MSI and an additional five samples from other clusters. Aside from the aforementioned Skin-Melanoma sample, the other four neither have a high percentage of (recurrent) mutations that are SIMs, nor do they show a high percentage of 1 bp C/G deletions in the context of a midsize homopolymer.

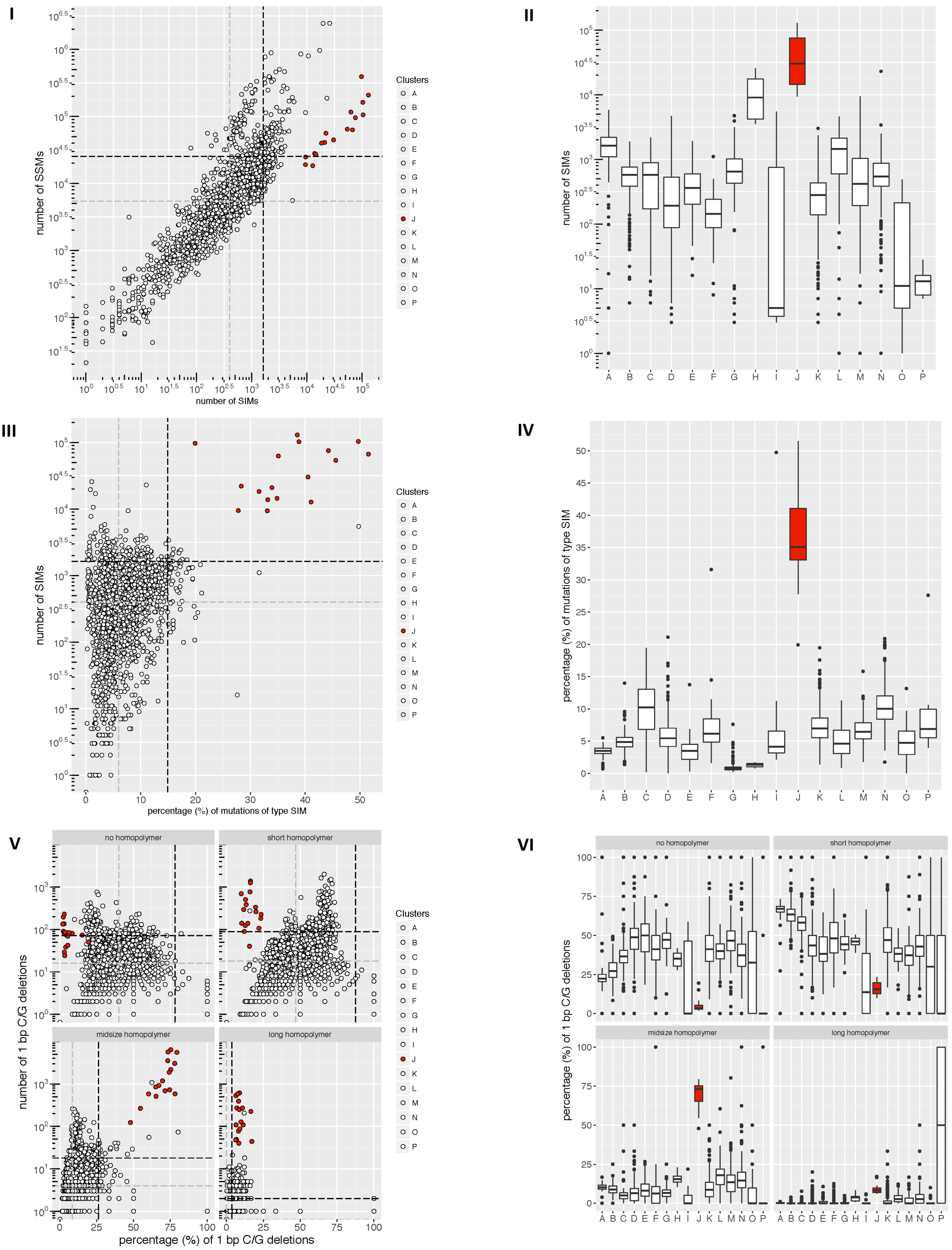

Fig M. Top three characteristics of cluster J.

For cluster J the three features with the strongest associations are the absolute number of SIMs, the percentage of mutations of type SIM and the percentage of 1 bp C/G deletions in a midsize homopolymer context (5-7 bp). (I) Absolute numbers of SIMs versus SSMs. The grey lines indicate the medians and the black lines indicate Q3+1.5xIQR, above which samples are outliers. (II) Boxplots of the absolute number of SIMs for each cluster. (III) The percentage of mutations of type SIM versus the absolute number of SIMs. The grey lines indicate the medians and the black lines indicate Q3+1.5xIQR, above which samples are outliers. (IV) Boxplots of the percentage of mutations of type SIM for each cluster. (V) Percentage versus number of 1 bp C/G deletions in the different homopolymer contexts. The grey lines indicate the medians and the black lines indicate Q3+1.5xIQR, above which samples are outliers. (VI) Boxplots of the percentage of 1 bp C/G deletions in the different homopolymer contexts for each cluster.

Annotation

The median percentage of SSMs and SIMs in late-replicating regions is only 1.1 times higher than in early-replicating regions. This is in line with what has previously been described, *i.e.* that mutations arising after the MMR pathway has been rendered dysfunctional, as is the case in MSI samples, are no longer enriched in late-replicating regions [38]. In agreement with the very high burden of SIMs, this cluster has the highest median number of genes mutated by SIMs (104), which is far more than the median value for the entire dataset (3). In terms of SSMs, this cluster has the highest median percentage of SSMs in CDS (1.3%), but a relatively large percentage (median of 28.3%) of those SSMs are synonymous. The median number of detected drivers is 8, which is higher than for most clusters, but none of them affect more than half of the samples. The two most common drivers in this cluster (47.1% of the samples) are *RPL22*, which encodes a ribosomal protein often altered in MSI-related tumours [39] and *ARID1A*. Ranking second is *ACVR2A* (41.2%), an ubiquitously expressed transmembrane kinase, which has previously been predicted, based on a dataset of 6,747 cancer exomes from TCGA, to contain recurrently mutated, cancer-driving microsatellites [40]. Interestingly, *TP53* is only a driver in roughly one third of the samples. Finally, there are seven SBS signatures (SBS6, SBS14, SBS15, SBS20, SBS21, SBS26 and SBS44) that have been linked to defective DNA MMR, two DBS signatures (DBS7, DBS10), and three ID signatures (ID1, ID2 and ID7). The signatures ID1 and ID2 are found in most cancer genomes, but it has been noted that they tend to have a high absolute contribution to MSI samples (>10,000) [1]. This is indeed the case for 15 of the 17 samples in this cluster and combined these two ID signatures fully explain all SIMs of these 17 samples. For the remaining two samples these two signatures also explain all SIMs except that the total number is slightly lower (>9,300). The signature ID7 is not present in any of the samples in this cluster. One MSI-annotated sample in this cluster has zero contribution of all seven SBS signatures combined. The other 16 samples of the cluster, including the two not marked as MSI by the PCAWG consortium, have a combined contribution of these seven signatures of at least 39%. With respect to the two DBS signatures, 11 samples have a combined contribution of at least ~26%, whereas the others have none.

Cluster K – high percentage of 1 bp A/T insertions

This is the largest of all 16 clusters with 522 samples from 33 different tumour types. The four tumour types that contribute the most samples and constitute more than half of this cluster are Prost-AdenoCA (20.1%), CNS-Medullo (14.4%), Breast-AdenoCA

(13.2%) and Panc-AdenoCA (11.7%). The strongest positive association in this cluster is with the percentage of 1 bp A/T insertions (Fig N). For this SIM subtype there is also a positive association in terms of recurrence. There is no significant association with recurrence of SSMs in general, but there are positive associations with three specific subtypes (C>A, C>T and T>A).

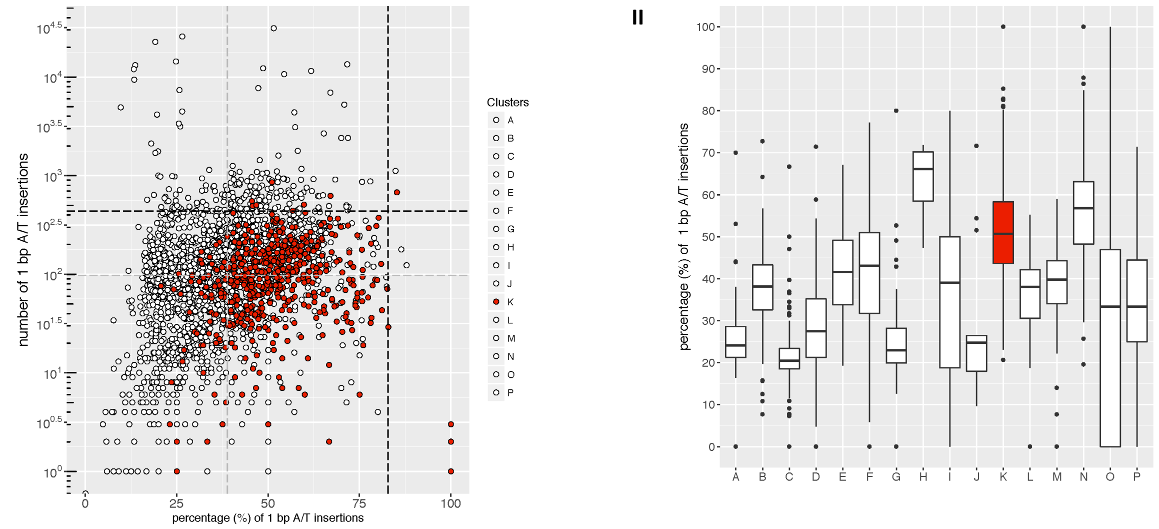

Fig N. Top characteristic of cluster K.

For cluster K the strongest association is with the <u>percentage of 1 bp A/T insertions</u>. (I) Percentage versus absolute number of 1 bp A/T insertions. The grey lines indicate the medians and the black lines indicate Q3+1.5xIQR, above which samples are outliers. (II) Boxplots of the percentage of 1 bp A/T insertions for each cluster.

Annotation

This cluster does not show any particular characteristics as far as the annotation layers are concerned. While the median percentages of SSMs and SIMs in CDS for this cluster are around the overall median, corresponding numbers for late-replicating regions are below the overall value. In about one third of the samples *TP53* is likely to be driver, followed by *CDKN2A* (15.7%) and *PTEN* (13%). The top three SBS signatures are the most common ones in the entire cohort (SBS1, SBS5, SBS40). Also for the DBS and ID signatures, this cluster does not stand out from the others.

Cluster L – high percentage of recurrent T>G SSMs

Eso-AdenoCA (62.5%) and Stomach-AdenoCA (20.2%) samples combined constitute the vast majority of the samples in cluster L. The strongest positive association in this cluster is with the percentage of T>G SSMs (Fig O). There is also a positive association with the percentage of T>C SSMs. As for absolute numbers of T>G and T>C SSMs, the median across samples is 6,769 and 4,956, respectively (Table A). This ranks the cluster second for T>G SSMs and fourth for T>C SSMs. Accordingly, two mutational signatures

have been described to be unique for Eso-AdenoCA, one with a high percentage of T>G SSMs and the other with additionally a relatively high percentage of T>C SSMs. These signatures have been suggested to be linked to gastric reflux [41, 42], whereby the low pH as well as bile acids could result in oxidative DNA damage particularly affecting purines [43]. Tomkova *et al.* recently proposed that, in the case of gastric cancers and oesophagus adenocarcinoma, the T>G SSMs may be explained by oxidative damage to dG in the dNTP pool [44]. In fact, error-prone DNA polymerases have been shown to incorporate the oxidized dG opposite dA during replication, which would then result in a A>C manifestation after the next replication round [45].

With respect to recurrence of SSMs, the cluster again shows a particularly strong association with T>G SSMs, followed by T>C SSMs. When only considering the samples within this cluster 3.6% of the T>G and 1.1% of the T>C SSMs are recurrent. These numbers correspond to the highest proportions of all clusters with respect to the total number of recurrent T>G and T>C SSMs of the entire cohort (45.0% and 19.7%, respectively). This suggests that the mutational processes behind both SSM subtypes are not random and more complex than only via the production of high levels of oxidative stress. In terms of SIMs, there is a positive association with recurrence of all subtypes except for the 1 bp C/G insertions. The strongest association is with the recurrence of 1 bp A/T deletions, for which there is also a positive association with the long homopolymer context feature.

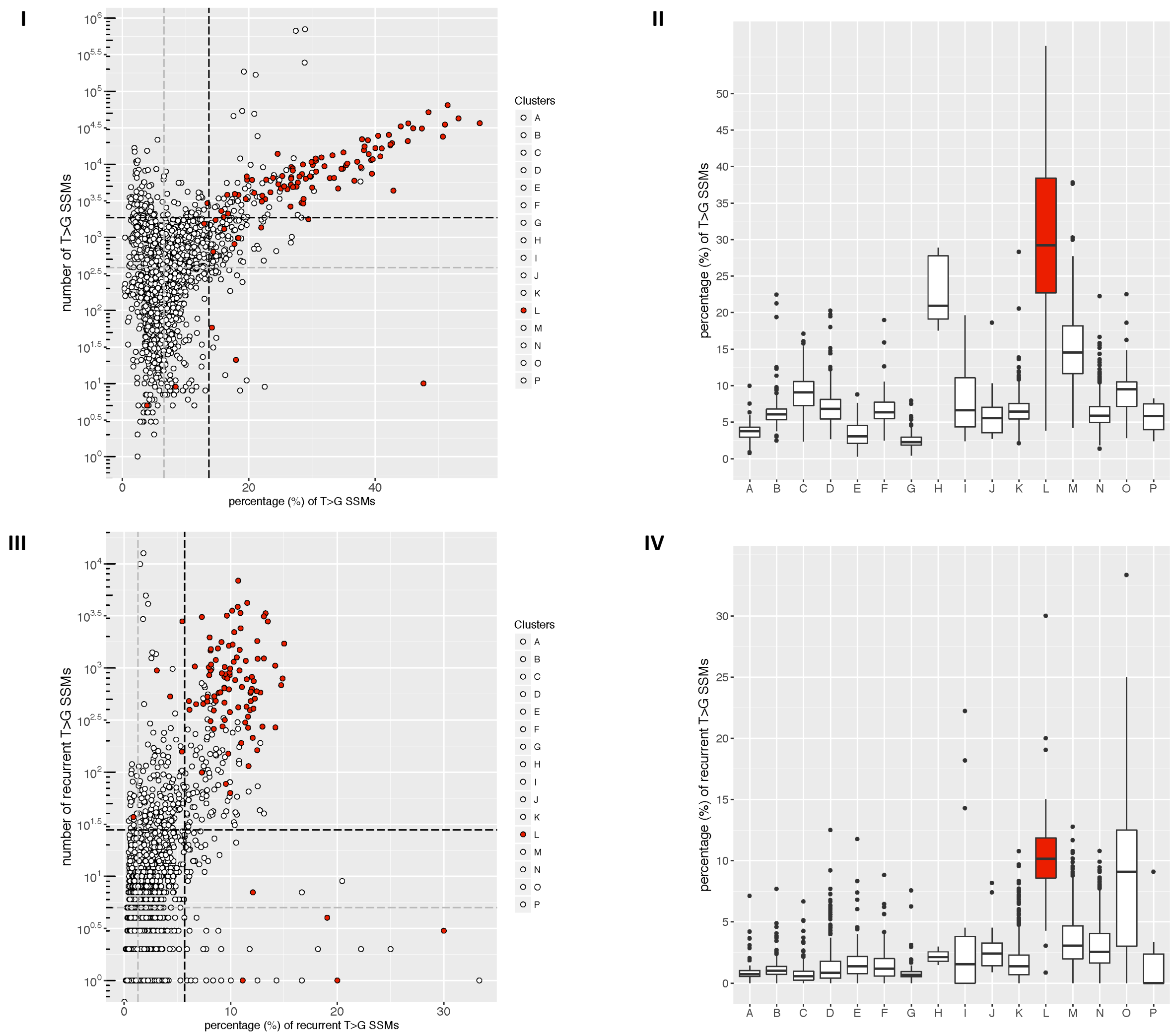

Fig O. Top two characteristics of cluster L.

For cluster L the two features with the strongest associations are the percentage of T>G SSMs and the percentage of recurrent T>G SSMs. (I) Percentage versus absolute number of T>G SSMs. The grey lines indicate the medians and the black lines indicate Q3+1.5xIQR, above which samples are outliers. (II) Boxplots of the percentage of T>G SSMs for each cluster. (III) Percentage versus absolute number of recurrent T>G SSMs. The grey lines indicate the medians and the black lines indicate Q3+1.5xIQR, above which samples are outliers. (IV) Boxplots of the percentage of recurrent T>G SSMs for each cluster.

Annotation

There is a strong enrichment of SSMs in late-replicating regions with a median percentage of 75.2%, which is three times higher than in early-replicating regions and the highest percentage of all clusters. Also for SIMs a high median percentage is in late-replicating region (67.2%), only marginally exceeded by cluster M. In line with these observations is that only 0.7% and 0.5% of SSMs and SIMs, respectively, fall in CDS, which are, as mentioned, typically in early-replicating regions. We speculate that mutagenic influences typical for this cluster (*e.g.* gastric or bile acid) could more severely affect late-replicating regions due to the longer transient exposure of single-stranded DNA [46]. Another explanation would be that if oxidative damage to the dNTP

pool underlies the T>G SSMs [44], the faulty incorporation of oxidized dG into DNA depends on error-prone polymerases [45] active in late stages of replication [47]. One caveat to these interpretations is the approximation of replication times by calculating the median of values from five cancer cell lines, of which none represents the cell types of the main tumour types in this cluster.

In terms of drivers, *TP53* stands out as it is considered a driver in 75% of the samples, which is the highest percentage in all clusters. At second place is *CDKN2A* (31.7%), followed by *ARID1A* and *SMAD4* (18.3%), which is part of the TGFβ signalling pathway. Although not considered a driver, *PCLO* was altered by non-synonymous mutations in 33.8% of the Eso-AdenoCA samples in this cluster and 29.8% of all samples in this cluster. Mutations in this gene encoding a presynaptic cytoskeletal protein have recently been suggested as a prognostic marker in oesophageal squamous-cell carcinoma (Eso-SCC) [48]. Further genes which are affected by non-synonymous mutations in a material number of samples include *HMCN1* (28.9%), a cell polarity-related gene previously found mutated in cancers of the gastro-intestinal tract [49], *SYNE1* (26%), encoding a nuclear membrane protein and candidate gene for oesophagogastric junctional adenocarcinoma [50], as well as *LRP1B* (25%), a tumour suppressor gene previously related to Eso-SCC [51]. Finally, the median number of mutational signatures present across samples is 14, the highest of all clusters. Signatures SBS17a and SBS17b are present in 95.2% of the samples with a median contribution of 23.7% and 12.1%, respectively. Despite the resemblance to the aforementioned signatures found by Secrier *et al.*, the PCAWG working group did not link the signatures to any aetiology.

Cluster M – high percentage of recurrent 1 bp A/T deletions and C/G SSMs

Two lymphoid cancers, Lymph-BNHL (51.6%) and Lymph-CLL (20.1%), together make up more than 70% of cluster M. Nearly all Lymph-BNHL samples are in this cluster (88.8%). While each of the 13 features capturing recurrence is positively associated with this cluster, the strongest association is observed with recurrent 1 bp A/T deletions (Fig P). Related to that is the strong association with the general feature of the percentage of 1 bp A/T deletions in a long homopolymer context. With respect to recurrent SSMs the strongest positive association is observed for C>G transversions, for which the median percentage of recurrence per sample is 3.8% (versus 0.6% across the entire dataset). Despite eight clusters having a higher median total number of C>G SSMs across samples, this cluster has the highest absolute number of C>G SSMs that are

recurrent within the cluster, which represent 10.7% of the total amount in the cohort. Interestingly, for the general features the cluster shows elevated percentages of the three possible thymidine substitutions (T>A/C/G), while the three possible cytosine substitutions (C>A/G/T) are reduced.

For the samples of Lymph-BNHL and Lymph-CLL in this cluster combined we observe peaks of recurrent mutations at immunoglobulin genes (Fig Q). This points to a large proportion of samples from non-naïve B-cells, in which somatic hypermutation has already taken place. This process involves the error-prone polymerase η and results in mutations preferably at A and T bases [52]. Polymerase η is further known to efficiently bypass various dT lesions, but with a higher chance of nucleotide misincorporation [53]. For the Lymph-CLL samples in PCAWG, annotation according to hypermutation status is available [4]. This earlier study classified 40 samples as hypermutated of which 36 samples are in cluster M. Of the 49 Lymph-CLL samples classified as non-hypermutated, only one is assigned to this cluster. The hypermutation of the immunoglobulin genes is likely one explanation of the observed high level of recurrence, as these regions represent only a small part of the genome. In contrast, Lymph-CLL samples without the hypermutation phenotype show negative association with recurrence (cluster D).

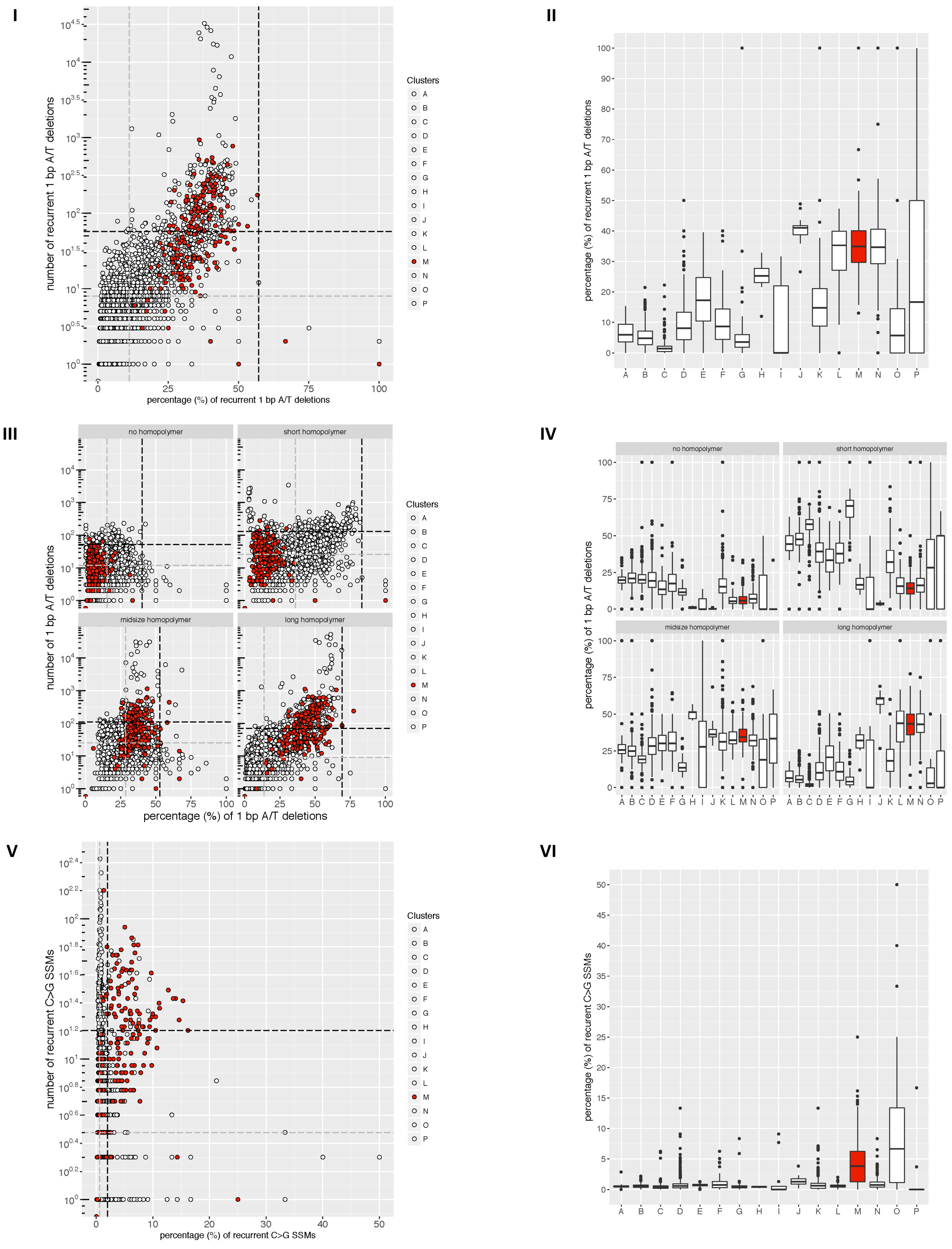

Fig P. Top three characteristics of cluster M.

For cluster M the three features with the strongest association are the percentage of recurrent 1 bp A/T deletions, the percentage of 1 bp A/T deletions in a long homopolymer context (≥ 8 bp), and the percentage of recurrent C>G SSMs. (I) Percentage versus absolute number of recurrent 1 bp A/T deletions. The grey lines indicate the medians and the black lines indicate Q3+1.5xIQR, above which samples are outliers. (II) Boxplots of the percentage of recurrent 1 bp A/T deletions for each cluster. (III) Percentage versus absolute number of 1 bp A/T deletions in the different homopolymer contexts. The grey lines indicate the medians and the black lines indicate Q3+1.5xIQR, above which samples are outliers. (IV) Boxplots of the percentage of 1 bp A/T deletions in the different homopolymer contexts for each cluster. (V) Percentage versus absolute number of recurrent C>G SSMs. The grey lines indicate the medians and the black lines indicate Q3+1.5xIQR, above which samples are outliers. (VI) Boxplots of the percentage of recurrent C>G SSMs for each cluster.

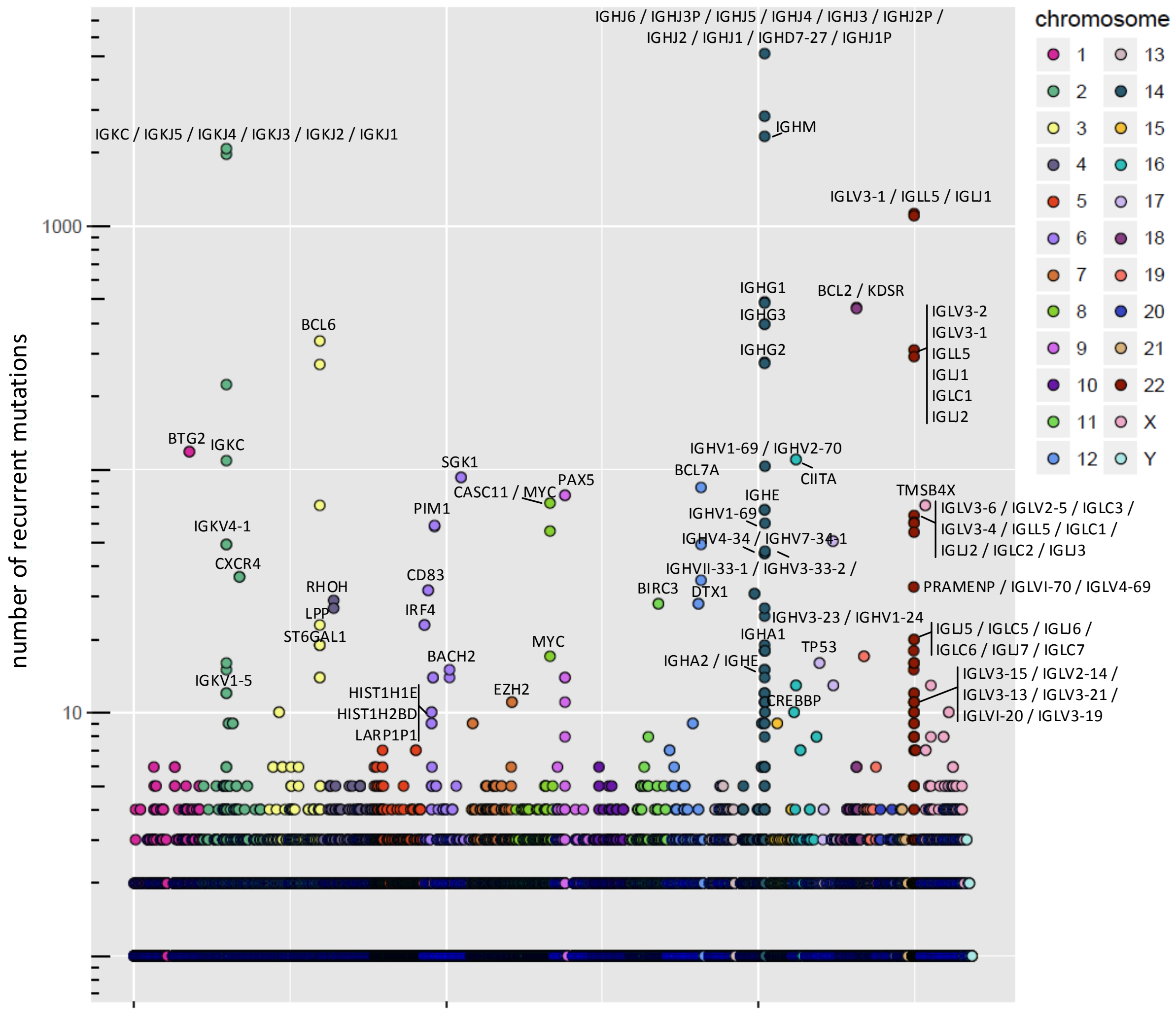

Fig Q. Peaks of recurrent mutations along the genome for Lymph-BNHL and Lymph-CLL in cluster M.
Each point in the plot represents a window and indicates the number of recurrent mutations in the combined set of 95 Lymph-BNHL and 37 Lymph-CLL samples that are in cluster M. Recurrence is defined with respect to the entire cohort. Windows without any recurrent mutations in these samples are left out. A subset of the windows that overlap with gene(s) are labelled accordingly.

Annotation

There are twice as many SSMs in late-replicating regions as in early-replicating regions (median across the samples). For SIMs, the imbalance is even more pronounced as the median percentage in late-replicating regions is 67.5%, which is the highest value of all clusters. In terms of drivers, *TP53* (29.3%) is the top-ranking gene, followed by *BCL2* (23.4%), playing a role in apoptosis of lymphocytes [54] and *CREBBP* (19%), encoding a nuclear protein previously associated with leukemia [55]. The *IGLL5* gene, located in the immunoglobulin lambda locus, is altered by non-synonymous mutations in ⅓ of the lymphoid cancer samples in this cluster and represents an effect of the somatic hypermutation. In 39.1% of the samples signature SBS9 is present, which has been linked to polymerase η activity [1]. With respect to the 36 Lymph-CLL samples that were indicated to be hypermutated in this cluster, 33 of them have a non-zero

contribution of this signature. Of the 86 samples in the entire cohort in which this signature is present, ~84% are in this cluster.

Cluster N – high proportion of recurrent SIMs

The majority of the samples in cluster N are from Panc-AdenoCA (46.6%) or CNS-Medullo (16.4%). Also ~56% of all ColoRect-AdenoCA samples are in this cluster. Except for C>G and T>C SSMs, all other features capturing recurrence are positively associated with this cluster. The level of recurrence is one aspect that differentiates the Panc-AdenoCA from tumours belonging to Panc-Endocrine (cluster D). The high level of recurrence is particularly notable for CNS-Medullo where the median total number of mutations (1,359) is much lower than, for example, in Panc-AdenoCA samples of this cluster (5,626). The strongest association is observed with recurrent 1 bp A/T deletions (Fig R) along with, as expected, the same SIM subtype in the context of a long homopolymer. Another feature that particularly stands out in terms of recurrence is the high proportion of SIMs. The median percentage of recurrent mutations that are SIMs is 34.1%, which is twice as high as in the entire dataset. Finally, this cluster is ranked second in terms of the percentage of T>A SSMs that are recurrent within the cluster (Table A). The same holds for recurrence in the entire cohort for this SSM subtype and in absolute numbers the cluster even ranks first.

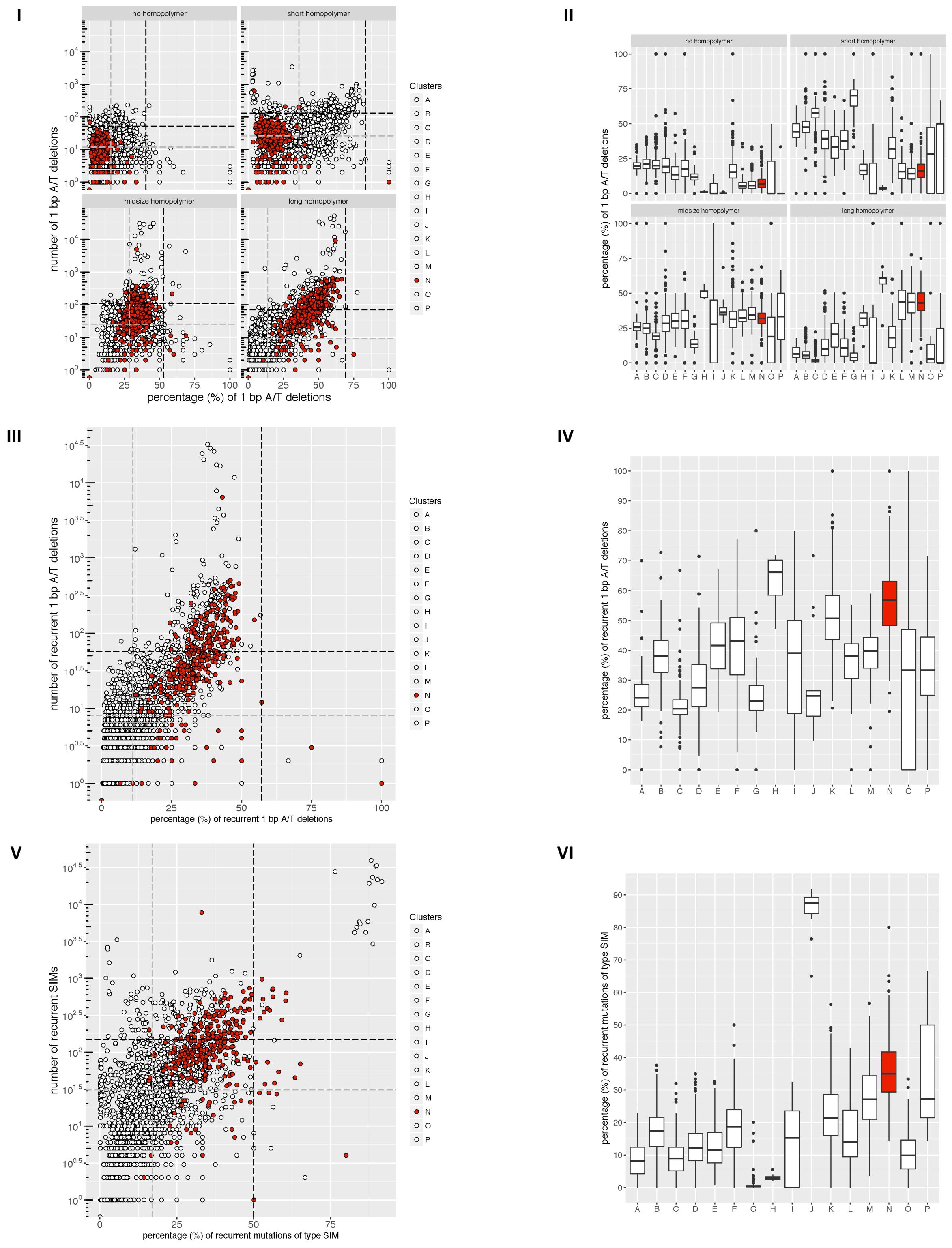

Fig R. Top three characteristics of cluster N.

For cluster N the three features with the strongest associations are the percentage of 1 bp A/T deletions in a long homopolymer context (≥ 8 bp), the percentage of recurrent 1 bp A/T deletions and the percentage of recurrent mutations that is of type SIM. (I) Percentage versus absolute number of 1 bp A/T deletions in the different homopolymer contexts. The grey lines indicate the medians and the black lines indicate Q3+1.5xIQR, above which samples are outliers. (II) Boxplots of the percentage of 1 bp A/T deletions in the different homopolymer contexts for each cluster. (III) Percentage versus absolute number of recurrent 1 bp A/T deletions. The grey lines indicate the medians and the black lines indicate Q3+1.5xIQR, above which samples are outliers. (IV) Boxplots of the percentage of recurrent 1 bp A/T deletions for each cluster. (V) Percentage of recurrent mutations of type SIM versus the absolute number of recurrent SIMs. The grey lines indicate the medians and the black lines indicate Q3+1.5xIQR, above which samples are outliers. (VI) Boxplots of the percentage of recurrent mutations of type SIM for each cluster.

Annotation

A median of 1.2% of the SSMs falls in CDS, which is, together with clusters A, F and H, the second highest percentage of all clusters. More than half of the samples have *TP53* (56.3%) and/or *KRAS* (51.8%) as predicted drivers. This percentage increases to ~81% and ~95%, respectively, when only considering the Panc-AdenoCA samples in this cluster. *CDKN2A* ranks third with 43.7%, which also increases substantially to ~78% in Panc-AdenCA samples. All three genes are considered relevant for the development of this tumour type [56]. In terms of signatures, SBS1, SBS5 and SBS40 show large contributions to the samples of this cluster, which is, however, in general the case across the samples of the entire cohort. A signature more specific to this cluster is SBS18, which is present in ~48% of the samples, including 28 of the 29 ColoRect-AdenoCA samples and 11 of the 12 Stomach-AdenoCA samples. A suggested aetiology for this signature is damage caused by reactive oxygen species [1]. For insertions/deletions, the main signatures are ID1 and ID2, which are present in most samples of the cohort.

Cluster O – high percentage of recurrent SSMs for each subtype

Samples from Prost-AdenoCA and CNS-PiloAstro together make up nearly 80% of this cluster, which consists of 43 samples in total. There are positive associations with 9 of the 13 recurrence features, particularly with recurrent SSMs of type T>C, C>G and T>A (Fig S). However, the absolute number of SSMs and SIMs is very low in this cluster with a median number of 182 and 11, respectively.

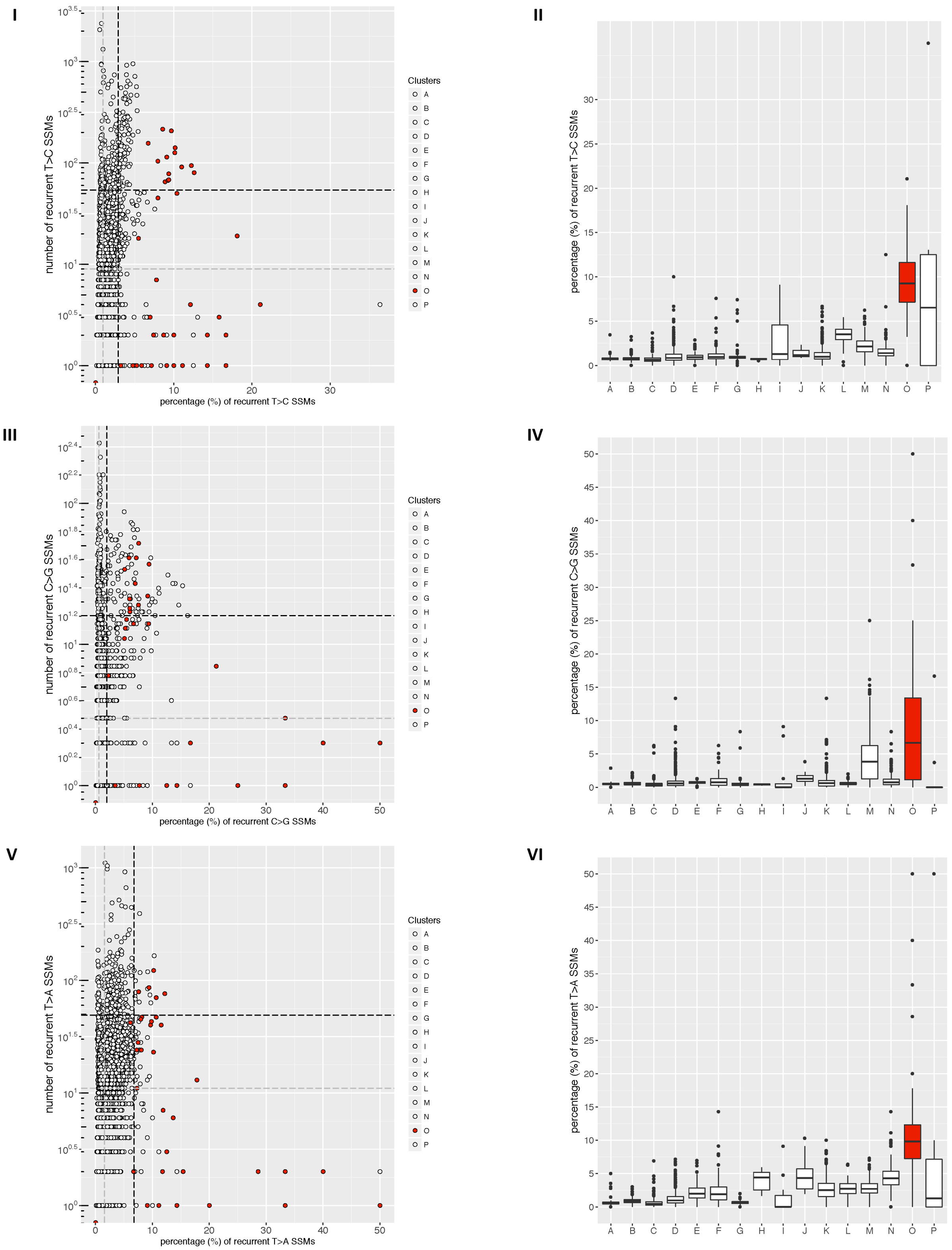

Fig S. Top three characteristics of cluster O.

For cluster O the three features with the strongest association are the <u>percentage of recurrent T>C, C>G and T>A SSMs</u>. (I) Percentage versus absolute number of recurrent T>C SSMs. The grey lines indicate the medians and the black lines indicate Q3+1.5xIQR, above which samples are outliers. (II) Boxplots of the percentage of recurrent T>C SSMs for each cluster. (III) Percentage versus absolute number of recurrent C>G SSMs. The grey lines indicate the medians and the black lines indicate Q3+1.5xIQR, above which samples are outliers. (IV) Boxplots of the percentage of recurrent C>G SSMs for each cluster. (V) Percentage versus absolute number of recurrent T>A SSMs. The grey lines indicate the medians and the black lines indicate Q3+1.5xIQR, above which samples are outliers. (VI) Boxplots of the percentage of recurrent T>A SSMs for each cluster.

Annotation

In 12 of the 43 samples no drivers were detected. The remaining samples have a median number of just one driver. Most frequently predicted driver genes are *BRAF* (14 samples), *ERG* (8 samples) and *CDKN1B* (5 samples). The latter plays a role in the cell cycle progression at the G1 phase. The median number of signatures linked to each sample is seven, which is surprisingly high given the low median number of mutations. The top three signatures for SBS, DBS and ID, are all among the most common ones in the entire cohort (SBS1, SBS5, SBS40; DBS2, DBS4, DBS9; ID1, ID2, ID5).

Cluster P – percentage of 1 bp C/G deletions in context of a long homopolymer

This cluster captures only nine samples from four different tumour types (CNS-Medullo, CNS-PiloAstro, Prost-AdenoCA and Thy-AdenoCA). A positive association with the percentage of 1 bp C/G deletions in context of a long homopolymer as well as with the percentage of recurrent 1 bp C/G deletions are the most notable features (Fig T). The absolute numbers of SSMs and SIMs are quite low per sample and range from 42 to 560 and from 7 to 28, respectively.

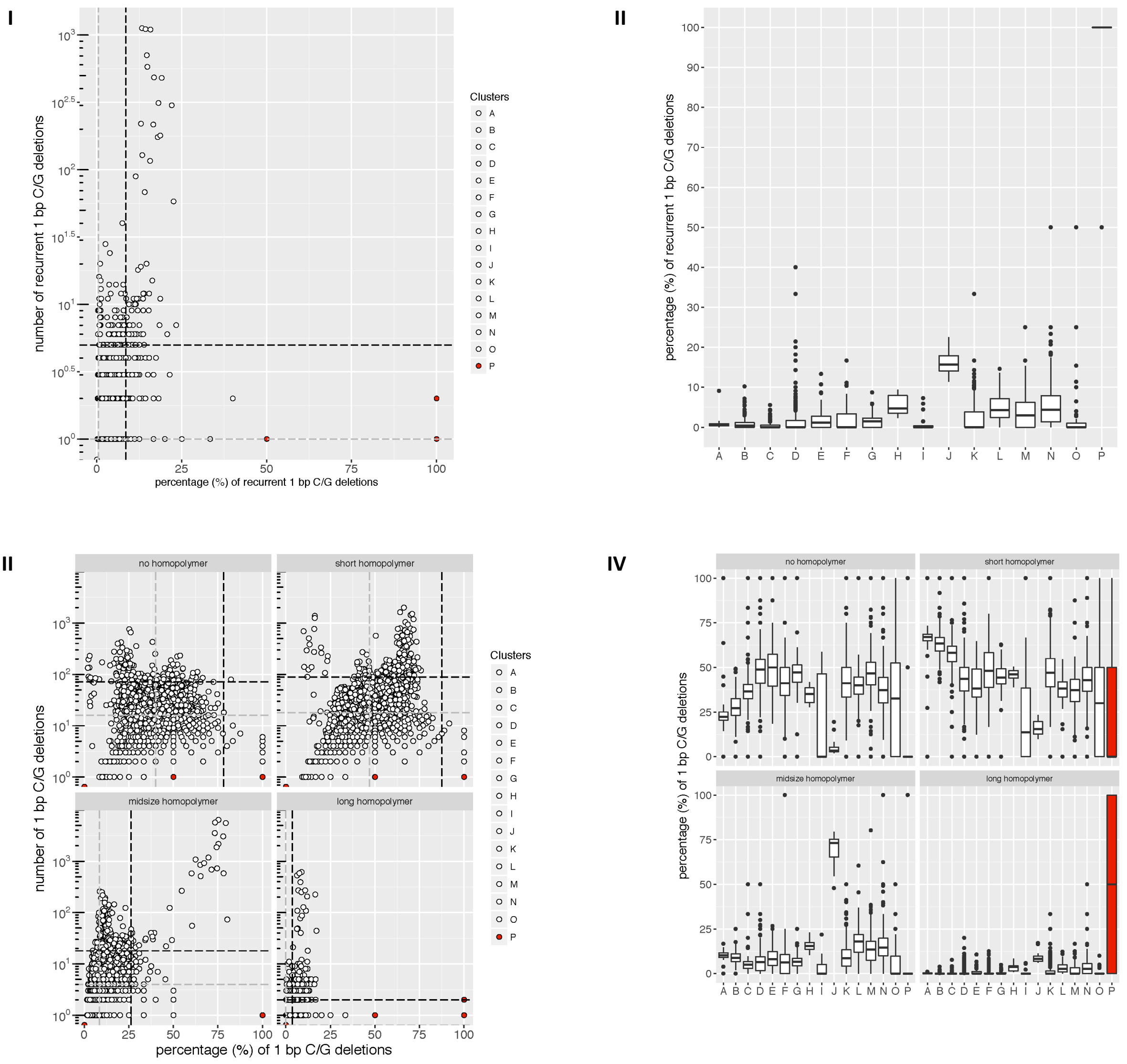

Fig T. Top two characteristics of cluster P.

For cluster P the two features with the strongest associations are the percentage of recurrent 1 bp C/G deletions and the percentage of 1 bp C/G deletions in a long homopolymer context (≥ 8 bp). (I) Percentage versus absolute number of recurrent 1 bp C/G deletions. The grey lines indicate the medians and the black lines indicate Q3+1.5xIQR, above which samples are outliers. (II) Boxplots of the percentage of recurrent 1 bp C/G deletions for each cluster. (III) Percentage versus absolute number of 1 bp C/G deletions in the different homopolymer contexts. The grey lines indicate the medians and the black lines indicate Q3+1.5xIQR, above which samples are outliers. (IV) Boxplots of the percentage of 1 bp C/G deletions in the different homopolymer contexts for each cluster.

Annotation

For six out of the nine samples *BRAF* is suggested as a driver. The median number of signatures active in a sample is five, the lowest number of all clusters. The most frequently contributing signatures for SBS, DBS and ID, are all the most common ones in the entire cohort (SBS1, SBS5, SBS40; DBS2, DBS9; ID1, ID2, ID5).

References

1. Alexandrov L, Kim J, Haradhvala NJ, Huang MN, Ng AWT, Boot A, et al. The Repertoire of Mutational Signatures in Human Cancer. bioRxiv. 2018:322859. doi: 10.1101/322859.

2. Waszak SM, Tiao G, Zhu B, Rausch T, Muyas F, Rodriguez-Martin B, et al. Germline determinants of the somatic mutation landscape in 2,642 cancer genomes. bioRxiv. 2017. doi: 10.1101/208330.

3. Niu B, Ye K, Zhang Q, Lu C, Xie M, McLellan MD, et al. MSIsensor: microsatellite instability detection using paired tumor-normal sequence data. Bioinformatics. 2014;30(7):1015-6. doi: 10.1093/bioinformatics/btt755.

4. Puente XS, Bea S, Valdes-Mas R, Villamor N, Gutierrez-Abril J, Martin-Subero JI, et al. Non-coding recurrent mutations in chronic lymphocytic leukaemia. Nature. 2015;526(7574):519-24. doi: 10.1038/nature14666.

5. Ramos AH, Lichtenstein L, Gupta M, Lawrence MS, Pugh TJ, Saksena G, et al. Oncotator: Cancer Variant Annotation Tool. Hum Mutat. 2015;36(4):E2423-E9. doi: 10.1002/humu.22771.

6. Harrow J, Frankish A, Gonzalez JM, Tapanari E, Diekhans M, Kokocinski F, et al. GENCODE: the reference human genome annotation for The ENCODE Project. Genome Research. 2012;22(9):1760-74. doi: 10.1101/gr.135350.111.

7. Rheinbay E, Nielsen MM, Abascal F, Tiao G, Hornshøj H, Hess JM, et al. Discovery and characterization of coding and non-coding driver mutations in more than 2,500 whole cancer genomes. bioRxiv. 2017. doi: 10.1101/237313.

8. Sabarinathan R, Pich O, Martincorena I, Rubio-Perez C, Juul M, Wala J, et al. The whole-genome panorama of cancer drivers. bioRxiv. 2017. doi: 10.1101/190330.

9. Hansen RS, Thomas S, Sandstrom R, Canfield TK, Thurman RE, Weaver M, et al. Sequencing newly replicated DNA reveals widespread plasticity in human replication timing. Proc Natl Acad Sci USA. 2010;107(1):139-44. doi: 10.1073/pnas.0912402107.

10. Avkin S, Livneh Z. Efficiency, specificity and DNA polymerase-dependence of translesion replication across the oxidative DNA lesion 8-oxoguanine in human cells. Mutation Research/Fundamental and Molecular Mechanisms of Mutagenesis. 2002;510(1–2):81-90. doi: 10.1016/S0027-5107(02)00254-3.

11. Liu B, Xue Q, Tang Y, Cao J, Guengerich FP, Zhang H. Mechanisms of mutagenesis: DNA replication in the presence of DNA damage. Mutation Research/Reviews in Mutation Research. 2016;768:53-67. doi: 10.1016/j.mrrev.2016.03.006.

12. Sale JE, Lehmann AR, Woodgate R. Y-family DNA polymerases and their role in tolerance of cellular DNA damage. Nature reviews Molecular cell biology. 2012;13(3):141-52. doi: 10.1038/nrm3289.

13. Yu X-J, Yang M-J, Zhou B, Wang G-Z, Huang Y-C, Wu L-C, et al. Characterization of Somatic Mutations in Air Pollution-Related Lung Cancer. EBioMedicine. 2015;2(6):583-90. doi: 10.1016/j.ebiom.2015.04.003.

14. Fujimoto A, Furuta M, Totoki Y, Tsunoda T, Kato M, Shiraishi Y, et al. Whole-genome mutational landscape and characterization of noncoding and structural mutations in liver cancer. Nature Genetics. 2016;48:500. doi: 10.1038/ng.3547

15. Totoki Y, Tatsuno K, Covington KR, Ueda H, Creighton CJ, Kato M, et al. Trans-ancestry mutational landscape of hepatocellular carcinoma genomes. Nature Genetics. 2014;46:1267. doi: 10.1038/ng.3126

16. Frank A, Seitz HK, Bartsch H, Frank N, Nair J. Immunohistochemical detection of 1,N^6-ethenodeoxyadenosine in nuclei of human liver affected by diseases predisposing to hepato-carcinogenesis. Carcinogenesis. 2004;25(6):1027-31. doi: 10.1093/carcin/bgh089.

17. Supek F, Lehner B. Clustered Mutation Signatures Reveal that Error-Prone DNA Repair Targets Mutations to Active Genes. Cell. 2017;170(3):534-47.e23. doi: 10.1016/j.cell.2017.07.003.

18. Schulze K, Imbeaud S, Letouzé E, Alexandrov LB, Calderaro J, Rebouissou S, et al. Exome sequencing of hepatocellular carcinomas identifies new mutational signatures and potential therapeutic targets. Nature Genetics. 2015;47:505. doi: 10.1038/ng.3252

19. Alexandrov LB, Nik-Zainal S, Wedge DC, Aparicio SAJR, Behjati S, Biankin AV, et al. Signatures of mutational processes in human cancer. Nature. 2013;500(7463):415-21. doi: 10.1038/nature12477.

20. Attaluri S, Bonala RR, Yang I-Y, Lukin MA, Wen Y, Grollman AP, et al. DNA adducts of aristolochic acid II: total synthesis and site-specific mutagenesis studies in mammalian cells. Nucleic Acids Research. 2010;38(1):339-52. doi: 10.1093/nar/gkp815.

21. Scelo G, Riazalhosseini Y, Greger L, Letourneau L, Gonzàlez-Porta M, Wozniak MB, et al. Variation in genomic landscape of clear cell renal cell carcinoma across Europe. Nature Communications. 2014;5:5135. doi: 10.1038/ncomms6135

22. Hashimoto K, Bonala R, Johnson F, Grollman AP, Moriya M. Y-family DNA polymerase-independent gap-filling translesion synthesis across aristolochic acid-derived adenine adducts in mouse cells. DNA Repair. 2016;46:55-60. doi: 10.1016/j.dnarep.2016.07.003.

23. Jeon Y, Bekiranov S, Karnani N, Kapranov P, Ghosh S, MacAlpine D, et al. Temporal profile of replication of human chromosomes. Proc Natl Acad Sci USA. 2005;102(18):6419-24. doi: 10.1073/pnas.0405088102.

24. Gnarra JR, Tory K, Weng Y, Schmidt L, Wei MH, Li H, et al. Mutations of the VHL tumour suppressor gene in renal carcinoma. Nature Genetics. 1994;7:85. doi: 10.1038/ng0594-85.

25. Varela I, Tarpey P, Raine K, Huang D, Ong CK, Stephens P, et al. Exome sequencing identifies frequent mutation of the SWI/SNF complex gene PBRM1 in renal carcinoma. Nature. 2011;469(7331):539-42. doi: 10.1038/nature09639.

26. Dalgliesh GL, Furge K, Greenman C, Chen L, Bignell G, Butler A, et al. Systematic sequencing of renal carcinoma reveals inactivation of histone modifying genes. Nature. 2010;463(7279):360-3. doi: 10.1038/nature08672.

27. Hoang ML, Chen CH, Sidorenko VS, He J, Dickman KG, Yun BH, et al. Mutational signature of aristolochic acid exposure as revealed by whole-exome sequencing. Science translational medicine. 2013;5(197):197ra02. doi: 10.1126/scitranslmed.3006200.

28. Hamblin TJ, Davis Z, Gardiner A, Oscier DG, Stevenson FK. Unmutated Ig V_H Genes Are Associated With a More Aggressive Form of Chronic Lymphocytic Leukemia. Blood. 1999;94(6):1848-54.

29. Chan K, Roberts SA, Klimczak LJ, Sterling JF, Saini N, Malc EP, et al. An APOBEC3A hypermutation signature is distinguishable from the signature of background mutagenesis by APOBEC3B in human cancers. Nature Genetics. 2015;47:1067. doi: 10.1038/ng.3378.

30. Dirican E, Akkiprik M, Özer A. Mutation distributions and clinical correlations of PIK3CA gene mutations in breast cancer. Tumor Biology. 2016;37(6):7033-45. doi: 10.1007/s13277-016-4924-2.

31. Nik-Zainal S, Alexandrov LB, Wedge DC, Van Loo P, Greenman CD, Raine K, et al. Mutational processes molding the genomes of 21 breast cancers. Cell. 2012;149(5):979-93. doi: 10.1016/j.cell.2012.04.024.

32. Nik-Zainal S, Wedge DC, Alexandrov LB, Petljak M, Butler AP, Bolli N, et al. Association of a germline copy number polymorphism of APOBEC3A and APOBEC3B with burden of putative APOBEC-dependent mutations in breast cancer. Nature Genetics. 2014;46:487. doi: 10.1038/ng.2955

33. Howard BD, Tessman I. Identification of the altered bases in mutated single-stranded DNA: II. In vivo mutagenesis by 5-bromodeoxyuridine and 2-aminopurine. Journal of Molecular Biology. 1964;9(2):364-71. doi:10.1016/S0022-2836(64)80213-8.

34. Rusch HP, Baumann CA. Tumor Production in Mice with Ultraviolet Irradiation. The American Journal of Cancer. 1939;35(1):55-62. doi: 10.1158/ajc.1939.55.

35. Brash DE. UV Signature Mutations. Photochemistry and Photobiology. 2015;91(1):15-26. doi: 10.1111/php.12377.

36. Shlien A, Campbell BB, de Borja R, Alexandrov LB, Merico D, Wedge D, et al. Combined hereditary and somatic mutations of replication error repair genes result in rapid onset of ultra-hypermutated cancers. Nature Genetics. 2015;47:257. doi: 10.1038/ng.3202.

37. Mertz TM, Sharma S, Chabes A, Shcherbakova PV. Colon cancer-associated mutator DNA polymerase δ variant causes expansion of dNTP pools increasing its own infidelity. Proceedings of the National Academy of Sciences. 2015;112(19):E2467-E76. doi: 10.1073/pnas.1422934112.

38. Supek F, Lehner B. Differential DNA mismatch repair underlies mutation rate variation across the human genome. Nature. 2015;521(7550):81-4. doi: 10.1038/nature14173.

39. Ferreira AM, Tuominen I, Dijk-Bos K, Sanjabi B, Sluis T, Zee AG, et al. High Frequency of RPL22 Mutations in Microsatellite-Unstable Colorectal and Endometrial Tumors. Human Mutation. 2014;35(12):1442-5. doi: doi:10.1002/humu.22686.

40. Maruvka YE, Mouw KW, Karlic R, Parasuraman P, Kamburov A, Polak P, et al. Analysis of somatic microsatellite indels identifies driver events in human tumors. Nature Biotechnology. 2017;35:951. doi: 10.1038/nbt.3966.

41. Dulak AM, Stojanov P, Peng S, Lawrence MS, Fox C, Stewart C, et al. Exome and whole genome sequencing of esophageal adenocarcinoma identifies recurrent driver events and mutational complexity. Nature genetics. 2013;45(5):478-86. doi: 10.1038/ng.2591.

42. Secrier M, Li X, de Silva N, Eldridge MD, Contino G, Bornschein J, et al. Mutational signatures in esophageal adenocarcinoma define etiologically distinct subgroups with therapeutic relevance. Nature Genetics. 2016;48:1131. doi: 10.1038/ng.3659.

43. Dvorak K, Payne CM, Chavarria M, Ramsey L, Dvorakova B, Bernstein H, et al. Bile acids in combination with low pH induce oxidative stress and oxidative DNA damage:

relevance to the pathogenesis of Barrett's oesophagus. Gut. 2007;56(6):763-71. doi: 10.1136/gut.2006.103697.

44. Tomkova M, Tomek J, Kriaucionis S, Schuster-Böckler B. Mutational signature distribution varies with DNA replication timing and strand asymmetry. Genome Biology. 2018;19(1):129. doi: 10.1186/s13059-018-1509-y.

45. Kamiya H. Mutations Induced by Oxidized DNA Precursors and Their Prevention by Nucleotide Pool Sanitization Enzymes. Genes and Environment. 2007;29(4):133-40. doi: 10.3123/jemsge.29.133.

46. Stamatoyannopoulos JA, Adzhubei I, Thurman RE, Kryukov GV, Mirkin SM, Sunyaev SR. Human mutation rate associated with DNA replication timing. Nature Genetics. 2009;41:393. doi: 10.1038/ng.363.

47. Seplyarskiy VB, Bazykin GA, Soldatov RA. Polymerase ζ Activity Is Linked to Replication Timing in Humans: Evidence from Mutational Signatures. Molecular Biology and Evolution. 2015;32(12):3158-72. doi: 10.1093/molbev/msv184.

48. Zhang W, Hong R, Xue L, Ou Y, Liu X, Zhao Z, et al. Piccolo mediates EGFR signaling and acts as a prognostic biomarker in esophageal squamous cell carcinoma. Oncogene. 2017;36(27):3890-902. doi: 10.1038/onc.2017.15.

49. Lee SH, Je EM, Yoo NJ, Lee SH. HMCN1, a cell polarity-related gene, is somatically mutated in gastric and colorectal cancers. Pathology & Oncology Research. 2015;21(3):847-8. doi: 10.1007/s12253-014-9809-3.

50. Chong IY, Cunningham D, Barber LJ, Campbell J, Chen L, Kozarewa I, et al. The genomic landscape of oesophagogastric junctional adenocarcinoma. The Journal of Pathology. 2013;231(3):301-10. doi: 10.1002/path.4247.

51. Chang J, Tan W, Ling Z, Xi R, Shao M, Chen M, et al. Genomic analysis of oesophageal squamous-cell carcinoma identifies alcohol drinking-related mutation signature and genomic alterations. Nature Communications. 2017;8:15290. doi: 10.1038/ncomms15290

52. Zanotti KJ, Gearhart PJ. Antibody diversification caused by disrupted mismatch repair and promiscuous DNA polymerases. DNA Repair. 2016;38:110-6. doi: dx.doi.org/10.1016/j.dnarep.2015.11.011.

53. Williams NL, Wang P, Wu J, Wang Y. In Vitro Lesion Bypass Studies of O^4-Alkylthymidines with Human DNA Polymerase η. Chemical Research in Toxicology. 2016;29(4):669-75. doi: 10.1021/acs.chemrestox.5b00509.

54. Tarte K, Jourdan M, Veyrune JL, Berberich I, Fiol G, Redal N, et al. The Bcl-2 family member Bfl-1/A1 is strongly repressed in normal and malignant plasma cells but is a potent anti-apoptotic factor for myeloma cells. British Journal of Haematology. 2004;125(3):373-82. doi: 10.1111/j.1365-2141.2004.04908.x.

55. Ding L-W, Sun Q-Y, Tan K-T, Chien W, Thippeswamy AM, Eng Juh Yeoh A, et al. Mutational Landscape of Pediatric Acute Lymphoblastic Leukemia. Cancer Research. 2017;77(2):390-400. doi: 10.1158/0008-5472.can-16-1303.

56. Notta F, Chan-Seng-Yue M, Lemire M, Li Y, Wilson GW, Connor AA, et al. A renewed model of pancreatic cancer evolution based on genomic rearrangement patterns. Nature. 2016;538(7625):378-82. doi: 10.1038/nature19823.
