# Supplementary material for: Recurrent somatic mutations reveal new insights into consequences of mutagenic processes in cancer
Source: PLoS Comput Biol. 2019 Nov 25;15(11):e1007496. doi: 10.1371/journal.pcbi.1007496 (PMC6901237; doi:10.1371/journal.pcbi.1007496)
Supplement: S4 Text — (PDF) [file pcbi.1007496.s010.pdf]

Smoking history and related mutational subtypes

There are 523 samples for which the smoking history of the donor was available. In terms of percentages of C>A SSMs and 1 bp C/G deletions, the donors with Lung-AdenoCA or Lung-SCC, who were smoking at diagnosis or who had quit at most 15 years before, clearly stand out from the rest along with three samples of Head-SCC (Fig A). In Fig B, we show tumour-type specific boxplots of the percentages for the aforementioned mutation types stratified by the different categories of smoking history. Note that cohorts did not always provide the same level of detail in this respect. A lifelong non-smoker is defined as someone that has smoked fewer than 100 cigarettes in total. For each tumour type listed in Table A we compare the percentage of 1 bp C/G deletions and the percentage of C>A SSMs across the different smoking history backgrounds. For this we combine, where applicable, the lifelong non-smokers with reformed smokers that quit >15 years before diagnosis, and the current smokers with reformed smokers that quit ≤ 15 years before diagnosis. The p-values given in Table A are calculated by Wilcoxon rank-sum tests and further corrected for multiple testing by FDR-adjustment using the Benjamini-Hochberg procedure. Lung-SCC does not show a statistically significant difference for either of the mutation types between the two smoking strata, which is partly explicable by the fact that the only reported never smoker shows proportions well in the range that is typically observed for smokers and likely represents a case of misclassification or strong second-hand smoking. Note that there is also limited statistical power for several other comparisons.

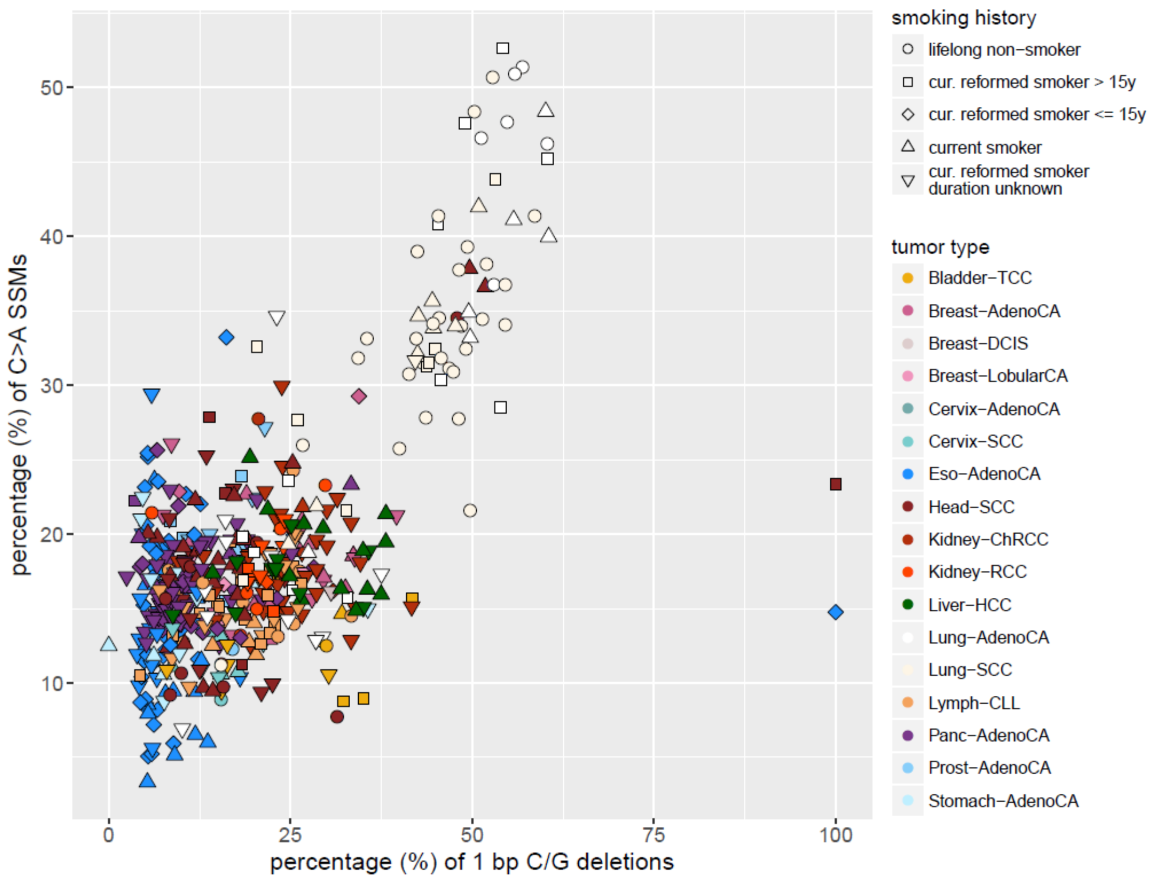

Fig A. The percentage of C>A SSMs versus 1 bp C/G deletions in context of smoking history.

The colours correspond to the tumour type and the shapes represent the different categories of smoking history.

I

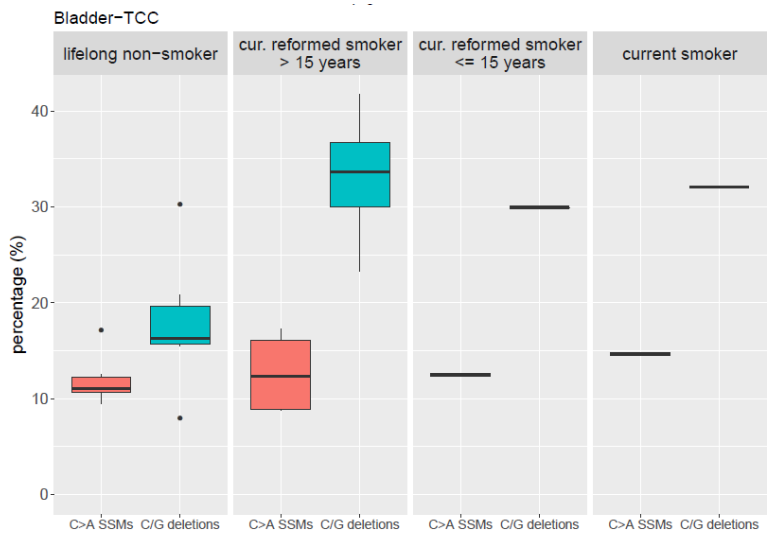

(continues below)

II

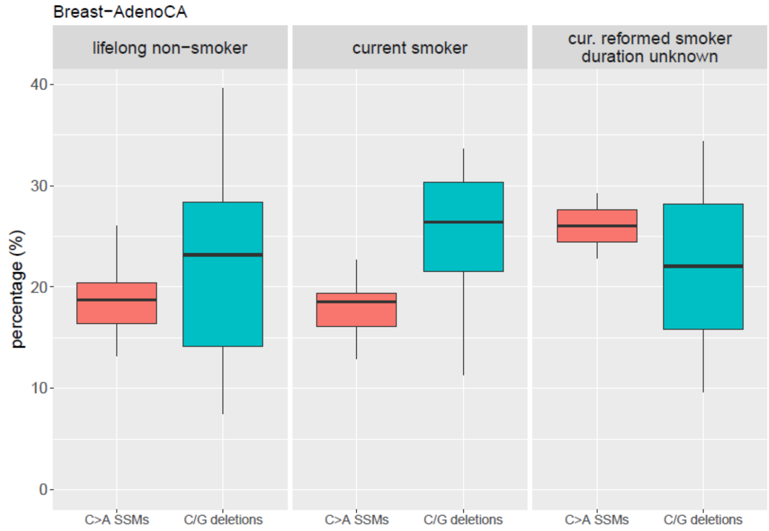

III

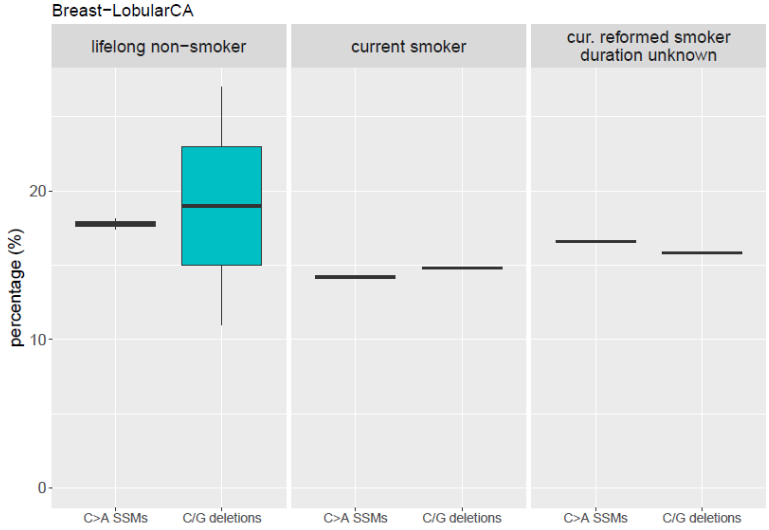

IV

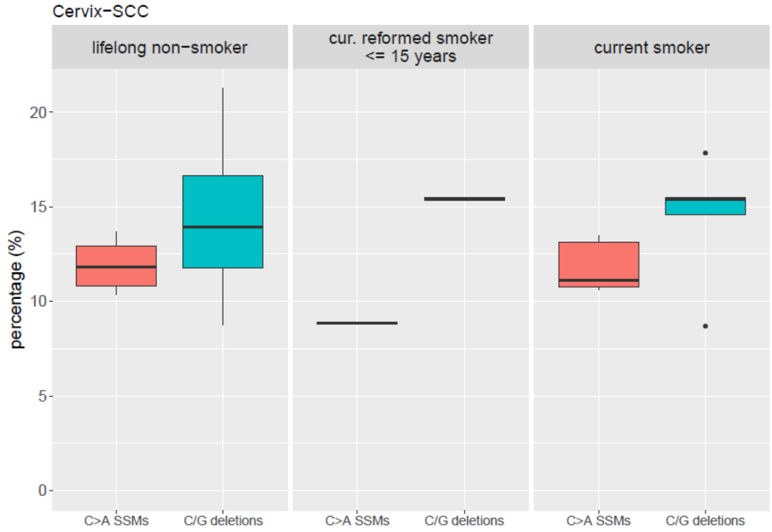

(continues below)

V

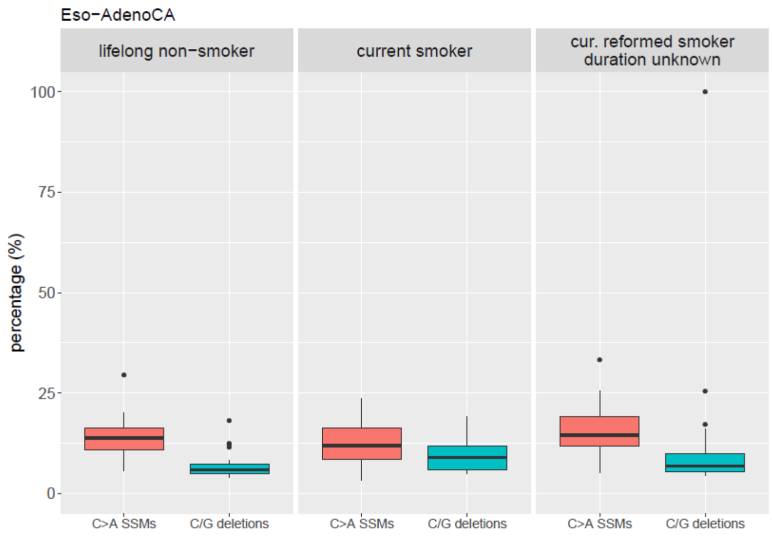

VI

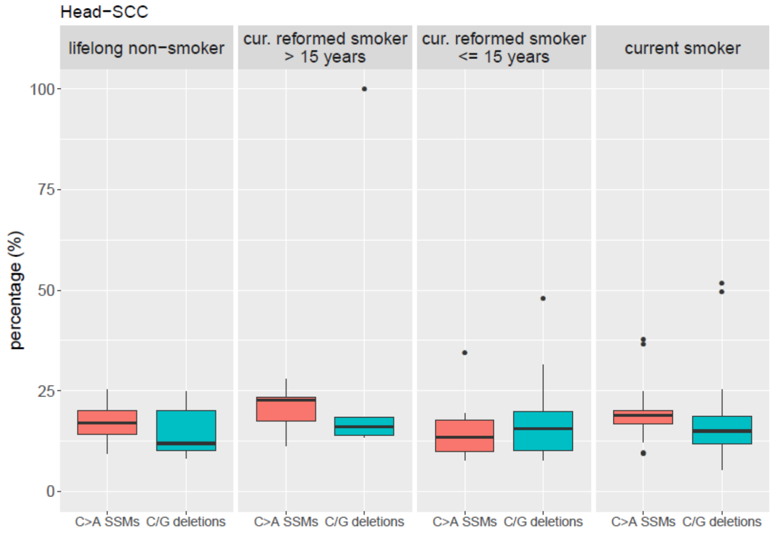

VII

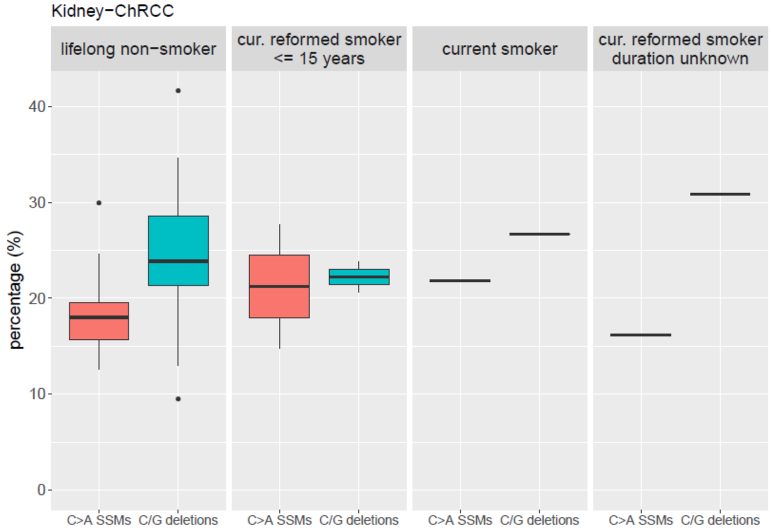

(continues below)

VIII

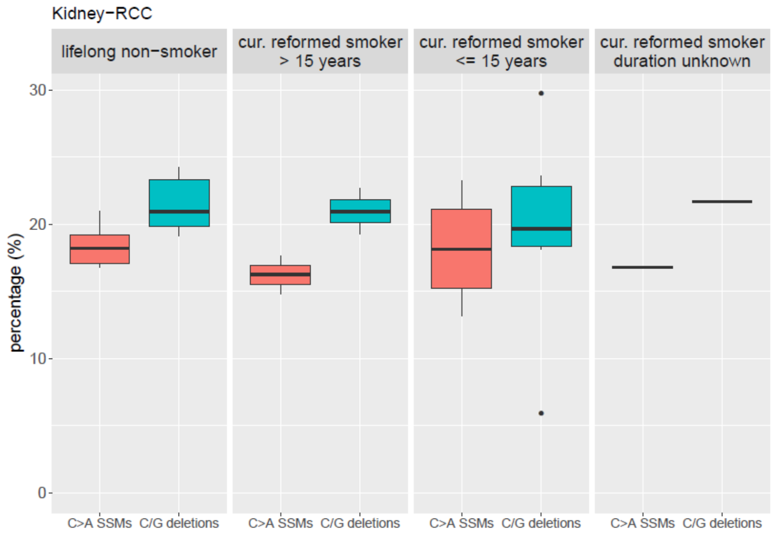

IX

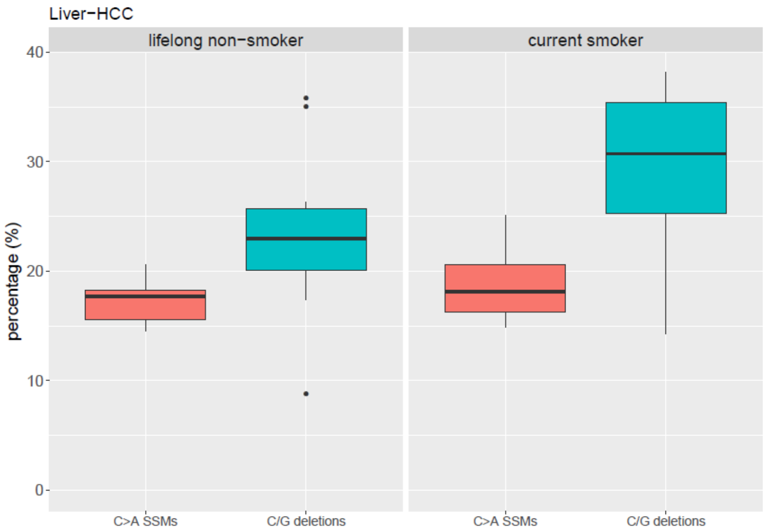

X

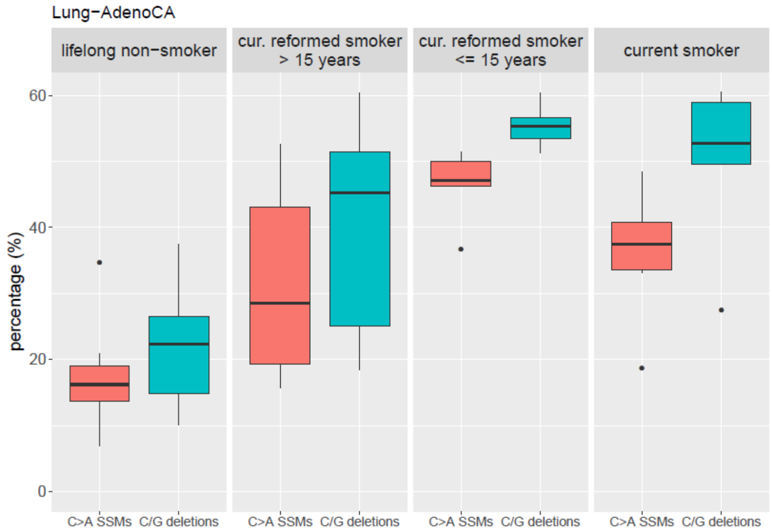

(continues below)

XI

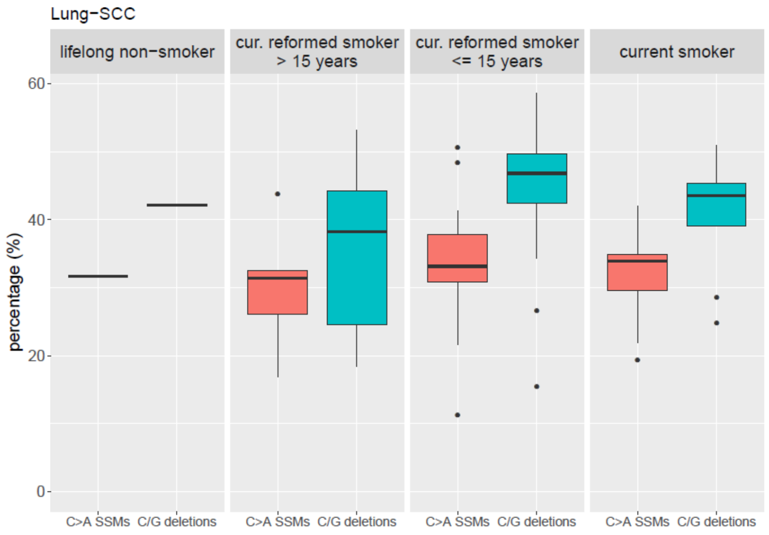

XII

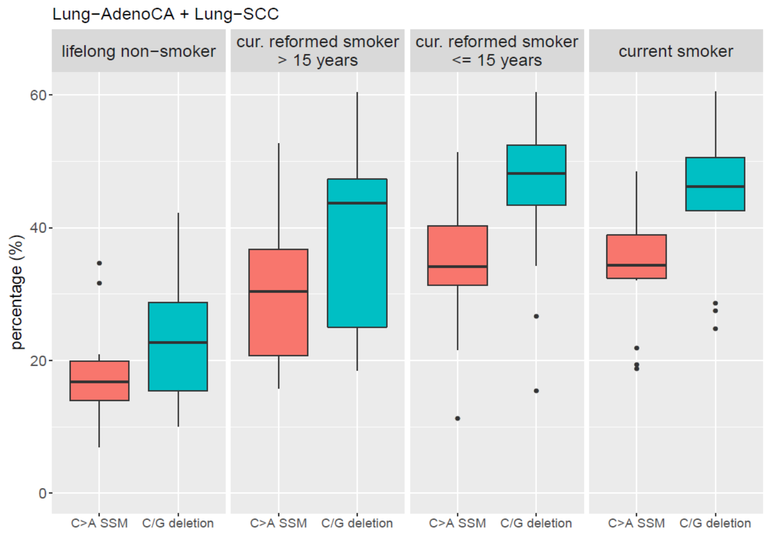

XIII

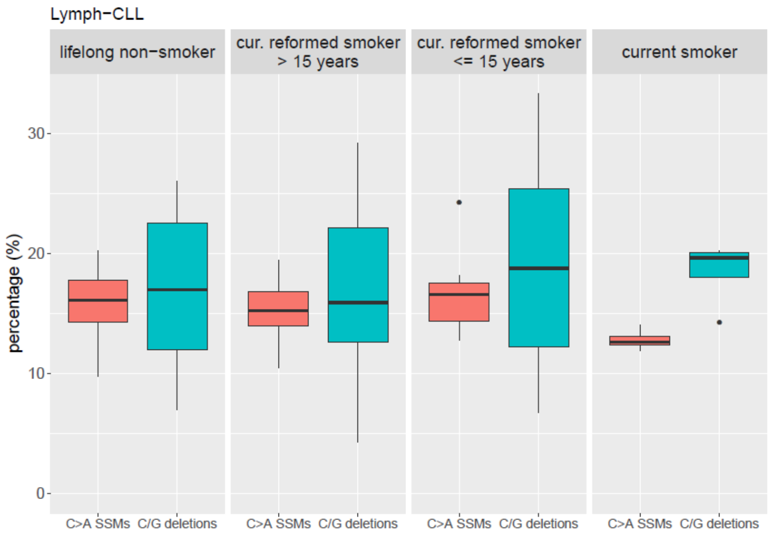

(continues below)

XIV

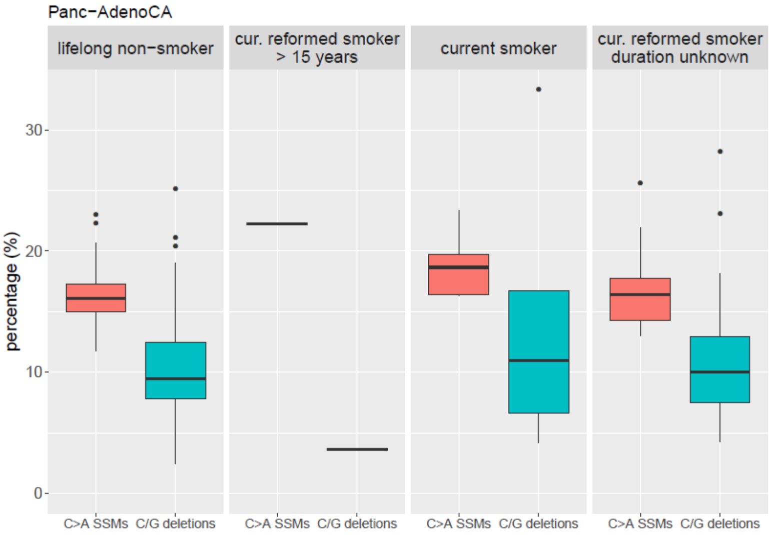

XV

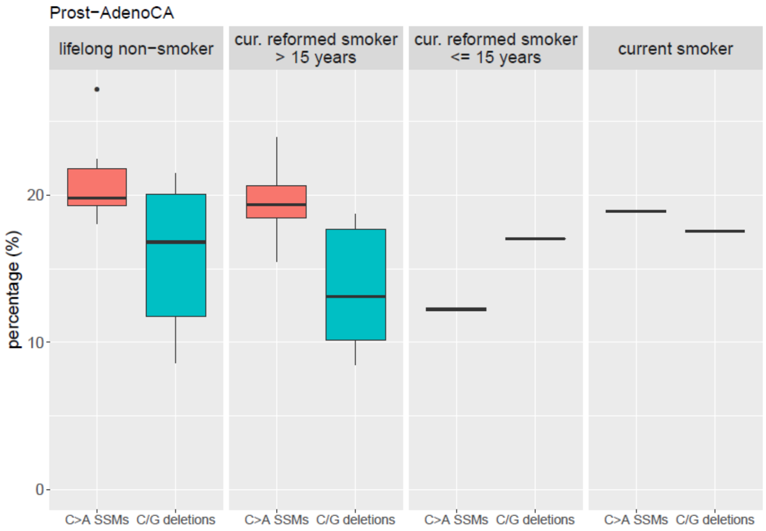

XVI

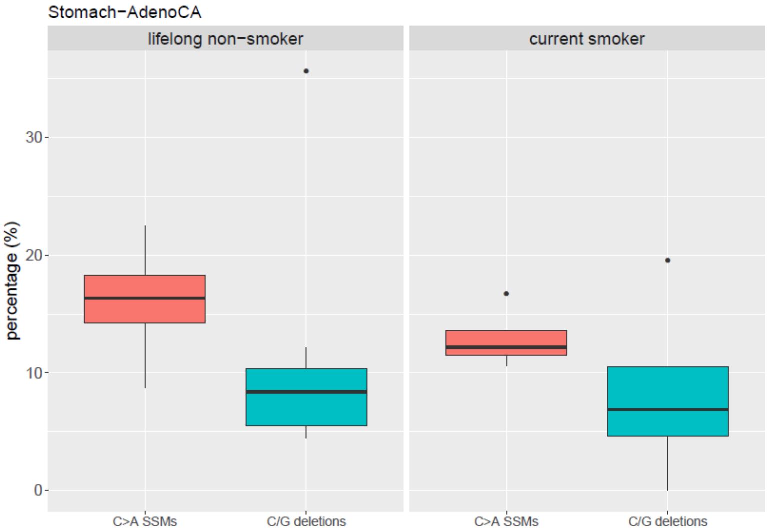

Fig B. Tumour-type specific boxplots of the percentage of C>A SSMs and 1 bp C/G deletions per smoking history category.

Table A. Smoking history versus percentage of 1 bp C/G deletions and C>A SSMs.

Tumour type	num. lifelong non-smokers + current reformed smokers > 15y	num. of current smokers + current reformed smokers ≤ 15y	adj. p-value percentage of 1 bp C/G del.	adj. p-value percentage of C>A SSMs
Breast-AdenoCA	12	12	0.72	0.88
Cervix-SCC	4	6	0.88	0.88
Eso-AdenoCA	20	20	0.21	0.68
Head-SCC	19	35	0.91	0.91
Kidney-ChRCC	34	3	0.88	0.72
Kidney-RCC	8	6	0.72	0.95
Liver-HCC	11	14	0.21	0.68
Lung-AdenoCA	22	12	5.5e-4	0.0034
Lung-SCC	9	37	0.21	0.39
Lung-AdenoCA+SCC	31	49	2.7e-4	5.5e-4
Lymph-CLL	40	16	0.88	0.88
Panc-AdenoCA	60	5	0.88	0.21
Stomach-AdenoCA	8	4	0.88	0.56

Shown are the tumour types for which we have more than two donors from each of the two groups defined in the second and third column. We also added the combination of the samples of Lung-AdenoCA and Lung-SCC. If the number of years since smoking cessation is indicated to be unknown, then the sample is not included in the comparison. All p-values are based on Wilcoxon rank-sum tests and corrected for multiple testing by controlling the false-discovery rate using the Benjamini-Hochberg procedure.
