# Supplementary material for: Recurrent somatic mutations reveal new insights into consequences of mutagenic processes in cancer
Source: PLoS Comput Biol. 2019 Nov 25;15(11):e1007496. doi: 10.1371/journal.pcbi.1007496 (PMC6901237; doi:10.1371/journal.pcbi.1007496)
Supplement: S1 File — (PDF) [file pcbi.1007496.s011.pdf]

Characteristic plots summarising each of the 42 features

Each cancer genome is described by 42 features (Table A). We display graphical representations for each feature (Fig A to I) and show absolute numbers in most cases on the y-axis (where applicable). We refer to a value as being an outlier if it is above the third quartile plus 1.5 times the interquartile range (Q3+1.5xIQR). We describe the main observations below the individual plots.

Table A. Overview of the 42 mutational features describing each cancer genome.

General features	mutational burden	number of	SSMs
			SIMs
	SIM vs. SSM ratio	% of mutations of type SIM	
	distribution of SSMs across the 6 subtypes	percentage of	C>A SSMs
			C>G SSMs
			C>T SSMs
			T>A SSMs
			T>C SSMs
			T>G SSMs
	distribution of 1 bp SIMs across the 4 subtypes	percentage of	A/T deletions
			C/G deletions
			A/T insertions
			C/G insertions
	homopolymer context of 1 bp SIMs	% of A/T deletions	no
			short
			midsize
			long
		% of C/G deletions	no
			short
			midsize
			long
		% of A/T insertions	no
			short
			midsize
			long
		% of C/G insertions	no
			short
			midsize
			long
Recurrence features	overall level of recurrence	% of recurrent	SSMs
			SIMs
	recurrent SIM vs. SSM ratio	% of recurrent mutations of type SIM	
	level of recurrence per SSM subtype	% of recurrent	C>A SSMs
			C>G SSMs
			C>T SSMs
			T>A SSMs
			T>C SSMs
			T>G SSMs
	level of recurrence per SIM subtype (1 bp)	% of recurrent	A/T deletions
			C/G deletions
			A/T insertions
			C/G insertions

Overview of the 29 general features and the 13 features related to recurrence that are used as input for the PCA. For deletions a 'no homopolymer context' means that the base next to the one that is deleted is not of the same type. For insertions a 'no homopolymer context' refers to a base that is inserted 5' to a base of a different type or a single base of the same type. Note that we do not have to consider the preceding bases as all SIM calls were left aligned. A short homopolymer context is defined as a 2-4 bp mononucleotide repeat of the same base as the 1 bp SIM, midsize is 5-7 bp in length and long ≥ 8 bp.

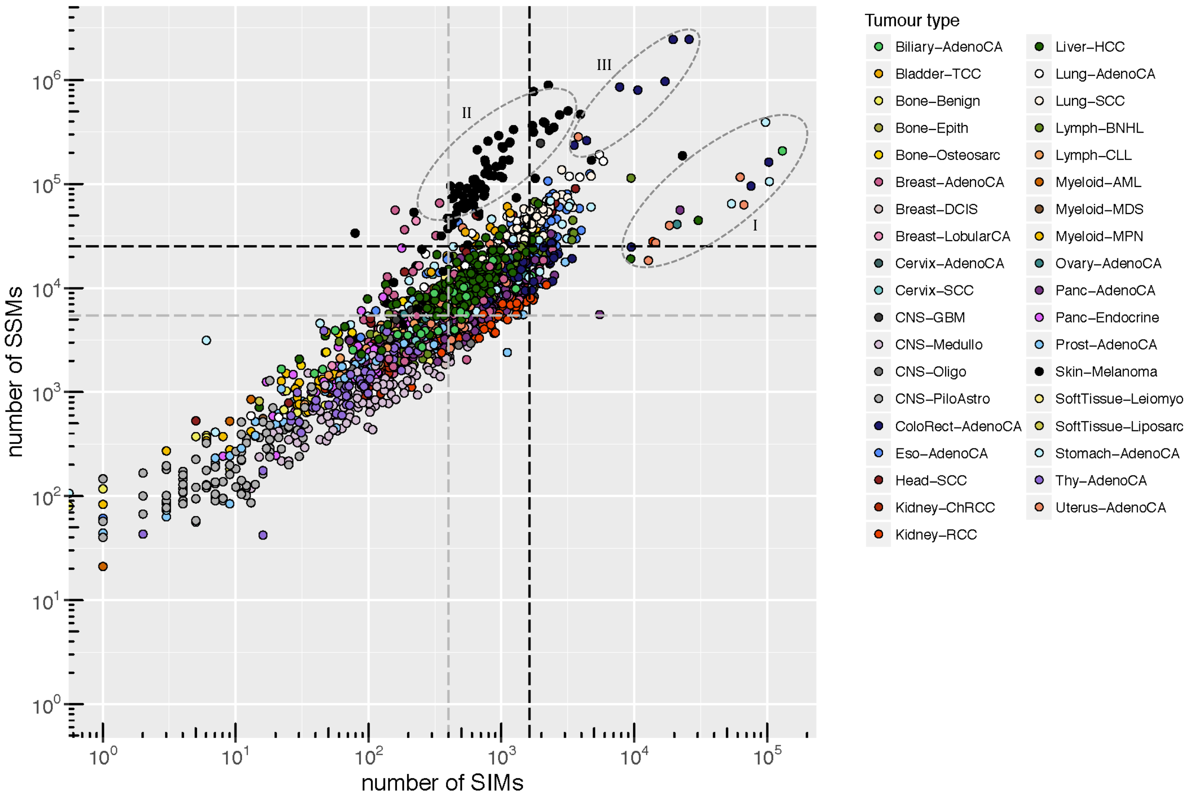

Fig A. Overall mutational burden in terms of SIMs and SSMs per sample.

The two grey lines indicate the median number of SIMs and SSMs, respectively, across the entire cohort. The black lines indicate the Q3+1.5xIQR. For SIMs there are 184 outliers, the highest number of samples are from Eso-AdenoCA (22.3%), followed by ColoRect-AdenoCA (13.6%) and Lung-SCC (13.0%). For Eso-AdenoCA this corresponds to 42.3% of the samples, 48.1% for ColoRect-AdenoCA and 51.1% for the Lung-SCC. Highlighted in the plot (I) are samples with a high mutational load, which have a particularly high proportion of SIMs. For SSMs there are 255 outliers of which the highest number of samples are from Skin-Melanoma (29.8%), followed by Eso-AdenoCA (16.1%) and Lung-SCC (14.9%). This corresponds for Skin-Melanoma to 71.0% of the samples, 42.3% for Eso-AdenoCA and 80.9% for Lung-SCC. The outliers of Skin-Melanoma (II) are above the bulk of the samples by having a higher proportion of SSMs. There are 122 samples that are outliers in terms of SIMs and SSMs of which the highest number of samples are from Eso-AdenoCA (23.0%), followed by Lung-SCC (19.7%) and Skin-Melanoma (11.5%). The eight samples highlighted in the plot (III) have a very high number of SSMs, but a lower proportion of SIMs compared to the samples highlighted in I.

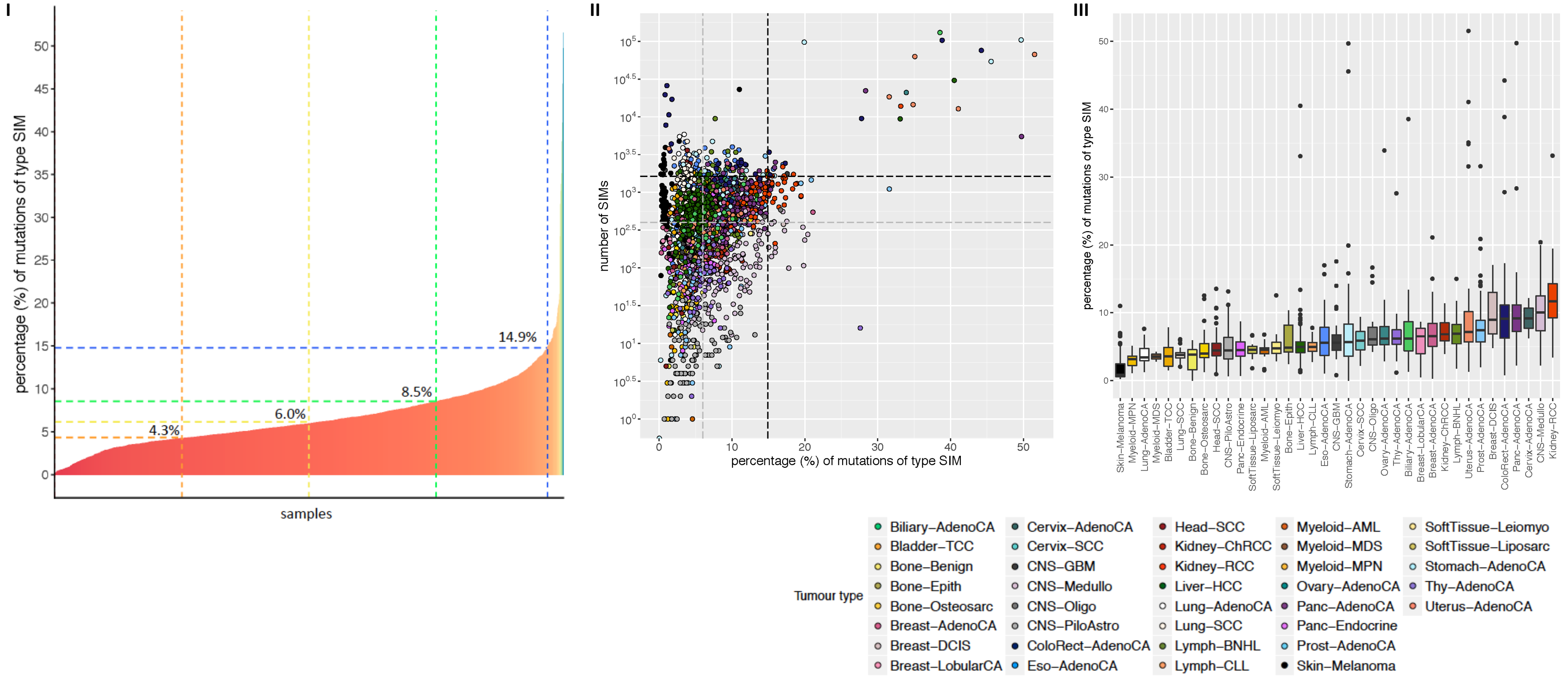

Fig B. The percentage of mutations of type SIM per sample.

(I) The percentage of mutations of type SIM is, with the exception of one Uterus-AdenoCA sample, below 50%. The yellow line indicates the median percentage of mutations of type SIM across the dataset (6.0%). To the right of the vertical yellow line the samples have a percentage above the median. The orange (4.3%) and green (8.5%) lines indicate the first and third quartile, respectively. The Q1-1.5xIQR is equal to 0% and is not shown. The blue line indicates the Q3+1.5xIQR (14.9%) to the right of which samples are outliers. (II) The percentage of mutations of type SIM versus the number of SIMs per sample. The grey lines indicate the medians and the black lines indicate the Q3+1.5xIQR. There are 32 samples from 11 different tumour types that are outliers in terms of percentage and absolute number. This includes 6 samples of ColoRect-AdenoCA and 5 samples each of Uterus-AdenoCA and Kidney-RCC. (III) Boxplots representing the percentage of mutations of type SIM show considerable variability among tumour types. They are ordered according to the median.

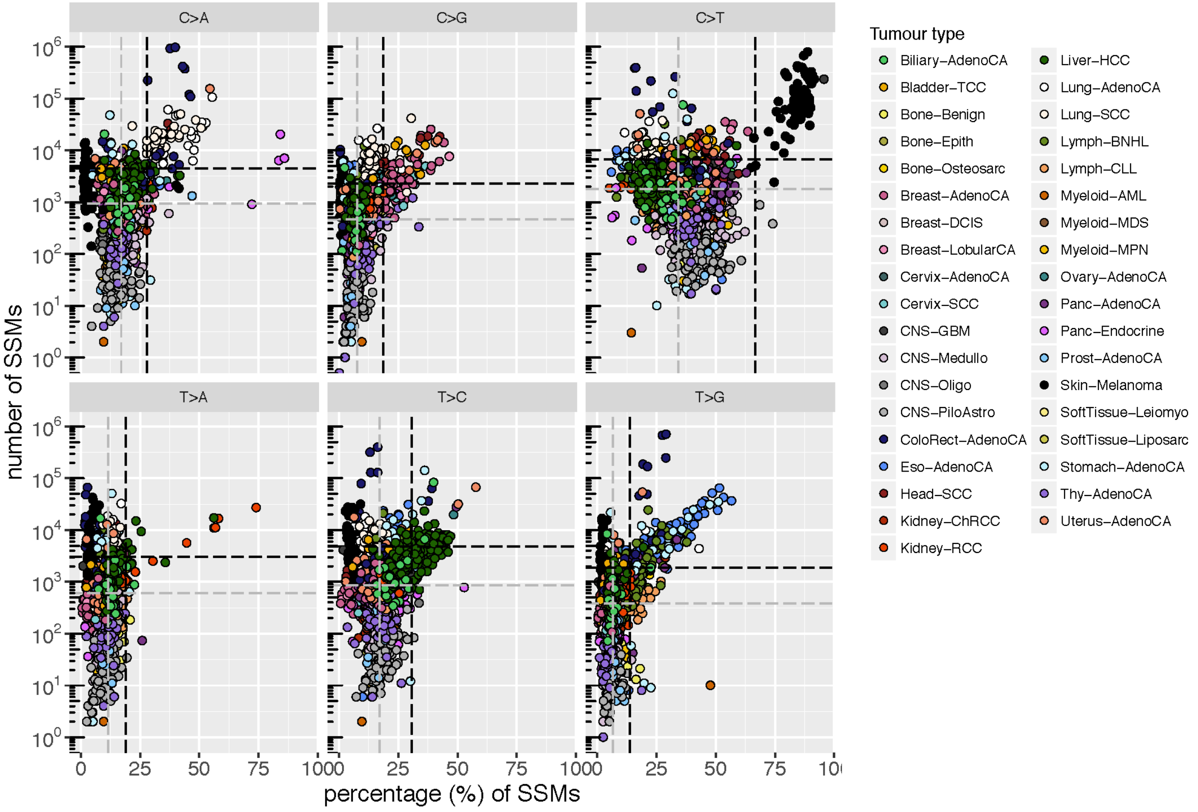

Fig C. Absolute and relative number of SSMs across the six subtypes.

Shown for each sample are the percentage of SSMs of the indicated subtype and the corresponding absolute number. Per sample the six percentages sum up to 100%. The grey lines indicate the medians and the black lines the Q3+1.5xIQR based on, for the vertical lines, the percentage of SSMs of the particular subtype, and for the horizontal lines, the absolute numbers. The median percentage across the entire dataset is highest for C>T (34.2%), followed by C>A and T>C (both 17.0%), T>A (11.5%), C>G (7.7%) and T>G (6.6%). For each of the six subtypes there are a number of samples that are outliers in terms of percentage and absolute number. For the C>A SSMs there are 78 outliers from eight different tumour types of which the highest number of samples are from Lung-SCC (46.2%), followed by Lung-AdenoCA (24.4%) and ColoRect-AdenoCA (16.7%). This corresponds for Lung-SCC to 76.6% of the samples, 51.4% for Lung-AdenoCA and 25% for ColoRect-AdenoCA. There are 84 outliers for C>G SSMs from 11 different tumour types of which the highest number of samples are from Breast-AdenoCA (32.1%), followed by Bladder-SCC and Head-SCC (17.9% for both). This corresponds for Breast-AdenoCA to 13.8% of the samples, 65.2% for Bladder-SCC and 26.8% for Head-SCC. For the C>T SSMs there are 80 outliers of which 79 are from Skin-Melanoma and 1 from CNS-GBM. For Skin-Melanoma this corresponds to 73.8% of the samples. For T>A SSMs there are only 11 outliers of which 6 are from Liver-HCC and 5 from Kidney-RCC. For the T>C SSMs there are 85 outliers from 7 different tumour types of which 87.1% are from Liver-HCC. This corresponds to 23.6% of the total number of Liver-HCC samples. Finally, for T>G SSMs there are 146 outliers from 13 different tumour types of which the highest number of samples are from Eso-AdenoCA (48.6%), followed by Lymph-BNHL (19.9%) and Stomach-AdenoCA (13.7%). This corresponds for Eso-AdenoCA to 73.2% of the samples, 27.1% for Lymph-BNHL and 29.4% for Stomach-AdenoCA.

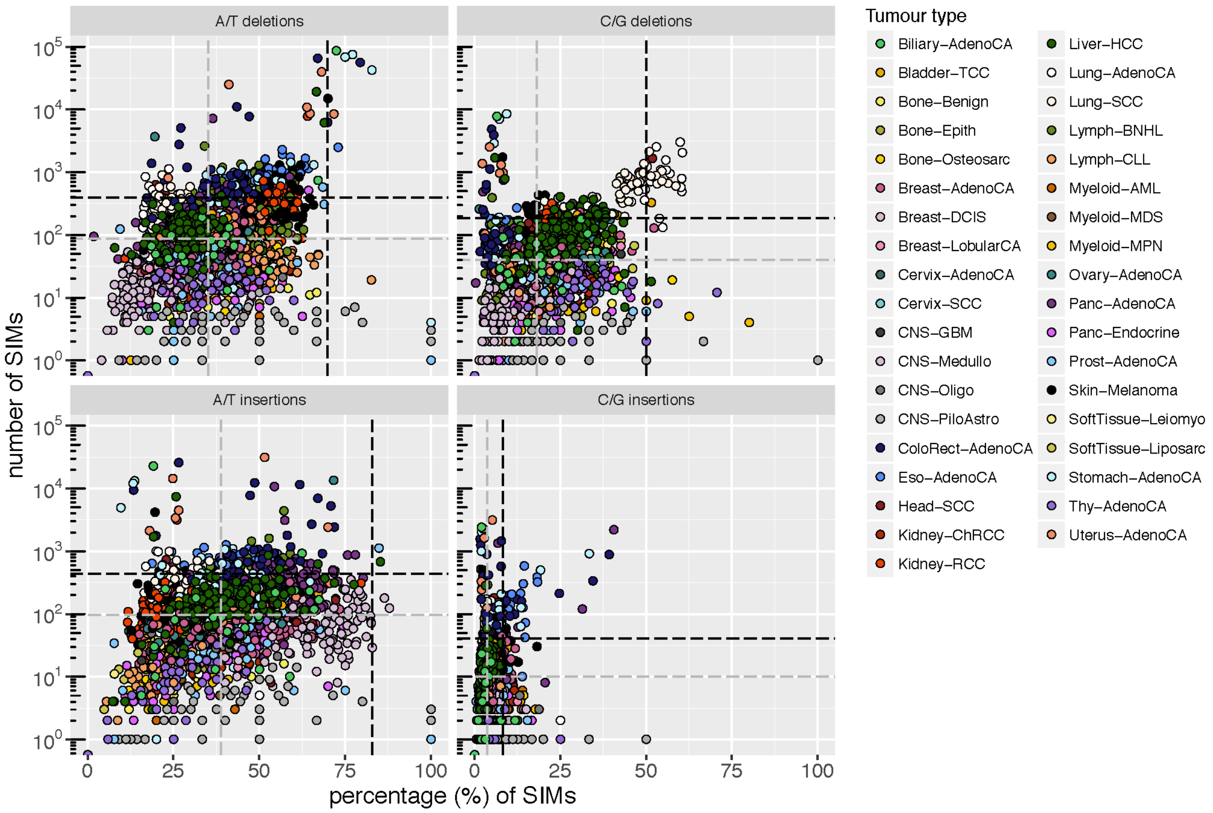

Fig D. Absolute and relative number of 1 bp SIMs across the four subtypes.

Shown for each sample are the percentage of SIMs of the indicated subtype and the corresponding absolute number. Per sample the four percentages sum up to 100%. The grey lines indicate the medians and the black lines the Q3+1.5xIQR based on, for the vertical lines, the percentage of SIMs of the particular subtype, and for the horizontal lines, the absolute numbers. The median percentage across the entire cohort is highest for 1 bp A/T insertions (38.8%), followed by 1 bp A/T deletions (35.2%), 1 bp C/G deletions (18.2%), and 1 bp C/G insertions (3.7%). Due to the large range of percentages for the 1 bp A/T deletions and insertions there are only 7 and 2 outliers, respectively, in terms of percentage and absolute number. There are 405 samples for which at least 50% of the 1 bp SIMs are A/T deletions. For three tumour types this holds for half or more of their samples: Kidney-RCC (71.3%), Skin-Melanoma (51.4%) and Lymph-CLL (50.0%). For 1 bp A/T insertions there are 630 samples for which this subtype makes up at least 50% of their 1 bp SIMs. For four tumour types this holds for half or more of their samples: Cervix-AdenoCA (100%, 2 samples), CNS-Medullo (87.2%), Cervix-SCC (72.2%) and Panc-AdenoCA (69.0%). For the 1 bp C/G deletions there are 23 outliers in terms of percentage and absolute number of which 11 are from Lung-AdenoCA, 10 from Lung-SCC, 1 each from Blader-TCC and Head-SCC. Interestingly, for these outliers 1 bp C/G deletions are the majority of their 1 bp SIMs. For 1 bp C/G insertions there are 39 outliers of which 16 are from Eso-AdenoCA, 10 from ColoRect-AdenoCA, 6 from Stomach-AdenoCA, 4 from Panc-AdenoCA and 3 from Skin-Melanoma.

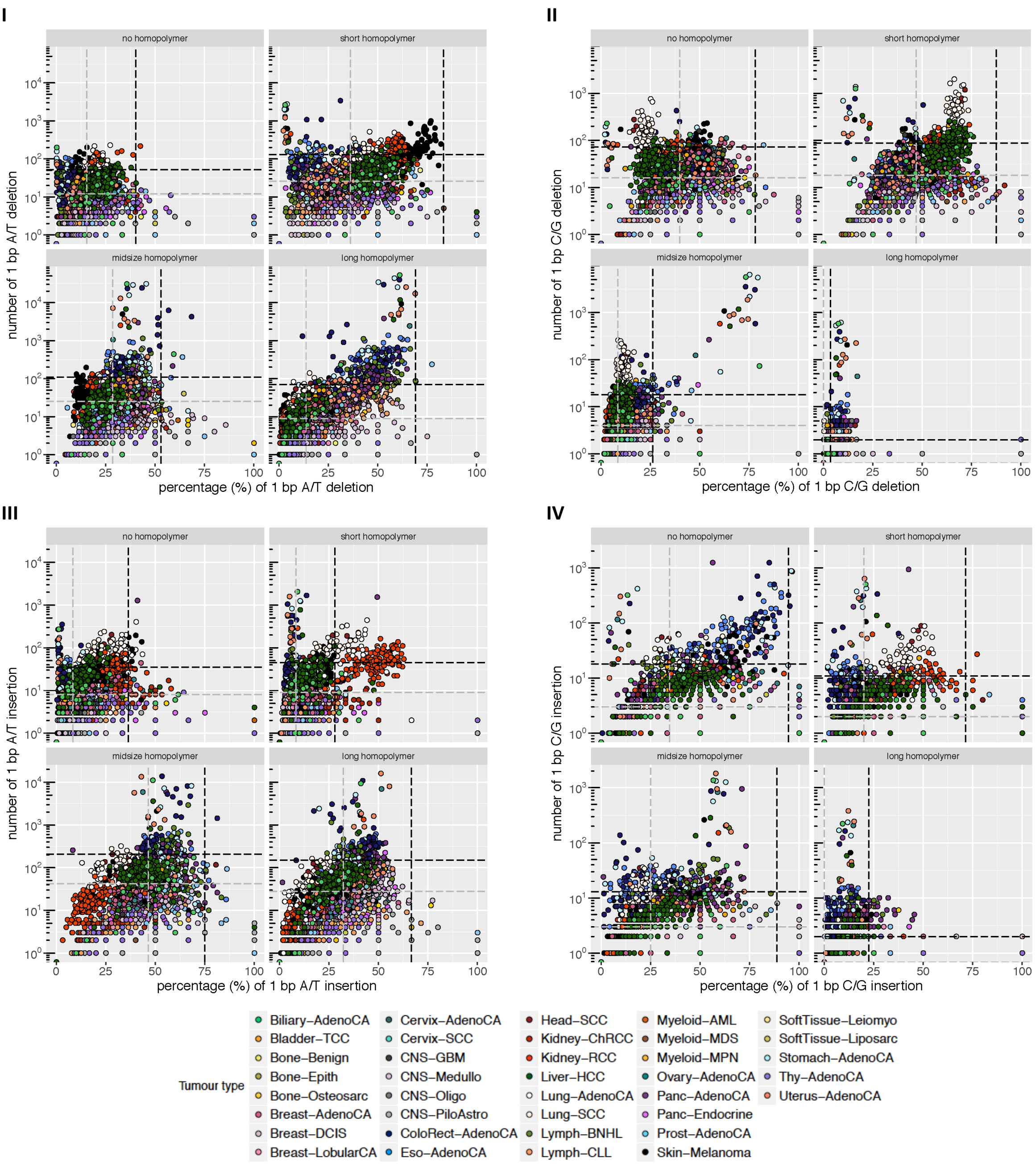

Fig E. Homopolymer context of 1 bp SIMs.

For each of the four SIM subtypes we computed per sample the percentage of 1 bp SIMs in the four homopolymer contexts (see **Main Text**). The grey lines indicate the medians and the black lines the Q3+1.5xIQR based on, for the vertical lines, the percentage of SIMs in the particular homopolymer context, and for the horizontal lines, the absolute numbers. For most contexts, there are few outliers (12 or less) in terms of percentage and absolute number. Exceptions are the midsize and long homopolymer context for 1 bp C/G deletions (33 and 161 cases, respectively), short homopolymer context for 1 bp A/T insertions (102 cases) and long homopolymer context for 1 bp C/G insertions (40 cases). For a number of samples more than 50% of a particular SIM subtype is in one of the four homopolymer contexts. These are for (I) 1 bp A/T deletions: 13 samples in no, 487 samples in a short, 77 samples in a midsize, and 174 samples in a long homopolymer context; (II) 1 bp C/G deletions: 507 samples in no, 1,013 samples in a short, 22 samples in a midsize and 3 samples in a long homopolymer context; (III) 1 bp A/T insertions: 18 samples in no, 66 samples in a short, 852 samples in a midsize and 100 samples in a long homopolymer context; (IV) 1 bp C/G insertions: 608 samples in no, 165 samples in a short, 321 samples in a midsize and 9 samples in a long homopolymer context.

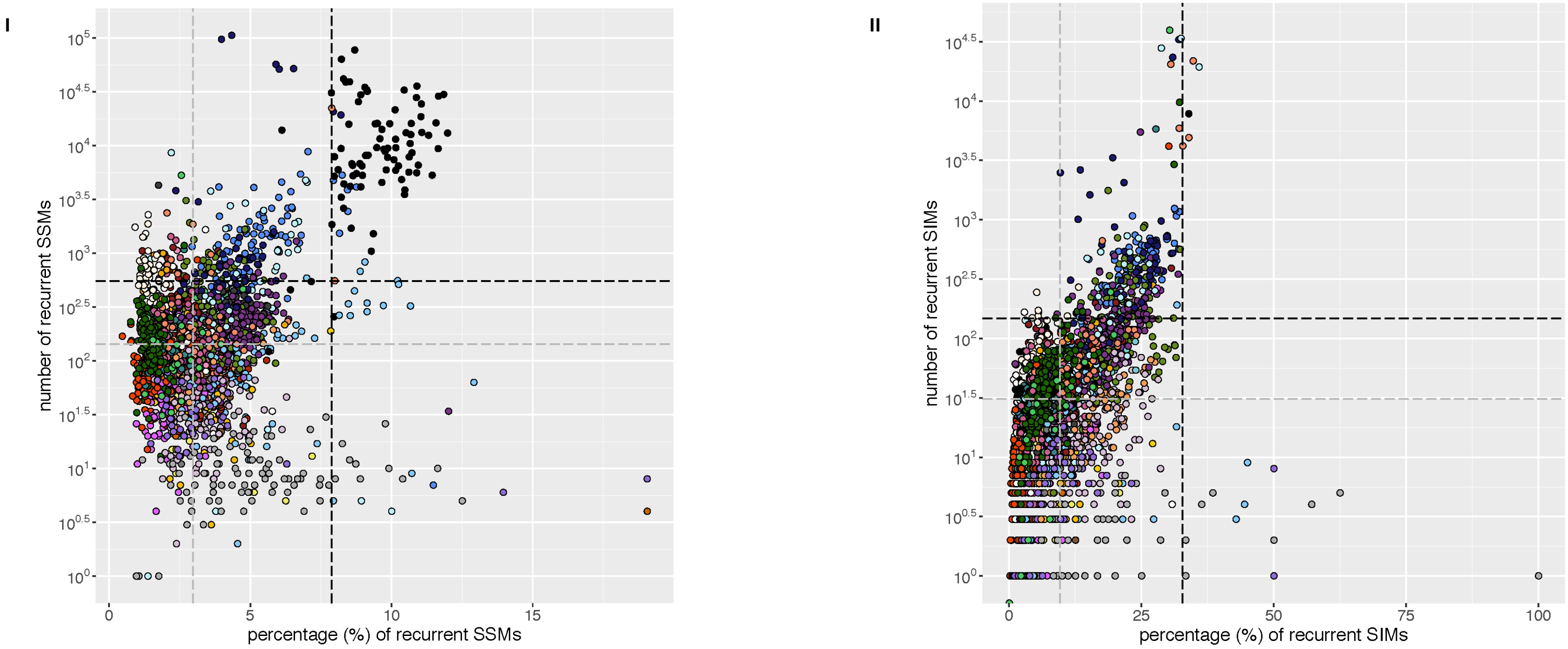

Fig F. Overall level of recurrence in terms of SSMs and SIMs per sample.

(I) The percentage versus the absolute number of recurrent SSMs. The grey lines indicate the medians and the black lines the Q3+1.5xIQR based on, for the vertical lines, the percentage of recurrent SSMs and, for the horizontal lines, the absolute numbers. There are 89 samples that are outliers in both relative and absolute terms of which 77 are Skin-Melanoma samples. Only based on absolute number, there are 333 outliers of which 24.6% are Skin-Melanoma samples, followed by 22.2% Eso-AdenoCA samples. Lung-SCC samples have a high absolute number of recurrent SSMs, but the percentage that is recurrent is below the median. (II) The percentage versus the absolute number of recurrent SIMs. The grey lines indicate the medians and the black lines the Q3+1.5xIQR based on, for the vertical lines, the percentage of recurrent SIMs and, for the horizontal lines, the absolute numbers. There are only 4 outliers for both measurements and 295 if we instead base it only on absolute number of recurrent SIMs of which the largest percentage are Eso-AdenoCA samples (25.4%), followed by Panc-AdenoCA (19.0%) and ColoRect-AdenoCA (15.9%). Noticeable is the group of eight samples from four different tumour types, each of which has over 19,000 recurrent SIMs and at least 28.7% of the SIMs are recurrent.

(*continues below*)

III

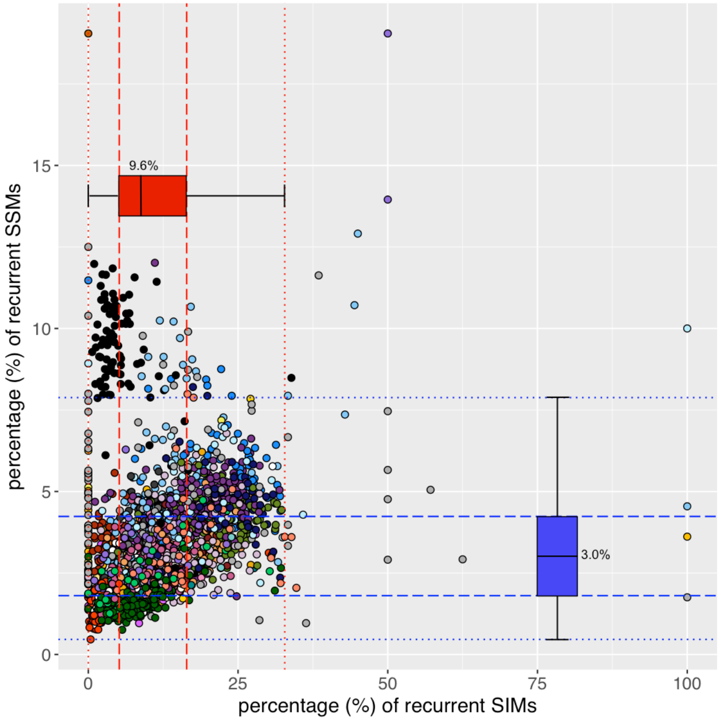

(*continued from above*)

(III) Percentage of recurrent SIMs versus recurrent SSMs. The red boxplot corresponds to the recurrent SIMs and the blue boxplot to the recurrent SSMs. There are 344 samples from 21 different tumour types for which the percentage of recurrent SSMs and SIMs are both above the third quartile. The four tumour types for which half or more of their samples are in this set: Eso-AdenoCA (54 out of 97), ColoRect-AdenoCA (28 out of 52), Panc-AdenoCA (116 out of 232) and Cervix-AdenoCA (1 out of 2). There are 381 samples from 18 different tumour types for which both percentages are below the first quartile. For Kidney-RCC 88.8% of the samples are in this set. This is followed by Lung-AdenoCA with 48.6%, Ovary-AdenoCA with 45.5% and Lung-SCC with 44.7%.

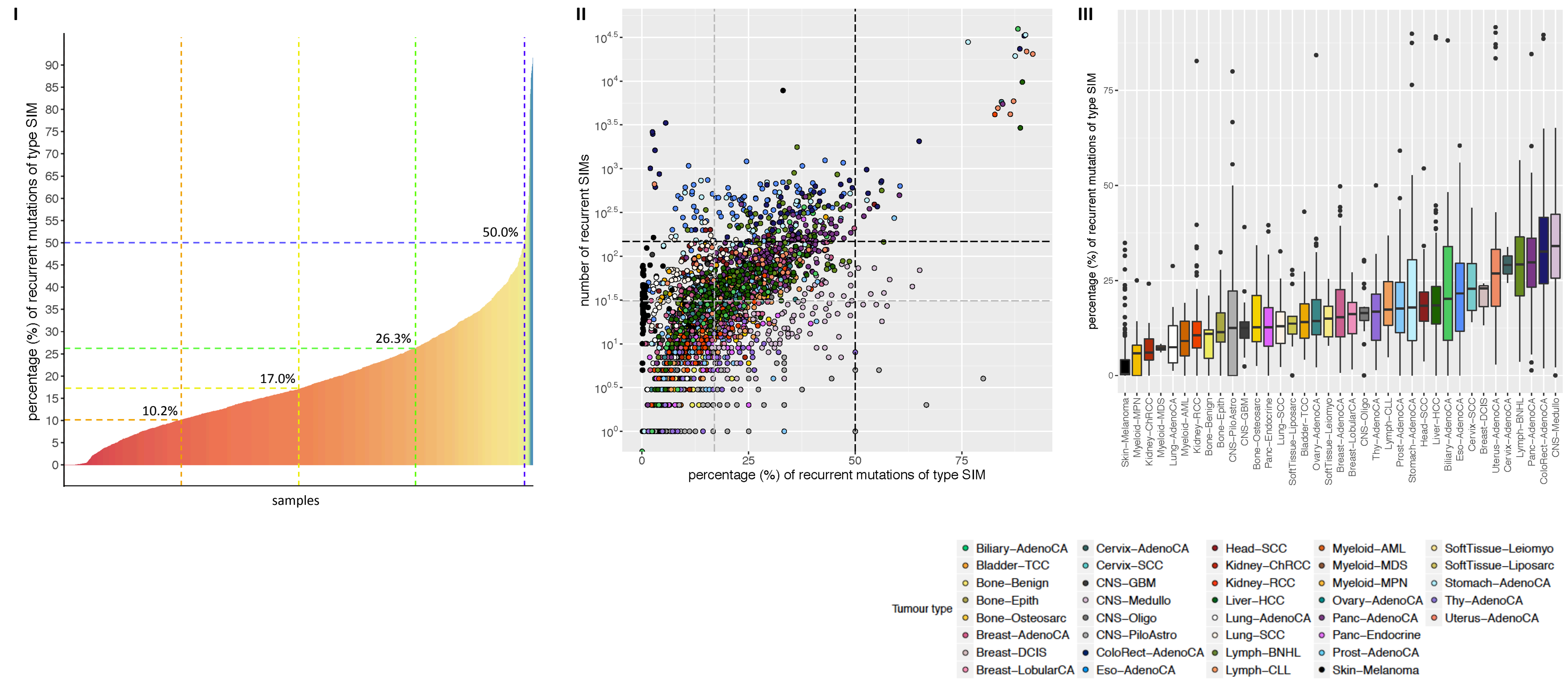

Fig G. The percentage of recurrent mutations of type SIM.

(I) Recurrent mutations show a higher percentage of type SIM than mutations overall. The yellow line indicates the median percentage of mutations of type SIM across the dataset (17%). To the right of the vertical yellow line the samples have a percentage above the median. The orange (10.2%) and green (26.3%) lines indicate the first and third quartile, respectively. The Q1-1.5xIQR is equal to 0% and is not shown. The blue line (50%) indicates the Q3+1.5xIQR, to the right of which samples are outliers. There are 45 samples with more recurrent SIMs than SSMs. (II) The percentage of recurrent mutations of type SIM versus the number of recurrent SIMs per sample. The grey lines indicate the medians and the black lines indicate the Q3+1.5xIQR. There are 30 samples from 12 different tumour types that are outliers in terms of percentage and absolute number. This includes 7 samples from ColoRect-AdenoCA, 5 samples from Uterus-AdenoCA and 4 each from Panc-AdenoCA and Stomach-AdenoCA. (III) The boxplots per tumour type representing the percentage of recurrent mutations of type SIM, which show a considerable variability within and between tumour types. They are ordered according to the median percentage.

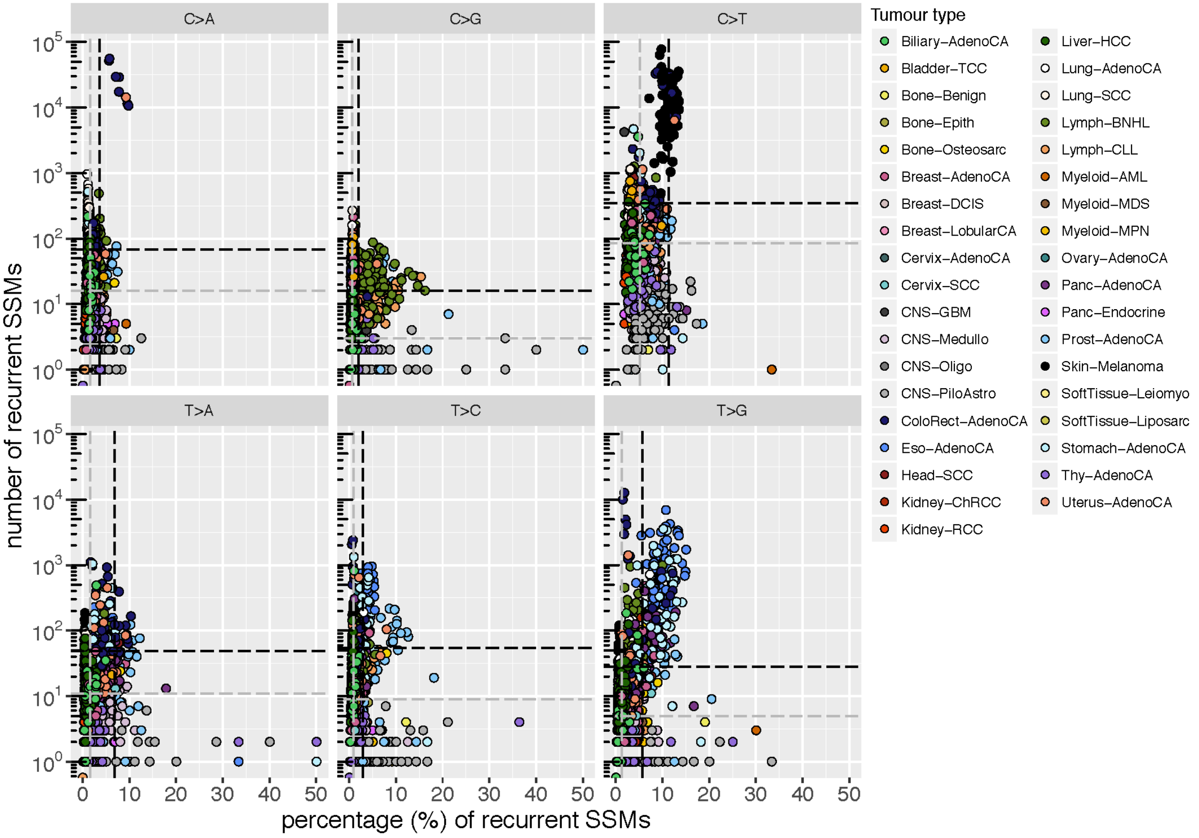

Fig H. Absolute and relative numbers of recurrent SSMs across the six subtypes.

For each sample the percentage and absolute number of recurrent SSMs per subtype is shown. The grey lines indicate the medians and the black lines the Q3+1.5xIQR based on, for the vertical lines, the percentage of SSMs of the particular subtype that is recurrent and, for the horizontal lines, the absolute numbers. For C>A SSMs there are 12 samples that are outliers in terms of percentage and number of recurrent SSMs. Of these 12 there are seven ColoRect-AdenoCA samples and one Uterus-AdenoCa sample that particularly stand out. Each has over 10,000 recurrent C>A SSMs and at least 5.6% are recurrent. For C>G SSMs there are 82 outliers of which 62 are from Lymph-BNHL. There are 37 outliers for the C>T SSMs of which 33 are Skin-Melanoma samples. For T>A SSMs there are 17 outliers of which 7 are from ColoRect-AdenoCA and 5 from Prost_AdenoCA. For T>C SSMs there are 99 outliers of which 58 are from Eso-AdenoCA and 17 from Stomach-AdenoCA. Finally, for T>G SSMs there are 187 outliers of which again Eso-AdenoCA and Stomach-AdenoCA form the majority with 83 and 42 samples, respectively.

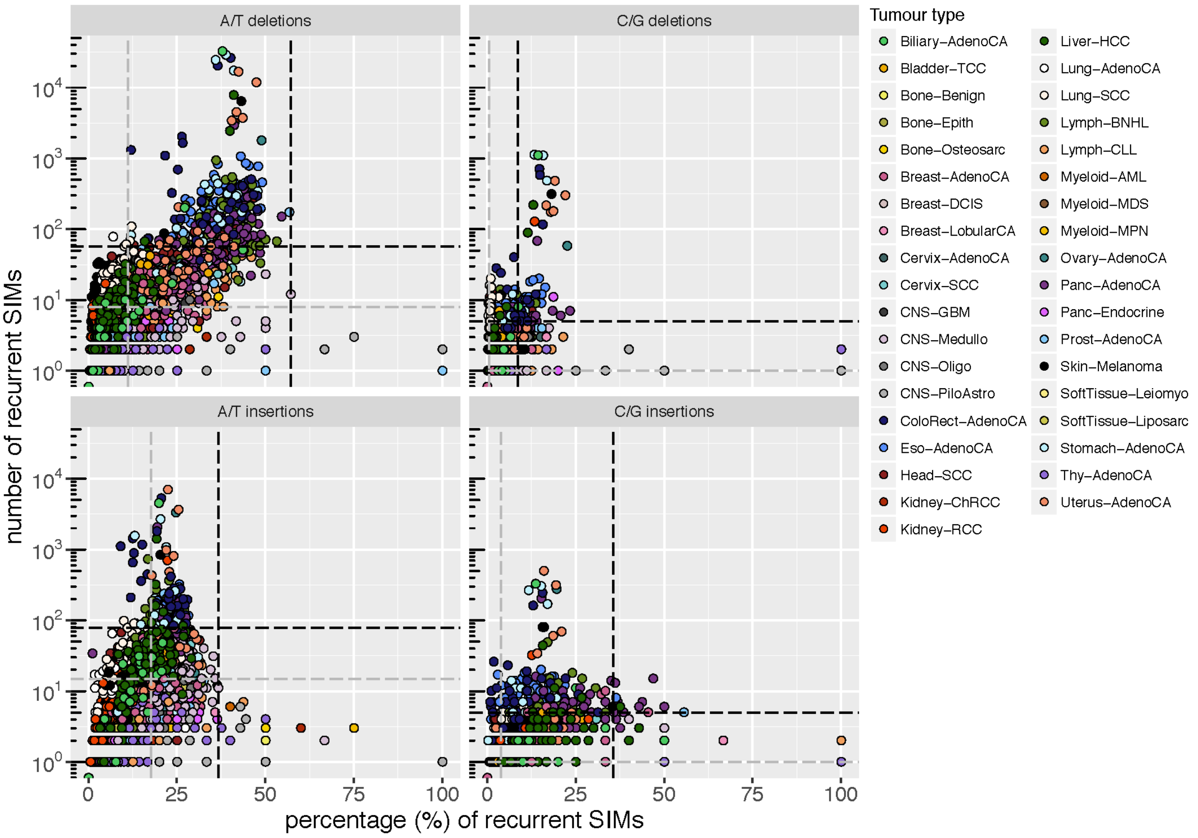

Fig I. Absolute and relative numbers of recurrent 1 bp SIMs across the four subtypes.

For each sample the percentage and absolute number of recurrent 1 bp SIMs per subtype is shown. The grey lines indicate the medians and the black lines the Q3+1.5xIQR based on, for the vertical lines, the percentage of SIMs of the particular subtype that is recurrent and, for the horizontal lines, the absolute numbers. There is a large spread of the percentages for 1 bp A/T deletions and insertions and therefore there are no outliers in terms of percentage and absolute number. There are 352 outliers in terms of absolute number of recurrent 1 bp A/T deletions of which Eso-AdenoCA constitutes the largest percentage (22.4%), followed by Panc-AdenoCA (20.2%) and Lymph-BNHL (18.8%). For the number of recurrent 1 bp A/T insertions there are 236 outliers of which again Eso-AdenoCA contributes the highest percentage of samples (26.7%), followed by ColoRect-AdenoCA (19.5%) and Panc-AdenoCA (16.1%). For recurrent 1 bp C/G deletions there are 58 outliers in terms of percentage and absolute number of which 29.3% are from Eso-AdenoCA and 19.0% from ColoRect-AdenoCA. For recurrent 1 bp C/G insertions there are 9 outliers in terms of percentages and absolute numbers of which 7 are from Panc-AdenoCA and 1 each from Eso-AdenoCA and Liver-HCC.
