# Supplementary material for: Recurrent somatic mutations reveal new insights into consequences of mutagenic processes in cancer
Source: PLoS Comput Biol. 2019 Nov 25;15(11):e1007496. doi: 10.1371/journal.pcbi.1007496 (PMC6901237; doi:10.1371/journal.pcbi.1007496)
Supplement: S2 File — (PDF) [file pcbi.1007496.s012.pdf]

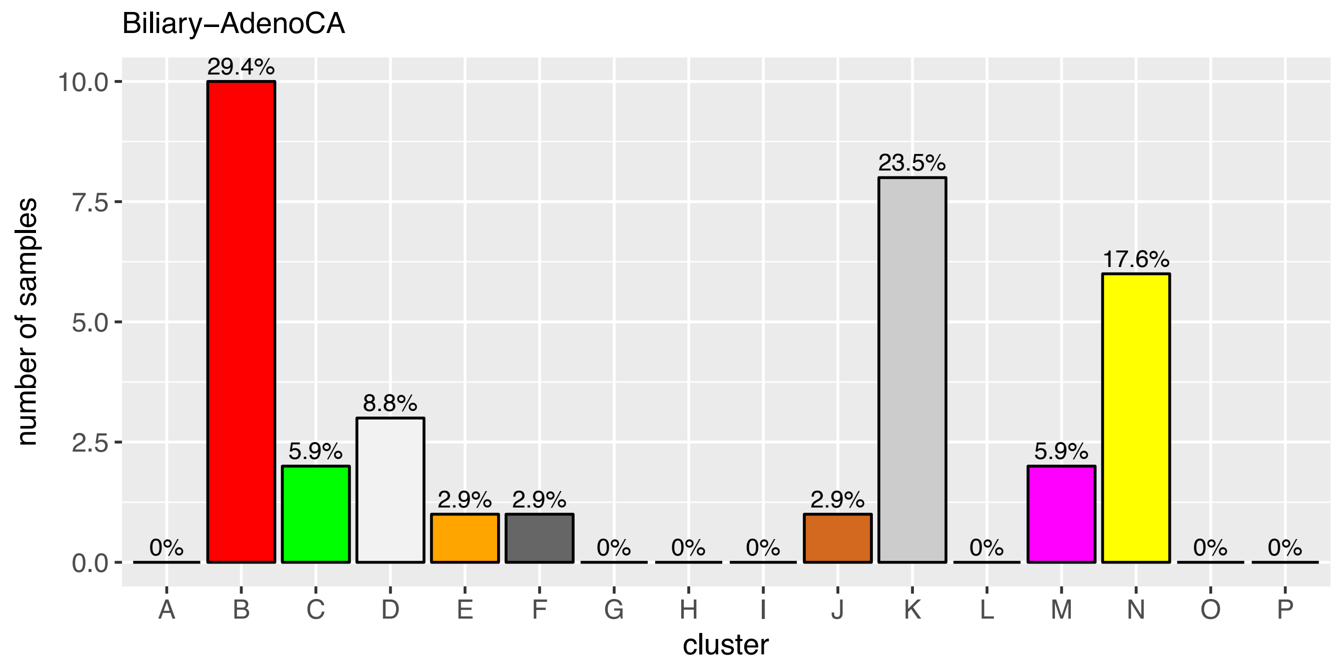

Biliary−AdenoCA
number of samples
0.0
2.5
5.0
7.5
10.0
0%
29.4%
5.9%
8.8%
2.9%
2.9%
0%
0%
0%
2.9%
23.5%
0%
5.9%
17.6%
0%
0%
A B C D E F G H I J K L M N O P
cluster

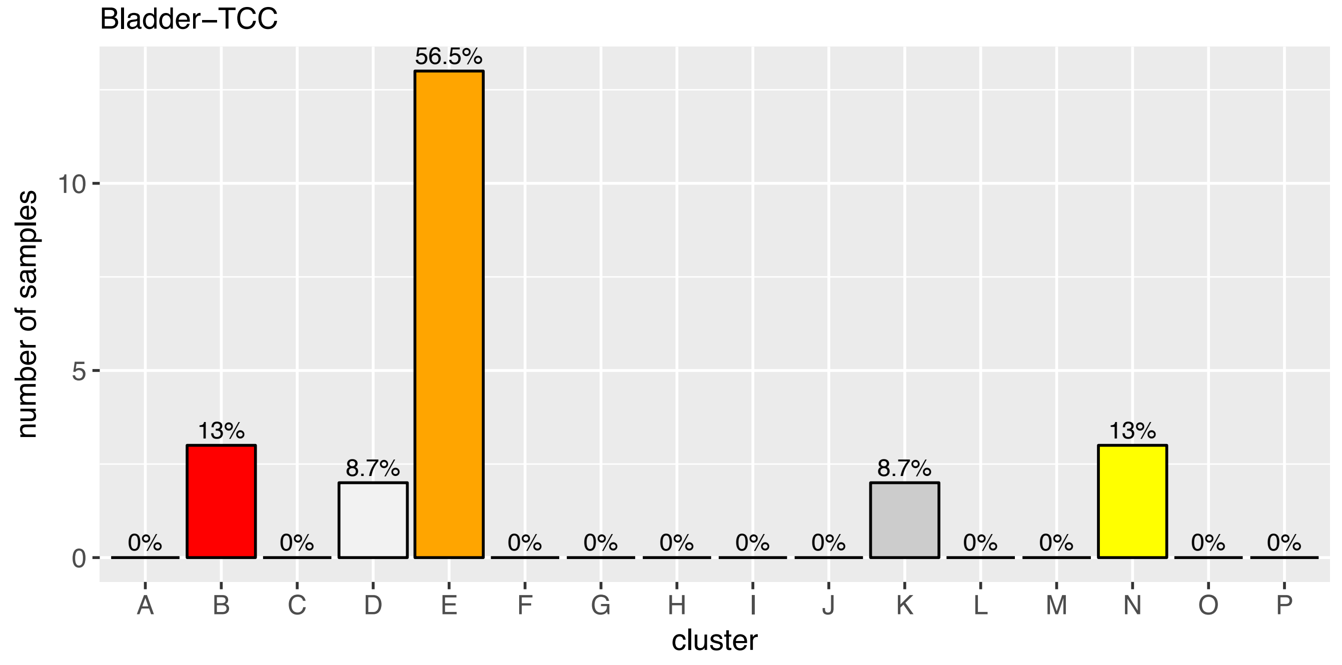

Bladder−TCC
number of samples
0
5
10
0%
13%
0%
8.7%
56.5%
0%
0%
0%
0%
0%
8.7%
0%
0%
13%
0%
0%
A B C D E F G H I J K L M N O P
cluster

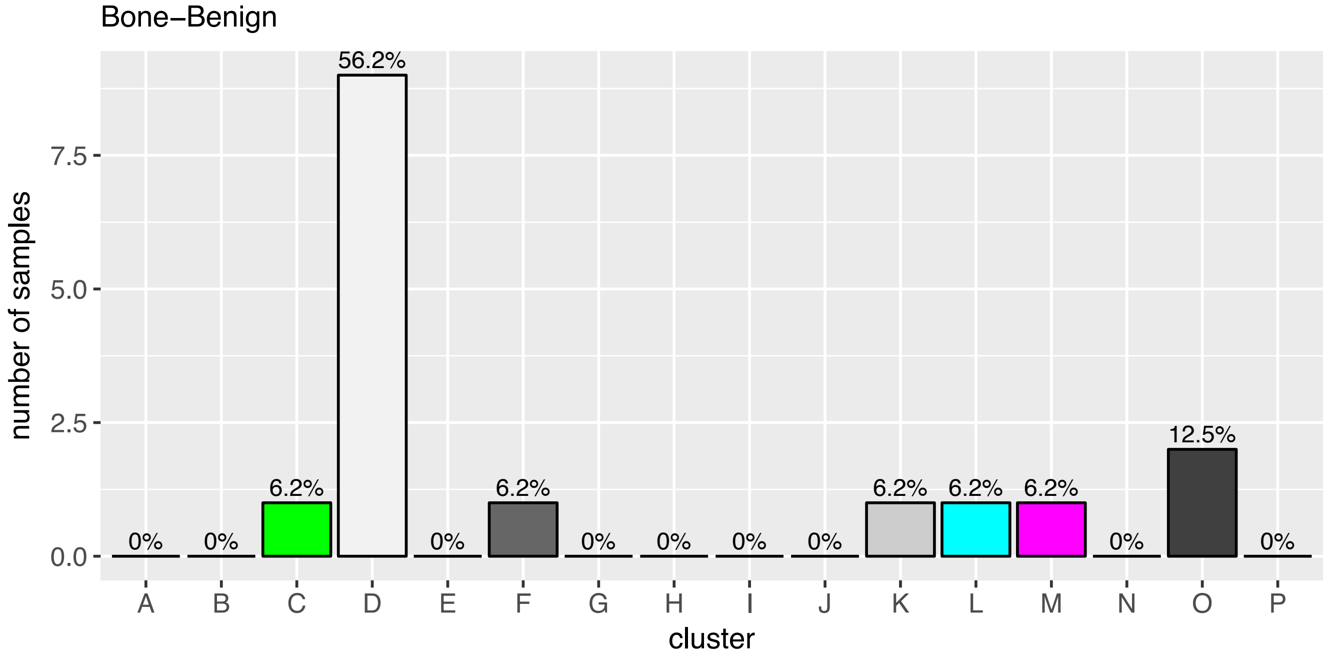
Bone−Benign
number of samples
7.5
5.0
2.5
0.0
0%
0%
6.2%
56.2%
0%
6.2%
0%
0%
0%
0%
6.2%
6.2%
6.2%
0%
12.5%
0%
A
B
C
D
E
F
G
H
I
J
K
L
M
N
O
P
cluster

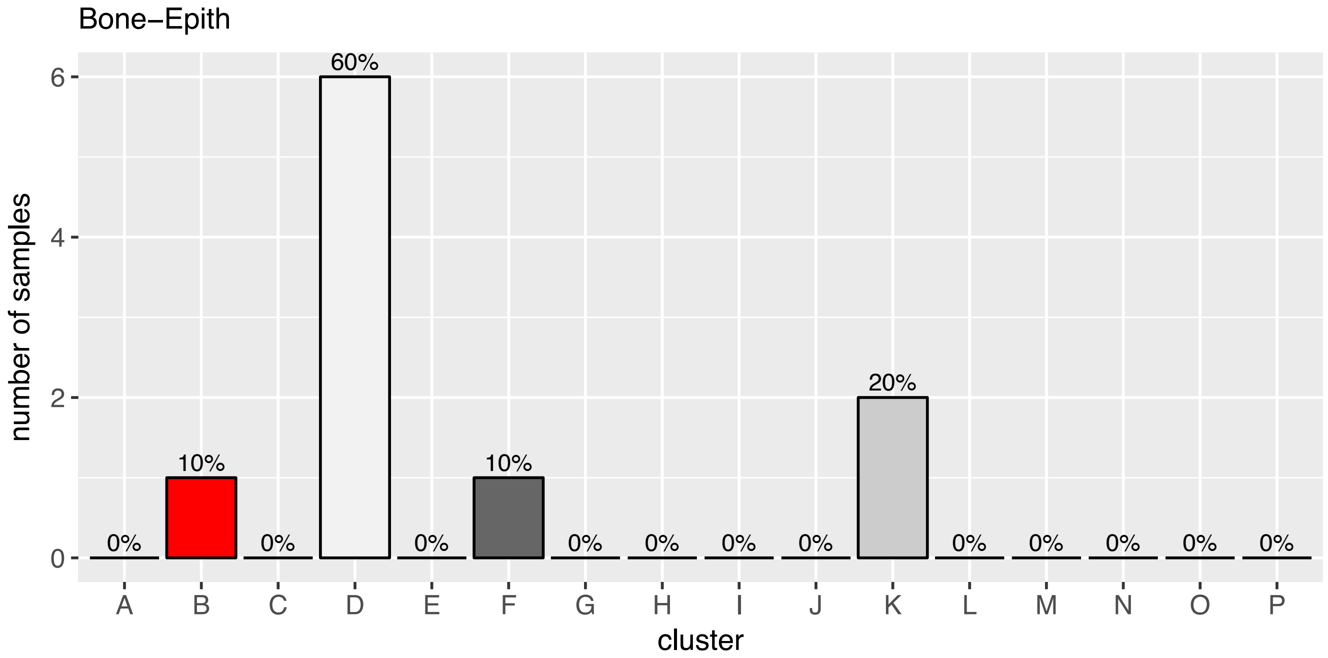
Bone−Epith
number of samples
6
4
2
0
0%
10%
0%
60%
0%
10%
0%
0%
0%
0%
20%
0%
0%
0%
0%
0%
A
B
C
D
E
F
G
H
I
J
K
L
M
N
O
P
cluster

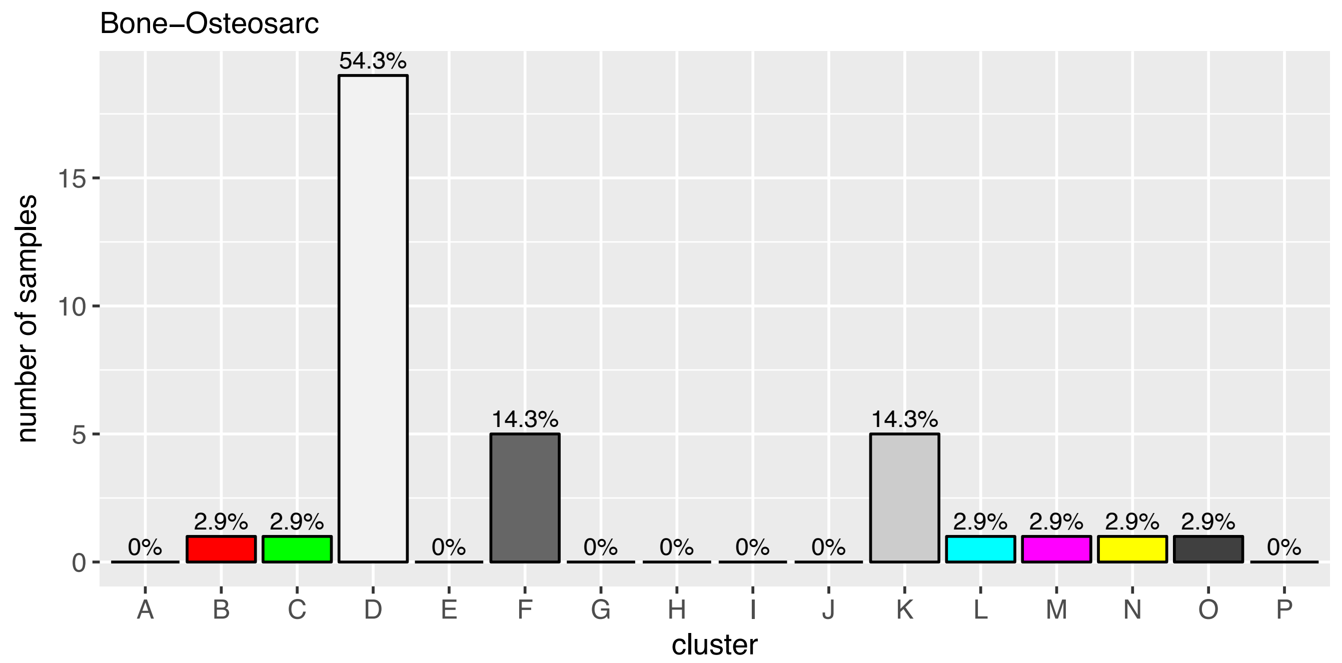
Bone−Osteosarc
number of samples
0
5
10
15
0%
2.9%
2.9%
54.3%
0%
14.3%
0%
0%
0%
0%
14.3%
2.9%
2.9%
2.9%
2.9%
0%
A B C D E F G H I J K L M N O P
cluster

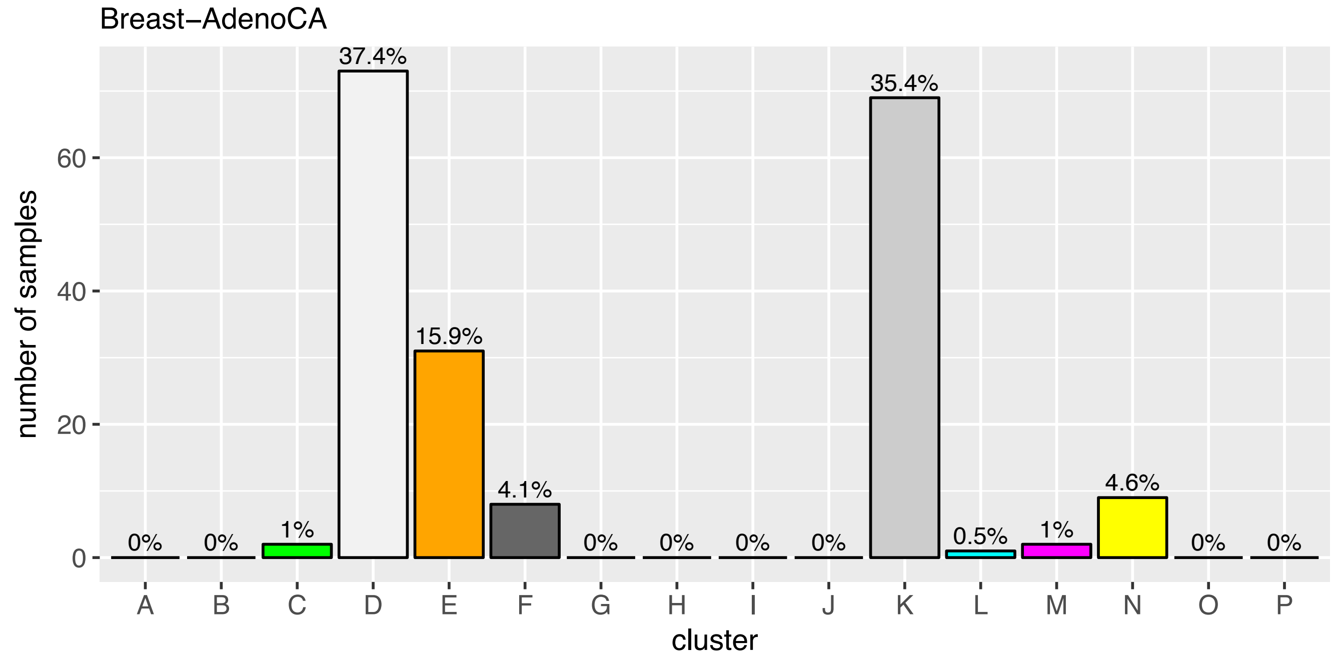
Breast−AdenoCA
number of samples
0
20
40
60
0%
0%
1%
37.4%
15.9%
4.1%
0%
0%
0%
0%
35.4%
0.5%
1%
4.6%
0%
0%
A B C D E F G H I J K L M N O P
cluster

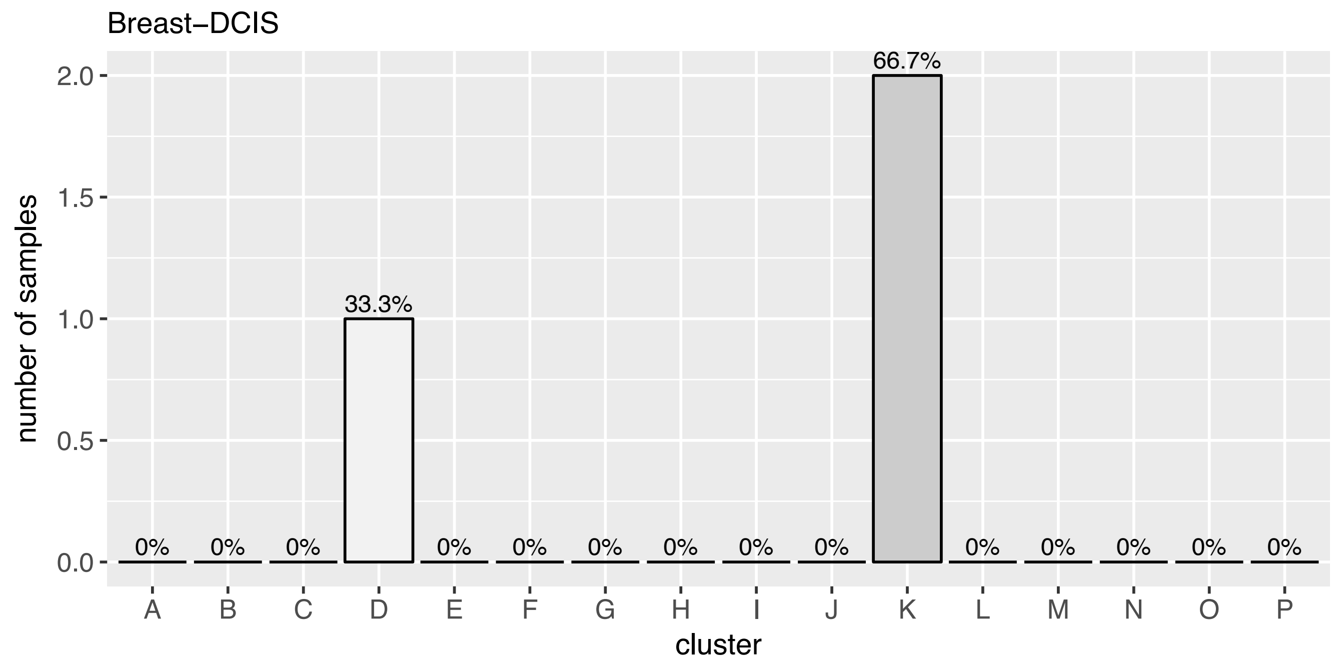
Breast−DCIS
number of samples
cluster
33.3%
66.7%
0%
A B C D E F G H I J K L M N O P
0.0 0.5 1.0 1.5 2.0

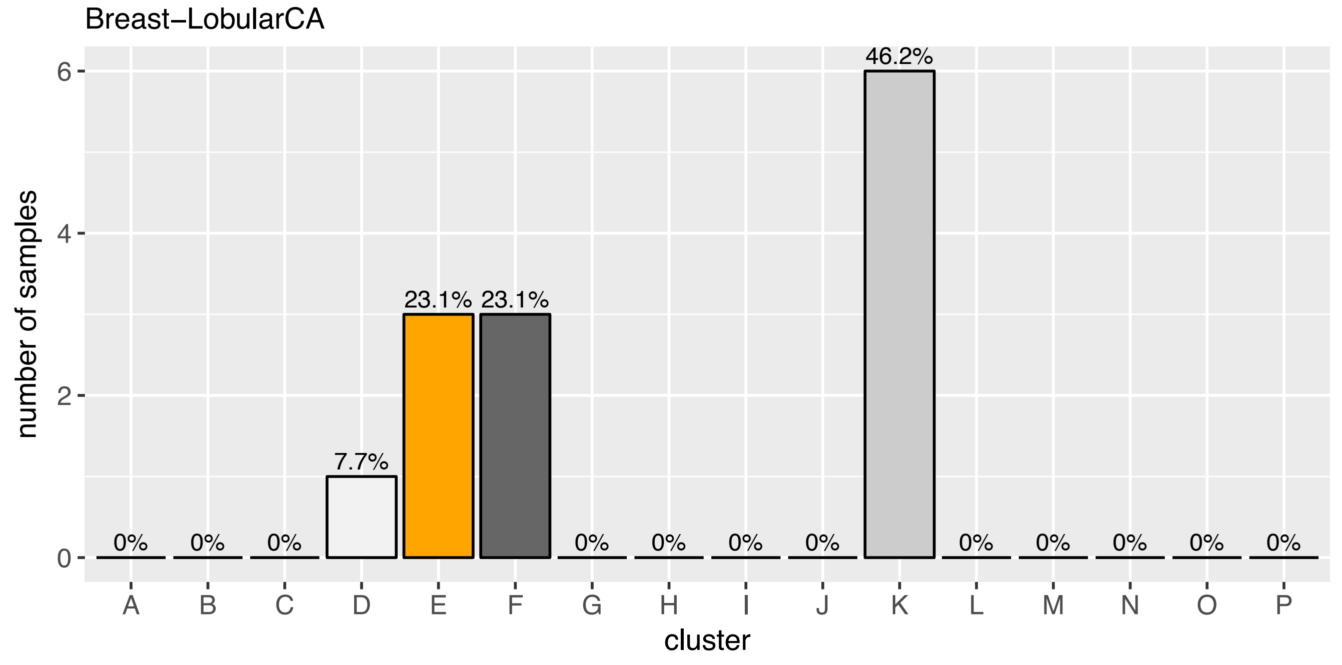
Breast−LobularCA
number of samples
cluster
7.7%
23.1%
23.1%
46.2%
0%
A B C D E F G H I J K L M N O P
0 2 4 6

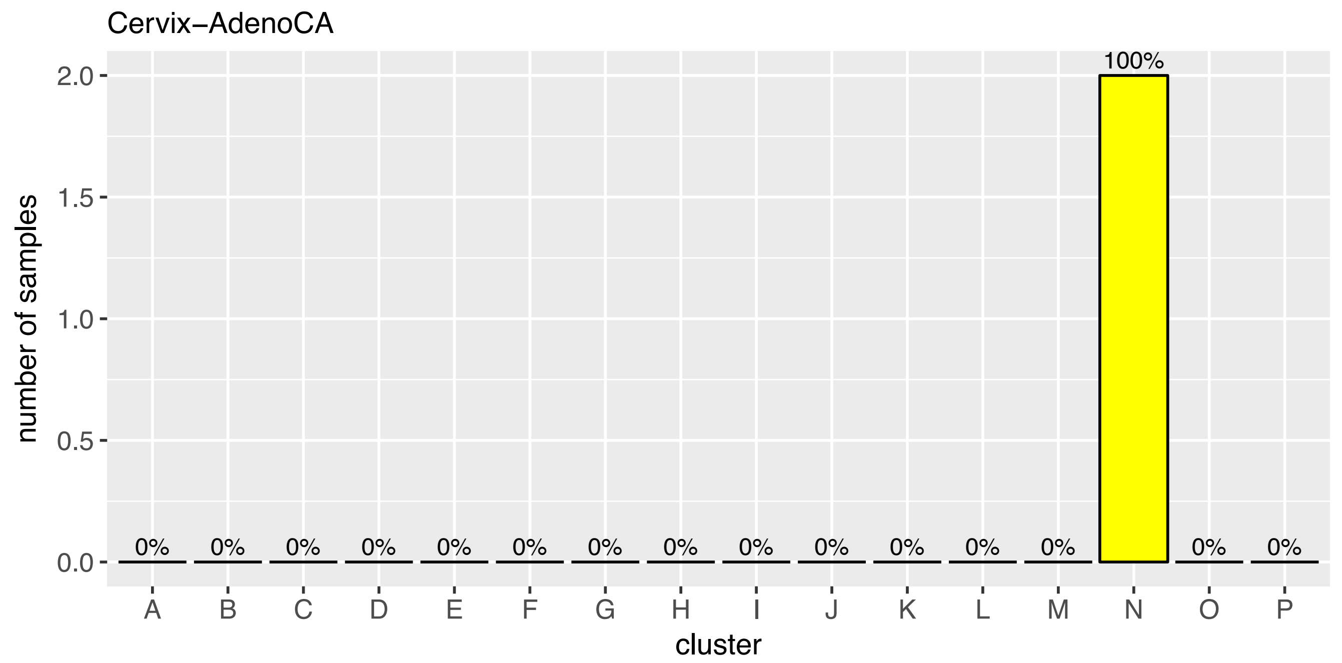

Cervix−AdenoCA
number of samples
2.0
1.5
1.0
0.5
0.0
100%
0%
A
B
C
D
E
F
G
H
I
J
K
L
M
N
O
P
cluster

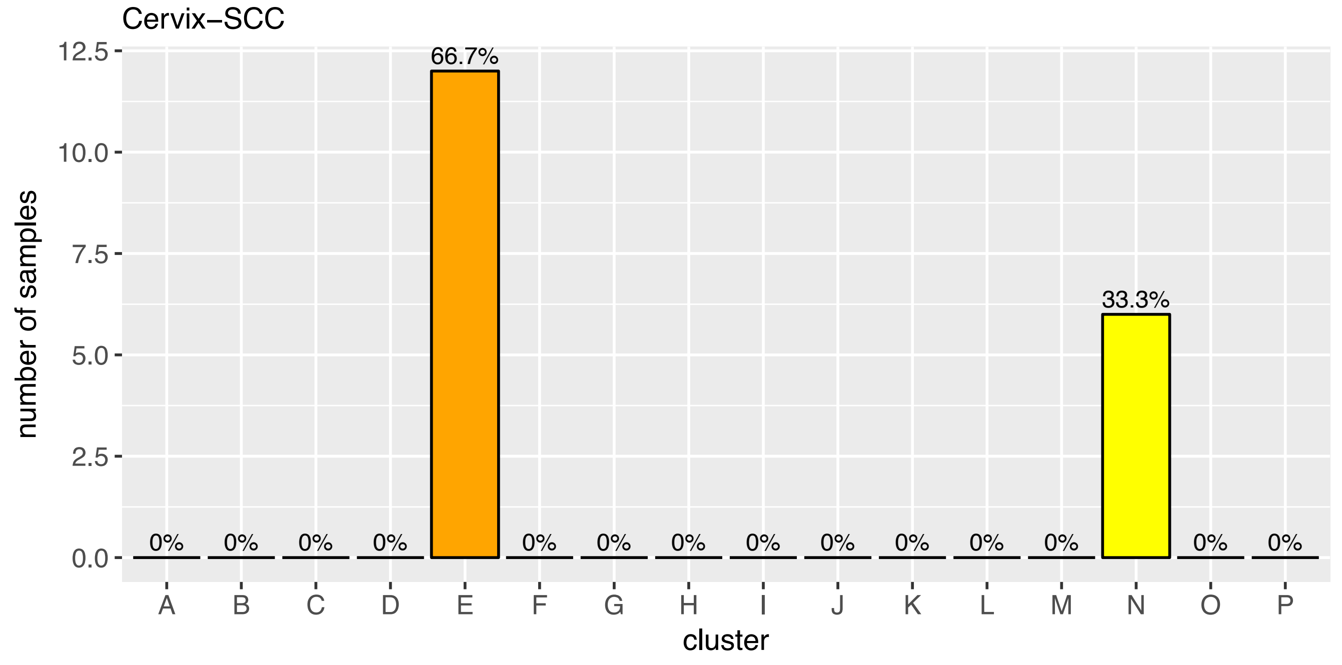

Cervix−SCC
number of samples
12.5
10.0
7.5
5.0
2.5
0.0
66.7%
33.3%
0%
A
B
C
D
E
F
G
H
I
J
K
L
M
N
O
P
cluster

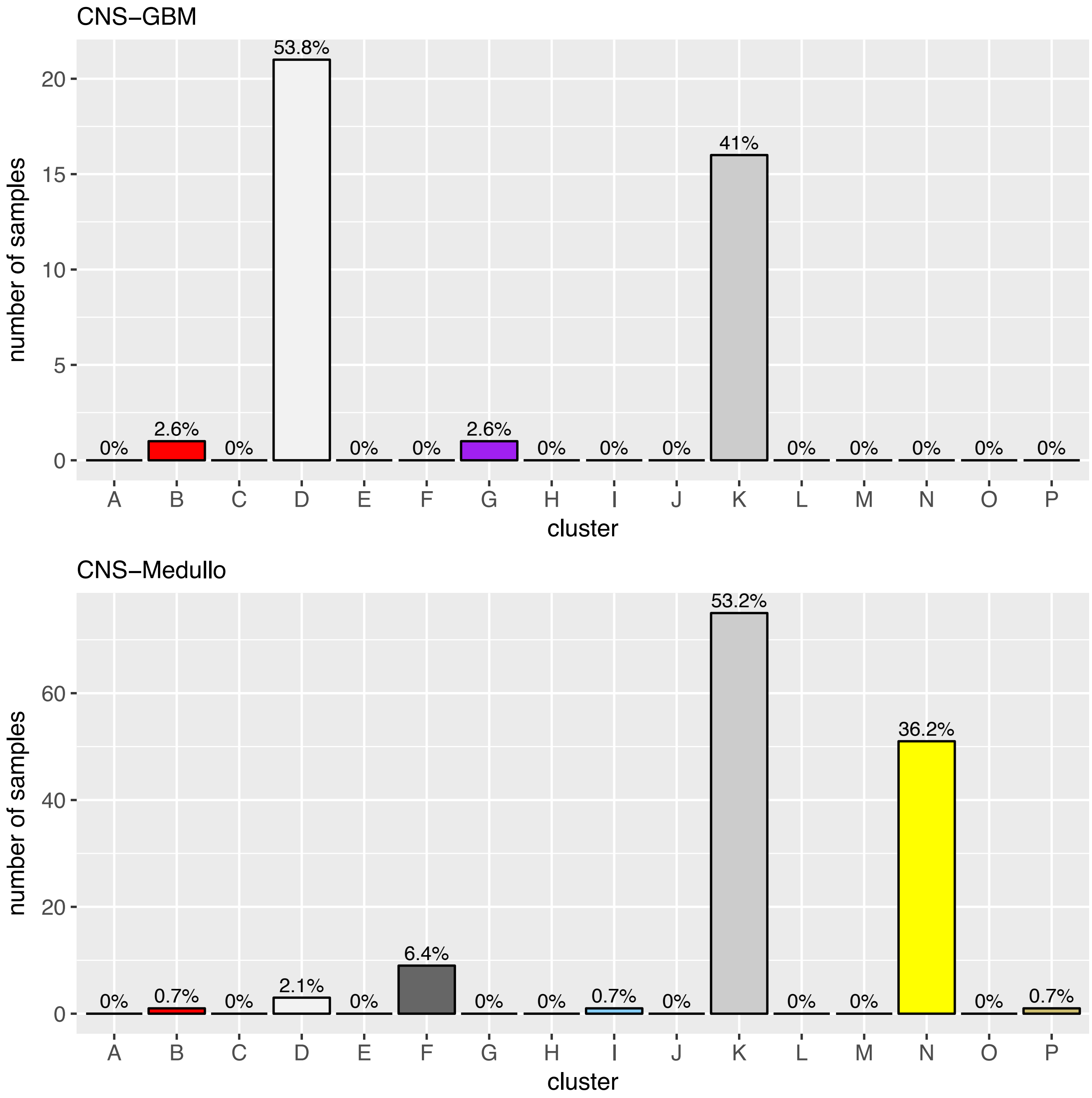
CNS−GBM
CNS−Medullo
number of samples
cluster
A
B
C
D
E
F
G
H
I
J
K
L
M
N
O
P
0%
2.6%
53.8%
41%
0.7%
2.1%
6.4%
53.2%
36.2%

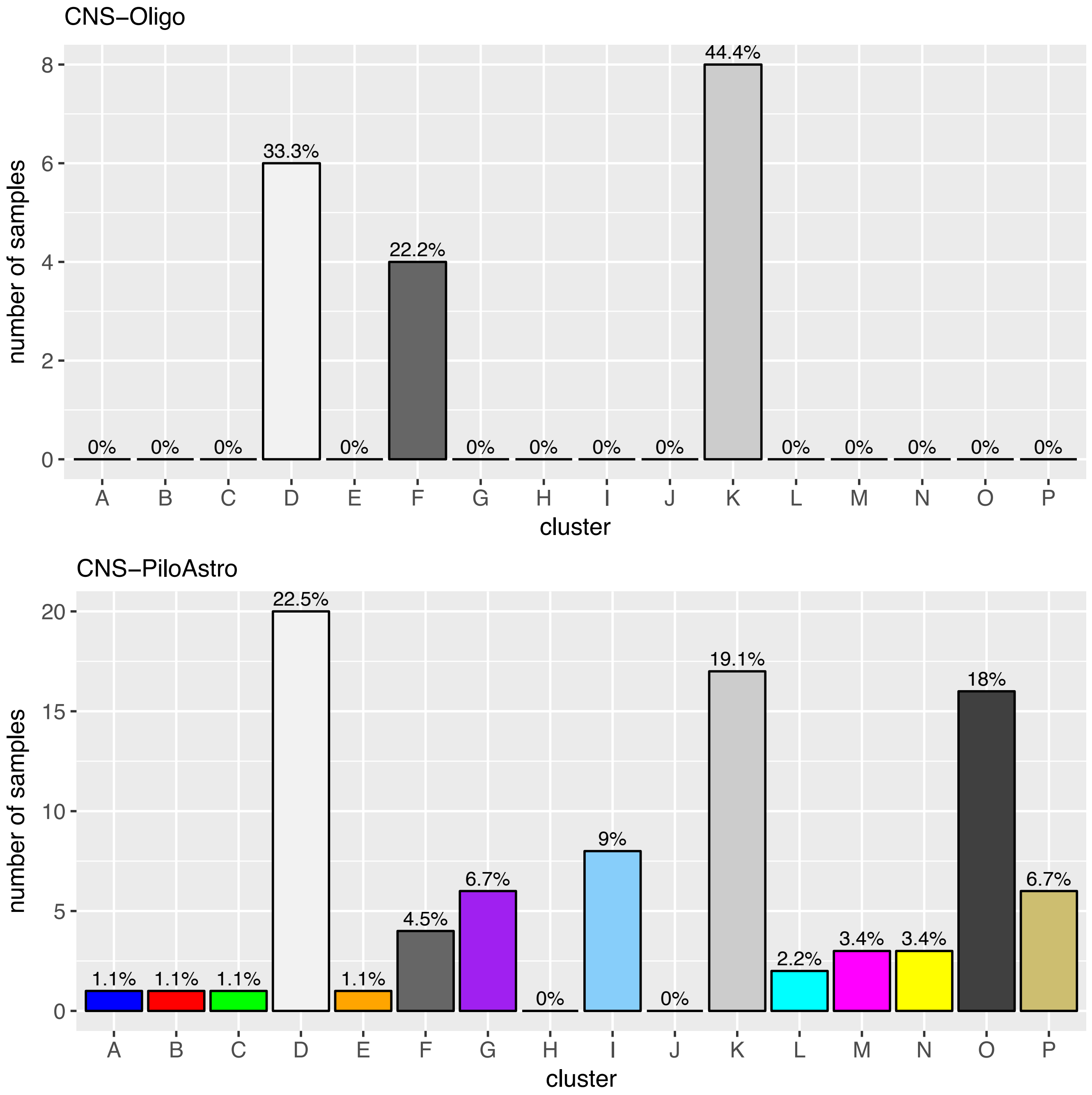

CNS−Oligo
number of samples
0
2
4
6
8
0%
0%
0%
33.3%
0%
22.2%
0%
0%
0%
0%
44.4%
0%
0%
0%
0%
0%
A
B
C
D
E
F
G
H
I
J
K
L
M
N
O
P
cluster
CNS−PiloAstro
number of samples
0
5
10
15
20
1.1%
1.1%
1.1%
22.5%
1.1%
4.5%
6.7%
0%
9%
0%
19.1%
2.2%
3.4%
3.4%
18%
6.7%
A
B
C
D
E
F
G
H
I
J
K
L
M
N
O
P
cluster

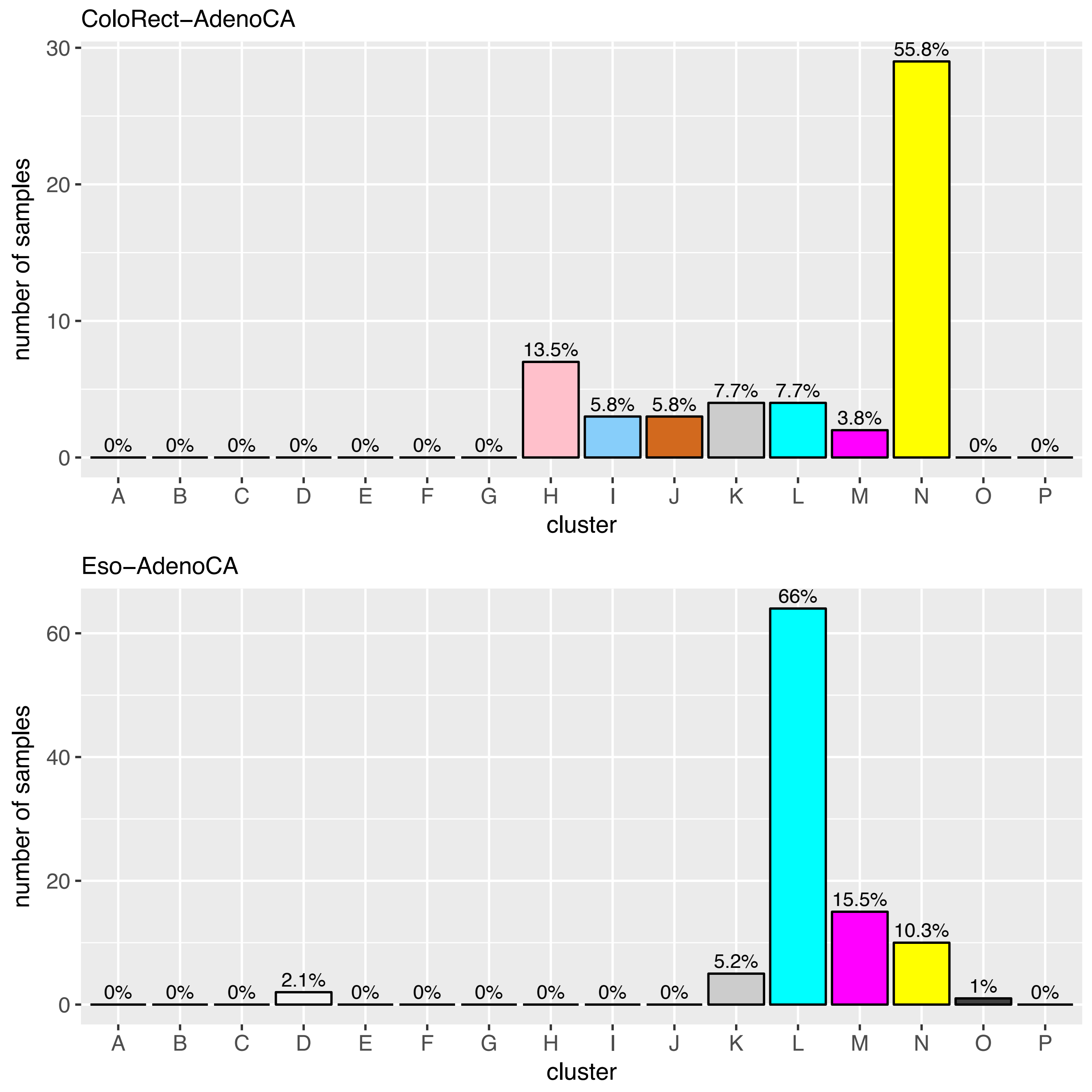
ColoRect−AdenoCA
number of samples
0
10
20
30
0%
0%
0%
0%
0%
0%
0%
13.5%
5.8%
5.8%
7.7%
7.7%
3.8%
55.8%
0%
0%
A B C D E F G H I J K L M N O P
cluster
Eso−AdenoCA
number of samples
0
20
40
60
0%
0%
0%
2.1%
0%
0%
0%
0%
0%
0%
5.2%
66%
15.5%
10.3%
1%
0%
A B C D E F G H I J K L M N O P
cluster

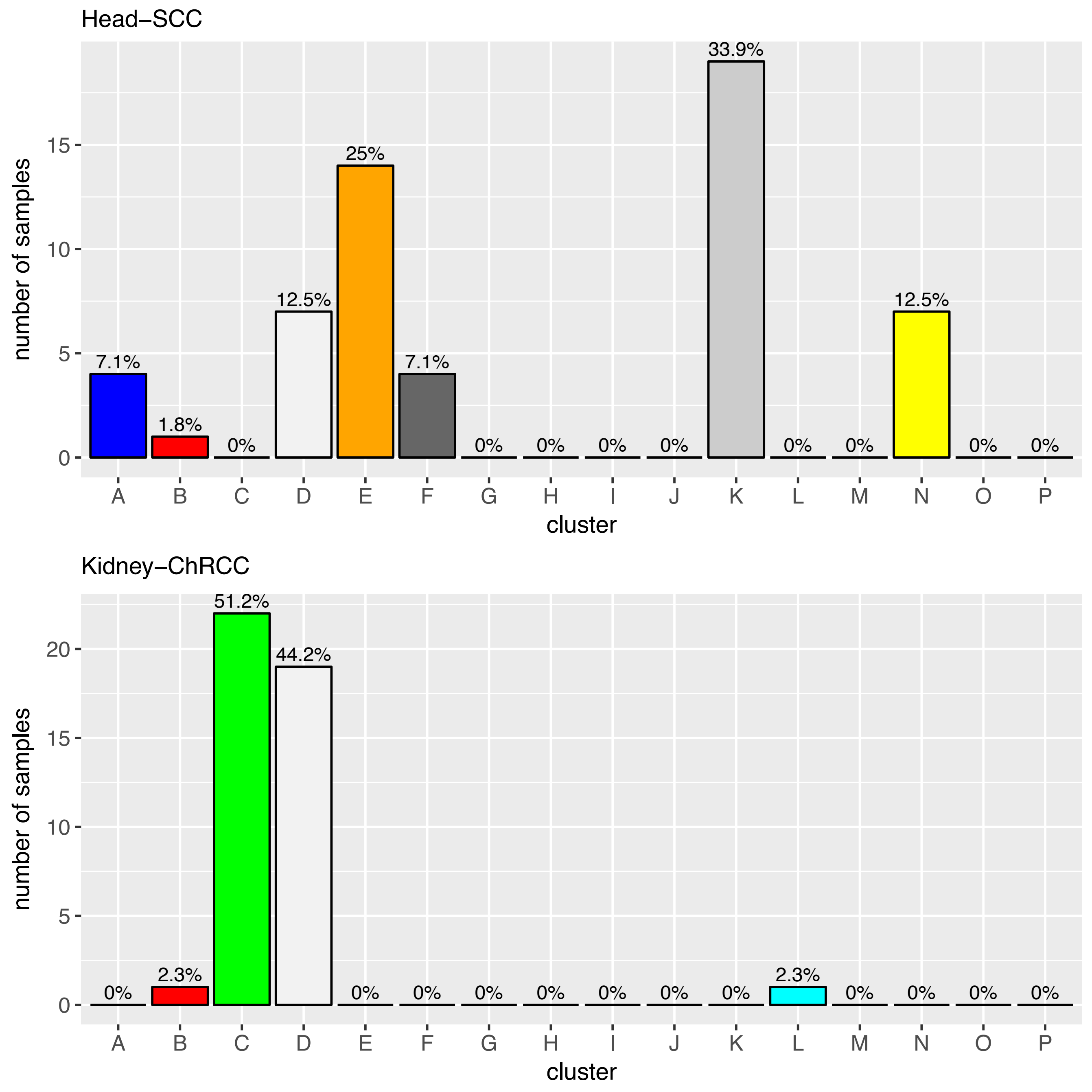
Head−SCC
number of samples
0
5
10
15
7.1%
1.8%
0%
12.5%
25%
7.1%
0%
0%
0%
0%
33.9%
0%
0%
12.5%
0%
0%
A B C D E F G H I J K L M N O P
cluster
Kidney−ChRCC
number of samples
0
5
10
15
20
0%
2.3%
51.2%
44.2%
0%
0%
0%
0%
0%
0%
0%
2.3%
0%
0%
0%
0%
A B C D E F G H I J K L M N O P
cluster

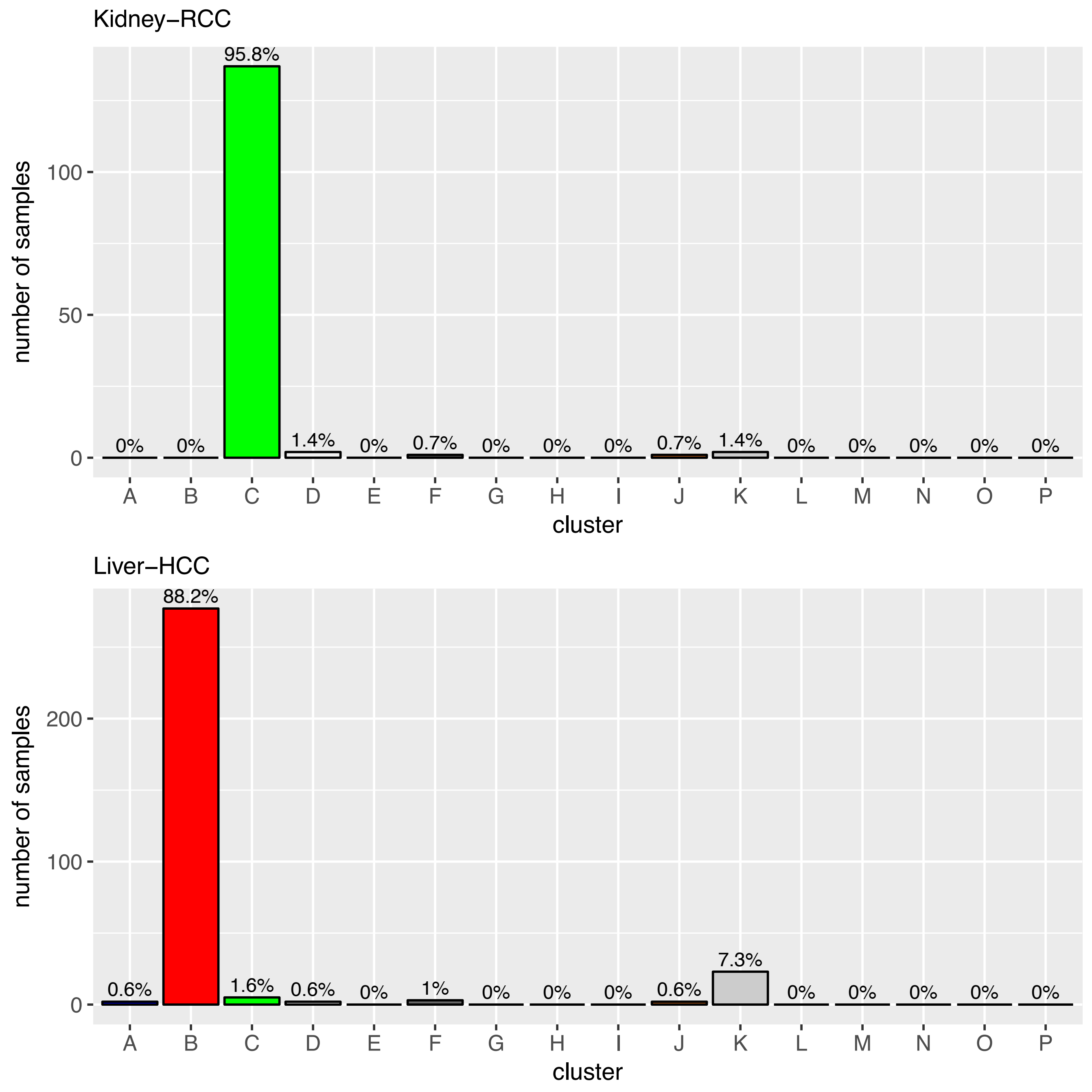

Kidney−RCC
95.8%
0%
0%
1.4%
0%
0.7%
0%
0%
0%
0.7%
1.4%
0%
0%
0%
0%
0%
number of samples
0
50
100
A B C D E F G H I J K L M N O P
cluster
Liver−HCC
88.2%
0.6%
1.6%
0.6%
0%
1%
0%
0%
0%
0.6%
7.3%
0%
0%
0%
0%
0%
number of samples
0
100
200
A B C D E F G H I J K L M N O P
cluster

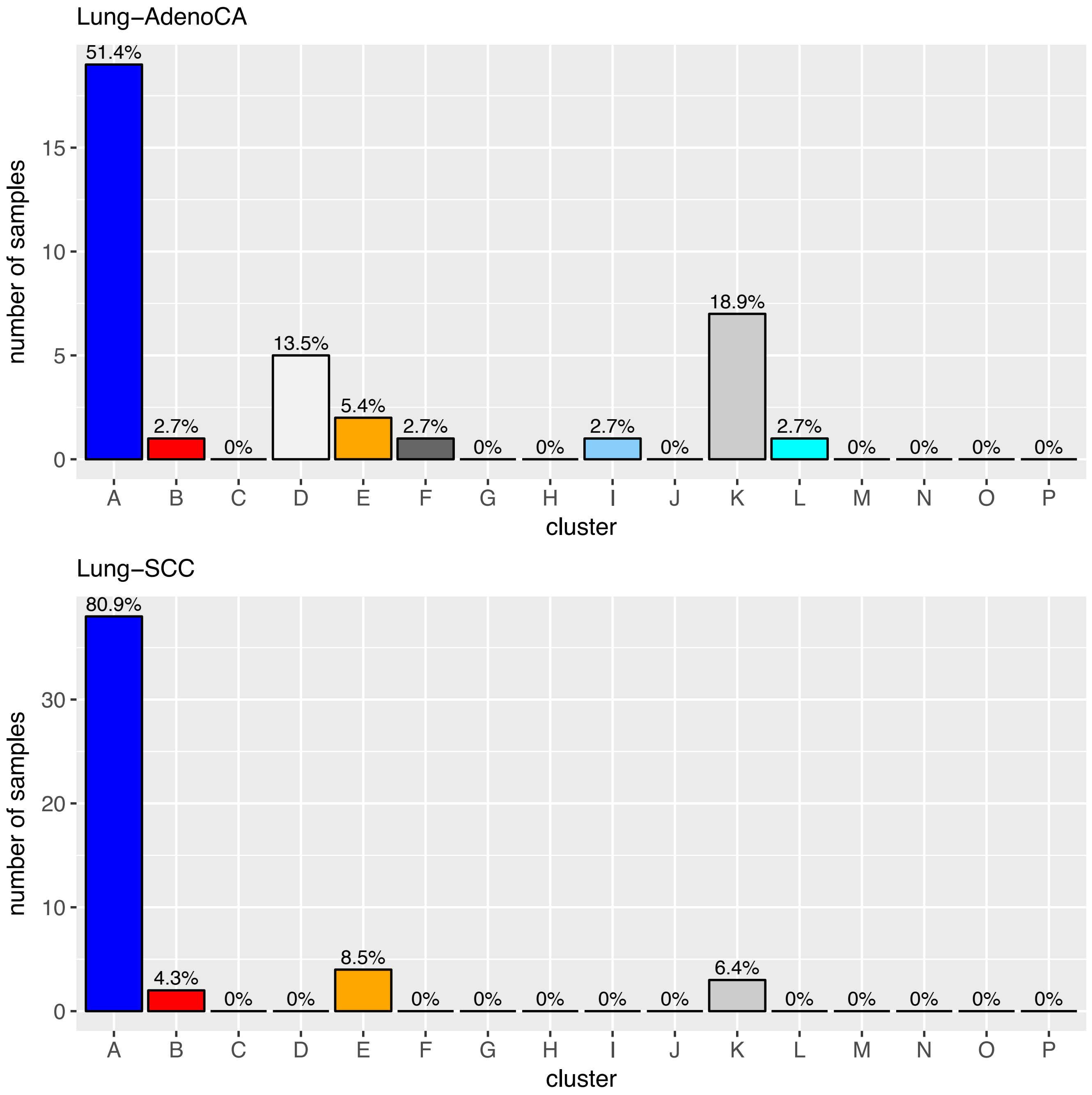

Lung−AdenoCA
51.4%
2.7%
0%
13.5%
5.4%
2.7%
0%
0%
2.7%
0%
18.9%
2.7%
0%
0%
0%
0%
number of samples
0
5
10
15
A B C D E F G H I J K L M N O P
cluster
Lung−SCC
80.9%
4.3%
0%
0%
8.5%
0%
0%
0%
0%
0%
6.4%
0%
0%
0%
0%
0%
number of samples
0
10
20
30
A B C D E F G H I J K L M N O P
cluster

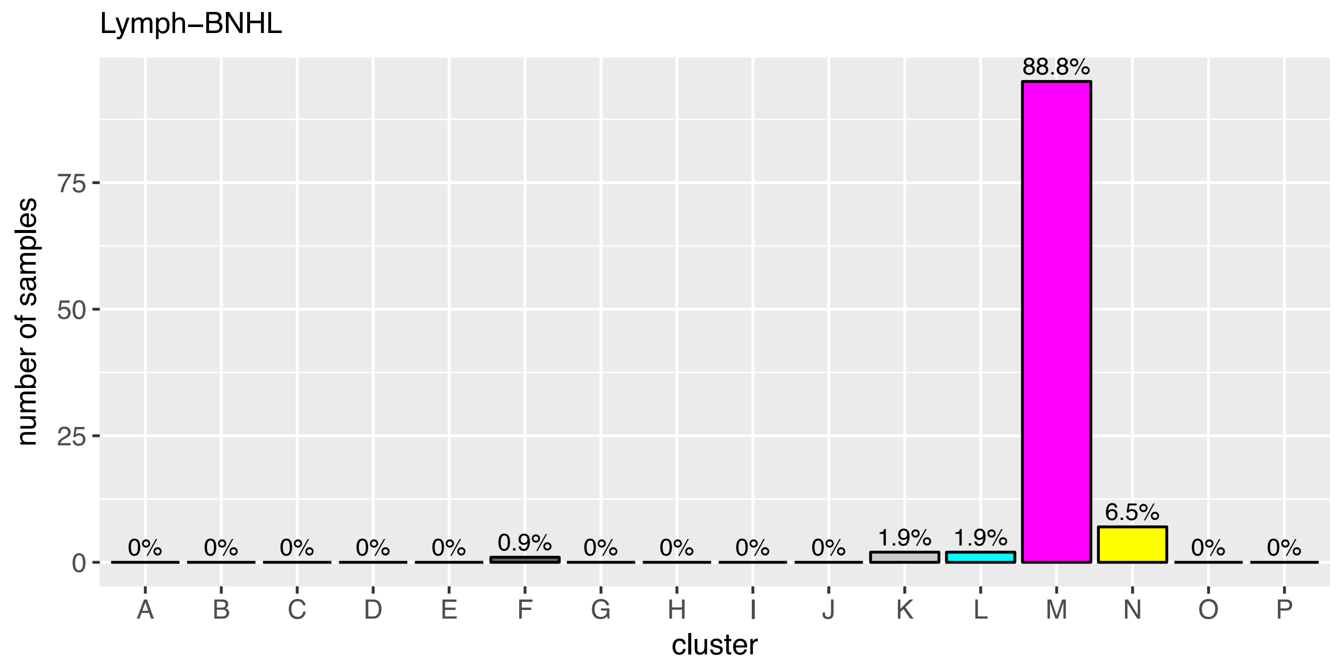
Lymph−BNHL
number of samples
0
25
50
75
0%
0%
0%
0%
0%
0.9%
0%
0%
0%
0%
1.9%
1.9%
88.8%
6.5%
0%
0%
A B C D E F G H I J K L M N O P
cluster

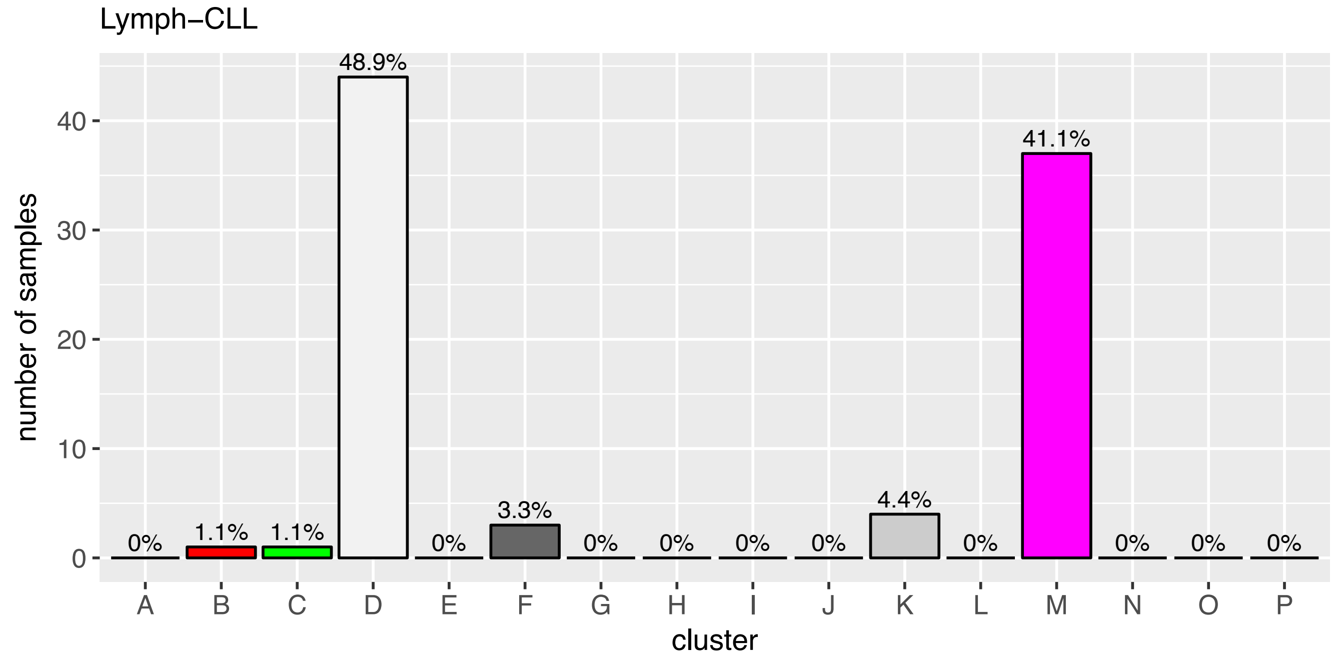
Lymph−CLL
number of samples
0
10
20
30
40
0%
1.1%
1.1%
48.9%
0%
3.3%
0%
0%
0%
0%
4.4%
0%
41.1%
0%
0%
0%
A B C D E F G H I J K L M N O P
cluster

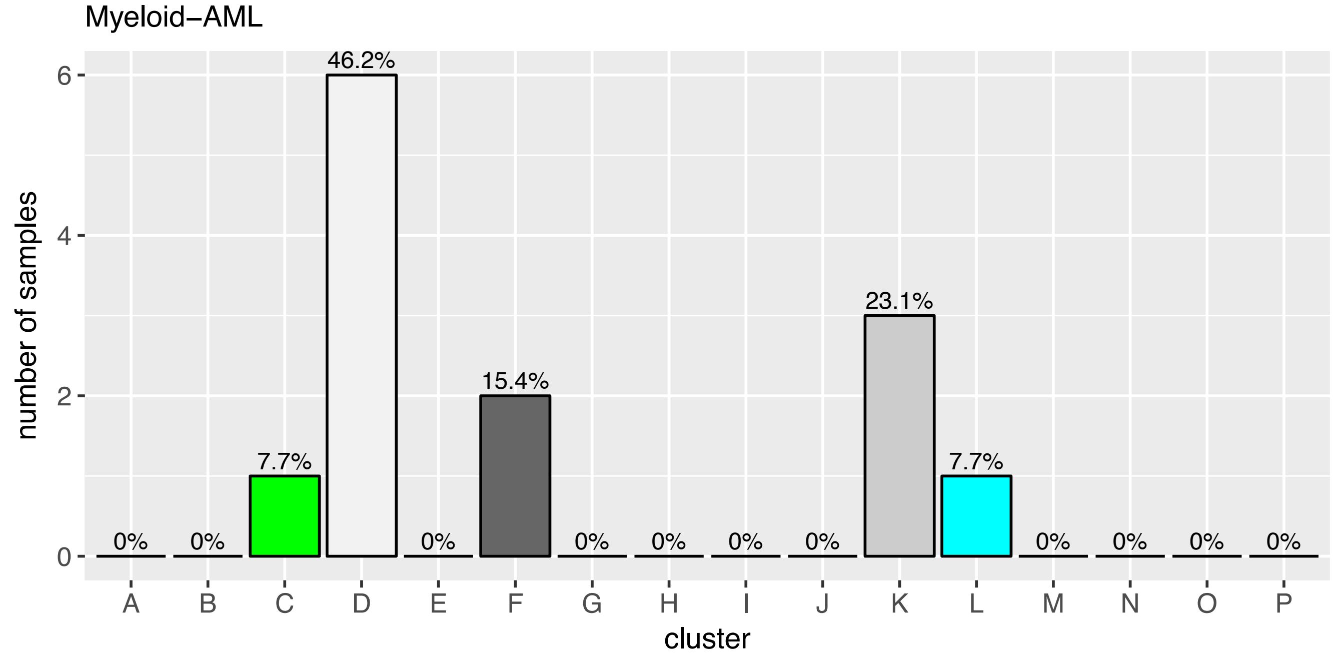

Myeloid−AML
number of samples
0
2
4
6
46.2%
23.1%
15.4%
7.7%
7.7%
0%
A
B
C
D
E
F
G
H
I
J
K
L
M
N
O
P
cluster

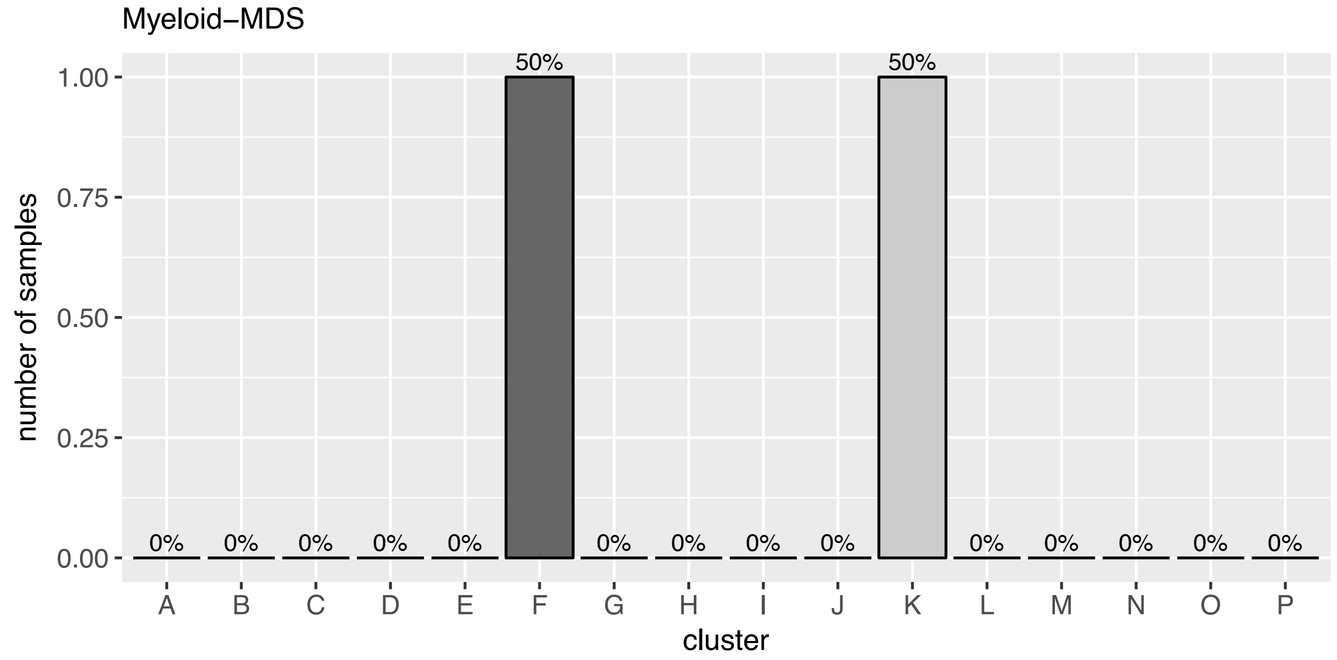

Myeloid−MDS
number of samples
0.00
0.25
0.50
0.75
1.00
50%
50%
0%
A
B
C
D
E
F
G
H
I
J
K
L
M
N
O
P
cluster

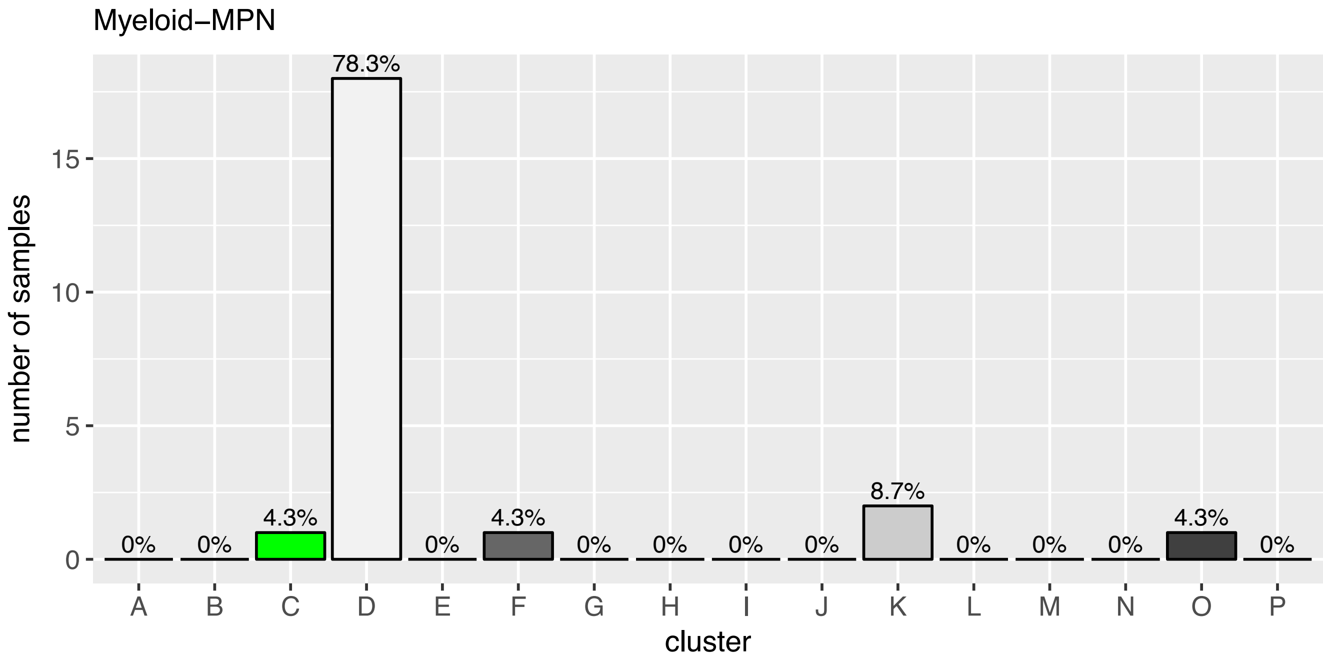
Myeloid−MPN
number of samples
0
5
10
15
78.3%
0%
0%
4.3%
0%
4.3%
0%
0%
0%
0%
8.7%
0%
0%
0%
4.3%
0%
A
B
C
D
E
F
G
H
I
J
K
L
M
N
O
P
cluster

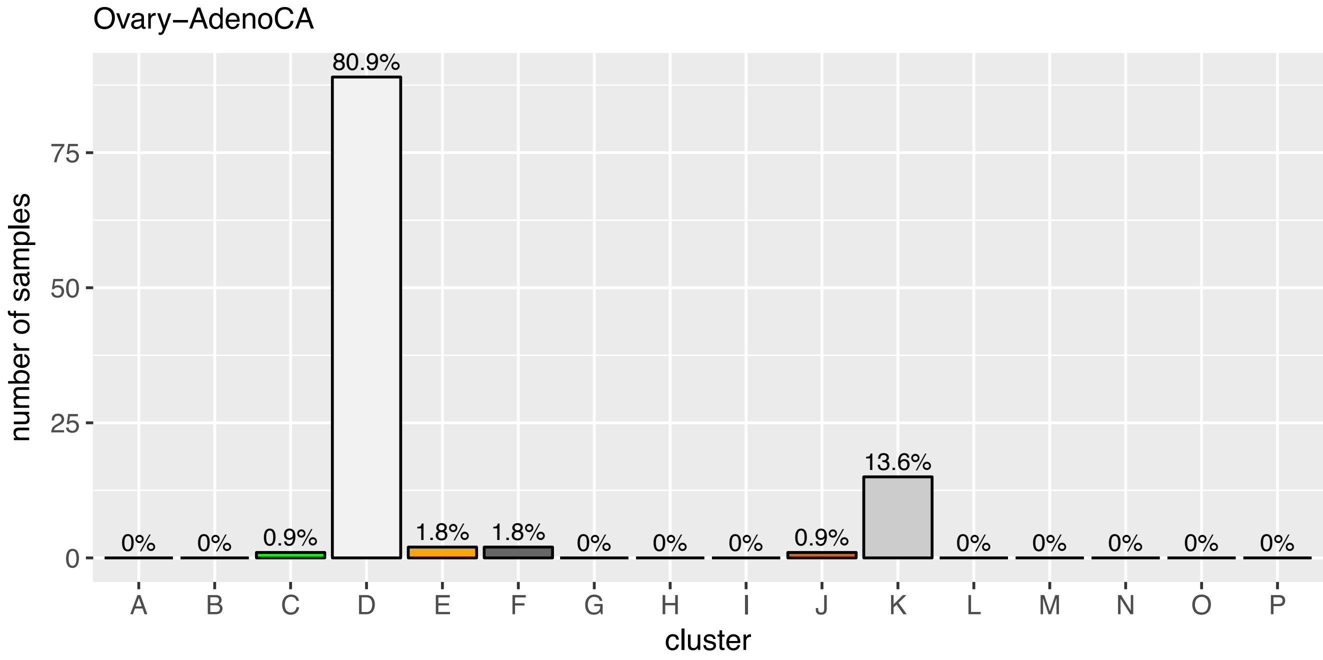
Ovary−AdenoCA
number of samples
0
25
50
75
80.9%
0%
0%
0.9%
1.8%
1.8%
0%
0%
0%
0.9%
13.6%
0%
0%
0%
0%
0%
A
B
C
D
E
F
G
H
I
J
K
L
M
N
O
P
cluster

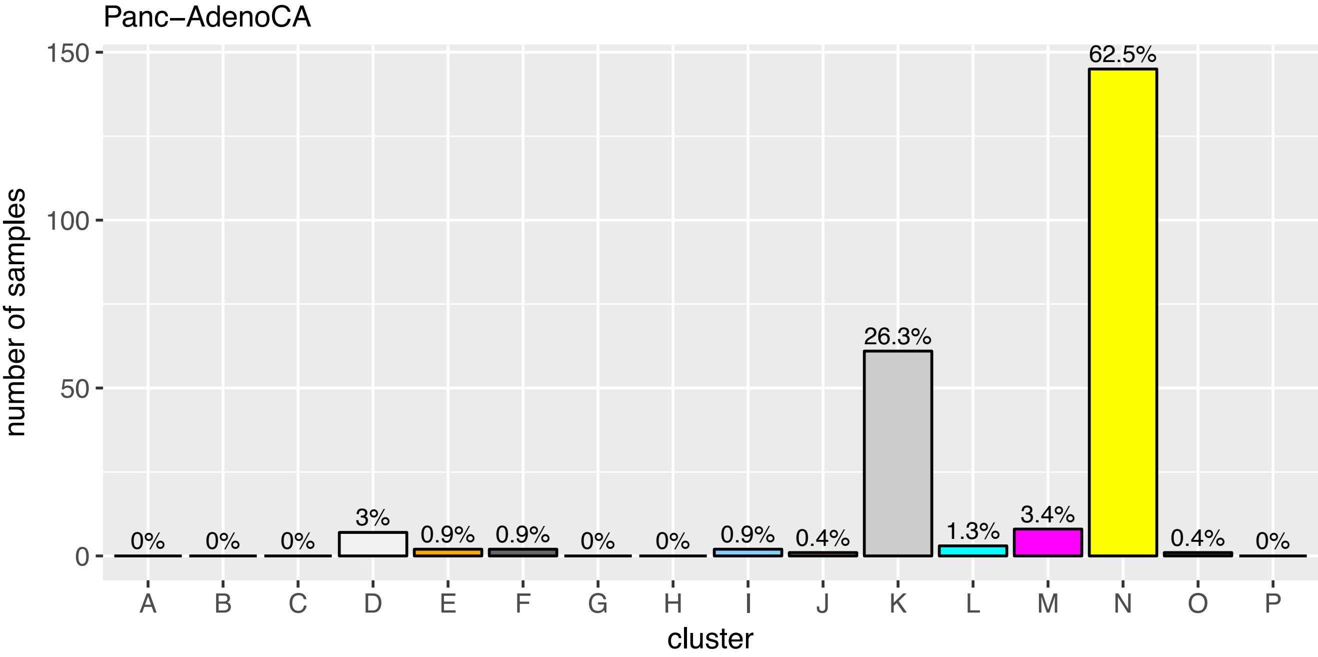
Panc−AdenoCA
number of samples
150
100
50
0
0%
0%
0%
3%
0.9%
0.9%
0%
0%
0.9%
0.4%
26.3%
1.3%
3.4%
62.5%
0.4%
0%
A B C D E F G H I J K L M N O P
cluster

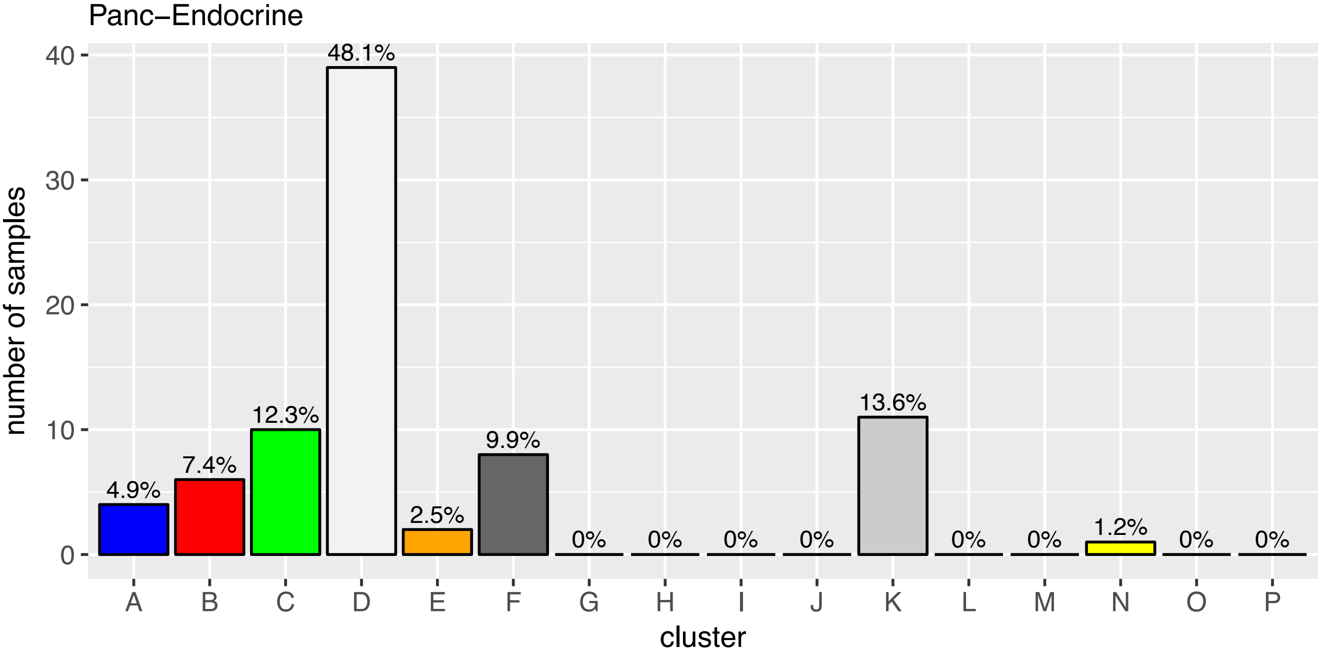
Panc−Endocrine
number of samples
40
30
20
10
0
4.9%
7.4%
12.3%
48.1%
2.5%
9.9%
0%
0%
0%
0%
13.6%
0%
0%
1.2%
0%
0%
A B C D E F G H I J K L M N O P
cluster

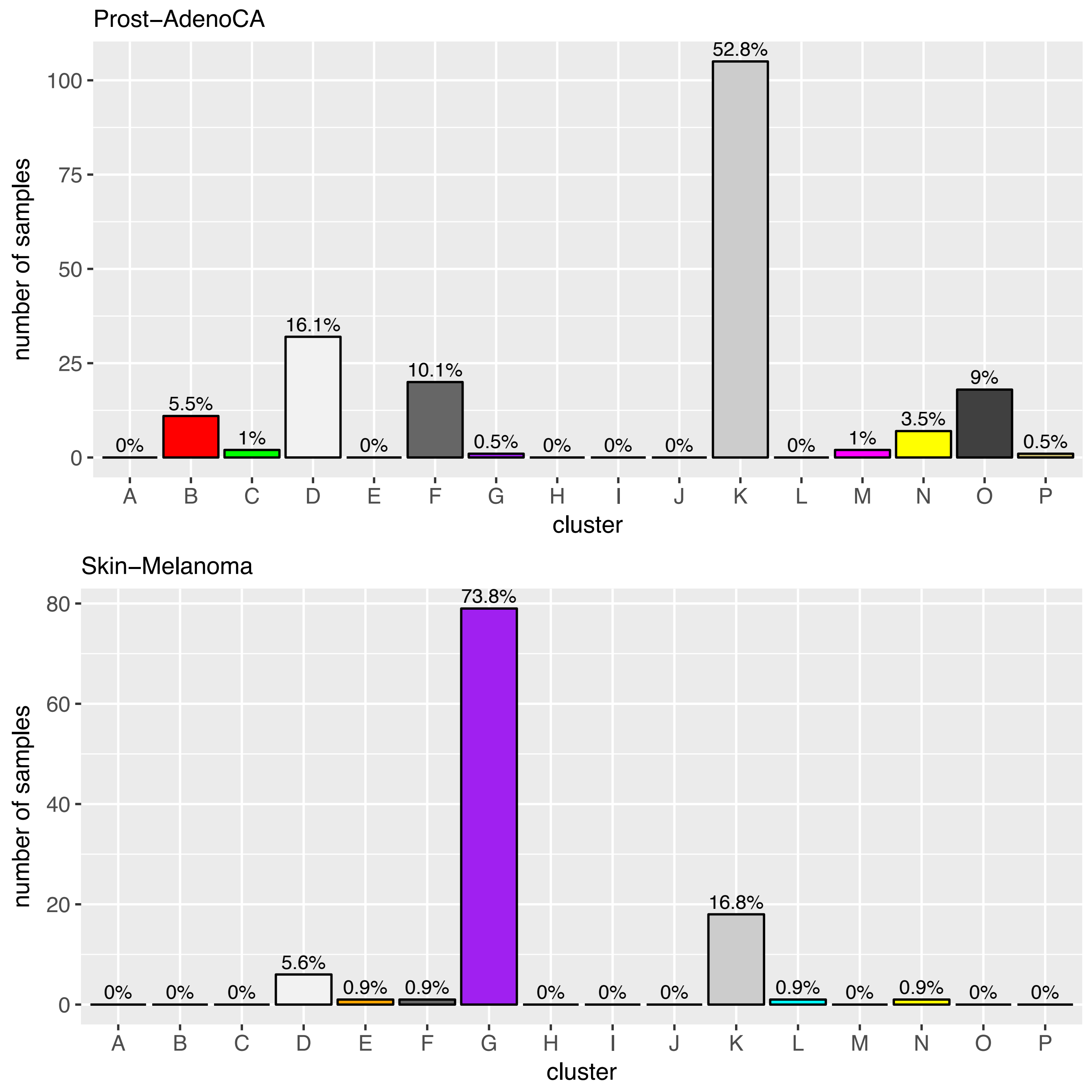

Prost−AdenoCA
number of samples
0
25
50
75
100
0%
5.5%
1%
16.1%
0%
10.1%
0.5%
0%
0%
0%
52.8%
0%
1%
3.5%
9%
0.5%
A B C D E F G H I J K L M N O P
cluster
Skin−Melanoma
number of samples
0
20
40
60
80
0%
0%
0%
5.6%
0.9%
0.9%
73.8%
0%
0%
0%
16.8%
0.9%
0%
0.9%
0%
0%
A B C D E F G H I J K L M N O P
cluster

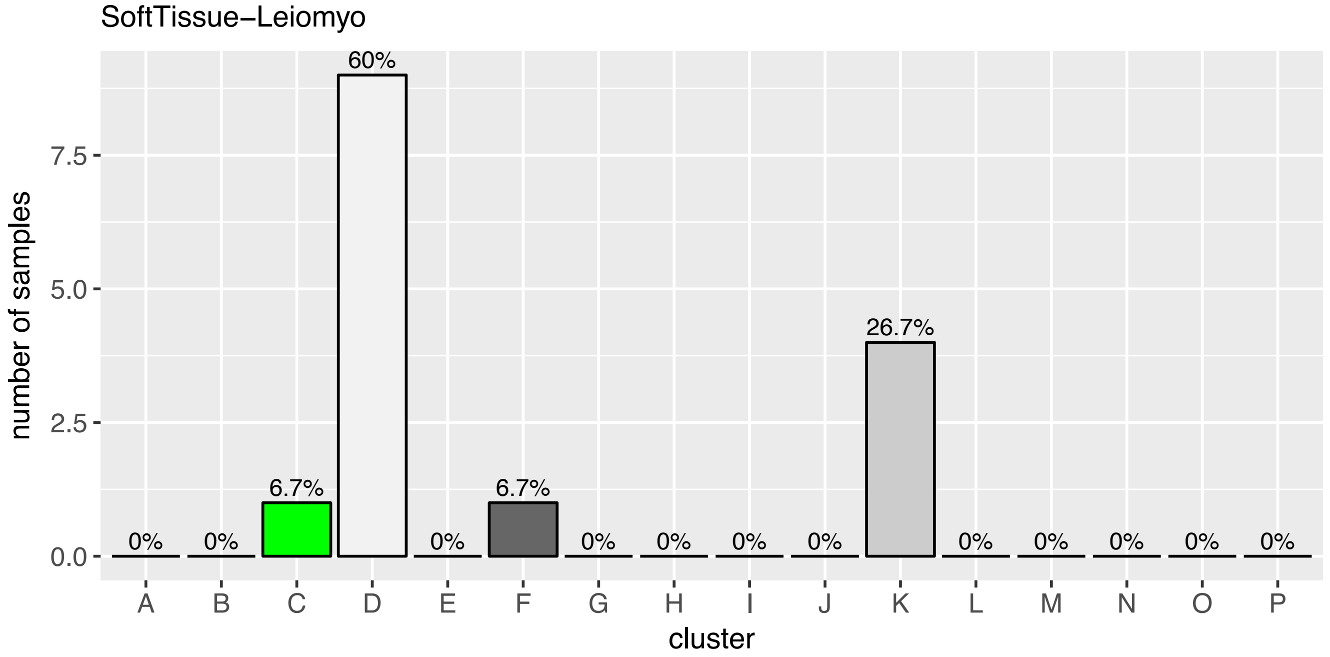

SoftTissue−Leiomyo
number of samples
0.0
2.5
5.0
7.5
0%
0%
6.7%
60%
0%
6.7%
0%
0%
0%
0%
26.7%
0%
0%
0%
0%
0%
A
B
C
D
E
F
G
H
I
J
K
L
M
N
O
P
cluster

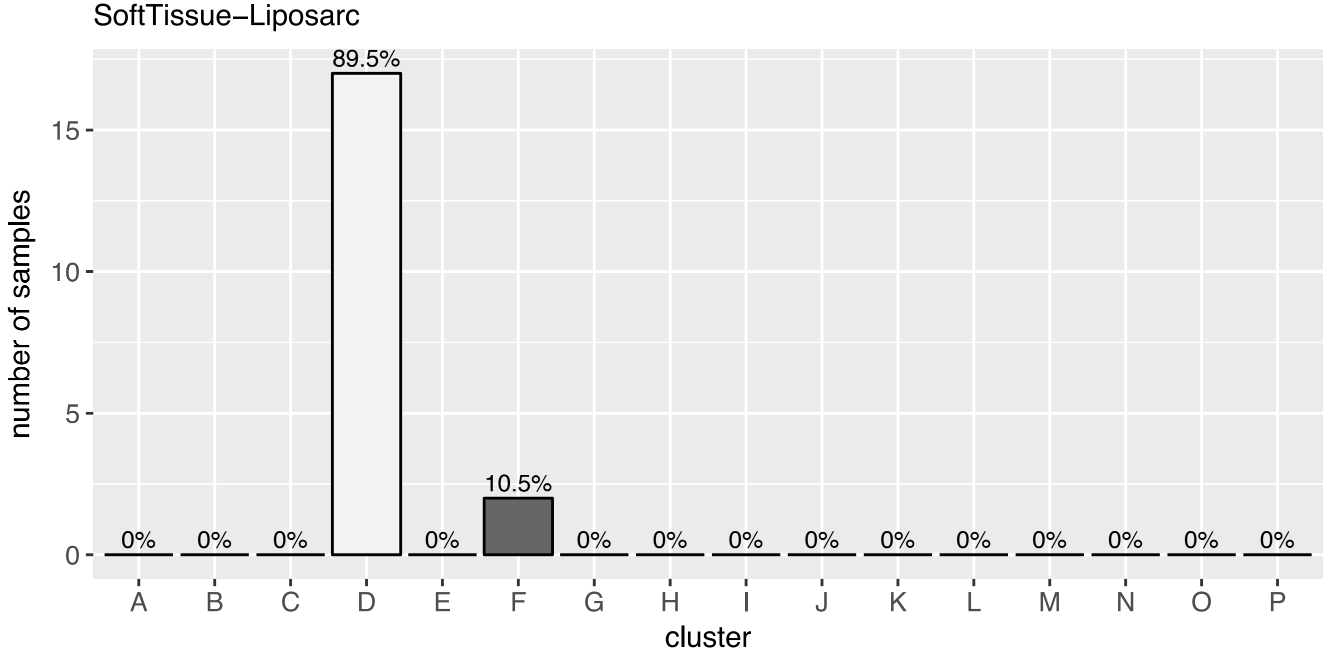

SoftTissue−Liposarc
number of samples
0
5
10
15
0%
0%
0%
89.5%
0%
10.5%
0%
0%
0%
0%
0%
0%
0%
0%
0%
0%
A
B
C
D
E
F
G
H
I
J
K
L
M
N
O
P
cluster

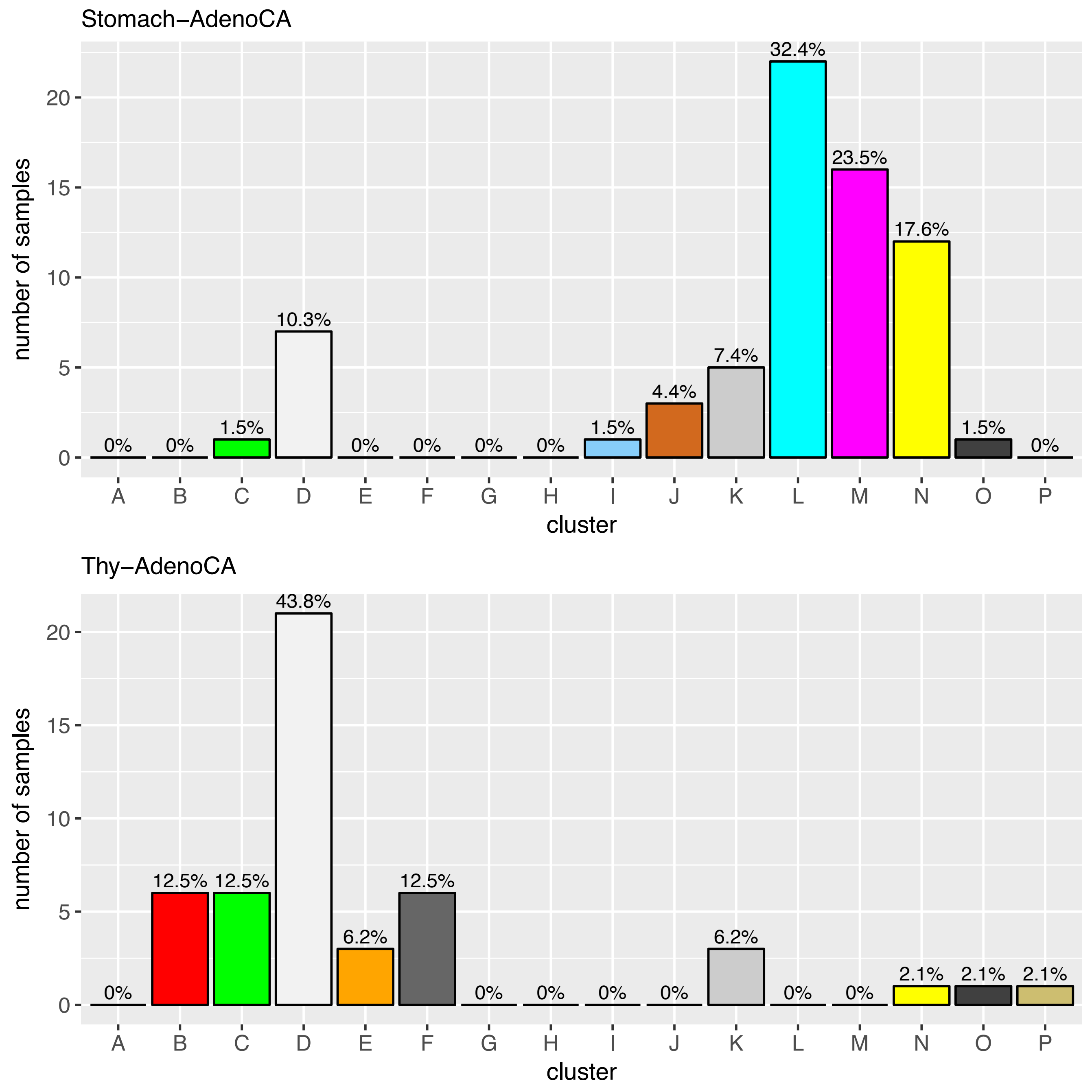
Stomach−AdenoCA
number of samples
0
5
10
15
20
0%
0%
1.5%
10.3%
0%
0%
0%
0%
1.5%
4.4%
7.4%
32.4%
23.5%
17.6%
1.5%
0%
A B C D E F G H I J K L M N O P
cluster
Thy−AdenoCA
number of samples
0
5
10
15
20
0%
12.5%
12.5%
43.8%
6.2%
12.5%
0%
0%
0%
0%
6.2%
0%
0%
2.1%
2.1%
2.1%
A B C D E F G H I J K L M N O P
cluster

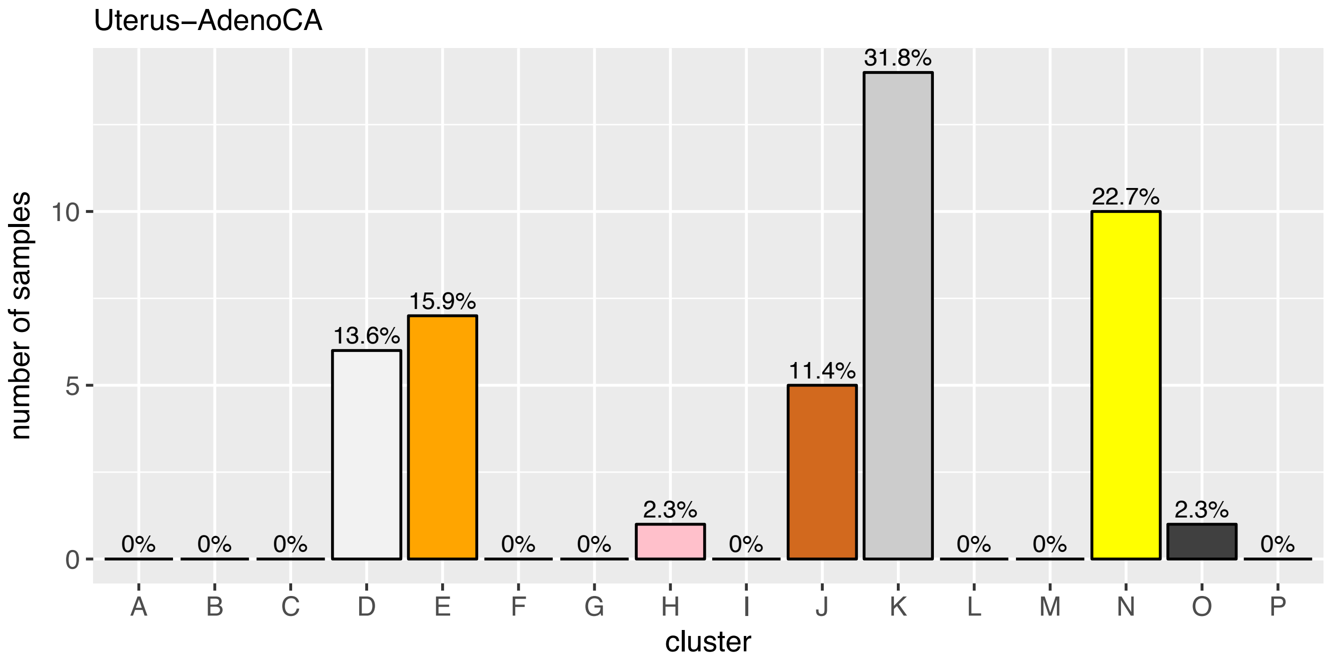

Uterus−AdenoCA
number of samples
0
5
10
0%
0%
0%
13.6%
15.9%
0%
0%
2.3%
0%
11.4%
31.8%
0%
0%
22.7%
2.3%
0%
A
B
C
D
E
F
G
H
I
J
K
L
M
N
O
P
cluster
